# Spatially resolved land and grid model of carbon neutrality in China

**DOI:** 10.1073/pnas.2306517121

**Published:** 2024-02-26

**Authors:** Da Zhang, Ziheng Zhu, Shi Chen, Chongyu Zhang, Xi Lu, Xiliang Zhang, Xiaoye Zhang, Michael R. Davidson

**Affiliations:** ^a^Institute of Energy, Environment and Economy, Tsinghua University, Beijing 100084, China; ^b^School of Environment, State Key Joint Laboratory of Environment Simulation and Pollution Control, Tsinghua University, Beijing 100084, China; ^c^State Key Laboratory of Severe Weather, Chinese Academy of Meteorological Sciences, Beijing 100081, China; ^d^Department of Mechanical and Aerospace Engineering, University of California San Diego, San Diego, CA 92093; ^e^School of Global Policy and Strategy, University of California San Diego, San Diego, CA 92093

**Keywords:** renewable energy, land use, power systems modeling, carbon neutrality, China

## Abstract

China has committed to achieve net carbon neutrality by 2060 to combat global climate change, which will require unprecedented deployment of negative emissions technologies, renewable energies (RE), and complementary infrastructure. At terawatt-scale deployment, land use limitations interact with operational and economic features of power systems. To address this, we developed a spatially resolved resource assessment and power systems planning optimization that models a full year of power system operations, sub-provincial RE siting criteria, and transmission connections. Our modeling results show that wind and solar must be expanded to 2,000 to 3,900 GW each, with one plausible pathway leading to 300 GW/yr combined annual additions in 2046 to 2060, a three-fold increase from today. Over 80% of solar and 55% of wind is constructed within 100 km of major load centers when accounting for current policies regarding land use. Large-scale low-carbon systems must balance key trade-offs in land use, RE resource quality, grid integration, and costs. Under more restrictive RE siting policies, at least 740 GW of distributed solar would become economically feasible in regions with high demand, where utility-scale deployment is limited by competition with agricultural land. Effective planning and policy formulation are necessary to achieve China’s climate goals.

## Significance

To support China’s goal of achieving carbon neutrality by 2060, we find that 2 to 4 terawatts are needed each for wind and solar power, eight to ten times its 2022 installations. A highly spatially resolved model reflecting key trade-offs in land availability per current government policies and grid integration shows that 80% of solar and 55% of wind should be constructed within 100 km of major load centers. The model also suggests that terawatt-level energy storage should be deployed from scratch, and ultra-high voltage inter-provincial transmission should double/triple its current size to ensure sufficient power supply. When land use is subject to even tighter policy restrictions, at least 25% of solar power should be distributed generation.

In order to keep global average temperature rise to within 1.5 to 2 °C, global anthropogenic CO_2_ emissions must reach net zero by around mid-century and become net negative in the second half of the century (1). Globally, this could imply a ten-fold increase in renewable energy capacity from 2020 to 2050 (2). As the world’s largest greenhouse gas (GHG) emitter and energy consumer, China’s energy sector transformation is at the heart of this challenge. The announcements by the government of China to peak carbon emissions in 2030 and achieve net economy-wide carbon neutrality by 2060 can be consistent with these goals, depending on the peaking level and pace of subsequent reductions (3–5). The electricity sector is central, as fossil fuel substitutes are more widely available in this sector, cost-effective decarbonization options in other sectors (e.g., transportation) often rely upon extensive electrification, and net-zero pathways often rely upon unprecedented deployment of negative emissions technologies (NETs), including in the power sector. China has already deployed wind and solar at rates far exceeding other countries, combined approximately 80 GW/yr from 2015 to 2022, but this pace will need to be accelerated to achieve these long-term targets (6). Understanding the impact of this renewable energy build-out on land use, given the large land footprints of these facilities and the potential for land competition, has important policy implications and could generate unforeseen constraints on the road to net-zero.

Modeling the composition and location of China’s renewable energy and complementary infrastructure reveals key methodological challenges in addressing this coupled land and grid problem. Individual energy resource assessments with a high degree of spatial granularity in deployment potential and variation in supply profiles show that onshore wind, offshore wind, and solar could all contribute to a net-zero energy system (7–10), but factors such as grid integration, geographic siting, and economic trade-offs among different technologies remain important (11–16). Comparisons of economy-wide integrated assessment models (IAMs) to achieve 1.5 °C from the ADVANCE Synthesis Project reveal substantial variation in renewable energy deployment by 2060, ranging from 1,000 to 5,500 GW of wind to 1,300 to 11,000 GW of solar for central cases (3, 17). Similarly, capacity expansion models focusing on long-term renewable energy development in China project 500 to 2,300 GW for wind and 250 to 5,000 GW for solar by mid-century (16, 18, 19). Some variation in IAMs is due to their aggregated model structure and coarse representation of spatial–temporal dynamicsof renewable energy integration. Electricity systems models incorporate greater fidelity in renewable energy integration with methodological approaches differing in terms of computational trade-offs between the complexity of power system representation and the geographic representation of renewable energy resource profiles and siting. Studies on deep decarbonization in China have emphasized power system constraints with typically only one or a few resource profiles per region, showing a higher reliance on solar out to 2050 compared to IAMs (18, 20–24). Alternative approaches applied outside of China retain diversity in renewable energy profiles and siting while reducing the number and complexity of power system constraints (25).

Analyses of high-penetration renewable energy scenarios demonstrate the importance of modeling several key features. First, in terms of operational requirements, diurnal and seasonal balancing needs as well as the availability of firm low-carbon sources have a large impact on system configurations and costs and demonstrate the need for modeling a long chronological time series, such as over a full 8,760-h of a representative year (26–30). Second, changes in transmission network topology can alter the cost-effectiveness of variable renewable energy (VRE) resources, i.e., wind and solar, in particular by reducing the costs of connecting resources distant from demand centers and managing intermittency over large geographic scales (30, 31). Third, the land use footprint of wind and solar at large-scale deployment can constrain deployment in high-quality resource areas and force trade-offs among competing land uses, such as agriculture and conservation, and other regulatory restrictions (25, 32, 33). Raising the share of wind and solar generation in China’s electricity system from roughly 15% today to a dominant share thus entails a number of interrelated questions: Where are there sufficient land resources to deploy terawatt (TW)-scale wind and solar systems? How much energy storage and transmission will be required? How much will this new system cost?

We develop a temporally and spatially resolved model, the Renewable Energy Siting and Power-system Optimization (RESPO) model, to explore the coupled land use and grid integration problem of large-scale renewable energy deployment, using the year 2060 when China would have already achieved its carbon neutrality target as a snapshot analysis. The RESPO model co-optimizes wind (onshore and offshore), solar (utility-scale and distributed), transmission (intra-provincial connections and inter-provincial corridors), and storage (pumped hydroelectric storage, battery, and long-duration energy storage), subject to meeting China’s 2060 carbon neutrality target supported by a power system as a carbon sink. Economy-wide models of China’s carbon neutrality pathways form boundary conditions for exogenous electricity parameters such as demand (34). Provincial zones are used for demand balancing and are further grouped into six main grid regions with reserve coordination: Northwest, Northeast, North, East, Central, and South. We further include negative sinks (bio-energy with carbon capture and sequestration, BECCS) that can help achieve about 550 Mt/yr negative emissions to offset other hard-to-abate sectors (34). A full year of power system constraints and geographically disaggregated renewable energy resource profiles and siting are accommodated computationally by modeling different classes of generators as “layers” with distinct operational characteristics instead of as individual units. Highly resolved land use siting criteria are derived from a detailed land use/cover dataset developed for China and an analysis of current government policies encouraging or discouraging renewable energy development. We model scenarios reflecting increasing tensions among land use priorities (*SI Appendix*, section 2.3). Combining geospatial, engineering, and economic features of renewable energy resources, we reveal facets of the challenge facing China’s energy planners on the road to net-zero, including siting wind and solar given sub-provincial load and transmission infrastructure, and trade-offs among different renewable energy options (e.g., utility- and distributed-scale solar). This model allows us to probe scenarios and implications of a terawatt-scale carbon-negative power system from a coupled land and grid perspective.

## Results

### Wind and Solar Development to Meet Carbon Neutrality.

**Fig. 1.**
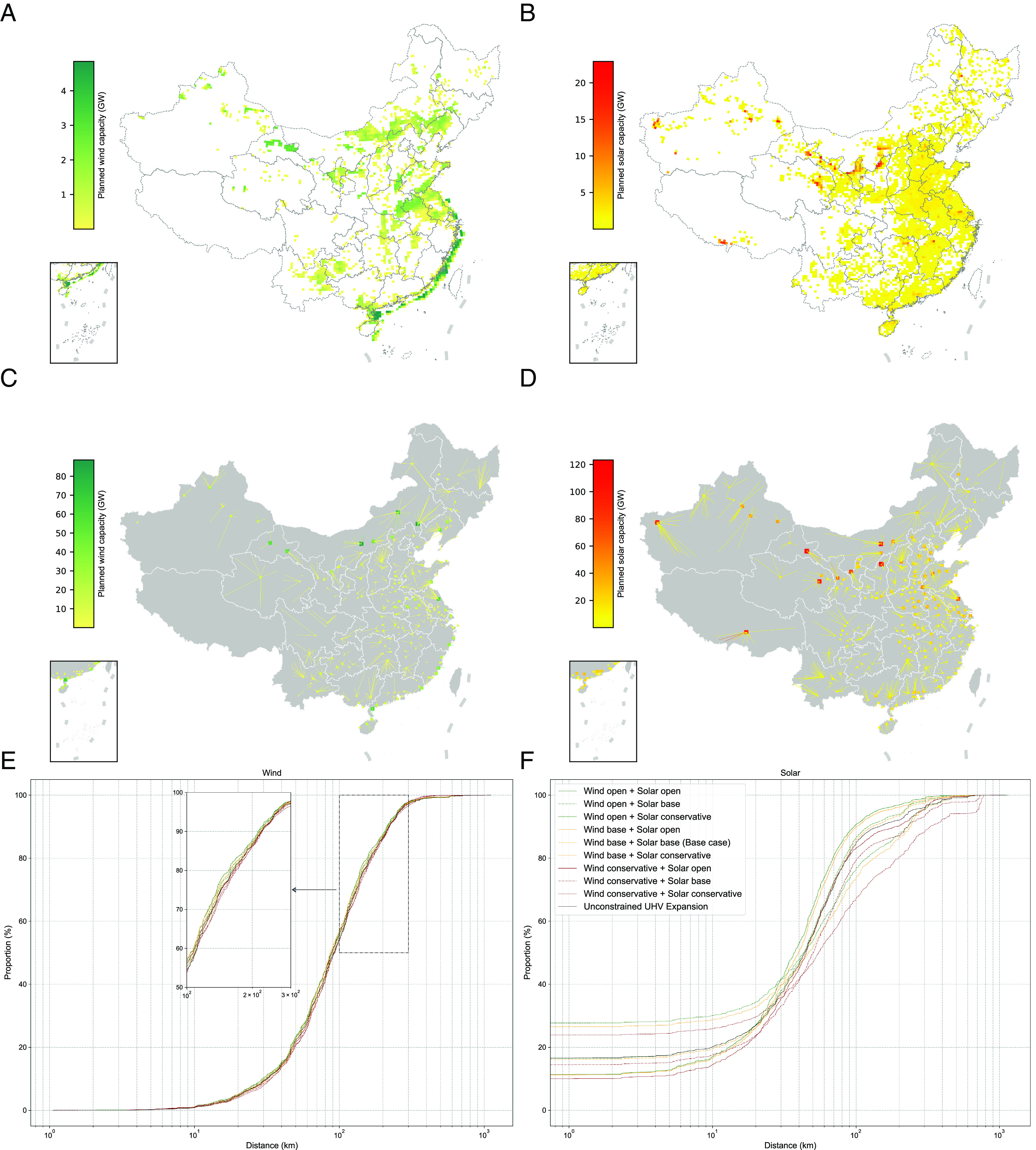
Optimized deployment of onshore, offshore wind and solar (utility-scale + distributed solar) in 2060. Planned capacity (GW, new+existing) of wind (*A*) and solar (*B*) by cell. Total capacity of wind (*C*) and solar (*D*) connected to a specific load center. Cumulative distribution of total distance (km) of spur and trunk lines that connect wind (*E*) and solar (*F*) cells (weighted by planned capacity) to their corresponding load center (distributed solar is modeled with zero connection distance). The land use setting for both wind and solar in the “Unconstrained UHV Expansion” scenario is the base case.

The base case results of wind and solar deployment in 2060 are shown in Fig. 1 *A* and *B*, including existing installations (assuming any retirements prior to 2060 are replaced) and new builds. We find 2.0 TW of wind and 3.9 TW of solar are installed, resulting in 6.0 and 5.3 PWh, respectively, and contributing to 65% of total annual generation. Wind installations are deployed throughout the country, including expanding from centers of existing deployment, which reflects the higher quality and lower costs for transmission connection and evacuation nearby already exploited resources. New, large wind power concentrations are found in Central and East China closer to demand centers, in line with previous research that showed the cost-effectiveness of wind deployment even in these lower quality areas in order to reduce transmission congestion (11, 35). Offshore wind is deployed at 416 GW, showing that it can play a role in certain coastal regions to meet low-carbon goals as has been identified elsewhere (12).

Solar deployment of 3.9 TW exhausts much of the suitable land for developing VRE (hereafter as suitable land) in eastern provinces where electricity demand is high and agricultural area (largely excluded from solar siting) takes up a large fraction, see *SI Appendix*, section 2.3 for suitable land calculations. For example, solar installations occupy more than 80% of suitable land in Anhui (99%), Zhejiang (98%), Jiangsu (86%), and Shanghai (80%), see *SI Appendix*, Fig. S17. Some new concentrations of solar capacity are also found in provinces that have few load centers but are less land-constrained, e.g., Xinjiang, Gansu, and West Inner Mongolia. Although previous research showed a huge physical potential for solar, up to 141 TW, mostly in Northwest and West Inner Mongolia, based on land and irradiance (14), our analysis finds that North, Central, and East China, which host most load centers, can accommodate the majority of solar (58%) even at slightly reduced irradiance. Distributed solar, which is assumed to have higher investment costs but no transmission connection costs, is deployed at 632 GW when accounting for remote-sensed estimates of suitable urban and built-up areas for deployment.

We consider two types of intra-provincial transmission lines connecting VRE cells to the local grid: spur lines connecting wind and solar resources to the nearest major substation, and trunk lines from the substation to the nearest major load center (30). Substations are assigned to county seats, and load centers are either defined by China’s long-term planning (36) or existing ultra-high voltage (UHV) terminals. Wind (68%) and solar (67%) capacity deployed in 2060 will be largely concentrated in major load-serving regions, such as North, Central, East, and South China, which together comprise 82% of total demand, despite the fact that Northwest and Northeast China have better wind and solar resources. Large wind and solar bases (greater than 80 GW per load center) are mostly located in Xinjiang, Gansu, East and West Inner Mongolia, where abundant VRE resources exist but the intra-provincial connection distance is significantly longer than other regions. This demonstrates the impact of VRE grid-connection to load centers on modeled build-out locations, typically ignored in long-term power sector decarbonization planning models (16, 18), as shown in Fig. 1 *C* and *D*. Nationally, over 80% of solar and 55% of wind is constructed within 100 km of major load centers and more than 80% of both wind and solar resources are located within 200 km of load centers, as seen in Fig. 1 *E* and *F*.

**Fig. 2.**
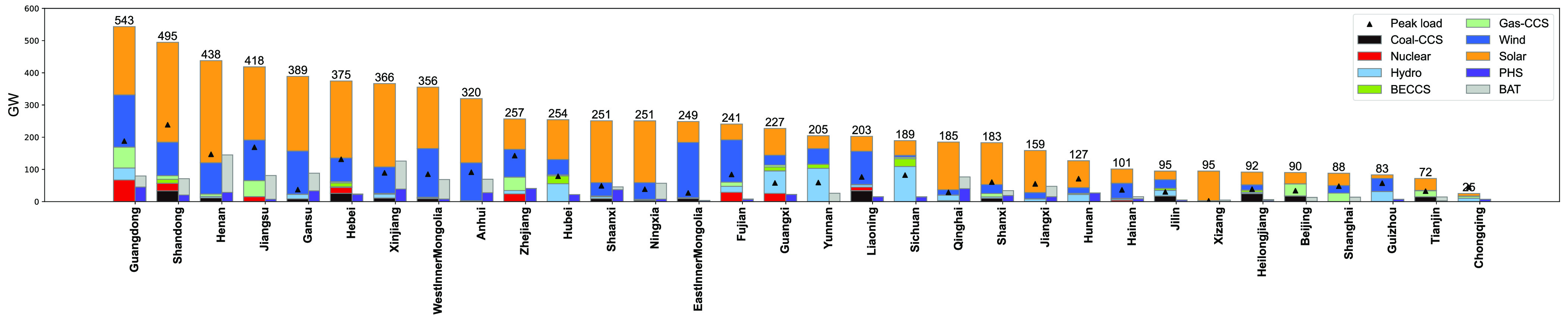
Capacity (firm generators and wind, solar, PHS, BAT) and peak load by province in 2060. Peak load refers to the maximum demand over the year.

Province-level results in Fig. 2 show larger wind, solar, and storage deployment where firm low-carbon resources are small relative to demand. Nationally, we assume that the following firm low-carbon resources are present in 2060 in China: 248 GW of combined heat and power (CHP) coal with carbon capture and sequestration (CCS), 110 GW of bio-energy with CCS (BECCS) operating as a NET, 218 GW of nuclear, 580 GW of hydropower, and 320 GW of natural gas with CCS (NG-CCS). These capacity and spatial distribution assumptions are driven by an assessment of capacity planning methods in China, which include both cost and noncost components: maximum exploitation of large-scale facilities according to resource or siting constraints (hydropower and nuclear), utilizing sunk infrastructure for cost-effective coupling with other sectors (CHP-CCS), necessary negative sink requirements (BECCS), and expected expansion of natural gas facilities nearby urban areas to address air pollution and flexibility needs (NG-CCS) (see *SI Appendix*, section 3.2 for more information on the determination of these parameters and the full provincial breakdown). For example, medium-sized Anhui province has only 2 GW of firm resources and installs 118 GW of wind, 200 GW of solar, 28-GW pumped hydroelectric storage (PHS), and 42 GW of battery storage (BAT). Sichuan, with comparable peak demand but plentiful hydropower resources, installs 6 GW of wind, 45 GW of solar, 15 GW PHS, and 350 MW of battery storage. At the national level, PHS of 538 GW (8-h storage) and battery capacity of 740 GW (4-h storage) are installed, about 22% of the combined capacity of wind and solar, comparable to results from previous work on the United States (30).

**Fig. 3.**
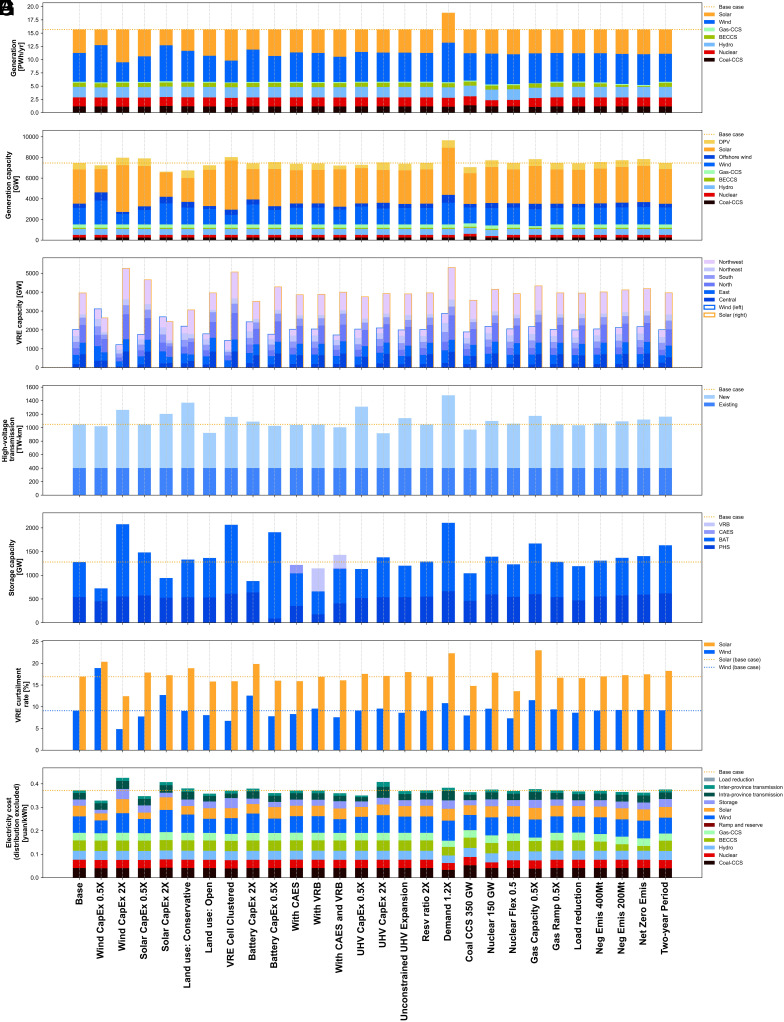
Sensitivity analysis on generation, capacity, transmission, curtailment, and costs. (*A*) Annual dispatched energy (PWh). (*B*) Installed power capacity (GW) of generation. (*C*) VRE (wind and solar) capacity (GW) by grid. (*D*) High-voltage transmission capacity (TW-km). (*E*) Power capacity (GW) of storage. (*F*) Annual wind and solar curtailment rates. (*G*) Average electricity cost (yuan/kWh).

Curtailment rates nationally are 9.2% and 16.0% for wind and solar, respectively, with significant variation among provinces: e.g., the highest wind curtailment rate occurs in Jilin in the far northeast (22.3%), the highest solar curtailment rate occurs in West Inner Mongolia and Tibet (∼30%), and the lowest wind and solar curtailment rates occur in Jiangxi at 2.8% and Chongqing at 4.4%, respectively. These rates are higher than today’s levels of 3% and 2% wind and solar curtailment, respectively (37), but in line with previous studies on China by mid-century, e.g., nationally 10 to 15% in ref. 16. More detailed results from the base case and a suite of sensitivity scenarios designed to test the robustness of the study’s major findings are illustrated in Fig. 3 (see *SI Appendix*, section 5 for scenario design).

Widespread electrification of transportation, industry, and buildings is expected to more than double electricity demand in 2020 to 15.4 PWh/yr by 2060 (34). However, if demand is 20% larger than the base case while holding firm low-carbon resources unchanged (see scenario “Demand 1.2X” in Fig. 3), wind, solar, and battery storage capacities would increase by 43, 34, and 95%, respectively. This overbuilding relative to demand also results in higher curtailment rates: 11.2% for wind and 21.9% for solar. The increase in curtailment occurs primarily at midday in autumn and winter when solar is plentiful (*SI Appendix*, section 6.4). We note that even with this additional wind and solar capacity, the shares of wind (66%) and solar (61%) capacity deployed in regions with more load centers (North, Central, East, and South China) remain almost unchanged (compared to 68% and 67% for wind and solar in the base case). Wind and solar deployment maps for all sensitivity scenarios are shown in *SI Appendix*, section 6.3.

### Supporting Grid Infrastructure: Transmission and Storage.

**Fig. 4.**
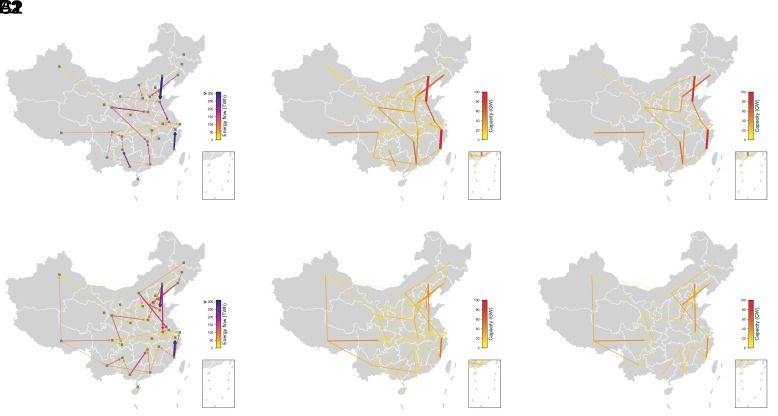
Results of inter-provincial transmission line in the base case (*A1*, *B1*, and *C1*) and “Unconstrained UHV Expansion” scenario (*A2*, *B2*, and *C2*). (*A1* and *A2*) Inter-provincial transmission flows (TWh). (*B1* and *B2*) Existing plus new transmission capacity (GW). (*C1* and *C2*) Newly added capacity (GW) by the model.

China boasts the world’s largest UHV grid—for our purposes, defined as all DC lines and all AC lines greater than 500 kV—and a rapidly growing 500-kV AC network. The existing network proves beneficial for integrating TW-scale wind and solar systems, though new capacity is still required for cost-effective deployment. The model captures three categories of transmission upgrades: 220-kV intra-provincial lines connecting VRE cells with substation and load centers including both spur and trunk lines (30), and inter-provincial lines connecting load centers between provinces. For inter-provincial transmission, we assume lines can be built between provinces with existing lines, and the highest existing voltage line or 500 kV (whichever is largest) is selected for new lines built in the future (*SI Appendix*, section 3.4.1). Results for transmission flows and newly built transmission capacity are shown in Fig. 4 (see *SI Appendix*, section 6.6.2 for all sensitivity scenarios). Existing pathways from west to east, such as Ningxia–Shandong (expanded from 4 GW today to 26 GW), are matched with new parallel pathways, such as Gansu–Henan (40 GW). Large increases in transmission capacity also occur from north to south, e.g., from East Inner Mongolia to Shandong (expanded from 10 GW today to 90 GW). This corridor brings largely wind resources in one of China’s windiest regions to the country’s largest power consumer in 2060. Another north–south corridor opens up in the south, connecting Fujian (with a large nuclear fleet and offshore wind deployment) and Zhejiang (a major load center). Distance-weighted transmission capacity is increased by 25%. Inter-provincial transmission line costs affect how much transmission is built and the distribution of wind and solar deployment. Increasing (decreasing) MW-km costs by a factor of two results in changes in new transmission capacity builds of −15% (+26%), but only +4% (+1.2%) and −1% (−5%) for wind and solar capacity, respectively, and modest changes of −0.6% (+3.8%) and +14% (−17%) for PHS and battery storage capacity, respectively.

We design a more flexible inter-provincial transmission line expansion scenario, permitting new transmission lines to be built between any two provinces regardless of existing connections, with the exception of Tibet and Hainan, which are allowed to connect to neighboring provinces only, owing to the challenges of estimating costs and building lines over heavily mountainous terrain and over the sea, respectively. A pre-optimized procedure considering distance and unit cost is used to determine the choice of AC or DC and voltage level for each line (16), see *SI Appendix*, section 3.4.2 for details. The main findings in the base case stay robust, and over 80% of wind and 55% of solar deployments are still within 100 km of major load centers (Fig. 1 *E* and *F*). Some transmission corridor capacities change, though total transmission capacity is nearly constant (Figs. 3 and 4). Total system costs decrease by 1.0%, installed capacity of BAT decreases by 10%, and wind and solar development decreases slightly by 1.1% and 0.7%, respectively.

Managing country-level variability of renewable energy requires transmission networks to balance energy and reserves. Some corridors operate almost entirely in one direction and have high congestion rates (defined as the percentage of the time transferring at full capacity), such as Guangxi–Hunan (32.3%) and Sichuan–Jiangxi (28.5%). Others, such as Anhui–Jiangsu, have large total transmission in both directions and are rarely congested, reflecting an important balancing role (*SI Appendix*, section 6.6.1). Increasing reserve requirements (from 5% to 10% of load and VRE integration, see scenario “Resv ratio 2X” in Fig. 3) causes a slight increase in solar curtailment but has limited impacts on system costs and other outcomes.

Storage plays two seasonally dependent roles in the 2060 system, providing reserves for peak demand periods and arbitrage to shift energy between high and low supply hours. Previous studies on the role of storage in VRE-dominated systems highlight the increasing importance of reserves (as conventional generators providing reserves are reduced and VRE-related reserve requirements increase), as well as long-duration energy storage (LDES) for extended periods of low VRE availability (28, 29, 38, 39). In the base case, we model both short-duration battery storage (4-h) and medium-duration PHS (8-h), and find total installed capacities reaching 1,278 GW, 54% of peak demand. Curtailment rates are generally higher during the shoulder seasons (spring and autumn) when demand is lower (*SI Appendix*, sections 6.4 and 6.5). We also consider the inclusion of LDES with either or both compressed air energy storage (CAES, 20-h duration) and a vanadium redox-flow battery (VRB, 10-h duration). When both are available, they are built at about 20 and 278 GW, respectively (battery and PHS installation reduced to 406 and 727 GW, respectively), displaying some complementarities in reducing the need for transmission.

### Firm Low-Carbon Power.

Firm low-carbon power has been shown to be an important component of a low-cost decarbonized power system (27). Notwithstanding local environmental impacts, we expect China to exploit its remaining conventional hydropower resources by 2060 (22). Additionally, natural gas with CCS could play an important role in addressing air pollution nearby urban areas as well as meeting flexibility needs. These two generators provide the greatest flexibility in balancing intermittent wind and solar resources.

Reducing natural gas requires additional build-out of VRE, transmission, and storage. For example, if only half NG-CCS capacity were built (160 GW, see “Gas capacity 0.5X” in Fig. 3), battery storage requirements would increase by 44% to 1,068 GW. Furthermore, with less flexible natural gas (reducing ramp rates from 50% of max capacity per hour to 25%/h, see “Gas Ramp 0.5X” in Fig. 3), wind and solar curtailment rates increase to 9.35 and 16.7%, respectively, with installations changing only slightly (+/− 0.1%).

China is currently the fastest-growing market for nuclear power, yet many obstacles have led to slower-than-anticipated development. New 3rd-generation (Gen-III) reactor designs are coming online and fourth-generation (Gen-IV) plants are now being tested. The largest long-run uncertainty for nuclear growth in China is whether nuclear plants are deployed inland. We assume growth in coastal nuclear only (218 GW) and moderate flexibility (minimum outputs of 85%) (30, 40). If nuclear becomes highly flexible, e.g., 50% minimum output (30), see “Nuclear Flex 0.5” in Fig. 3, VRE curtailment is reduced from 12.8 to 10.2%, with minimal changes to capacity deployment. If we assume a more conservative nuclear deployment (150 GW, see “Nuclear 150 GW” in Fig. 3), the share of nuclear in total supply would decrease from 10.2 to 6.5%, with wind and solar capacities increasing to 2,169 GW (+8%) and 4,136 GW (+6%), respectively, and limited changes in other technologies.

The only role for coal in China’s 2060 power system is envisioned in CHP-CCS to support its large district heating network in the north in our base case. Electric source heat pumps are increasingly low cost and could be deployed in a decentralized manner in rural and potentially many urban settings (41). Nevertheless, China has already built an extensive district heating network fed by large, centralized generators, and approximately 99% of the network is less than 25 y old (heating areas in 1998 and 2020 are about 0.1 billion m^2^ and 9.8 billion m^2^, respectively) (42, 43). Retiring this network completely by 2060 is likely unnecessary given its expected useful lifetime. Furthermore, it may not be cost-effective to displace central networks as global net-zero studies demonstrate the positive role of combined heat and power (44, 45). Therefore, we require all of the remaining coal CHP fleet to install CCS (248 GW).

Making thermal fleets more flexible is an important element of addressing current renewable energy integration challenges in China, and many options exist to improve the performance of coal plants (46). China’s central government has initiated a flexibility program, which aims to retrofit a significant share of its existing coal fleet by 2025 (47). In our base case, we take these CHP-CCS plants as must-run during the winter heating season (mid-November to mid-March) and allow them to operate flexibly (with minimum outputs of 15% if online) during the rest of the year. The average committed capacity outside of the heating season reaches about 100 GW, indicating some residual benefit of flexible coal for renewable energy integration.

We also introduce a scenario with higher coal CCS capacity (an additional 102 GW of non-CHP coal with no must-run hours, equivalent to about 10% of the capacity in 2020 for each province). We assume this additional 102 GW coal CCS costs the same as coal CHP with CCS capacity. As expected, this scenario has less wind (128 GW/6.4% less) and solar (385 GW/9.8% less) capacity compared to the base case. The system cost has a slight 1% decrease, see “Coal CCS 350 GW” in Fig. 3. However, this cost estimate may be optimistic because the marginal cost of carbon sequestration may increase following exploitation of the cheapest locations (48, 49).

### Cost Evolution of Low-Carbon Technologies.

We assume capital costs of VRE and energy storage continue to decline through 2060 owing to improvements in technology, soft costs and construction costs (*SI Appendix*, section 2.5): onshore wind 3,000 yuan/kW, 469 $/kW, 48% of 2020 (37), offshore wind 5,400 yuan/kW, 844 $/kW, 42% of 2020 (16), utility-scale solar 1,500 yuan/kW, 234 $/kW, 40% of 2020 (37), distributed solar 2,000 yuan/kW, 313 $/kW, 60% of 2020 (37), and battery 675 yuan/kWh, 106 $/kWh, 30% of 2020 (50).

As a sensitivity test, we bracket the range of possible relative costs by adjusting capital expenditure (CapEx) by factors of 0.5X and 2X separately for wind, solar, battery storage, and UHV transmission capacity. For example, reducing wind CapEx in half results in a 54% increase in wind capacity, a 35% decrease in solar capacity, and a 63.4% decrease in battery storage capacity (see “Wind CapEx 0.5X” in Fig. 3). Reducing solar CapEx in half results in a 12.9% decrease in wind capacity, a 17.8% increase in solar capacity, and a 22.5% increase in battery storage capacity (see “Solar CapEx 0.5X” in Fig. 3. Doubling wind CapEx leads to significantly larger solar deployment (5,243 GW, a 33% increase), see scenario “Wind CapEx 2X” in Fig. 3.

Storage deployment is more correlated with solar than wind, reflecting storage needs to manage strong diurnal trends in solar radiation. Wind (both offshore and onshore), because of its greater availability throughout the day, becomes more cost-effective as battery storage costs increase. In the base case, battery costs are expected to fall roughly in half from today’s levels. Compared to the base case, increasing (decreasing) battery costs by a factor of two results in changes to capacity of −68% (+146%) for battery storage, +19% (−84%) for PHS, +20% (−12.4%) for wind, and −11% (+8.3%) for solar, see “Battery CapEx 2X” (“Battery CapEx 0.5X”) in Fig. 3. Therefore, lower storage costs enable higher solar to wind shares in China’s carbon-neutral power system.

Transmission is in general used preferentially to balance wind resources. When wind CapEx decreases or solar CapEx increases, more inter-provincial transmission is built. Doubling solar costs (“Solar CapEx 2X” in Fig. 3) has a similar impact on newly built transmission capacity (+22%) as cutting transmission costs in half (+25%, “UHV CapEx 0.5X” in Fig. 3). An increase in transmission costs (see “UHV CapEx 2X” in Fig. 3) will increase offshore wind deployment (+31%) and distributed solar (+17%) and lead to more battery storage (+14%) for load balancing.

We estimate system cost in 2060 (excluding costs of local distribution) at 0.35 yuan/kWh (about 0.055 $/kWh). For reference, we validated the generation layers model by fixing 2020 capacities, demand and costs, and optimizing only operations. Based on this exercise, our model estimates present-day costs at 0.36 yuan/kWh, nearly the same as our 2060 results, indicating that China’s future power system may be cost-effective by today’s terms with assumed technological improvement, similar to previous results from studies on China (51) and India (52). In the extreme case of no further cost reduction of low-carbon technologies by 2060, the system cost per kWh would be about 50% higher (0.54 yuan/kWh). The system cost per kWh in all sensitivities is shown in Fig. 3*G*, showing that overall, uncertainties in cost assumptions on CapEx of low-carbon technologies affect the system cost more than land use, system operation (reserve requirements, nuclear flexibility, and cost of load reduction) or capital costs of storage. Transmission cost uncertainties have similar effects compared to CapEx of low-carbon technologies on total system cost. Quantities of CCS available as well as levels of negative emissions required have only a moderate effect on total costs, though can have a large impact on storage deployment and renewable energy curtailment. Taking a high capital cost scenario of various CCS technologies can increase total system costs by 4.3%.

### Carbon Sinks in Power.

Given the availability of low-cost zero-carbon substitutes, the electricity sector is expected to reach neutrality earlier than other sectors. In addition, economy-wide studies show that it is cost-effective for several hard-to-abate economic activities with high marginal costs of abatement to be offset by negative emissions elsewhere, in particular power (34, 53, 54). In this study, we model carbon sinks exogenously in order to meet a certain negative emissions target (−550 Mt/yr in the base case) adopted from an economy-wide decarbonization study (34), which used an integrated modeling approach to analyze China’s energy transition pathways to carbon neutrality. It projected that approximately 650 Mt CO_2_ of negative emissions would need to be provided annually from BECCS by 2060 (considering positive emissions from partial capture fossil fuel CCS), which is in line with more detailed analyses on BECCS potential (53, 54).

We treat BECCS as all-year must-run with a capacity factor (total power generation divided by theoretical generation operating at maximum capacity) of 0.8, adopted from ref. 55. If we assume a medium value (−850 g/kWh) of BECCS life-cycle GHG emissions factor (g/kWh), 110 GW of BECCS needs to be deployed, resulting in an annual generation of 770 TWh. The 110 GW BECCS capacity is allocated to specific provinces considering the transportation cost of carbon emissions from sources to sites of storage and the availability of biomass resources, see details in *SI Appendix*, section 3.2. BECCS generation requirements incorporate positive emissions from the partial capture rates of CHP-CCS and NG-CCS. Resulting negative emissions across the scenarios (except for 50% reduction in NG-CCS capacity scenario) vary slightly due to wind and solar deployment differences, between −539 and −552 Mt/yr.

For negative emissions targets of 400, 200, and 0 Mt/yr (see “Neg Emis 400 Mt”, “Neg Emis 200 Mt”, and “Net Zero Emis” in Fig. 3), BECCS generation is successively reduced and VRE capacity fills in with deployment increases of 84, 264, and 377, respectively.

### Increasing Land Use Tensions.

**Fig. 5.**
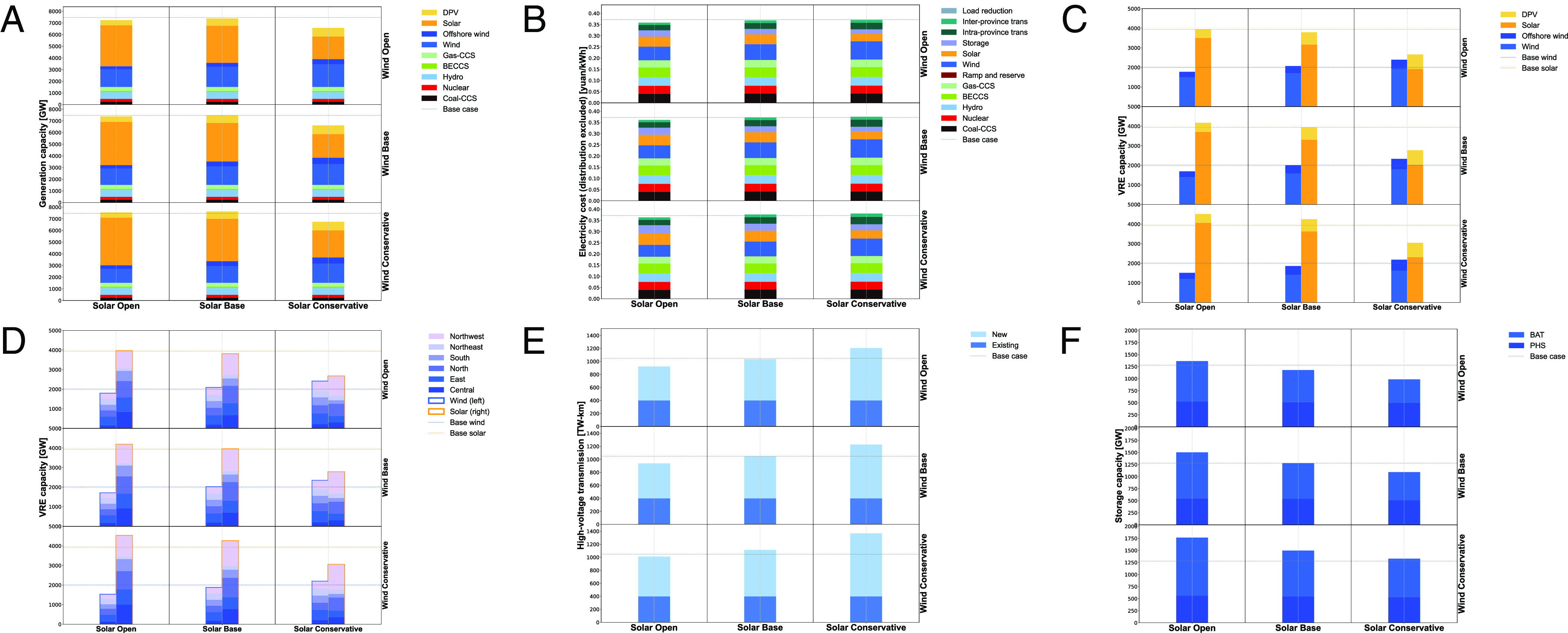
Results of 3 × 3 land use assumptions. (*A*) Capacity (GW) of generators. (*B*) Average electricity cost (yuan/kWh). (*C*) Capacity (GW) of VRE by grid region in China. (*D*) Capacity (GW) of wind (onshore + offshore) and solar (utility-scale + distributed). (*E*) High-voltage transmission capacity (TW-km). (*F*) Power capacity (GW) of storage.

We explore the sensitivity of model results to changes in land use policies for renewable energy development, reflecting possible evolution in government priorities for energy security, food security, and decarbonization. We use a pixel-level spatial resolution land use dataset (56) for China to estimate the suitable land area for developing VRE. This land use dataset classifies land into 25 types, including paddy/irrigated and dry cropland, etc. The suitable land area of each cell and energy type is calculated by aggregating its pixels’ area multiplied by the suitability score (0 to 100%), which represents the ratio of area suitable for VRE development to the area of a pixel based on a review of land use policies for renewable development and geographic characteristics such as slope and altitude, see details in *SI Appendix*, section 2.3. The base case reflects current policy guidance, and two additional scenarios (“conservative” and “open”) each for wind and utility-scale solar are formed to represent more strict or loose land use policies in the future, respectively, see more details in *SI Appendix*, section 2.3. For example, solar deployment on dry cropland has a suitability score of 5% in our base case, which falls to 0 in the “conservative” scenario and doubles to 10% in our “open” scenario. A full analysis is then performed, resulting in nine distinct land use scenarios (Fig. 5 and *SI Appendix*, section 6.2).

We find under different land use scenarios that the total generation capacity installed (Fig. 5*A*) can vary by −15.2% to +3.3%, while system costs (Fig. 5*B*) vary to a lesser degree (−3.6% to +2.1%). The most conservative land use scenario increases wind by 175 GW but reduces solar installations by 890 GW. Compared to land use scenarios for wind, solar land use scenarios show larger changes in aggregate solar and wind capacities (Fig. 5*C*) as well as their geographic distribution (Fig. 5*D*). As utility-scale solar siting assumptions become more conservative, distributed solar replaces utility-scale solar to a greater extent in high-demand and large agricultural provinces where competition for land is more pronounced: Henan, Shanxi, Shandong, and Anhui each have a high share of suitable land taken up by agriculture (*SI Appendix*, Figs. S17 to S25). This is consistent with findings in the base case that these provinces exploit most of their suitable solar potential. Furthermore, lower solar installations lead to larger inter-regional transmission and lower storage capacities (Fig. 5 *E* and *F*). By contrast, wind installations are less sensitive to land use assumptions, indicating sufficient availability of land (inclusive of agricultural land) to build wind turbines.

### Spatial and Temporal Variability in Renewable Resources.

Spatial characteristics of renewable energy create heterogeneity in generation output as a function of siting, and standard formulations usually aggregate renewable resource profiles into a small number of clusters per load zone to maintain computational tractability (16, 18, 24, 30, 52, 57). We compare our high spatial resolution modeling approach with a “VRE Cell Clustered” scenario which combines cells within each province into several clusters by VRE type based on their hourly capacity factor profiles and distance to load centers (see details in *SI Appendix*, section 2.7). Compared to the cell-level optimization, the clustered wind and solar scenario tends to concentrate build-out in certain areas in a province and thus cannot be used to readily infer siting locations, see *SI Appendix*, Fig. S39. More importantly, clustering substantially favors solar over wind in the aggregate, changing the optimal build-out of wind, solar, and battery capacities, by −28.9%, +29.2%, and +96.5%, respectively. By treating all cells within a cluster equivalently, clustering underestimates the costs of connecting VRE cells to the local grid. Within solar capacity, distributed solar is substantially decreased (−63%) as its cost advantage compared to utility-scale solar is very sensitive to its grid connection benefits.

Inter-annual variability of renewable energy potential can alter the optimized deployment of VRE (30). Forecasting beyond mid-century, of increasing concern is a secular decline observed in some of China’s most productive wind regions (58). The base case uses the 2015 historical weather year, which is in line with recent annual averages, and we consider a scenario with two consecutive weather years of 2015 and 2016 (2016 has 2% lower wind potential). Solar capacity factors exhibit much less variation from year to year. At the reduced wind potential, the optimal build-out of wind, solar, PHS, and battery capacities change by −0.5%, −0.2%, +14.5%, and +37.0%, respectively, see “Two-year Period” scenario in Fig. 3.

## Discussion

Achieving carbon neutrality in China by 2060 requires a massive transformation of the electricity sector by deploying a suite of zero- and negative-emissions generating technologies as well as complementary storage and transmission infrastructure. Wind and solar will need to be deployed at levels of 2,000 to 3,900 GW each, occupying much of the suitable land near load centers and leading to high rates of distributed RE deployment. Unprecedented storage deployments on the order of 1,000 GW are required to accompany them. These results have several implications for near- and long-term policy. First, land use will become increasingly contentious, an issue which to date has been relatively minor in China due to high-quality resources in areas without major land competition. In some eastern coastal provinces, nearly two-thirds of suitable land for utility-scale solar is utilized by 2060. Land policies must support this deployment while preserving agricultural and other uses. A significant capacity of distributed solar (632 GW in the base case, 740 GW under conservative land use assumptions) becomes cost-effective as a result.

Second, both reinforcements of existing transmission corridors and new corridors are needed to optimally balance increased VRE. China has already constructed the world’s largest UHV grid, but a near doubling of capacity (in terms of MW) of lines ≥ 500 kV will require careful planning. The configurations in terms of main corridors and predominant flow directions are largely unchanged across scenarios. However, capacities are sensitive to the cost evolution of other technologies, suggesting the need for flexible transmission plans in line with market changes.

Third, a prerequisite for realizing the benefits of the 2060 system developed here is progress on reforms to the markets, planning, and operations of China’s power sector. In 2015, China launched a new round of reforms aimed at improving efficiency, accommodating renewable energy, and reducing emissions (59). First, embracing dispatch that prioritizes zero-marginal cost resources such as wind and solar over fuel-burning generators requires the further evolution of markets and operations to consider economic dispatch. Second, to address the geographic diversity of renewable resources, improvements to long-distance transmission line operation, which is currently operated relatively rigidly based mostly on long-term contracts and fixed flow directions that are infrequently changed, must be modified. Third, transmission cost regulation, which is currently based on total flows in a way that incentivizes high, uni-directional utilization of the lines, must be revised to realize the balancing benefits for large-scale renewable energy provided to the system of many new corridors.

Fourth, the pace of annual wind and solar installations will need to increase several-fold from today’s ∼80 GW/yr in order to reach cumulative capacity targets by 2060. Assuming a scale-up of installations over time and incorporating wind and solar retirements (existing plants as well as a 25-y lifetime for new installations), annual capacity additions will need to average roughly 150 GW/yr in the period 2021 to 2045. In the later period (2046 to 2060), annual additions will need to reach roughly 300 GW/yr (see *SI Appendix*, section 6.1 for annual installation calculation and related analysis on supply chain feasibility and critical mineral availability to meet these install requirements). Importantly, with assumed technological improvement in these technologies, our model estimates that China’s future power system may be cost-effective in today’s terms.

This study has several limitations in uncovering the full range of possible carbon-neutral pathways for China. First, by capturing fine spatial heterogeneity in renewable resources, we limit ourselves computationally to exogenous scenarios of non-VRE generation sources, following economy-wide and other energy model outputs and government targets, and to simplifications in operational constraints. Exogenous firm power assumptions are based on an assessment of capacity planning in China, which includes both cost and noncost components, and appropriate sensitivities. Alternative assumptions may lead to different aggregate results. For example, other studies co-optimize resources given a specific renewable energy target (16) or emissions trajectory (20). Our model emphasizes the unique deployment constraints and potential for VRE. Neglecting unit-level modeling of operational constraints may overestimate the flexibility of the system. Beyond the sensitivities explored here, future work could examine the implications of large changes in the expected firm power mix and emissions reduction activities in nonelectricity sectors. Furthermore, we do not currently co-optimize the delivery of electricity and centralized heating. Future work could examine whether CHP-CCS may be substituted with electricity-based heating alternatives.

Second, the model has only a limited representation of demand flexibility, neglecting future sources of demand-side options that could reduce the need for generation and storage capacity, including flexible electric vehicle charging, commercial and industrial demand response, and cross-sector energy storage options such as hydrogen.

Third, real-world siting of generation and transmission resources would consider a larger range of factors than modeled here. In addition to connection and spur line costs to the transmission backbone, the contiguity and terrain of the land affect VRE project costs. The development of distributed solar is not only relative to differences in capital costs and connection methods, but also end-user preferences regarding electricity usage behaviors (60). New transmission siting must consider the cost and availability of rights of way. Highly granular geospatial models such as those developed here provide a framework for directly incorporating these considerations in future work.

In summary, the RESPO model developed here can reveal details of large-scale carbon-negative power systems from a coupled land and grid perspective. Since this study focuses on a milestone year (2060) of the transition of China’s power system, we only run the model as a cross-section analysis for 2020 and 2060 (focusing on the results in 2060). By co-optimizing spatially resolved renewable energy siting decisions and complementary infrastructure, the model allows for exploring practical constraints on the road to economy-wide net-zero emissions. The approach can be scaled to incorporate additional siting constraints where more abundant geospatial data are available. This modeling approach is particularly important at very high levels of VRE deployment in populous areas where geospatial constraints are more binding.

## Materials and Methods

### Input Data.

The potential hourly capacity factors for wind (onshore and offshore) and solar (utility-scale and distributed) power are assessed using meteorological data, including wind speed, solar irradiation, and surface temperature, with a geographical resolution of 0.3125° longitude by 0.25° latitude (approximately 30 km × 28 km at mid-latitudes) from version 5 of the Goddard Earth Observing System (GEOS-5) and GEOS-5 Forward Processing database. The potential deployable capacity given land availability is determined for onshore wind and solar according to constraints on altitude, slope, and land use type. For offshore wind, locations at a distance of less than 80 km from the shoreline and with a water depth of less than 60 m are considered. Power curves and technical parameters for the GE 2.5 MW turbine and Vestas 8.0 MW turbine are used to estimate the capacity potential and hourly capacity factor of onshore and offshore wind, respectively. We use a fixed-tilt solar evaluation model to calculate utility-scale solar capacity potential (9, 61). For distributed solar, we first estimate the suitable rooftop potential of each grid cell from MODIS before estimating capacity potential. See *SI Appendix*, section 2 for detailed assumptions, parameters, and visualizations.

China installed 282 GW wind and 253 GW solar by the end of 2020, respectively (37). We locate these installations by matching them with specific grid cells in this study. First, we acquire a project-level dataset of China’s utility-scale projects through 2020 from the National Renewable Energy Information Management Center, including the name, capacity, and location information of each project. This dataset covers 95% (267 GW) of total utility-scale wind installations (280 GW) and 98% (171 GW) of total utility-scale solar installations (175 GW), respectively. The location information is either the latitude and longitude of the project or the county where the project is located. For projects with county information only, we sum the installed capacity of this type by county and assign the total installations in each county to cells according to their capacity factors from high to low. For the remaining unmatched utility-scale installations and distributed installations, we first calculate the gap between the total wind/solar installations that have been already assigned in the previous step by province and total wind/solar installations by province. We then distribute installations to cells to fill this gap in each province, according to their capacity factors from high to low (see *SI Appendix*, Fig. S7 for the geographic distribution of installed wind and solar).

We adopt predictions for levelized-costs-of-electricity (LCOEs) from existing work (22, 62–65): 0.2 yuan/kWh for onshore wind, 0.3 yuan/kWh for offshore wind, 0.1 yuan/kWh for utility-scale solar, and 0.15 yuan/kWh for distributed solar, assuming that the annual national average capacity factor is 0.24 for onshore wind, 0.32 for offshore wind, 0.19 for utility-scale solar, and 0.16 for distributed solar, and the O&M cost is a fixed fraction of capital cost (1.5% for wind and 0.5% for solar) (66). We can then use a standard LCOE calculation model, e.g., ref. 11 to estimate the capital cost of each technology and calculate the LCOE of each technology for each cell (*SI Appendix*, Fig. S8).

As many VRE projects are located in remote areas with low population density far from more populous load centers, we incorporate the cost of connecting VRE generation in each grid cell to the nearest major demand node for local consumption or long-distance transmission. We follow the method of assigning trunk and spur lines in ref. 30. Since China does not disclose detailed locations of substations, based on our communication with the grid company, a reasonable assumption is that there is a substation at the seat of each county or district in a city. We determine the locations of major nodes based on future load centers and grid topology. Prefectural-level and above cities included in the city clusters for China’s future urbanization proposed in the 14th Five-Year Plan (36) and the terminals of existing ultra-high voltage (UHV) lines are treated as load centers. We further add a few cities (Chifeng and Hulunbeier in East Inner Mongolia, Kashgar in Southern Xinjiang, and Lhasa in Tibet) as load centers to guarantee that there is at least one major node designated for some large provinces/regions (see *SI Appendix*, Fig. S9 for substation and load center distribution and the connection patterns).

### Other Key Assumptions for China’s 2060 Power System.

Aggregate electricity demand is a function of macro-economic conditions and multi-sector interactions; therefore, we adopt demand forecasts in 2060 from an economy-wide integrated modeling analysis that used a general equilibrium model and a bottom–up power system planning model (34), with a baseline annual nationwide demand of 15.4 PWh (about 2.1 times of 2020 levels). Our base case VRE supply is 9.9 PWh, close to 9 PWh in the economy-wide model. We use regional demand profiles in ref. 11 to simulate provincial demand profiles by scaling each province’s profile to match its total electricity demand forecast in 2060. We do not consider any changes to diurnal or seasonal demand patterns from the present day.

Firm power capacity and spatial distribution assumptions are driven by an assessment of capacity planning methods in China, which include both cost and non-cost components. We assume coal CHP outfitted with CCS will supply winter district heating for urban areas in North China, and its provincial capacity is determined by the comparison of heat demand forecasts in 2060 (67, 68) and the observed heat demand in ref. 43. For natural gas, we scale provincial capacity numbers in 2018 provided in ref. 69 to match a national forecast (320 GW) by the GEIDCO (22) in 2060 driven by air pollution and flexibility objectives. We assume that in order to reach neutrality objectives, all natural gas plants will be equipped with CCS as well. We adopt the assumption of 580 GW hydro installations by 2060 from ref. 22, and we allocate to provinces by considering both detailed provincial hydro installation in refs. 70 and 71 and the share of expected installation in each grid region in 2060 (22). Coastal nuclear installation forecasts (218 GW) and detailed location information for China by 2060 given by ref. 40 is adopted, and the must-run rate (0.85) of nuclear is from ref. 30.

This study assumes that China’s power sector will support the overall carbon neutrality target by becoming a significant emission sink through the use of BECCS. We assume roughly 550 Mt/yr of negative emissions from the power sector in the base case as suggested in ref. 34 and adjust the national BECCS installation by 2060 to match this target, incorporating the slight positive emissions from partial capture rates of CHP-CCS and NG-CCS. With a capacity factor of 0.8 (about 7,000 annual generation hours) (55), China’s total BECCS capacity needs to reach about 110 GW. *SI Appendix*, section 3 shows detailed provincial capacity numbers for each firm generation technology.

In the base case, we consider two storage technologies: pumped hydro storage (PHS) and electrochemical battery storage (BAT). For sensitivity analyses, we design specific scenarios to evaluate the role of long-duration energy storage (LDES), as LDES is expected to play a significant role in better utilizing VREs and lowering the total system cost when VRE penetration is high (28, 72–75). We consider two representative LDES technologies in our sensitivity analyses, one with relatively low power capacity cost but also low round trip efficiency (RTE; e.g., compressed air energy storage, CAES) and the other with relatively high power capacity cost but also high RTE (e.g., vanadium redox-flow battery, VRB). We do not set any deployment constraints for BAT or LDES. For PHS, however, deployment depends on geological conditions. We collect data on installed capacity (32.5 GW), capacity under construction (53.9 GW), and additional long-term potential capacity (765.5 GW) by province from ref. 71 (see *SI Appendix*, Table S16 for details). We set the installed capacity plus capacity under construction as the minimum capacity in 2060, and the long-term potential capacity as the maximum capacity.

In our base case, we assume inter-provincial transmission lines can be built to strengthen existing inter-provincial links. We show the capacity of existing inter-provincial transmission lines in China by 2020 in *SI Appendix*, Fig. S15, based on the data collected from the State Grid and China Southern Power Grid. For provinces that do not have existing links, we assume no new lines would be built. To determine the voltage level of the transmission line between two specific provinces, we collect the voltage level information of existing transmission lines connecting these two provinces and assume that new lines built in the future will be the highest current voltage level or 500 kV, whichever is larger. The resulting voltage level matrix is in *SI Appendix*, Table S18. The distance between two provinces is calculated as their capitals’ geographic distance.

### Spatially Resolved Renewable Energy Siting and Power-System Optimization (RESPO) Model.

The RESPO model minimizes the sum of annualized investment costs and hourly operational costs over 8,760 h in a year, subject to constraints on hourly demand balance, hourly VRE availability, spatially disaggregated VRE deployment potential, firm generator (coal, hydro, nuclear, bio-energy, and natural gas) availability, storage energy balance, transmission constraints, and reserve requirements. The model outputs VRE, storage, and transmission deployment and their operations. The capacity of non-VRE generators in our model is pre-determined at the provincial level to meet various requirements, including negative emissions, winter heating demand, and must-run requirements, together with robust sensitivity analysis around these assumptions. RESPO is implemented with the Gurobi optimizer in Python, requiring roughly 200 GB of memory to solve.

We expand upon the layer dispatch model in ref. 11 by co-optimizing decision variables as well as considering inter-annual variability (in the “Two-year Period” scenario). Related to new capacity investment, the model includes cell-level capacities of onshore wind, offshore wind, utility-scale solar, and distributed solar, provincial capacities of storage technologies, and capacities of inter-provincial transmission lines. Decision variables related to operations include hourly power output and integration of each VRE grid cell, power output of firm generators, power transmitted through the transmission networks, charging/discharging of storage devices, and reserve capacity in each province (see *SI Appendix*, section 4 for the detailed model formulation). Our approach incorporates spatially disaggregated VRE decisions and availability profiles while simulating VRE integration using full chronological year data and inter-temporal considerations such as ramping. This is accomplished computationally primarily by simplifying operational complexity via modeling generation “layers” instead of individual units and by fixing non-VRE generation capacity decisions which are explored through scenario analysis.

## Supplementary Material

Appendix 01 (PDF)

## Data Availability

The land use data for China can be found at https://www.resdc.cn/DOI/DOI.aspx?DOIID=54 (56). The VRE resource assessment results (installation capacity potential under different land use scenarios) and raw data for all the figures can be found at https://doi.org/10.6084/m9.figshare.25140842.v1 (76). Given the file size of the VRE capacity factor data, it is available from the corresponding author upon reasonable request. All other data are included in the manuscript and/or *SI Appendix*. The code for the RESPO model can be found at https://github.com/mrziheng/RESPO (77).
