# Supplementary material for: Spatially resolved land and grid model of carbon neutrality in China
Source: Proc Natl Acad Sci U S A. 2024 Feb 26;121(10):e2306517121. doi: 10.1073/pnas.2306517121 (PMC10927511; doi:10.1073/pnas.2306517121)
Supplement: Supplementary file 1 — Appendix 01 (PDF) [file pnas.2306517121.sapp.pdf]

Supporting Information for
Spatially Resolved Land and Grid Model of Carbon Neutrality in China

Da Zhang, Ziheng Zhu, Shi Chen, Chongyu Zhang, Xi Lu, Xiliang Zhang, Xiaoye Zhang, Michael R. Davidson

Michael R. Davidson, Xi Lu
Email: mrdavidson@ucsd.edu; xilu@tsinghua.edu.cn

This PDF file includes:

Supporting text
Figures S1 to S161
Tables S1 to S26
SI References

Contents

1 Abbreviations **1**

2 Wind and solar supply **2**

2.1 Hourly capacity factor assessment of wind . . . 2

2.2 Hourly capacity factor assessment of solar . . . 3

2.3 Installation capacity potential assessment of wind and solar . . . 4

2.3.1 Land use policies for wind and solar development . . . 4

2.3.2 Estimation of suitable areas for wind and utility-scale solar . . . 10

2.3.3 Estimation of installation capacity potential of wind and utility-scale solar . . . 13

2.3.4 Estimation of installation capacity potential of distributed solar . . . 15

2.4 Locations of installed capacity . . . 16

2.5 Generation cost estimation . . . 17

2.6 Transmission connection costs . . . 19

2.6.1 Matching cells with substations and major nodes . . . 19

2.6.2 Calculating levelized connecting cost . . . 20

2.7 VRE cell clustered sensitivity . . . 21

3 Other input data and assumptions **24**

3.1 Provincial electricity demand profiles . . . 24

3.2 Firm generators . . . 24

3.3 Energy storage . . . 28

3.4 Inter-provincial transmission . . . 31

3.4.1 Strengthen existing transmission line . . . 31

3.4.2 Unconstrained UHV expansion . . . 34

4 Model formulation **36**

4.1 Definitions . . . 36

4.2 Objective function . . . 39

4.3 Constraints . . . 41

4.3.1 Demand . . . 41

4.3.2 VRE output . . . 41

4.3.3 Dispatchable supply . . . 41

4.3.4 Transmission operation . . . 42

4.3.5 Reserve capacity . . . 42

4.3.6 Ramp capacity . . . 43

4.3.7 Storage operation . . . 44

5 Scenario design **45**
5.1 Scenario definitions . . . 45
5.2 Load reduction . . . 47

6 Results and sensitivity analysis **48**
6.1 Annual installation capacity and supply chain capabilities . . . 48
6.2 VRE land use sensitivity . . . 49
6.3 VRE distribution . . . 51
6.4 Load profile . . . 61
6.5 Storage operation . . . 72
6.6 Transmission . . . 92
6.6.1 UHV utilization and congestion . . . 92
6.6.2 Sensitivity results . . . 92

1 Abbreviations

Table S1: Abbreviations.

Abbreviation	Definition	Abbreviation	Definition
AC	Alternating-current	MODIS	Moderate-Resolution Imaging Spectroradiometer
BAT	Battery storage	NASA	The US National Aeronautics and Space Administration
BECCS	Bio-energy carbon capture and sequestration	NREL	National Renewable Energy Laboratory
CAES	Compressed air energy storage	NPV	Net present value
CapEx	Capital expenditure (upfront)	O&M	Operation and maintenance
CCS	Carbon capture and sequestration	PHS	Pumped hydroelectric storage
CHP	Combined heat and power	PV	Photovoltaic
CF	Capacity factor	RESDC	Resource and Environmental Science and Data Center of the Chinese Academy of Science
CRF	Capital recovery factor	RTE	Round-trip efficiency
EIT	Enterprise income tax	SRTM	Shuttle Radar Topography Mission
FOM	Fixed operation & maintenance	SSE	Sum of squares of errors
GEOS–5	Version 5 of the Goddard Earth Observing System Data Assimilation System	VAT	Value added tax
IBT	Income before tax	VOM	Variable operation & maintenance
IRR	Internal rate of return	VRB	Vanadium redox-flow battery
LCOE	Levelized cost of electricity	VRE	Variable renewable energy
LDES	Long-duration energy storage	WACC	Weighted average cost of capital

Table S2: Province names, abbreviations, and corresponding grid region.

Province	Abbreviation	Grid region	Province	Abbreviation	Grid region
Anhui	AH	East	Jiangxi	JX	Central
Beijing	BJ	North	Liaoning	LN	Northeast
Chongqing	CQ	Central	East Inner Mongolia[a]	MD	Northeast
Fujian	FJ	East	West Inner Mongolia[a]	MX	North
Guangdong	GD	South	Ningxia	NX	Northwest
Gansu	GS	Northwest	Qinghai	QH	Northwest
Guangxi	GX	South	Sichuan	SC	Central
Guizhou	GZ	South	Shandong	SD	North
Henan	HA	Central	Shanghai	SH	East
Hubei	HB	Central	Shaanxi	SN	Northwest
Hebei	HE	North	Shanxi	SX	North
Hainan	HI	South	Tianjin	TJ	North
Heilongjiang	HL	Northeast	Xinjiang	XJ	Northwest
Hunan	HN	Central	Tibet	XZ	Northwest
Jilin	JL	Northeast	Yunnan	YN	South
Jiangsu	JS	East	Zhejiang	ZJ	East

[a] Inner Mongolia is split into two regions (East and West Inner Mongolia) as they belong to two different grid regions.

2 Wind and solar supply

2.1 Hourly capacity factor assessment of wind

We evaluate wind energy potential in China using version 5 of the Goddard Earth Observing System Data Assimilation System (GEOS-5) by the US National Aeronautics and Space Administration (NASA) [1]. Our base case uses data for the year of 2015. The GEOS-5 data provides a simulated hourly record of global wind speeds at specific heights (2 m, 10 m, and 50 m) with a spatial resolution of 0.3125° longitude by 0.25° latitude (approximately equivalent to 31.25 km × 25 km at mid-latitude per grid cell) [2]. We estimate hourly wind speeds at 100 m, which is the hub height for the GE 2.5 MW turbines, using a vertical power law profile [3]:

$$V(z) = V_{50}(\frac{z}{z_{50}})^{\alpha}, \qquad \text{(S2-1)}$$

where z and z_{50} refer to the turbine hub height and reference height (50 m), $V(z)$ and V_{50} indicate hourly values of the wind speed at the turbine hub height and 50 m respectively, and α defines the friction coefficient, a parameter varying as a function of the terrain where wind farms are located. Instead of taking a value of 1/7 as a rough approximation for α, we apply equation S2-1 on the basis of the wind speeds at 10 m and 50 m to estimate the value of α for each hour at each grid cell of the GEOS-5 domain. Both of these wind speed values are compiled through retrospective assimilation analysis, taking account of spatial and temporal variations in surface roughness and the stability of the atmosphere. Power curves and technical parameters for the GE 2.5 MW turbine and Vestas 8.0 MW turbine are used to estimate hourly capacity factor of onshore and offshore wind in China, respectively, with an overall power loss rate of 10% due to interference in airflow [4], see details in [5]. Detailed parameters of these wind turbines are listed in Table S3. We show the yearly average capacity factor of onshore and offshore wind for each cell in mainland China in Fig. S1.

Table S3: Parameters of wind turbines used in the wind potential estimation.

Parameters	GE 2.5 MW (onshore)	Vestas 8.0 MW (offshore)
Nameplate capacity (MW)	2.5	8.0
Cut-in wind speed (m/s)	3.0	4.0
Rated wind speed (m/s)	13.0	13.0
Cut-out wind speed (m/s)	25.0	25.0
Diameter (m)	100	164
Hub height (m)	100	100

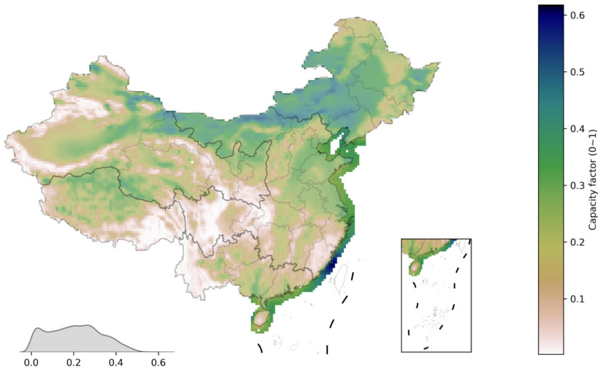

Fig. S1: Yearly average capacity factor of onshore and offshore wind in mainland China.

2.2 Hourly capacity factor assessment of solar

The utility-scale solar photovoltaic (PV) energy potential is evaluated based on GEOS-5 Forward Processing (GEOS-5 FP) of the Data Assimilation System (DAS) by the NASA. The GEOS-5 FP data provides a simulated hourly record of solar radiation-surface incident shortwave flux and temperature at 2 m above the displacement height with high spatial resolution (0.25° latitude by 0.3125° longitude), consistent with that of wind grid cells, for year 2015 used in our base case.

The solar module conversion efficiency is assumed to be 19.9%, and the system performance coefficient is assumed to be 83.2% [6, 7]. We adopt these assumptions and use the fixed-tilt solar evaluation model to calculate hourly capacity factor of utility-scale solar in each grid cell, see details in [6, 7]. As the study [8] introducing that, GEOS5-FP data is a reanalysis data that is based on modeling and assimilation, which could deviate from observed data in some regions. Since the GEOS5-FP data is the most comprehensive meteorological dataset that can provide consistent high-resolution (both temporally and spatially) solar resource information, we use another observation dataset to adjust the deviation. We first calculate annual capacity factor for each cell in our model using Global Solar Atlas data [9], which is based on observation, and then use this annual capacity factor to scale our original hourly capacity factors calculated by GEOS5-FP of each cell. Fig. S2 shows the yearly average capacity factor of utility-scale solar based on GEOS5-FP and adjusted by GSA for each cell in mainland China.

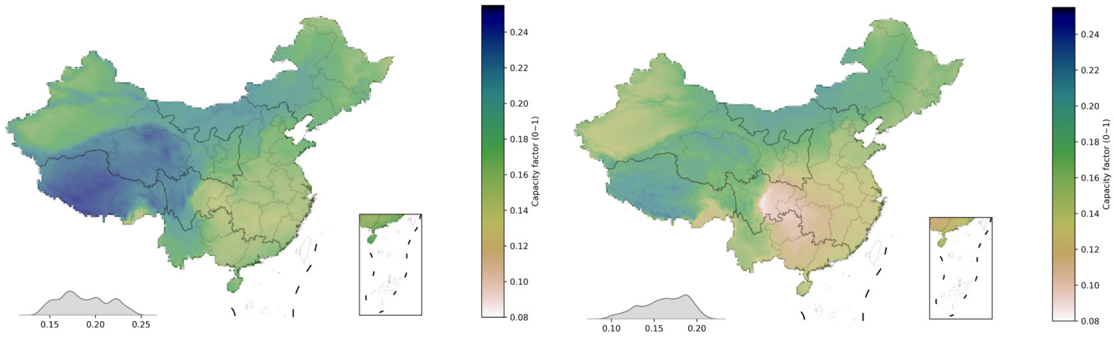

(a)

Fig. S2: Yearly average capacity factor of solar in mainland China, (a) origin from GEOS5-FP, and (b) adjusted by Global Solar Altlas data.

2.3 Installation capacity potential assessment of wind and solar

We estimate the installation capacity potential of wind and utility-scale solar based on the available land area and assumed installation density [4, 6] for each 0.3125° by 0.25° grid cell, consistent with the resolution of the GEOS-5 meteorological data. To evaluate the available land area for developing wind and utility-scale solar, we employ China Land Use/Cover Change Dataset (CNLUCC) for 2018 [10], provided by the Resource and Environmental Science and Data Center of the Chinese Academy of Sciences (RESDC), to represent land use and land cover at the "pixel" level within each grid cell. The CNLUCC land use dataset utilizes a two-level classification system. Level 1 categorizes land into six primary types: cropland, forest land, grassland, inland water areas, urban and built-up land, and unused land. Level 2 further delineates these into 24 specific land use types. Definitions for each land use type are provided in Table S5. We first exclude pixels in each grid cell that do not satisfy natural condition constraints, such as steep slopes, high altitudes, or deep water depths. The threshold values for the natural conditions are presented in Table S4. We then estimate the suitable area within the remaining pixels based on land use type and policy requirements. Finally, we aggregate the suitable area of qualified pixels in each grid cell to determine the total available area.

Table S4: Natural condition constraints that are unsuitable for developing wind and utility-scale solar PV.

Natural condition	Onshore wind			Offshore wind			Utility-scale solar		
	Conservative	Base	Open	Conservative	Base	Open	Conservative	Base	Open
Slope (%)	$>$15	$>$20	$>$25	-	-	-	$>$3	$>$5	$>$7
Altitude (m)	$>$3000	-	-	-	-	-	-	-	-
Distance from shore (km)	-	-	-	$< 10 \cup > 80$	$>$80	$>$80	-	-	-
Water depth (m)	-	-	-	$>$40	$>$60	$>$100	-	-	-

In this section, we summarize China's land use policies for wind and solar development and employ a range of parameter settings to account for various land use requirements. We then calculate the suitable area and corresponding installation capacity potential for each VRE type.

2.3.1 Land use policies for wind and solar development

To coordinate relevant land use regulations for supporting wind and solar power development, the Ministry of Natural Resources Land and Resources (formerly the Ministry of Land and Resources), the National Forestry and Grassland Administration (formerly the National Forestry Administration), and other ministries have formulated various policies. Some local governments have also issued relevant policies, which we summarize here.

For solar power projects, various policies [11–14] encourage to utilize unused lands, which refers to sandy land, Gobi land, saline-alkali land, marshland, bare land, rocky land and other unused lands including alpine desert and tundra. For example, the Opinions on Supporting the Development of New Industries and New Business Forms to Promote Mass Entrepreneurship and Innovation Land Use, jointly issued by the Ministry of Land and Resources and six other ministries in 2015, allows solar power generation projects to obtain the use rights of unused lands including desert and barren grassland by leasing.

The General Office of the Ministry of Land and Resources on Land Use for Photovoltaic Power Generation released in 2016 also supports utilizing unused lands and unused construction lands with utilization potential to develop solar power. In terms of agricultural land, relevant policies [12–14] stipulate that non-permanent basic farmland can be applied for the construction of agriculture and photovoltaic complementary projects which, however, need to meet the requirements to ensure the sustainable use of agricultural land. The National Forestry Administration has issued special regulations [15] on the use of forest land for solar projects, which mandate that forest lands such as nature reserves, forest parks, and habitats of endangered species are prohibited from construction, but some suitable forest areas can install solar panels on shade-resistant vegetation. Using woodland with a canopy density greater than or equal to 0.1 for solar projects is not encouraged. In addition, the guidance issued by the Ministry of Water Resources in 2022 [16] specifies that solar power station projects shall not be built on rivers, lakes, and reservoirs. China currently lacks specific policies related to solar projects on grassland, but Article 38 of the Grassland Law of the People's Republic of China [17] stipulates that grasslands are not encouraged for engineering construction use.

The land use requirements for wind power projects are similar to those for solar. Wind projects are encouraged to use unused lands as much as possible [11, 14, 18], and occupy less cropland, grassland, and forest land [14, 17, 18]. Permanent basic farmland is strictly prohibited, and construction in rivers, lakes, and reservoirs is prohibited [16]. In addition, the National Forestry Administration issued a notice on the use of forest land for wind farm projects [19], which clearly states that the construction of closed forest land and forest land in ecologically important and ecologically fragile and sensitive areas cannot be carried out.

1. Extracts from key general policies

- **Basic Farmland Protection Regulations [20]**[1]

  After the basic farmland protection area has been delimited according to law, no unit or individual may change or occupy it. Key national construction projects such as energy, transportation, water conservancy, and military facilities that have to occupy the basic farmland, involving the conversion of agricultural land or land expropriation, must be approved by the State Council.

  The following cropland shall be classified into basic farmland protection areas and strictly managed:

  1. *Cropland within the grain, cotton, and oil production bases approved by the relevant competent department under the State Council or the local people's governments at or above the county level;*
  2. *Cropland with good water conservancy and soil and water conservation facilities, and medium and low-yielding land that is under renovation plan, and can be transformed;*
  3. *Vegetable production bases;*
  4. *Experimental fields for agricultural scientific research and teaching.*

[1] Basic farmland is called permanent basic farmland after the Land Administration Law of the People's Republic of China came into effect on January 1, 2020.

- **Opinions on Supporting the Development of New Industries and New Business Forms to Promote Mass Entrepreneurship and Innovation Land Use [11]**

  For solar power, wind power, and other projects that use unused land such as Gobi land, desert, and barren grassland, the part of the project area that does not directly occupy land or change the land surface can still be identified as the original land use type.

  The land is allowed to be obtained by leasing and other ways. The two parties sign a compensation agreement, and the land use will be reported to the local Land and Resources Department at the county level for filing. For the part of permanent construction land of the project, the formalities shall be handled according to the construction land management rules.

- **The Guiding Opinions of the Ministry of Water Resources on Strengthening the Spatial Control of the Shoreline of River and Lake Waters [16]**

  Solar power stations, wind power generation, and other projects shall not be built on rivers, lakes, and reservoirs. The construction of solar and wind power projects around lakes and in the branches of reservoirs should be scientifically demonstrated and strictly controlled. They should not be placed in areas with flood control, water supply functions, and water ecological and water environmental protection needs, and should not hinder the smooth flow of flood, endanger the safety of water conservancy engineering facilities such as reservoirs, dams, and embankments, and affect the stability of river regime and navigation safety.

- **Grassland Law of the People's Republic of China [17]**

  Mining of mineral resources and engineering construction should not occupy grassland or occupy as little as it can. If it is really necessary to expropriate, requisite, or use grasslands, the examination and approval procedures for the use of construction land must be completed in accordance with the laws and administrative regulations on land management after the examination and approval of the grassland competent administrative department of the people's government at or above the provincial level.

- **Guidelines for Industrial Land Use Policy (2019 Version) [14]**

  All localities should actively guide the rational location of industrial projects according to the land space planning, and make the best use of unused land and unused construction land. Industrial projects should not occupy cropland or occupy as little as they can and strictly protect permanent basic farmland. The agricultural land or unused land used according to provisions can be certified and managed as the original land category, and the land must be used in strict accordance with the specified conditions.

2. Extracts from key policies for wind

- **Interim Measures for the Administration of Construction Land and Environmental Protection of Wind Farm Projects [18]**

The construction of wind farm projects should be based on the principle of saving and efficient use of land. It should try to use unused land, occupy less or no cropland, and avoid areas requiring special protection approved by government departments at or above the provincial level according to law.

- **Notice on Regulating the Use of Forest Land in the Construction of Wind Farm Projects [19]**

  Strictly protect the forest land in ecologically important and ecologically fragile, and sensitive areas. Natural heritage sites, national parks, nature reserves, forest parks, wetland parks, geological parks, scenic spots, main migration routes, and migration sites of birds, as well as coastal basic trunk forest belt and wave forest belt, are prohibited areas for wind farm projects. Wind turbine foundations, construction and maintenance roads, booster stations, and power lines are prohibited from occupying natural arbor forest (bamboo forest) land, woodland with canopy density greater than or equal to 0.2 and annual rainfall of less than 400 mm, first-class and second-class national public welfare forest land with canopy density greater than or equal to 0.2. In addition, according to the Administrative Measures for the Examination and Approval of the Use of Forest Land for Construction Projects, the use of forest land for wind power construction projects shall be subject to the examination and approval of the competent forestry authorities (the forest vegetation restoration fee shall be paid in advance according to the national standards, and the approval letter for the use of forest land shall be obtained), and then the construction units shall go through the examination and approval procedures for the use of construction land in accordance with laws and regulations.

3. Extracts from key policies for solar

- **Notice on Issues Related to the Use of Forest Land in the Construction of Solar Power Stations [15]**

  Various nature reserves, forest parks (including national parks of the same type), habitats of endangered species, natural forest protection project areas, and key state-owned forest areas in Northeast Inner Mongolia are prohibited from construction. Other areas with important ecological locations, fragile ecology, and broken terrain are restricted construction areas. The module array of the solar power station is prohibited from development in woodland with canopy density greater than or equal to 0.1, virgin forest land, cutting land, burning land, as well as shrub land with an annual rainfall of less than 400 mm and a coverage of more than 30% and shrubs with an annual rainfall of more than 400 mm and area coverage of more than 50%. For the land that is determined to be suitable forest land by the forest resource survey and the land that is determined to be unused by the second national land survey, the "forest solar-PV complementary" land use mode should be adopted, and the "forest solar-PV complementary" mode photovoltaic power station should ensure that the suitable forest land used does not change the nature of forest land.

- **Letter from the General Office of the Ministry of Land and Resources on Land Use for Solar Power [13]**

Support the development of solar power generation using unused land and unused construction land. For the solar power generation projects of "integration of agriculture and solar-PV" and "integration of fishing and solar-PV" that have been constructed using agricultural land before, the local competent departments of the Ministry of Land and Resources at all levels shall strengthen the tracking and monitoring together with relevant departments, and report the new situation and problems to the Ministry in time. For new solar power generation projects using agricultural land, all the land, including the photovoltaic array, shall be managed according to the construction land management rules, and the planning, conversion, collection, and supply procedures shall be performed according to law.

- **Opinions on Supporting Solar Poverty Alleviation and Regulating the Land Use of Solar Power Generation Industry [12]**

Solar power generation planning shall comply with the overall land use planning and other relevant plans. If unused land can be used, agricultural land shall not be occupied. If poor land can be used, good land shall not be occupied. It is forbidden to occupy permanent basic farmland in any way, and it is forbidden to develop photovoltaic power generation projects in areas explicitly prohibited by relevant national laws, regulations, and plans. For the construction of photovoltaic composite projects using agricultural land other than permanent basic farmland, the provincial energy and land resources authorities, in consultation with the relevant departments at the same level, need to study and propose the construction requirements (including the erection height of solar array) and identification standards of solar composite projects in the region, and clarify the regulatory measures to avoid the impact on agricultural production, on the premise of ensuring the sustainable use of agricultural land.

Table S5: Land use classification and definition used in this study.

Level 1		Level 2		Defination
ID	Name	ID	Name	
1	Cropland	-	-	Land used for cultivation of crops, including mature cropland, newly reclaimed land, fallow land, rotation land, grassland used for crop rotation; land used mainly for fruits, sericulture, agroforestry; land reclaimed from shoals and mudflats for over three years.
	-	11	Paddy/irrigated cropland	Land with assured water source and irrigation facilities that can be normally irrigated in a typical year for cultivation of aquatic crops like rice, lotus, etc., including land where rice and upland crops are rotated.
	-	12	Dry cropland	Land without irrigation water source and facilities, relying on natural precipitation for cultivation of crops; land with water source and irrigation facilities that can be normally irrigated for cultivation of upland crops in a typical year; land used mainly for vegetable cultivation; land with normal crop rotation like fallow and rotation land.
2	Forest land	-	-	Land used for forestry purposes with the dominance of trees, shrubs, bamboo, and mangroves along the coastline.
	-	21	Closed forest land	Natural and artificial forests with crown density $>$30%, including timber forests, economic forests, protected forests, and other large patches of forests.
	-	22	Shrubland	Dwarf forests and shrub forests with crown density $>$40% and height $<$2 m.
	-	23	Open forest land	Forest land with crown density of 10-30%.
	-	24	Other forest land	Afforested land but not yet forested, sparse land, nurseries and various orchards (fruit, mulberry, tea, agroforestry orchards, etc.).
3	Grassland	-	-	Various grasslands dominated by herbaceous plants, with vegetation cover $>$5%, including shrub grasslands used mainly for grazing and open forest grasslands with crown density $<$10%.
	-	31	High coverage grassland	Natural, improved and mown grasslands with vegetation cover $>$50%. Such grasslands generally have good moisture conditions and lush grass growth.
	-	32	Moderate coverage grassland	Natural and improved grasslands with vegetation cover 20-50%. Such grasslands generally have insufficient moisture and sparse grass growth.
	-	33	Low coverage grassland	Natural grasslands with vegetation cover 5-20%. Such grasslands lack moisture, have sparse grass growth, and poor grazing conditions.
4	Inland water area	-	-	Natural inland water bodies and water conservancy facilities.
	-	41	Rivers and streams	Naturally formed or artificially dredged rivers and streams, as well as land below the normal water level within river banks, including artificial canals and embankments.
	-	42	Lakes	Naturally formed water bodies and land below the normal water level within lake basins.
	-	43	Reservoirs and ponds	Artificially built water bodies and land below the normal water level within reservoirs and ponds.
	-	44	Permanent ice and snow	Land permanently covered by glaciers and snow.
	-	45	Tidal flats	The intertidal zone between high tide and low tide lines along the coast.
	-	46	River/lake shoals	Land between the normal water level and flood water level of rivers and lakes.
5	Urban and built-up land	-	-	Urban settlements and other built-up land outside settlements like industrial, mining, and transportation facilities.
	-	51	Urban land	Built-up areas of large, medium, and small cities as well as county towns.
	-	52	Rural settlements	Rural settlements independent of urban areas.
	-	53	Other construction land	Land for factories, mines, large industrial zones, oil fields, salt fields, quarries, etc. as well as transportation, airports, and other special purposes.
6	Unused land	-	-	Currently unused land, including land difficult to use.
	-	61	Sandy land	Land covered by sand, with vegetation cover $<$5%, including deserts but excluding river/lake shoals.
	-	62	Gobi land	Land covered mainly by gravels and rocks, with vegetation cover $<$5%.
	-	63	Saline-alkali land	Land with salt accumulation on the surface and sparse vegetation that can only grow highly salt-alkali resistant plants.
	-	64	Wetland	Flat and low-lying land with poor drainage, long-term dampness, seasonal or permanent waterlogging, and hydrophytes on the surface.
	-	65	Bare land	Land with soil cover and vegetation cover $<$5%.
	-	66	Bare rock	Land with $>$5% cover of rocks or rock fragments on the surface.
	-	67	Other unused land	Other unused land like alpine deserts, tundra, etc.

2.3.2 Estimation of suitable areas for wind and utility-scale solar

After excluding pixels that do not satisfy natural condition constraints, we implement additional exclusions for pixels situated within nature reserves and biodiversity conservation regions, thus adhering to policy stipulations concerning nature preservation [15, 19]. Subsequently, for the remaining pixels, the suitable area in each cell is determined by aggregating these pixels' area for offshore wind, and for onshore wind and utility-scale solar, based on land use policies reviewed in the last section and previous study [4–6, 21–24], we establish suitability scores for each land use type by three cases (base, conservative, and open). The suitability score is defined as the ratio of the suitable area for developing onshore wind or utility-scale solar to the total area of a pixel. Finally, we determine the suitable land area for each grid cell by aggregating suitable areas of all the pixels within it.

The Chinese government strictly protects permanent basic farmland to ensure food security. Article 35 of China's Land Administration Law stipulates that once permanent basic farmland has been delineated, no unit or individual may occupy or change its use without authorization [25]. Guidelines for Implementing Industrial Land Policies issued by the Ministry of Natural Resources in 2019 also stipulate that construction land shall not occupy cropland and permanent basic farmland shall be strictly protected [14]. Notice on Supporting the Development of Photovoltaic Power Generation Industry issued by the Ministry of Natural Resources in 2023 further states that the permanent basic farmland shall not be occupied in any way [26]. Unfortunately, there is no public information about the detailed boundary of permanent basic farmland, and we have to make assumptions about cropland in the CNLUCC data. Since paddy/irrigated cropland includes cropland equipped with reliable water sources and irrigation facilities, consistent with the second category of permanent basic farmland, we set the suitability scores for wind and solar on irrigated cropland at 0%. This measure ensures adherence to the permanent basic farmland protection requirements. For the dry cropland, however, current policies do not strictly prohibit wind or solar development if it is not permanent basic farmland. We make a conservative assumption for the suitability score for solar at 5% in the base case, as solar PV panels need to cover significant land areas. In contrast, wind turbines only occupy very small land areas (about 0.1% of the area of a wind farm, estimated using the required deployment density and turbine tower base size), and their impact on farming is limited, so we set the suitability score for wind at 80% in the base case.

We delineated corresponding suitability scores based on canopy closure of forested areas and percent cover of grasslands, respectively. In 2015, the State Forestry Administration issued a notice stipulating that all types of nature reserves, forest parks (including national parks of the same type), habitats of endangered species, natural forest protection project areas, as well as state-owned forests in Northeast China and Inner Mongolia, are prohibited for wind or solar development [15]. We then exclude "closed forest land" (level 2 ID 21 in CNLUCC data) land type because the State Forestry and Grassland Administration further stipulated strict protection of ecologically important and fragile forest areas in 2019 [19]. We furthermore exclude "high coverage grassland" and "moderate coverage grassland" (level 2 ID 31 and 32 in CNLUCC data) land type because Article 38 of the Grassland Law of the People's Republic of China stipulates that mining and engineering construction should avoid or minimize occupation of grass-

lands [27]. For the remaining forest land and grassland types, we set the suitability score at 80% for onshore wind and 5% and 20% for utility-scale solar, respectively.

We set the suitability score for inland water bodies at 0% for the reason that the Guiding Opinions on Strengthening the Spatial Regulation and Control of River and Lake Shorelines issued by the Ministry of Water Resources in May 2022 stipulate that solar PV power stations, wind power projects, and other constructions shall not be built within river channels, lakes or reservoirs [28].

In urban and built-up areas, for onshore wind, we set the suitability scores in these land types to 0%, due to its noise and greater visual impact; and for solar PV, as it can be potentially deployed on some industrial, mining, and transportation land, we make a conservative assumption for the suitability score for solar PV at 5% in the base case.

Wind and solar are encouraged by the Chinese government to be deployed on unused land (level 1 ID 6 in CNLUCC dataset). For example, in 2019, the Ministry of Natural Resources stipulated that unused land should be utilized to the maximum extent possible for project construction and development [14]. In 2023, the Ministry of Natural Resources announced its support for using unused land and existing construction land to develop photovoltaic power generation [26]. Considering that utility-scale solar PV panels are more land intensive while wind turbines have smaller footprints, we set lower suitability scores for utility-scale solar PV (40%) than onshore wind (80%) in the unused land.

The detailed suitability score values for each land use type in three cases are listed in Table S6. We show the suitable area for wind and utility-scale solar for each cell in Fig. S3.

Table S6: Suitability score of each land use type for determining suitable areas for onshore wind and utility-scale solar.

Level 1		Level 2		Onshore wind (%)			Utility-scale solar (%)		
				Conservative	Base	Open	Conservative	Base	Open
ID	Name	ID	Name	-	-	-	-	-	-
1	Cropland	-	-	-	-	-	-	-	-
-	-	11	Paddy/irrigated cropland	0	0	0	0	0	0
-	-	12	Dry cropland	60	80	100	0	5	10
2	Forest land	-	-	-	-	-	-	-	-
-	-	21	Closed forest land	0	0	0	0	0	0
-	-	22	Shrubland	60	80	100	0	5	10
-	-	23	Open forest land	60	80	100	0	5	10
-	-	24	Other forest land	60	80	100	0	5	10
3	Grassland	-	-	-	-	-	-	-	-
-	-	31	High coverage grassland	0	0	0	0	0	0
-	-	32	Moderate coverage grassland	0	0	0	0	0	0
-	-	33	Low coverage grassland	60	80	100	15	20	25
4	Inland water area	-	-	-	-	-	-	-	-
-	-	41	Rivers and streams	0	0	0	0	0	0
-	-	42	Lakes	0	0	0	0	0	0
-	-	43	Reservoirs and ponds	0	0	0	0	0	0
-	-	44	Permanent ice and snow	0	0	0	0	0	0
-	-	45	Tidal flats	0	0	0	0	0	0
-	-	46	River/lake shoals	0	0	0	0	0	0
5	Urban and built-up land	-	-	-	-	-	-	-	-
-	-	51	Urban land	0	0	0	0	5	10
-	-	52	Rural settlements	0	0	0	0	5	10
-	-	53	Other construction land	0	0	0	0	5	10
6	Unused land	-	-	-	-	-	-	-	-
-	-	61	Sandy land	80	90	100	30	40	50
-	-	62	Gobi land	80	90	100	30	40	50
-	-	63	Saline-alkali land	80	90	100	30	40	50
-	-	64	Wetland	80	90	100	30	40	50
-	-	65	Bare land	80	90	100	30	40	50
-	-	66	Bare rock	80	90	100	30	40	50
-	-	67	Other unused land	80	90	100	30	40	50

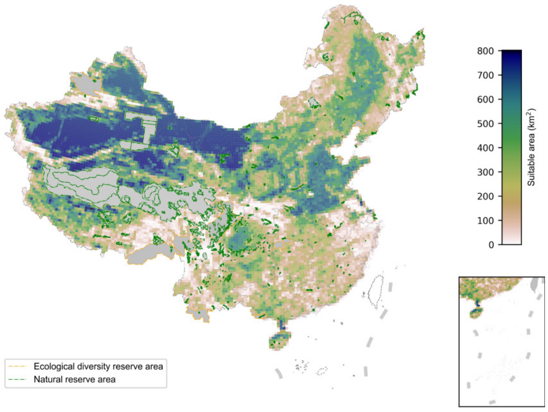

(a) wind open

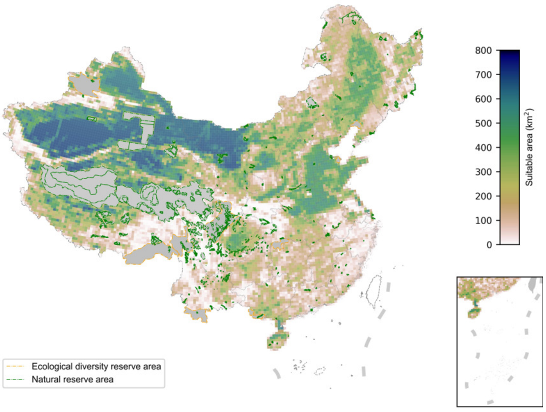

(b) wind base

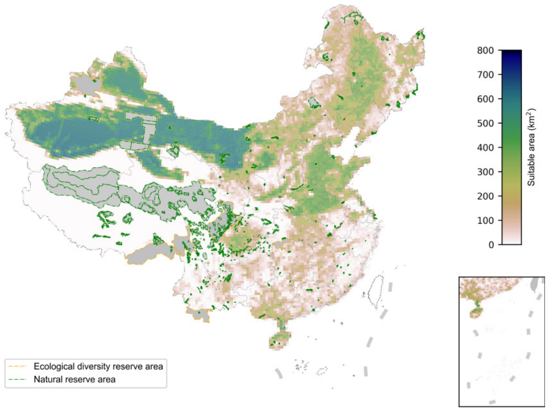

(c) wind conservative

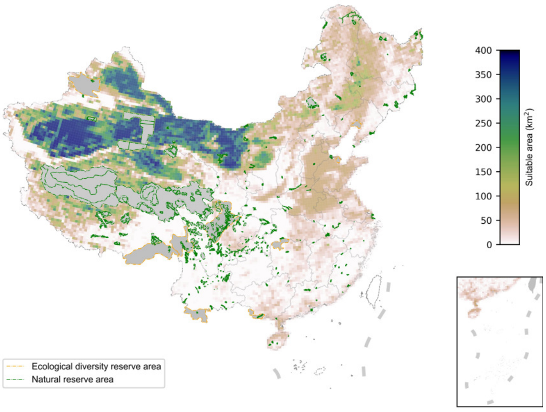

(d) solar open

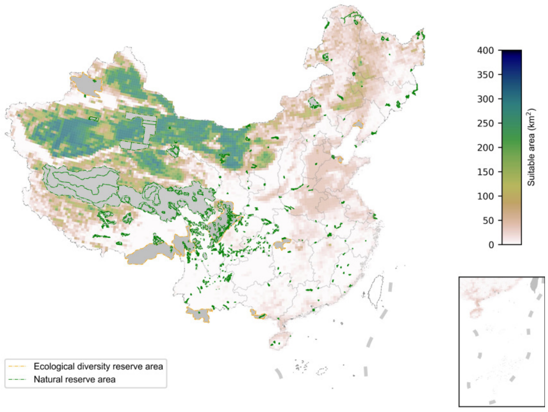

(e) solar base

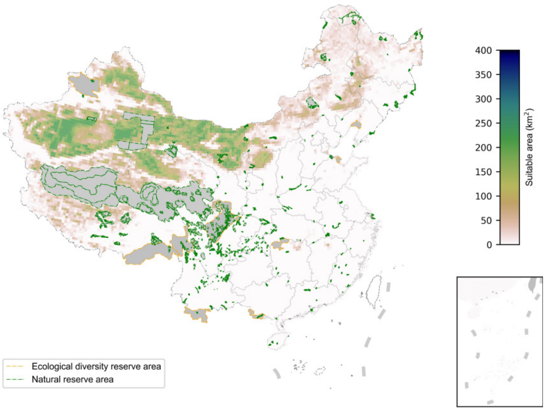

(f) solar conservative

Fig. S3: Suitable area (km^2) for wind (a, b, and c) and solar (d, e, and f) in open, base, and conservative cases.

2.3.3 Estimation of installation capacity potential of wind and utility-scale solar

We assume that each wind turbine requires an area of ten rotor diameters in the downstream direction and five diameters in the cross-wind direction, which means one km^2 of suitable land can be installed approximately 5 MW (GE 2.5 MW turbine) and 6 MW (Vestas 8.0 MW turbine) for onshore and offshore

wind, respectively [4]. The wind capacity potential of each cell can be calculated by multiplying the suitable area (km^2) and installation density (MW/km^2).

We use the fixed-tilt solar evaluation model to calculate the packing factor (PF) for each cell. The packing factor denotes the effective panel area per square meter of land area (ranges from zero to one) and is a function of latitude: the higher the latitude, the smaller the PF, see details in [6]. The installation capacity potential for utility-scale solar in each cell is calculated by PF, the power per unit area of the PV panel, and the area of suitable land:

$$\text{Capacity potential} = \text{PF} \times P_{wp} \times A_s, \tag{S2-2}$$

where P_{wp} represents the power per unit area of the panel (P_{wp} = 161.9 MW/km^2 in this study), and A_s is the area of suitable land for installing PV panels. We show the installation capacity potential of wind and solar under different land use cases for each cell in mainland China in Fig. S4

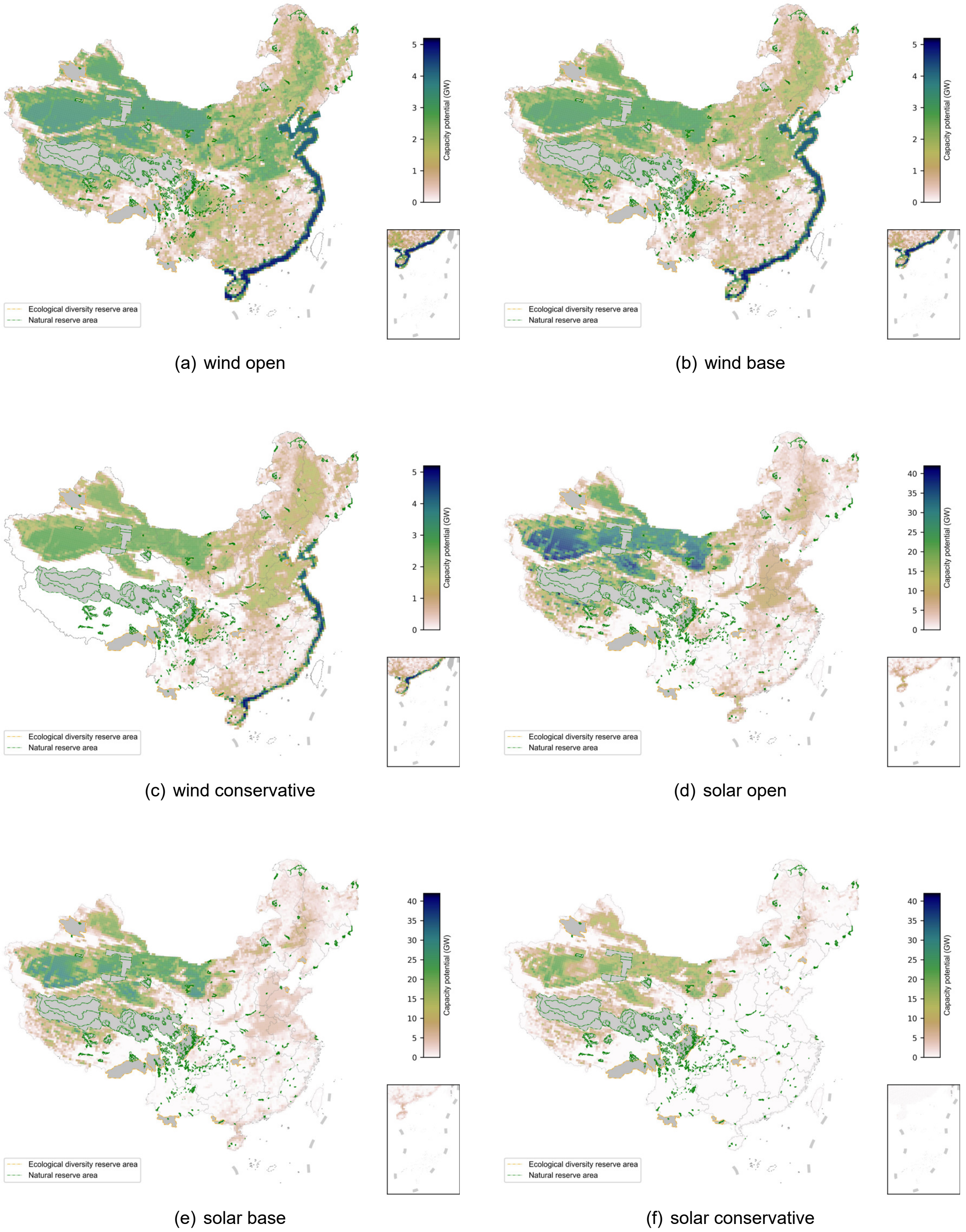

(a) wind open

(b) wind base

(c) wind conservative

(d) solar open

(e) solar base

(f) solar conservative

Fig. S4: Installation capacity potential (GW) for wind (onshore and offshore, a, b, and c) and utility-scale solar (d, e, and f) in open, base, and conservative cases.

2.3.4 Estimation of installation capacity potential of distributed solar

For distributed solar photovoltaic (DPV) potential, we first estimate the available rooftop potential of each grid cell. First, we use the MODIS land cover type yearly 500 m (MCD12Q1.006) [29] to calculate the built-up area density for each grid cell. We then use a 15% building density for each built-up area and

50% available rooftop area per building area based on [30]. The solar radiation condition for each cell is estimated using the same methods as the utility-scale solar evaluation. The installation capacity potential for distributed solar in each cell is estimated by using the solar radiation condition and the available building area. We show this capacity installation potential for each grid cell in Fig. S5 (unsuitable areas are shown in white). The hourly capacity factor is the same as the value for the utility-scale solar, and the yearly average capacity factor is shown in Fig. S2 (b).

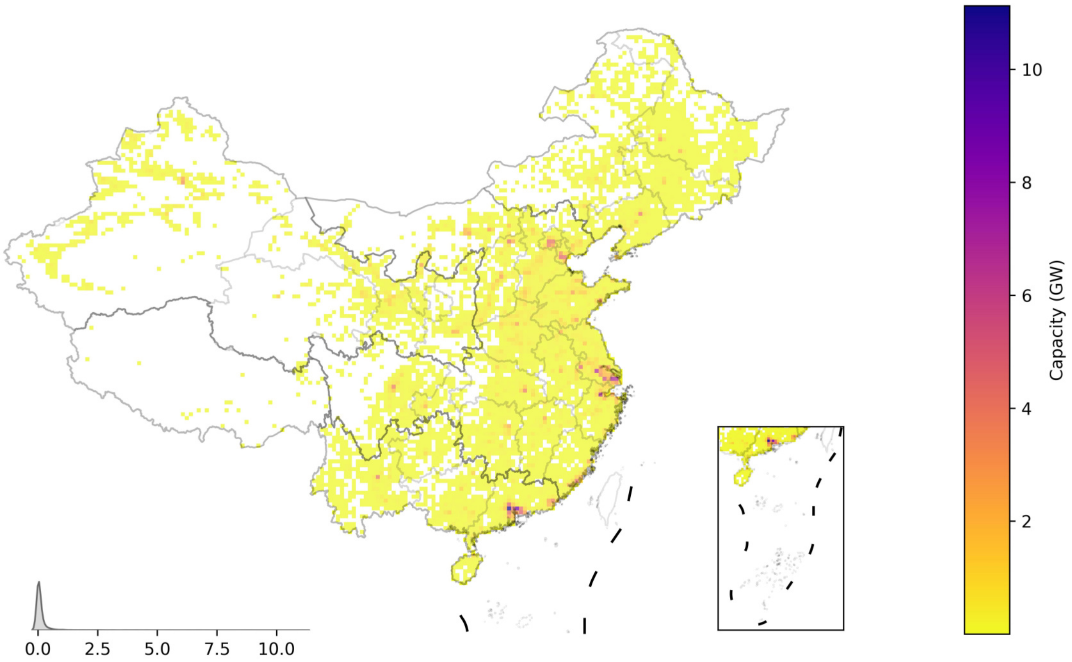

Fig. S5: Distributed solar capacity installation potential in mainland China.

2.4 Locations of installed capacity

By the end of 2020, China has installed 282 GW wind and 253 GW solar [31], respectively. Fig. S6 shows the total wind and solar installed capacity by province. In this study, we need to locate these installations by matching them with specific grid cells. This process is implemented as follows.

First, we acquire a unique project-level data set that collects most of China's utility-scale projects by 2020 from the National Renewable Energy Information Management Center, including the name, capacity, and location information of each project. This data set covers 95% (267 GW) of total utility-scale wind installations (280 GW) and 98% (171 GW) of total utility-scale solar installations (175 GW), respectively. The location information is either the latitude and longitude of the project that can directly match to a cell, and/or the county where the project locates. For projects with county information only, we sum the installed capacity of this type by county and assign the total installations in each county to cells according to their capacity factors from high to low.

To match the remaining utility-scale installations and distributed installations to specific cells, we first calculate the gap between the total wind/solar installations that have been already assigned to cells in the previous step by province and total wind/solar installations by province. To fill this gap, we then

distribute installations to cells in each province, according to their capacity factors from high to low. The geographic distribution of installed wind and solar capacity is shown in Fig. S7. Note that there is no existing wind capacity in Tibet, due to high altitude and ecological constraints. In our modeling analysis, we also assume that no new wind capacity could be installed in Tibet.

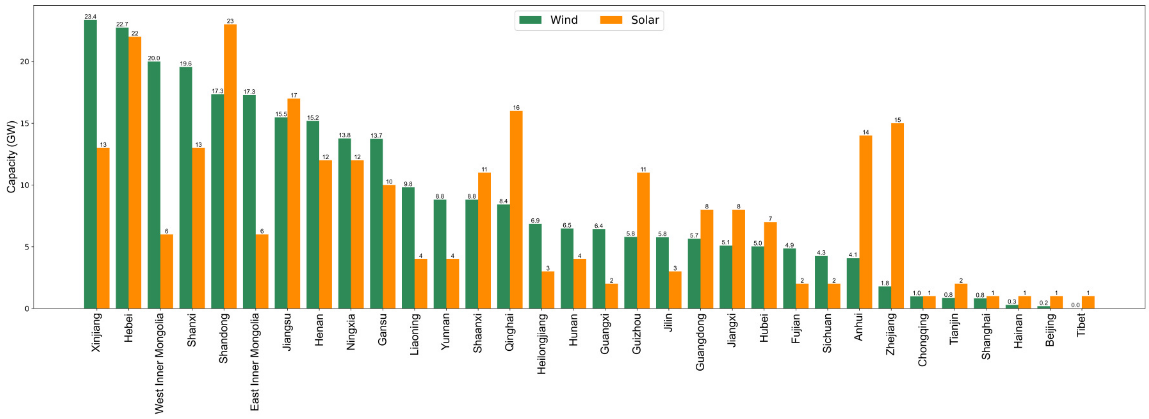

Fig. S6: Provincial installed wind/solar capacity by the end of 2020.

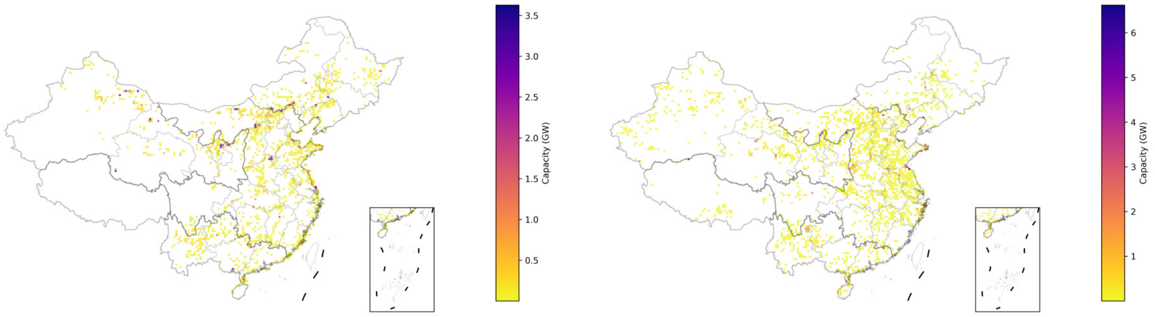

(a) (b)

Fig. S7: Geographic distribution of installed wind (a) and solar (b) capacity in mainland China.

2.5 Generation cost estimation

We estimate the generation cost of wind and solar energy by each grid cell, based on its capacity factor and corresponding capital and operation and maintenance (O&M) costs. Following the standard levelised-cost-of-electricity (LCOE) calculation as described in [32], we calculate the LCOE of wind and solar for each grid cell. We adopt the same financial parameters, including value-added tax (VAT) rate, enterprise income tax (EIT) rate, share of equity in the initial investment, loan interest rate, required pay-back period, and required internal rate of return (IRR) for equity, from [32].

Capital expenditure (CapEx, including equipment costs and other initial investment costs) and operation and maintenance costs are two important parameters in the LCOE calculation. As technology advances, we expect that the LCOE of wind and solar power will continue to decline dramatically, and we adjust the CapEx of each variable renewable energy (VRE) technology to match the target LCOE

assumptions described as below. [33] predicts that the LCOE of onshore wind and fixed-bottom offshore wind in Asia by 2050 range from about 28–36 \$/MWh and about 32–66 \$/MWh, respectively. A report [34] gives that the LCOE of onshore and offshore wind in 2060 are 0.15 yuan/kWh and 0.3 yuan/kWh. We adopt predictions that the LCOE of onshore and offshore wind power could reach 200 yuan/MWh and 300 yuan/MWh (about 31 \$/MWh and 47 \$/MWh, the median value of the prediction by [33] and close to that from [34]) assuming yearly average capacity factors of 0.24 and 0.32, respectively. The LCOE of utility-scale solar in 2060 is predicted at about 12–21 \$/MWh by the National Renewable Energy Laboratory (NREL) [35] and at 0.07 yuan/kWh by [34], respectively. Therefore, We assume the prediction that the LCOE of utility-scale solar in 2060 could reach 100 yuan/MWh (about 16 \$/MWh, the median value of the prediction by the NREL and close to that of [34]) if the yearly average capacity factor is 0.17. Based on [36, 37], which indicate the LCOE of DPV in China is about 50% higher than utility-scale solar, we assume the LCOE of DPV power could reach 150 yuan/MWh if the yearly average capacity factor is 0.16.

The O&M cost is assumed as 1.5% for wind (onshore and offshore) and 0.5% for solar (utility-scale and distributed) of the initial CapEx [38]. Based on these cost assumptions, we have corresponding CapEx and O&M cost assumptions shown in Table S7. We then calculate the LCOE of each grid cell of mainland China shown in Fig. S8.

Table S7: Generation cost assumptions of VRE.

	CapEx (yuan/kW)	O&M cost (yuan/kW·yr)	National average CF
Onshore wind	3,000	45	0.24
Offshore wind	5,400	81	0.32
Utility-scale solar	1,500	7.5	0.17
Distributed solar	2,000	10	0.16

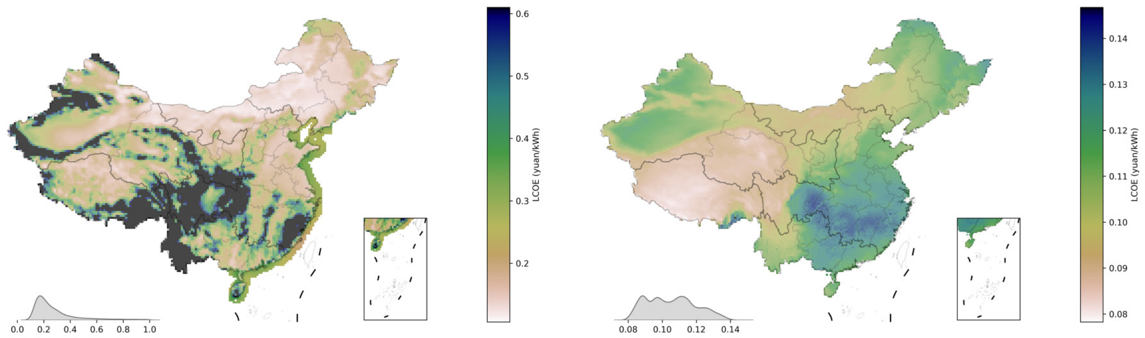

(a) (b)

Fig. S8: LCOE of wind (a) and utility-scale solar (b) generation. In (a), the selected range of offshore wind power is 80 km from the shoreline. The wind power resources in the black area are poor, so the LCOE is greater than or equal to 0.6 yuan/kWh. In the lower-left corner of (a) and (b) is the LCOE distribution.

2.6 Transmission connection costs

Many wind and solar projects are located in remote areas far from load centers. Therefore, we need to estimate the cost of connecting wind or solar generation in each cell to the nearest major node (described below) for use or further transmission. We estimate these connecting costs based on the method proposed in [39]. Wind or solar power generated in a specific cell needs to be first connected to a close-by substation through “spur lines”, then “trunk lines” are needed to be built or strengthened for the transmission from the substation to the nearest major node in the province.

2.6.1 Matching cells with substations and major nodes

Since China does not disclose detailed locations of substations, based on our communications with the grid company, we can reasonably assume that there is a substation at the seat of a county/district in a city, represented by the center of grey radial lines (spur lines) in Fig. S9.

We determine the locations of major nodes based on future load centers and grid topography. The 14th Five-Year Plan [40] presents a detailed layout of city clusters for China’s future urbanization. We treat cities with prefecture level or above included in this layout as load centers. We further add a few cities (Chifeng and Hulunbeier in East Inner Mongolia, Kashgar in Southern Xinjiang, and Lhasa in Tibet) as load centers to guarantee that there is at least one major node designated for some large provinces/regions. Endpoints of existing ultra-high voltage (UHV) lines are also treated as major nodes. These major nodes are represented by the center of red radial lines (trunk lines) in Fig. S9.

We match each cell first to a substation and then to a major node in the same province with an algorithm that guarantees the total geodesic distance of connecting lines for this cell is minimal.

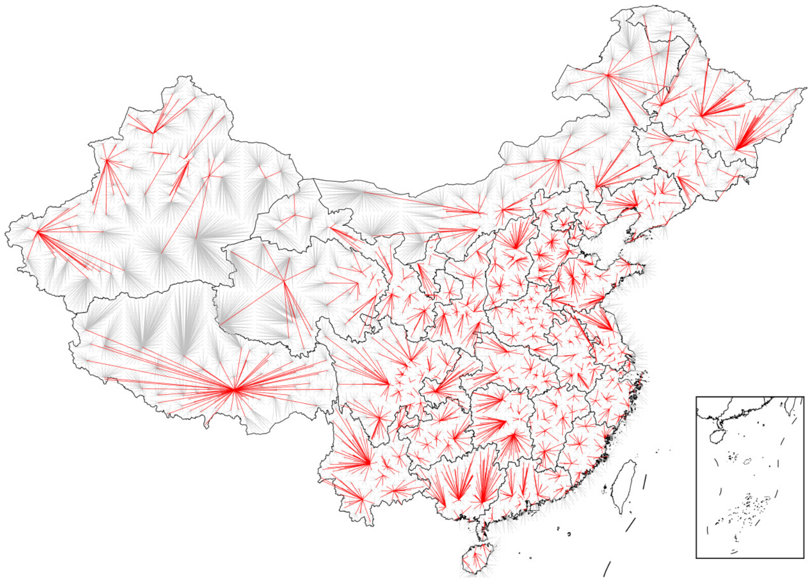

Fig. S9: Illustration of grid cell interconnection to the main transmission network. Connections consist of spur lines to the nearest substation (silver) and trunk lines to the nearest load center or UHV terminal (red) in a given province.

2.6.2 Calculating levelized connecting cost

We calculate the cost of spur lines and trunk lines based on the required capacity for transmission and incorporate these costs into the objective function of our optimization model. For a spur line that connects a cell to a substation, its capacity is equal to the total capacity chosen in this cell (installed capacity discussed in Section S2.3 plus capacity for future development selected by the model) multiplied by the maximum hourly capacity factor of this cell. For a trunk line that connects a substation to a major node, its capacity depends on the peak output of aggregated profiles of the total capacity in all the cells connected to this substation. Since the peak output of each cell connected to the substation may occur at different time, we cannot simply sum the peak output over all the cells to obtain the required capacity of the trunk line. Detailed representation of spur line and trunk line in our optimization model is described in Section S4.2.

We assume 220 kV alternating-current (AC) transmission is used for both the spur and trunk lines. CapEx is given as 8.3 yuan/kW· km and transmission losses are given as 0.0032 [%/km] [41]. We use a capital recovery factor (CRF) ξ to convert upfront CapEx to annuities, which is given by:

$$\xi = \frac{\omega(1+\omega)^{\tau}}{(1+\omega)^{\tau}-1}, \tag{S2-3}$$

where ω is the real weighted average cost of capital (WACC) expressed as a fraction, and τ is the required financial lifetime (or capital recovery period) of the investment, ω and τ values are technology-specific and 4.2% real WACC and 50-year lifetime are chosen in our calculation.

The levelized cost of spur line ($\mathcal{L}$, unit: yuan/kWh) for a given cell can be calculated as follows,

$$\mathcal{L} = \frac{\xi \times \max\limits_{h \in [1,\dots,8760]} cf_h \times K^{cap} \times D}{CF \times (8760\text{ h/yr}) \times (1-loss)^{D}}, \tag{S2-4}$$

where K^{cap} is the CapEx per kW, CF is the yearly average capacity factor of this cell, $loss$ is the transmission loss, and D is the distance of the spur line connecting the cell and the substation. Fig. S10 visualizes the levelized cost of spur line for wind and solar transmission.

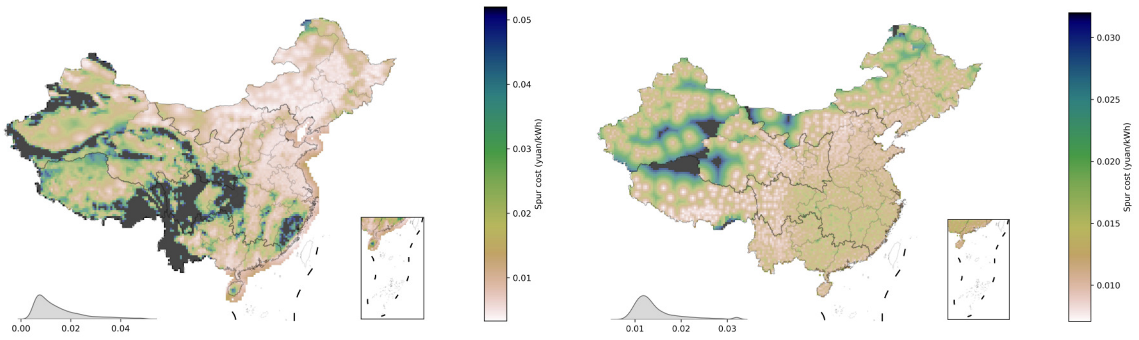

(a) (b)

Fig. S10: Levelized cost of spur line for wind (a) and solar (b) in mainland China. In (a), cells with short connection distances have high costs because their yearly average capacity factors are low. In (b), the connection distance of many cells in the west is larger than that in the east, but they have a smaller spur line cost, which is due to their better solar resources.

For the levelized trunk line cost for each cell, we cannot provide a straightforward visualization because the levelized cost of one cell depends on the output profiles of total capacity in other cells that are connected to the same substation, which are endogenously determined by the model.

2.7 VRE cell clustered sensitivity

In this study, we carefully formulate our optimization model to handle gridded VRE resource profiles while retaining computational feasibility (see model formulation details in Section S4). Traditional continent-scale renewable expansion studies (e.g., [39,42]) typically aggregate high-resolution renewable resource data into a few resource classes per region. To compare the difference in results from incorporating high spatio-temporal granularity, we implement a VRE aggregation sensitivity.

In the cell clustered sensitivity, we cluster each type of VRE technology (onshore wind, offshore wind, utility-scale solar, and distributed solar) separately. For each VRE technology, since generation costs and connection costs are two major factors that determine the cost of deploying VREs in each cell, we use a two-step clustering strategy based on capacity factor and connecting distance to group cells in each province. First, we use the k-means algorithm to cluster cells with similar hourly capacity factor profiles together. Euclidean distance is used to measure hourly capacity factor distances between cells. The Elbow method based on the sum of squares of errors (SSE) and the Silhouette Coefficient method is used to determine the number of clusters in each province. Within each cluster in each province, we then use the same clustering algorithm to further group cells into smaller clusters based on the total connecting distance (spur line length plus trunk line length) of each cell. Clustering results for wind and utility-scale solar are shown in Fig. S11 and S12.

For each cluster, we sum the capacity of all the cells within it to calculate its capacity. The weighted average capacity factors and connecting distances are used to calculate the generation cost and connecting cost of each cluster.

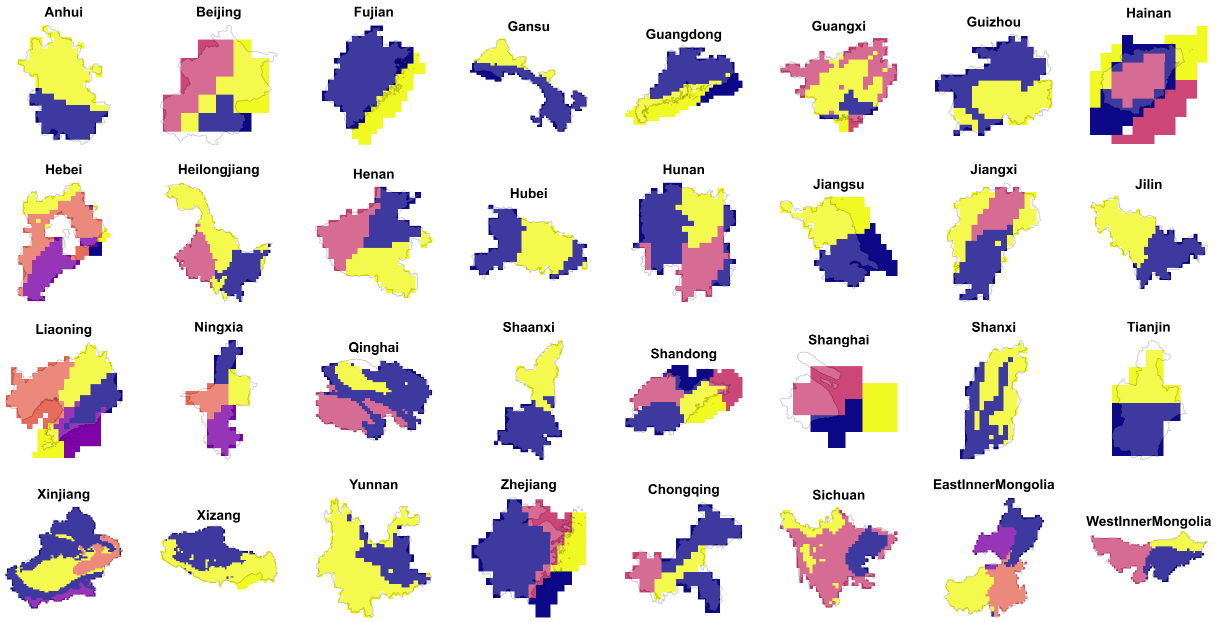

(a)

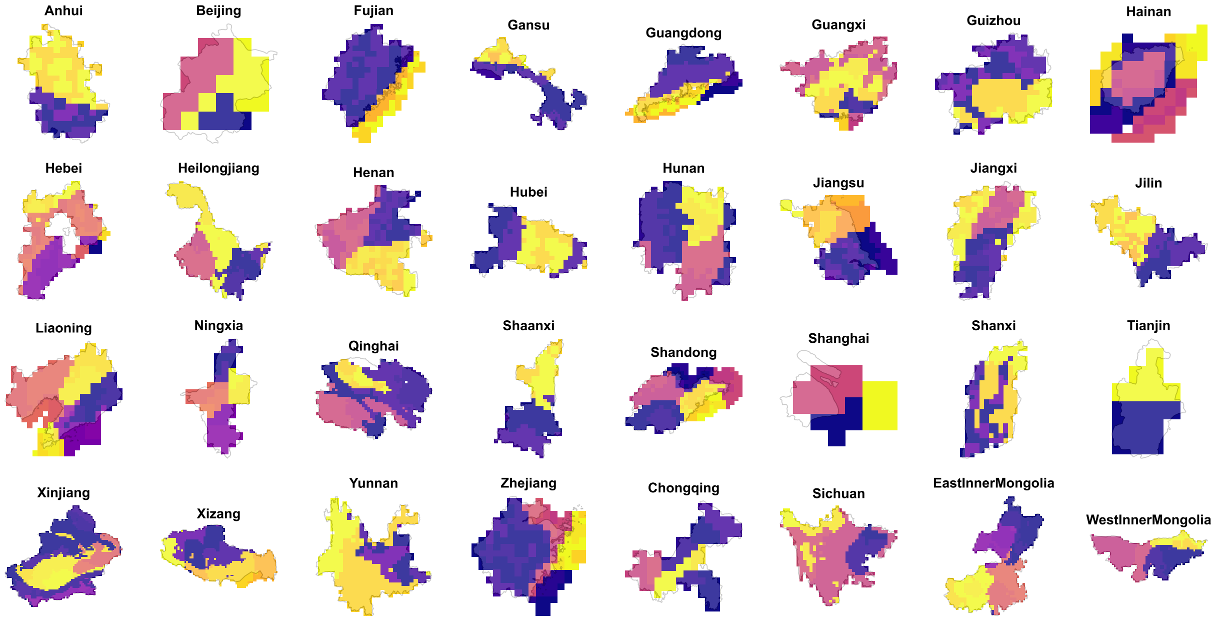

(b)

Fig. S11: Provincial aggregation results of wind in mainland China by capacity factor (a) and connection distance (b). In each sub-figure, cells with the same color belong to the same cluster.

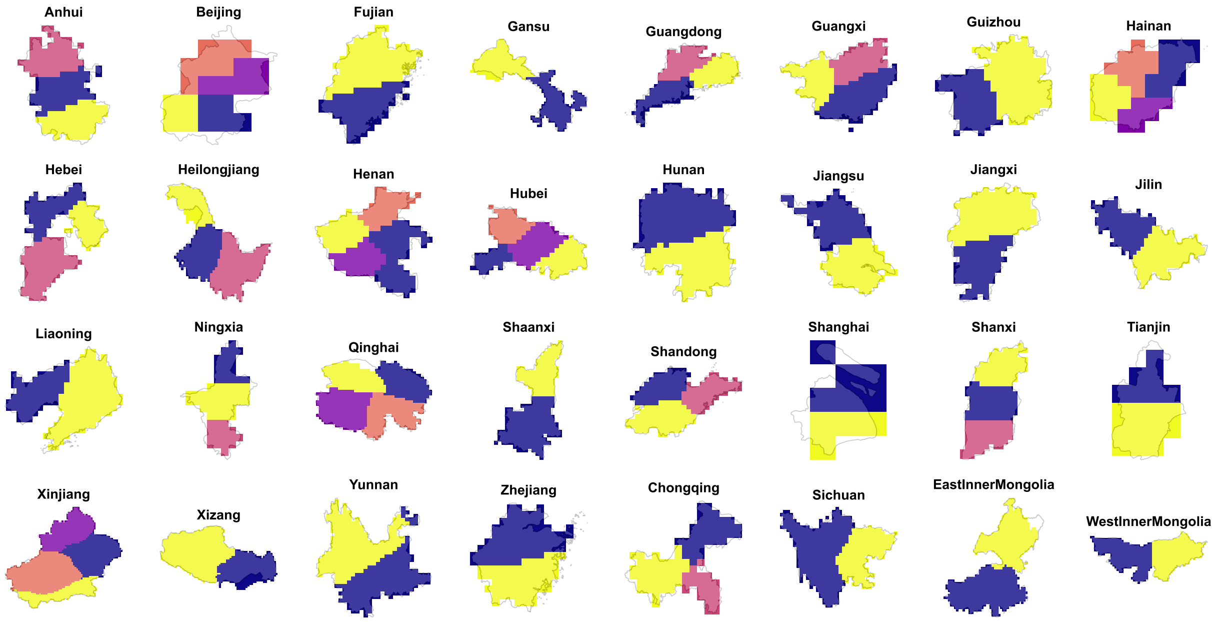

(a)

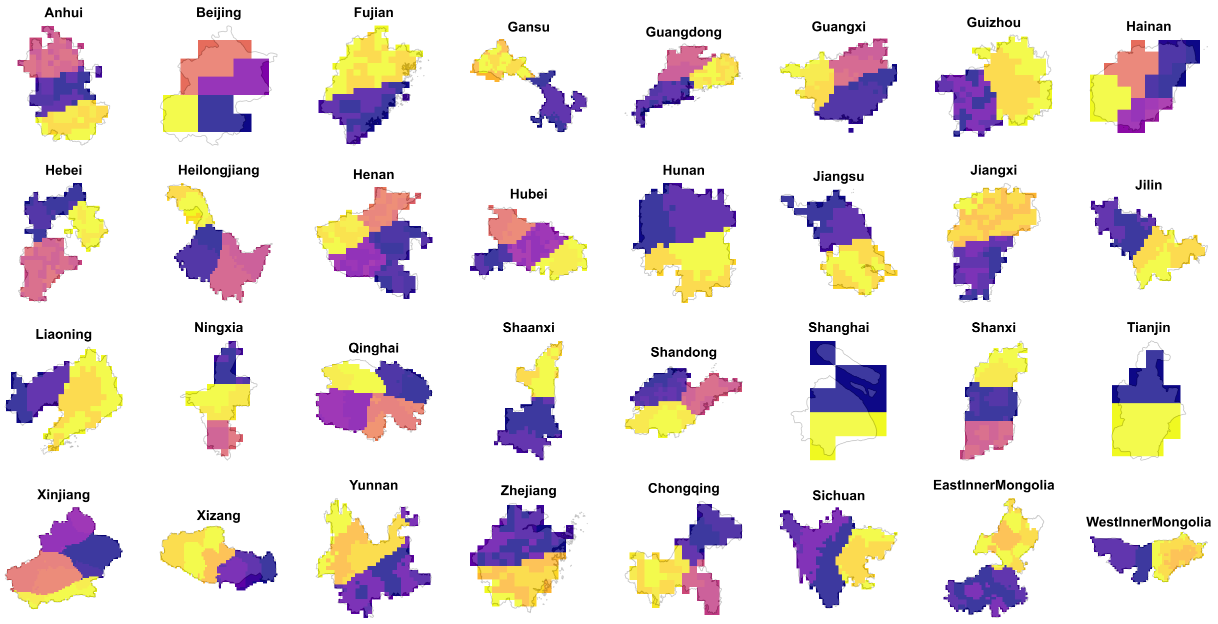

(b)

Fig. S12: Provincial aggregation results of solar in mainland China by capacity factor (a) and connection distance (b). In each sub-figure, cells with the same color belong to the same cluster.

3 Other input data and assumptions

3.1 Provincial electricity demand profiles

Our base case adopts electricity demand forecasts in 2060 from [43], which predicts China's national electricity demand totals reaching 15.4 PWh (about 2.1 times of the total demand in 2020) and provides numbers for each province. We use regional demand profiles in [32] to acquire provincial demand profile shapes by assuming provinces in the same grid region have the same shape. We then scale each province's profile to match its total electricity demand forecast in 2060.

3.2 Firm generators

We adopt the generation layer concept developed in [32] to assign firm generator types into five different layers, the order of which determines the dispatch order. The matching between firm generator types and generation layers is shown in Table S8. However, the generation layers we use in our new modeling framework have two major distinctions from [32]: first, bio-energy carbon capture and sequestration (BECCS) is assigned as L1 (must-run) layer because a fixed amount of its generation must be guaranteed to realize a certain level of negative emissions assumed in our scenario analyses. Second, non-must-run nuclear is assigned to a separate layer to incorporate the (limited) flexibility of nuclear in the future grid.

Table S8: The matching between firm generator types and generation layers

Layer	Generator types
L1	Must-run BECCS, nuclear, and coal CHP CCS
L2	Hydro
L3	Remainder of nuclear
L4	Remainder of coal CHP CCS
L5	Natural gas CCS

Although we assume that coal plants used solely for electricity generation will be completely phased out by 2060, we assume that coal CHP with CCS would still supply winter heating for urban areas in North China with existing central heating infrastructure to fully utilize this massive incumbent investment. We estimate that this type of heat demand would be about 3.1 billion GJ based on forecasts of urban areas in North China, the share of central heating, and heating demand per area provided by [44,45], not significantly different from the demand in 2019 (3.3 billion GJ) [46]. We then acquire the coal CHP with CCS capacity in each province by scaling its coal CHP capacity in 2019 ([47], see Table S9) with the ratio of demand between 2019 and 2060. The ramp rate of coal CCS (CHP and non-CHP) is assumed as 25%/h in this study, more conservative than 35%/h in [42].

Table S9: Coal CHP CCS capacity in north China by province in 2060.

Province	Capacity (GW)	Province	Capacity (GW)
Beijing	17	Heilongjiang	25
Tianjin	15	Shandong	33
Hebei	26	Henan	12
Shanxi	11	Shaanxi	10
East Inner Mongolia	10	Gansu	9
West Inner Mongolia	10	Qinghai	3
Liaoning	34	Ningxia	4
Jilin	18	Xinjiang	11

We think natural gas generation will be pivotal for maintaining the stability of a grid with a high share of VRE. By the end of 2018, China had installed 90.2 GW of natural gas plants with provincial numbers provided in [48]. We scale these numbers to match a national forecast (320 GW) by [34] in 2060. We assume all the natural gas plants will also be equipped with CCS, and their flexibility will be limited by CCS. We adopt the maximum ramp rate as 50%/hour in our base case, which is significantly lower than that of conventional natural gas plants ($\sim$500%/hour in [49]). This value is consistent with choices in existing studies (50%/hour for combined-cycle gas turbines with CCS and 100%/hour for open-cycle gas turbines with CCS in [39] and 50%/hour [42]). We further conduct a sensitivity analysis that assumes an even lower ramp rate (25%/hour) and find the results do not significantly differ from our base case: the wind and solar installations only change by 0.2% and -0.2%, respectively, and the system cost slightly increases by about 2%.

An important assumption we impose in our analysis is that China's power sector needs to support the overall carbon neutrality by offering a significant emission sink with BECCS. We assume roughly 550 million tons (Mt) of negative emissions from the power sector suggested in [43] as a reference and adjust the national BECCS installation by 2060 to match this target, incorporating slight positive emissions (about 100 Mt) from partial capture CHP-CCS and NG-CCS. This negative emission from BECCS is within the range of estimates from IAMs focused on China in the ADVANCE Synthesis project. Under the scenario "2020_1.5C-2100", negative emissions from BECCS in IAMs for China vary from 132–2,815 Mt/yr by 2050 (see detailed values in Table S10)

Table S10: China's carbon sequestration results of BECCS in 2050 from multiple IAM models in scenario (2020_1.5C-2100). Data source: ADVANCE Synthesis Project [50].

Model	CO_2 [Mt/yr]	Model	CO_2 [Mt/yr]
AIM/CGE V.2	1047	MESSAGE-GLOBIOM 1.0	131
GCAM4.2 ADVANCEWP6	1573	POLES ADVANCE	2815
IMACLIM V1.1	2036	REMIND V1.7	1306
IMAGE 3.0	2182	WITCH2016	800

The life-cycle greenhouse gas emission (g/kWh) of BECCS is about -651 from [51] and about -1570 from [52], assuming BECCS only uses agricultural or forestry residues. In this study, we adopt a medium value (-850 g/kWh). With an average capacity factor of 0.8 (about 7,000 annual generation hours) [51], China's total BECCS capacity needs to reach about 110 GW (770 TWh in terms of generation) for about

−650 Mt/yr negative emissions. We estimate that the total demand for agricultural or forestry residues is around 470 million tons, well below China's total potential ($>$ 600 million tons), see [53] and [54]. Assuming a thermal efficiency of 0.35 for bioenergy generator [51], the annual heat consumption of 770 TWh BECCS generation is around 7.9 EJ, far below the 42 EJ of annual bioenergy potential (of which about 20 EJ potential is from residues alone) assessed in [55] and [56], and also lower than the 15.6 EJ of annual bioenergy consumption in [57].

Similar to the wind and solar deployment scheme studied as the main research question in this study, there is no clear planning for the BECCS deployment in China yet. Therefore, we need to apply certain criteria to distribute the targeted −650 Mt/yr negative emissions capacity to different provinces. We adopt one possible BECCS deployment scheme based on existing studies. The criteria that we apply to determine the BECCS capacity distribution by province are the transportation cost of carbon emissions from the source (mainly sites of thermal power) to sites of storage and the availability of biomass resources (preferable forest residues because less land use issues are involved, compared to agricultural-based biomass). Related to the first criterion, [58] identifies regions for CCS development based on source-sink match optimization: Sichuan Basin in Sichuan Province, Jianghan oil field in Hubei Province, Bohai Bay Basin in Hebei Province and Shandong Province, and North Jiangsu in Jiangsu Province are identified as the most preferable regions (hereafter as first-tier regions), followed by Guangxi Province, Yunnan Province, and Songliao Basin in Jilin Province and Heilongjiang Province (hereafter as second-tier regions), see dark-red and red regions in Fig. 4a by [58] (referenced here as Fig. S13a). Given that all the above regions have a total sequestration potential of about 35 Gt (based on the assessment method from [58] and the original geological dataset it adopted [59]), which is enough for decades of sequestration with a rate of −650 Mt/yr, we allocate the BECCS capacity among these regions. Related to the second criterion, [60] provides detailed forest residue potential distribution information (visualized as SI Appendix Fig. 2 in [61], referenced here as Fig. S13b), which can be applied to exclude regions that are lack of forest residue resources. Among the regions identified above, only North Jiangsu is excluded based on this criterion.

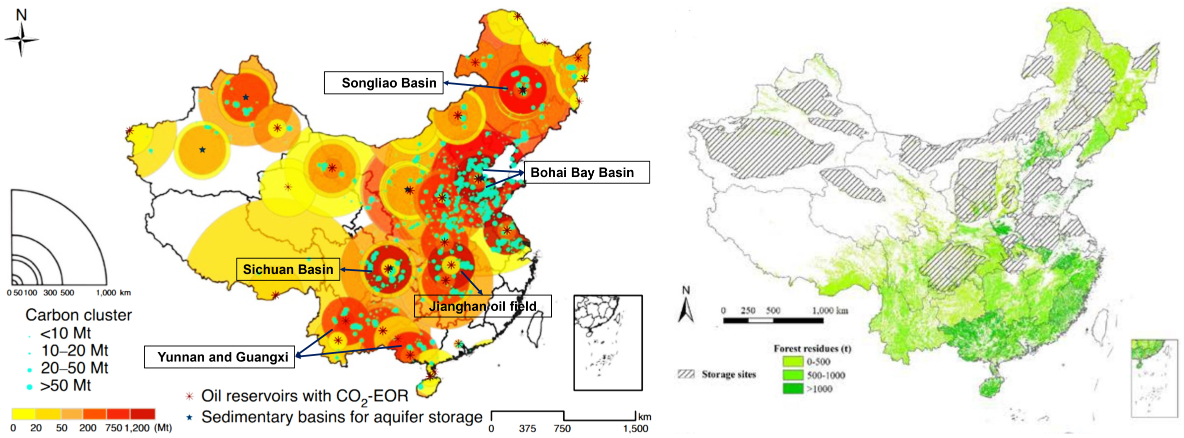

(a) Regions for CCS development identified based on source-sink match optimization

(b) The potential of forest residues

Fig. S13: Regions for CCS development identified based on source-sink match optimization (a, source: [58]) and the potential of forest residues (b, source: [61]) in mainland China.

Finally, we adopt a simple rule to allocate the 110 GW BECCS capacity to the remaining three first-tier regions (Sichuan Basin, Jianghan oil field, and Bohai Bay Basin) and three second-tier regions (Guangxi Province, Yunnan Province, and Songliao Basin), assuming that regions in the same tier have the same capacity and a region in the first-tier region has a capacity twice of the capacity of a second-tier region. For a region that straddles two provinces, we assume these two provinces share the same capacity. We therefore have an allocation scheme of BECCS capacity by province used in our modeling, as shown in Table S11.

Table S11: BECCS capacity by province in 2060.

Province	Sichuan	Hubei	Hebei	Shandong	Guangxi	Yunnan	Jilin	Heilongjiang
Capacity (GW)	25	25	12	12	12	12	6	6

Cost and performance assumptions of CCS are important for the total system cost estimations. There are three main streams of CCS technology: post-combustion capture, oxyfuel, and pre-combustion capture. Since post-combustion capture is the most established one and is predicted to have the largest deployment [60, 62], we choose cost and performance assumptions based on post-combustion capture. The values of CapEx, fixed operation & maintenance (FOM), variable operation & maintenance (VOM), lifetime, and CO_2 capture rate for coal CHP and natural gas CCS are derived from [62], and these for BECCS are from [51]. We also adopt high capital cost assumptions from these references for high CCS cost scenarios. Transport and storage costs for coal CHP and natural gas CCS are given by [62], and we assume this cost for BECCS is the same as that of natural gas CCS. Power loss occurs in generators when combined with CCS technology. [60] suggest power loss between 5% and 8% in 2050 and we assume 5% in this study. Cost and performance parameters are shown in Table S12.

Table S12: Cost and performance parameters of CCS.

Generator	Ref. CapEx (yuan/kW)	High CapEx (yuan/kW)	FOM (yuan/kW·yr)	VOM (yuan/MWh)	Transport and storage cost (yuan/MWh)	Lifetime (yr)	CO_2 captured rate (%)	Ramp rate (%/h)
Coal CCS	17,850	23,940	447	38	49	40	86	25
Natural gas	10,500	14,700	263	28	28	30	86	50
BECCS	15,726	17,118	753	60	28	35	88	-

We adopt the total hydro installation forecast in 2060 (580 GW) by [34], which also provides the share of installation in each region (Southwest: 44.4%, Northwest China: 8.5%, East and Central: 20.9%, and others: 26.2%). By the end of 2020, China has built about 338 GW hydro, with detailed provincial installations derived from [63, 64]. We apply different scaling ratios to provinces in different regions to match total regional hydro installation forecasts in 2060. The CapEx of existing hydro in China ranges from 800 to 1600 $/kW [38]. We use the upper bound of this range (10,000 yuan/kW, about 1,563 $/kW) in this study as the cost of future hydro construction is expected to be higher. We set the fixed O&M cost of hydro at 230 yuan/kW·yr, based on [39] (about 36 $/kW·yr).

Nuclear installation forecasts for China typically assume only coastal builds. One study forecasts that China's nuclear installation will reach 250 GW by 2060, but does not provide detailed location information [34]. Another forecast (218 GW) given by [65] provides detailed location information for each nuclear

group site. As the installation capacities of these two forecasts are close, we adopt the forecast from [65] with its clear location information (see Table S13). The CapEx of existing nuclear projects in China ranges from about 1,800 to about 4,800 $/kW [66], and we choose a medium value (15,000 yuan/kW, about 2,344 $/kW). The fixed O&M cost (630 yuan/kW·yr, about 100 $/kW·yr) is selected based on [39] of nuclear. In our base case, we set the minimum generation of nuclear at 85% of installation capacity, and the maximum hourly ramp rate at 5%, based on [39].

Table S13: Nuclear capacity by province in 2060.

Province	Capacity (GW)	Province	Capacity (GW)
Guangdong	67	Hebei	18
Fujian	29	Jiangsu	16
Guangxi	26	Liaoning	11
Shandong	24	Hainan	4
Zhejiang	24		

In summary, capacity assumptions of firm generators at the national level are shown in Table S14. We further show capacity assumptions of firm generators and peak load (maximum hourly demand throughout the year) by province in Fig. S14.

Table S14: Capacity assumptions of firm generators.

Generator	Capacity (GW)	Generator	Capacity (GW)
Coal CCS	0	Nuclear	218
Coal CHP CCS	248	Hydro	580
BECCS	110	Gas CCS	320

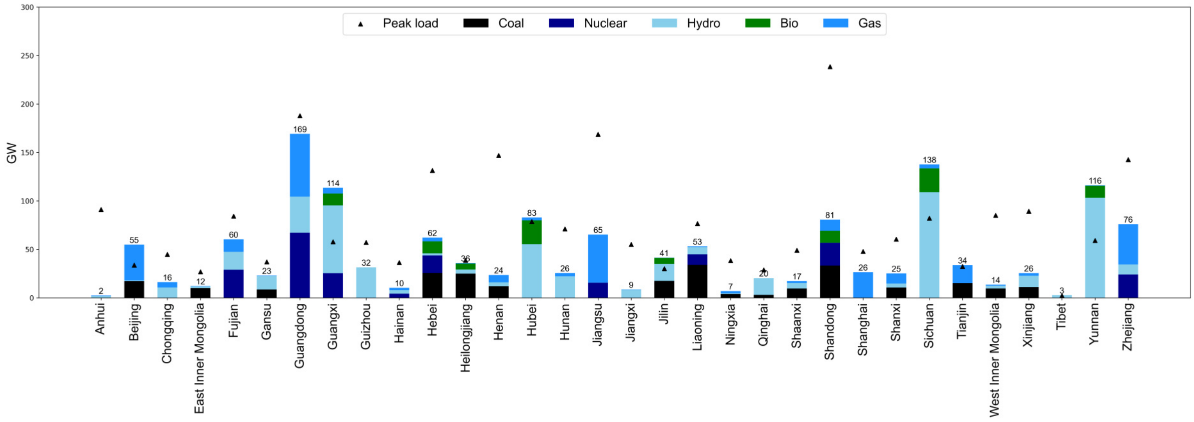

Fig. S14: Capacity of firm generators and peak load of each province in 2060.

3.3 Energy storage

In the base case of our analysis, we consider two storage technologies: pumped hydro storage (PHS) and electrochemical battery storage (BAT). Since the CapEx of PHS in the future is predicted to remain

stable [67], we adopt the CapEx of PHS as 480 yuan/kWh (about 77 \$/kWh) from [67]. Round-trip efficiency (RTE) is the ratio of energy discharged to energy charged, and we assume the charge efficiency and discharge efficiency are both equal to $\sqrt{\text{RTE}}$ when formulating the model. Based on an analysis of PHS by [67, 68], we assume the RTE of the PHS is 78%, the self-discharge rate is 0%, and the duration is 8 hours. The fixed O&M costs and variable O&M cost are assumed to be 39 yuan/kW·yr [69] and 0.0015 yuan/kWh [70], respectively. The life span is assumed to be 40 years [68].

We choose Li-ion battery as the representative BAT technology. The National Renewable Energy Laboratory (NREL) documents that the CapEx of a 4-hour Li-ion battery is about 350 \$/kWh in 2020 and it will decline by 25%–75% by 2050 [71]. We assume the CapEx of BAT in China by 2060 is 675 yuan/kWh (106 \$/kWh), which is optimistic but still higher than the lower limit of the prediction by the NREL. We assume fixed O&M cost is 18 yuan/kW·yr (about 2.8 \$/kW·yr) in 2060 as suggested by [72], roughly the same cost reduction rate as CapEx, compared to the current O&M costs (around 10 \$/kW·yr). We assume variable O&M cost is 20 \$/MWh (3 \$/MWh), which is in line with the range (about 1.2–4.8 \$/MWh) given by [67]. The self-discharge rate is assumed as 2% per month (approximately 0.07%/day) [73]. We assume the RTE is 95% in 2060, a further improvement compared to about 90% at the current level [74]. The life span is assumed to be 15 years [68].

For sensitivity analyses, we design a specific scenario to evaluate the role of long-duration energy storage (LDES), as LDES is expected to play a significant role in better utilizing VREs and lowering the total system cost when VRE penetration is high [75–79]. Compared to the BAT, most LDES technologies have a higher energy-to-power capacity ratio (duration) and lower RTE. We consider two representative LDES technologies in our sensitivity analyses, one with relatively low power capacity cost but also low RTE (e.g., compressed air energy storage, CAES) and the other with relatively high power capacity cost but also high RTE (e.g., vanadium redox-flow battery, VRB).

The forecasts for the CapEx of the CAES has a large range, e.g., 1348 \$/kW by [80], 400–800 \$/kW by [68], and 800–1000 \$/kW by [81]. We use 750 \$/kW (about 4800 yuan/kW) and assume the duration as 20 hours, and thus the CapEx of CAES by energy is around 40 \$/kWh. The fixed O&M cost of CAES is estimated by [80] as 16.3 \$/kw·yr for a 100 MW system, 11.50 \$/kw·yr for a 1,000 MW system, and 10.78 \$/kw·yr for a 10,000 MW system, respectively. Considering the labor cost in China is lower than the US, we assume the fixed O&M cost of CAES is 50 yuan/kw·yr (about 8 \$/kw·yr). The variable O&M cost is assumed as 0.5125 \$/MWh (about 3.3 yuan/MWh) by [80]. We also use the assumption of RTE (52%) by [80]. We choose the middle value (30 years) for the life span based on [68]'s range (20–40 years). The self-discharge rate of CAES is assumed as 1%/day from [82].

We set our assumptions for the VRB parameters using a typical type with 100 MW and 10 hours duration. The CapEx cost of VRB in 2020 ranges from 600 to 1500 \$/kW by [83]. A study [67] forecasts that the CapEx in 2050 will be 49% of that in 2020. Assuming the cost in 2060 will be the same as 2050, the CapEx would be around 470 \$/kW (about 3000 yuan/kW, 300 yuan/kWh) in 2060. For the fixed O&M cost, we assume it would be roughly the same as that of Li-ion battery in 2060 (2.8 \$/kW·yr, about 18 yuan/kW·yr), a significant reduction compared to the current level (7–16 \$/kW·yr in [72] and 12 \$/kW·yr in [67]). The variable O&M cost is also assumed to be the same as that of Li-ion battery

following [67] (3 $/MWh, about 20 yuan/MWh). The RTE (70%) and life span (15 years) values are also adopted from [80]. The self-discharge rate of flow battery is reported from 0.5% to 0.7% per day by [82], and we use 0.6%/day in this study.

Cost and performance parameters for each storage technology adopted in this study are shown in Table S15.

Table S15: Cost and performance parameters for storage technologies.

Technology	CapEx cost [yuan/kWh]	Fixed O&M cost [yuan/MW·yr]	Variable O&M cost [yuan/MWh]	Round-trip efficiency [%]	Self-discharge rate [%/day]	Duration [h]	Life span [yrs]
PHS	480	39,000	1.5	78	0	8	40
BAT	675	18,000	20	95	0.07	4	15
CAES	240	50,000	3	52	1	20	30
VRB	300	18,000	20	70	0.6	10	15

We do not set any deployment constraints for the BAT or LDES. For the PHS, however, deployment depends on geological conditions. We use the latest nationwide pumped storage planning by provincial energy authorities in 2020 organized by the National Energy Administration (NEA) [84]. In the planning process, factors like geography, geology, water resources, submergence areas, environmental impacts, and technical conditions were comprehensively evaluated. Over 1,500 potential sites were identified in China, with a total capacity of around 1,600 GW. Building on this, the NEA proposed priority projects for long-term development totaling about 679 GW, with the provincial breakdown shown in Table S16. Since China has 32.5 GW of PHS installed by the end of 2020 and 54 GW under construction [64], we set a minimum capacity in 2060 for each province (national totals: 86.5 GW) as well as a long-term potential capacity by province (national totals: 765 GW).

Table S16: Installed capacity, under construction capacity and long-term potential capacity (MW) of the PHS by province in mainland China.

Province	Installed capacity	Under construction capacity	Long-term potential capacity
Anhui	3,480	2,480	27,760
Beijing	800	0	800
Chongqing	0	1,200	9,600
Fujian	0	1,200	6,200
Guangdong	1,200	5,600	6,800
Gansu	0	0	33,500
Guangxi	7,280	2,400	45,480
Guizhou	0	0	22,800
Henan	0	0	40,600
Hubei	600	0	8,800
Hebei	1,270	7,400	27,470
Hainan	0	1,200	42,500
Heilongjiang	1,320	3,600	28,620
Hunan	1,270	0	37,670
Jilin	1,200	1,400	35,410
Jiangsu	2,600	1,350	7,550
Jiangxi	1,200	1,200	15,410
Liaoning	650	2,250	35,200
East Inner Mongolia	1,200	2,800	22,600
West Inner Mongolia	0	0	7,800
Ningxia	0	0	40,300
Qinghai	0	1,400	36,950
Sichuan	1,000	6,000	20,600
Shandong	0	0	0
Shanghai	1,200	2,700	18,900
Shaanxi	0	0	14,600
Shanxi	0	0	2,400
Tianjin	1,200	0	7,900
Xinjiang	0	2,400	3,9000
Tibet	90	0	72,040
Yunnan	0	0	0
Zhejiang	4,930	7,350	50,275
Total	32,490	53,930	765,535

3.4 Inter-provincial transmission

3.4.1 Strengthen existing transmission line

In our base case, we assume inter-provincial transmission lines can be built to strengthen existing inter-provincial links. We show the capacity of existing inter-provincial transmission lines in China by 2020 in Fig. S15, based on the data collected from the State Grid and China Southern Power Grid. For provinces that do not have existing links, we assume no new lines would be built.

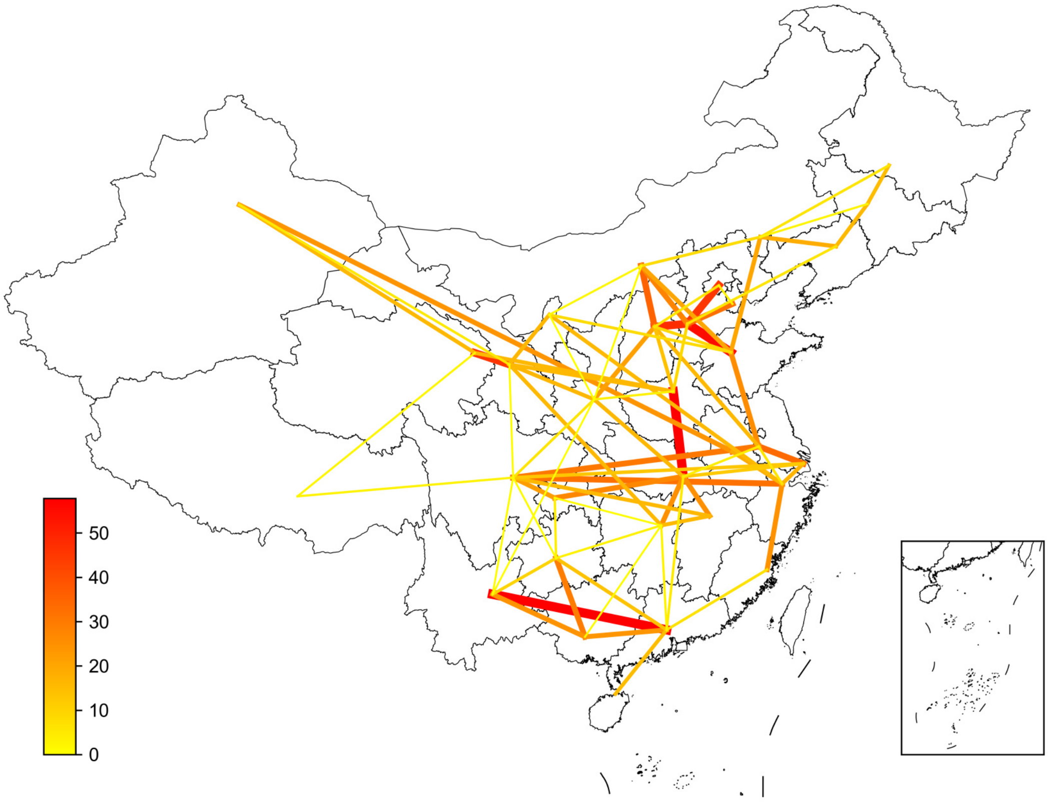

Fig. S15: Capacity (GW) of existing inter-regional transmission lines.

The cost of inter-provincial transmission line per unit of capacity-distance depends on its voltage level. We adopt the cost assumption on the CapEx of transmission line and substation by voltage level from [85], shown in Table S17.

Table S17: CapEx assumptions of substations and overhead lines.

Voltage [kV]	±1100	1000	±800	750	±500	500	330
Substation [yuan/kW]	637	370	592	148	912	159	241
Overhead line [1000 yuan/km]	7,000	7,080	4,950	2,990	1,986	2,640	1,214

To determine the voltage level of the transmission line between two specific provinces, we collect the voltage level information of existing transmission lines connecting these two provinces and assume the highest would be the voltage level for new lines built in the future. For a few lines with voltage level lower than 500 kV (due to small capacity at the current level), we assume 500 kV lines will be built in the future. The resulting voltage level matrix is shown in Table S18. The distance between two provinces is calculated as the geographic distance between their capitals.

Table S18: Voltage level of new planning inter-provincial transmission line.

	HL	JL	LN	MD	SN	GS	QH	NX	XJ	XZ	HE	BJ	TJ	SX	SD	MX	HB	HN	JX	HA	CQ	SC	GD	HI	GX	GZ	YN	SH	JS	AH	FJ	ZJ
HL		500		500																												
JL	500		500	500																												
LN	500	500		500							500																					
MD	500	500	500												±800	500																
SN						750		500						1000		500	±800			500		500					500					
GS					750		800	750	750									800		800		500										
QH						±800			±800	500										800												
NX					500	750									750	500																800
XJ						750	±800																							1000		
XZ							500															500										
HE			500									1000	500	1000	1000	500				500												
BJ											1000		500	500																		
TJ											1000	500																				
SX					1000						1000	500			500	1000				1000									±800			
SD				±800				750			1000			500		±800													500			
MX				500	500			500			1000			1000	800																	
HB					±800													1000	±800	1000	1000		500					500	500			
HN						±800											1000		±800		500		500		500	500						
JX																	500	500				800										
HA					500	±800	±800				500			1000			1000															
CQ																	1000	500				1000				220						
SC					500	500				500									800		1000					500	500	±800	±800			±800
GD																	500	500						500	500	500	±800				500	
HI																							500									
GX																		500					500			500	500					
GZ																		500			500	500	500		500		500					
YN					500																	500	±800		±800	500						
SH																	500					800							1000			1000
JS														±800	±800		500					800						1000		1000		500
AH									1000																				1000			1000
FJ																							500									1000
ZJ								±800														±800						1000	500	1000	1000	

3.4.2 Unconstrained UHV expansion

In addition to our base case, we model an “Unconstrained UHV Expansion” scenario that allows any two provinces to construct new inter-provincial transmission lines, with the caveat that Tibet and Hainan are limited to connecting only to neighboring provinces owing to the challenges of estimating costs and building lines over heavily mountainous terrain and over the sea, respectively. For inter-provincial lines already constructed, we model the transmission technology according to the specifications in Section S3.4.1. To determine the optimal AC/DC configuration and voltage levels for new lines, we implement a pre-optimization procedure following the approach of [42]. First, we consider 330 kV, 500 kV, 750 kV, and 1000 kV for AC lines, and ±500 kV, ±800 kV, and ±1100 kV for DC lines, with AC 330 kV and AC 750 kV only suitable for the Northwest region [42]. Second, maximal transmission distances for AC lines by voltage level are applied. For the remaining AC and DC options, we calculate the total cost per kW and select the voltage level with the lowest unit cost for both AC and DC as the optimal expansion choice. The total cost for each voltage level includes overhead lines and substations/converters, with CapEx values shown in Table S17. To determine the total substation/converter cost and unit cost (yuan/kW) for each DC voltage, we assume fixed capacities (3000 MW for ±500 kV, 8000 MW for ±800 kV, and 12000 MW for ±1100 kV) [42]. The transmission capacity of AC lines is related to distance and voltage level. Based on capacity and distance data of existing lines, we use a polynomial fitting model to determine the numerical relationship between them [42], with results shown in Fig. S16. Finally, we present the pre-optimized choice (AC/DC and voltage level) of newly built inter-provincial lines in Table S19.

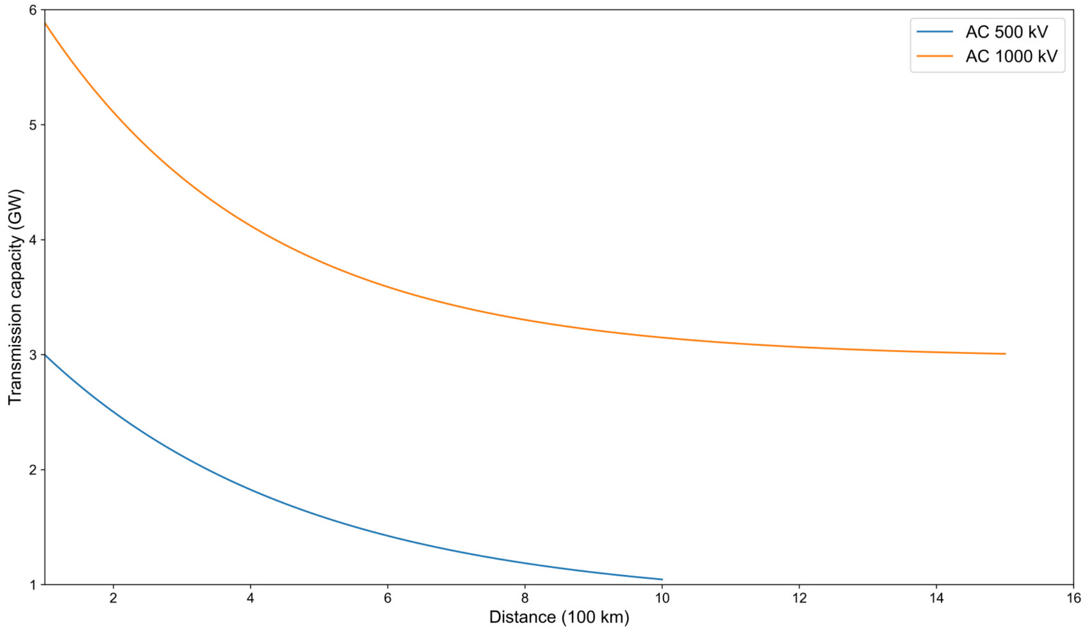

Fig. S16: Relationship between transmission capacity and distance for 500kV and 1000kV AC lines [42]

Table S19: Inter-provincial transmission corridor options for newly expanding.

Province	AH	BJ	FJ	GS	GD	GX	GZ	HN	HB	HA	HL	HB	HN	JL	JS	JX	LN	NX	QH	SD	SX	SN	SH	SC	TJ	XJ	YN	ZJ	CQ	MX	MD	XZ
AH	-	±800	±800	±1100	±800	±800	±800	-	±800	500	±1100	500	±800	±1100	1000	500	±800	±800	±1100	500	±800	±800	500	±1100	±800	±1100	±1100	1000	±800	±1100	±1100	-
BJ	±800	-	±1100	±800	±1100	±1100	±1100	-	1000	±800	±800	±800	±1100	±800	±800	±1100	500	±800	±1100	500	500	±800	±800	±1100	500	±1100	±1100	±1100	±1100	500	±800	-
FJ	±800	±1100	-	±1100	500	±800	±800	-	±1100	±800	±1100	±800	±800	±1100	±800	500	±1100	±1100	±1100	±800	±1100	±1100	±800	±1100	±1100	±1100	±1100	1000	±800	±1100	±1100	-
GS	±1100	±800	±1100	-	±1100	±1100	±800	-	±800	±800	±1100	±800	±800	±1100	±1100	±1100	±1100	750	±800	±1100	±800	750	±1100	500	±800	750	±1100	±1100	±800	±800	±1100	-
GD	±800	±1100	500	±1100	-	500	500	500	±1100	±800	±1100	500	500	±1100	±1100	500	±1100	±1100	±1100	±1100	±1100	±1100	±800	±1100	±1100	±1100	±800	±800	±800	±1100	±1100	-
GX	±800	±1100	±800	±1100	500	-	500	500	±1100	±800	±1100	±800	500	±1100	±1100	±800	±1100	±1100	±1100	±1100	±1100	±1100	±1100	±800	±1100	±1100	±800	±800	±800	±1100	±1100	-
GZ	±800	±1100	±800	±800	500	500	-	-	±1100	±800	±1100	±800	500	±1100	±1100	±800	±1100	±800	±1100	±1100	±1100	±800	±1100	500	±1100	±1100	500	±1100	500	±1100	±1100	-
HN	-	-	-	-	500	500	-	-	-	-	-	-	-	-	-	-	-	-	-	-	-	-	-	-	-	-	-	-	-	-	-	-
HB	±800	1000	±1100	±800	±1100	±1100	±1100	-	-	500	±1100	±800	±1100	±800	±800	±1100	500	±800	±1100	1000	1000	±800	±800	±1100	1000	±1100	±1100	±800	±800	1000	±800	-
HA	500	±800	±800	±800	±800	±800	±800	-	500	-	±1100	1000	±800	±1100	±800	±800	±800	±800	±800	500	1000	500	±800	±800	±800	±1100	±1100	±800	±800	±800	±1100	-
HL	±1100	±800	±1100	±1100	±1100	±1100	±1100	-	±1100	±1100	-	±1100	±1100	500	±1100	±1100	±800	±1100	±1100	±1100	±1100	±1100	±1100	±1100	±1100	±1100	±1100	±1100	±1100	±1100	500	-
HB	500	±800	±800	±800	500	±800	±800	-	±800	1000	±1100	-	1000	±1100	500	±800	±1100	±800	±1100	±800	±800	±800	500	±800	±800	±1100	±1100	±800	1000	±800	±1100	-
HN	±800	±1100	±800	±800	500	500	500	-	±1100	±800	±1100	1000	-	±1100	±800	±800	±1100	±800	±1100	±800	±800	±800	±800	±800	±1100	±1100	±800	±800	500	±1100	±1100	-
JL	±1100	±800	±1100	±1100	±1100	±1100	±1100	-	±800	±1100	500	±1100	±1100	-	±1100	±1100	500	±1100	±1100	±800	±1100	±1100	±1100	±1100	±800	±1100	±1100	±1100	±1100	±1100	500	-
JS	1000	±800	±800	±1100	±1100	±1100	±1100	-	±800	±800	±1100	500	±800	±1100	-	±800	±800	±1100	±1100	±800	±800	±800	1000	±800	±800	±1100	±1100	500	±800	±1100	±1100	-
JX	500	±1100	500	±1100	500	±800	±800	-	±1100	±800	±1100	±800	±800	±1100	±800	-	±1100	±1100	±1100	±800	±800	±800	±800	±800	±1100	±1100	±1100	500	±800	±1100	±1100	-
LN	±800	500	±1100	±1100	±1100	±1100	±1100	-	500	±800	±800	±1100	±1100	500	±800	±1100	-	±1100	±1100	±800	±800	±1100	±800	±1100	500	±1100	±1100	±1100	±1100	±800	500	-
NX	±800	±800	±1100	750	±1100	±1100	±800	-	±800	±800	±1100	±800	±800	±1100	±1100	±1100	±1100	-	±800	660	±800	750	±1100	±800	±800	750	±1100	±800	±800	±800	±1100	-
QH	±1100	±1100	±1100	±800	±1100	±1100	±1100	-	±1100	±800	±1100	±1100	±1100	±1100	±1100	±1100	±1100	±800	-	±1100	±1100	750	±1100	±800	±1100	±800	±800	±1100	±800	±1100	±1100	500
SD	500	500	±800	±1100	±1100	±1100	±1100	-	1000	500	±1100	±800	±800	±800	±800	±800	±800	660	±1100	-	500	±800	±800	±1100	±800	±1100	±1100	±800	±800	±800	±800	-
SX	±800	500	±1100	±800	±1100	±1100	±1100	-	1000	1000	±1100	±800	±800	±1100	±800	±800	±800	±800	±1100	500	-	1000	±800	±800	500	±1100	±1100	±800	±800	1000	±1100	-
SN	±800	±800	±1100	750	±1100	±1100	±800	-	±800	500	±1100	±800	±800	±1100	±800	±800	±1100	750	750	±800	1000	-	±800	500	±800	750	500	±800	±800	±800	±1100	-
SH	500	±800	±800	±1100	±800	±1100	±1100	-	±800	±800	±1100	500	±800	±1100	1000	±800	±800	±1100	±1100	±800	±800	±800	-	±800	±800	±1100	±1100	1000	±1100	±1100	±1100	-
SC	±1100	±1100	±1100	500	±1100	±800	500	-	±1100	±800	±1100	±800	±800	±1100	±800	±800	±1100	±800	±800	±1100	±800	500	±800	-	±1100	±1100	500	±800	1000	±1100	±1100	500
TJ	±800	500	±1100	±800	±1100	±1100	±1100	-	1000	±800	±1100	±800	±1100	±800	±800	±1100	500	±800	±1100	±800	500	±800	±800	±1100	-	±1100	±1100	±800	±1100	±800	±800	-
XJ	±1100	±1100	±1100	750	±1100	±1100	±1100	-	±1100	±1100	±1100	±1100	±1100	±1100	±1100	±1100	±1100	750	±800	±1100	±1100	750	±1100	±1100	±1100	-	±1100	±1100	±1100	±1100	±1100	±800
YN	±1100	±1100	±1100	±1100	±800	±800	500	-	±1100	±1100	±1100	±1100	±800	±1100	±1100	±1100	±1100	±1100	±800	±1100	±1100	500	±1100	500	±1100	±1100	-	±1100	±800	±1100	±1100	±1100
ZJ	1000	±1100	1000	±1100	±800	±800	±1100	-	±800	±800	±1100	±800	±800	±1100	500	500	±1100	±800	±1100	±800	±800	±800	1000	±800	±800	±1100	±1100	-	±800	±1100	±1100	-
CQ	±800	±1100	±800	±800	±800	±800	500	-	±800	±800	±1100	1000	500	±1100	±800	±800	±1100	±800	±800	±800	±800	±800	±1100	1000	±1100	±1100	±800	±800	-	±1100	±1100	-
MX	±1100	500	±1100	±800	±1100	±1100	±1100	-	1000	±800	±1100	±800	±1100	±1100	±1100	±1100	±800	±800	±1100	±800	1000	±800	±1100	±1100	±800	±1100	±1100	±1100	±1100	-	500	-
MD	±1100	±800	±1100	±1100	±1100	±1100	±1100	-	±800	±1100	500	±1100	±1100	500	±1100	±1100	500	±1100	±1100	±800	±1100	±1100	±1100	±1100	±800	±1100	±1100	±1100	±1100	500	-	-
XZ	-	-	-	-	-	-	-	-	-	-	-	-	-	-	-	-	-	-	500	-	-	-	-	500	-	±800	±1100	-	-	-	-	-

4 Model formulation

Our model is formulated to find the optimized layout of new VRE installations in 2060 compatible with an economy-wide carbon neutrality for China, which requires its power sector to achieve a significant amount negative emissions with exogenous generation capacity of BECCS, coal CHP CCS, gas CCS, hydro, and nuclear.

Although the model adopts an important formulation that determines the dispatch order by aggregating generation technologies in each province into generation layers with dispatch orders similar to [32], we have introduced several important extensions and adjustments to address specific challenges in our study.

First, unlike [32] that adopt a heuristic algorithm, we have set up a full optimization problem to search for the global optimal layout of VRE technologies in all the grid cells in China. Although we adopt the layer dispatch model similar to [32], the capacity of the must-run layer (L1) in our model is pre-determined to meet the negative emissions requirement (for BECCS) or winter heating demand (for CHP) in each province. Second, we represent connecting costs of VREs in each grid cell in detail and fully consider the potential of profile complementarities across cells and between wind and solar to reduce costs through bundling transmission. Third, we represent several storage technologies, including pumped-hydro, battery, and long-duration energy storage, which would be crucial for high penetration of VREs. We provide a full description of the model formulation as below.

4.1 Definitions

As the introduction of generation layers (see section S3.2), we need to distinguish the operation and cost parameters of generation layers and firm generators. There is only one generator technology in the generation layers (L2–L5), and the must-run layer is pre-determined so that the operation constraints about the generation layers (L2–L5) refer to the corresponding generator technology (see Table S8). CapEx of firm generators and variable costs of must-run generation are constant terms in the objective function (see section S4.2) which can be calculated separately. Based on this, we use decision variables and parameters of generation layers to present the model formulation.

Table S20: Model indices.

Index	Description
r	Province (shown in Table S2)
$\widetilde{r}$	Province (alias of r, only used alongside r)
gr	Grid region (shown in Table S2)
v	VRE technology type (onshore wind, offshore wind, utility-scale solar, or distributed solar)
i	Cell for a VRE technology type in a province
sub	Substation
lc	Load center in mainland China
s	Energy storage technology type (PHS, BAT, CAES, or VRB)
l	Generation layer (shown in Table S8)
t	Time step (length of 1 hour)

Table S21: Model sets.

Index	Description
R	Set of provinces
R_{gr}	Subset of R, representing provinces in a grid region gr
GR	Set of grid regions
V	Set of VRE technology types
$I_{r,v}$	Set of cells for VRE technology type v in province r
$I_{r,v,sub}$	Set of cells for VRE technology type v connected to substation sub in province r
Sub	Set of substations
LC	Set of load centers in mainland China
S	Set of storage technologies
L	Set of generation layers
T	Set of time steps

Table S22: Model parameters.

Index	Description	Unit
C_l^{ru}	Unit ramp up cost for generation layer l	[yuan/kW]
C_l^{rd}	Unit ramp down cost for generation layer l	[yuan/kW]
C_l^{resv}	Unit reserve cost using generation layer l	[yuan/kW]
C_l^{fuel}	Unit fuel cost for generation layer l	[yuan/kWh]
C_l^{VOM}	Unit variable O&M cost for generation layer l	[yuan/kWh]
C_s	CapEx of storage s	[yuan/kW]

Table S22 continued from previous page

Index	Description	Unit
C_s^{FOM}	Fixed O&M cost of storage s	[yuan/kW·yr]
C_s^{VOM}	Variable O&M cost of storage s	[yuan/kWh]
$C_{r\widetilde{r}}^{Fixed}$	Fixed CapEx of transmission line between r and $\widetilde{r}$	[yuan/kW]
$C_{r\widetilde{r}}^{Var}$	Variable CapEx of transmission line between r and $\widetilde{r}$	[yuan/kW-km]
$CAP_{r,l,t}$	Maximum capacity of generation layer l in province r	[GW]
$CAP_{r,v,i}$	Maximum capacity potential of the i-th VRE v cell in province r	[GW]
$CAP_{r\widetilde{r}}^{0}$	Capacity of existing transmission line between r and $\widetilde{r}$	[GW]
$CAP_{r,PHS}^{\max}$	Maximum capacity of PHS to be developed in province r	[GW]
$CAP_{r,PHS}^{0}$	Existing capacity of PHS in province r	[GW]
M_r^{CHP}	Big-M used to determine the coal CHP CCS if online in province r	[GW]
$af_{r,l,t}$	Hourly availability factor of generation layer l in province r and time step t time-variant values for l=L2, hydro; 1 for other generation layers	[fraction]
$af_{r,v,i,t}$	Hourly availability factor of i-th cell for VRE type v in province r and time step t	[fraction]
$D_{r\widetilde{r}}$	Geodesic distance of transmission line between r and $\widetilde{r}$	[km]
$D_{r,i}^{sub}$	Geodesic distance connecting the i-th VRE v cell to the nearest substation sub in province r	[km]
$D_{r,sub}^{lc}$	Geodesic distance connecting the substation in province r to the nearest load center lc	[km]
$DEM_{r,t}$	Hourly electricity demand in province r and time step t	[GW]
$DEM_{r,t}^{nmr}$	Hourly demand net of must-run capacity in province r and time step t	[GW]
$X_{r,v,i}$	The ratio of integrated capacity to total development potential capacity in i-th VRE v cell in province r	[fraction]
$\mathcal{L}_{r,v,i}$	LCOE of the i-th VRE v cell in province r	[yuan/kWh]
ξ_s	CRF of storage s	[fraction/yr]
$\xi_{r\widetilde{r}}$	CRF of transmission line from province r to $\widetilde{r}$	[fraction/yr]
ξ_{spur}	CRF of spur line	[fraction/yr]
ξ_{trunk}	CRF of trunk line	[fraction/yr]
μ_l^{up}	Maximum ramp up rate of generation layer l	[%]
μ_l^{dn}	Maximum ramp down rate of generation layer l	[%]
η_s^c	Charge efficiency of storage s	[%]
η_s^d	Discharge efficiency of storage s	[%]
θ	Minimum output rate of coal CHP CCS if online during the non-must run period	[%]
ζ_s	Hourly self-discharge rate of storage s	[%]
ρ_0	Required rate for load reserve capacity (see section S4.3.5 for details)	[%]
ρ_1	Required rate for VRE reserve capacity (see section S4.3.5 for details)	[%]
Δ_s	Duration of storage s	[h]

Table S22 continued from previous page

Index	Description	Unit
Δ_t	Length of time step (1 hour)	[h]
$\varphi_{r\widetilde{r}}$	Power loss rate of transmission line between r and $\widetilde{r}$	[%/km]
φ_l	Power loss rate of generation l if using CCS	[%]

Availability factor in Table S22 refers to the maximum available power generation divided by capacity in a time step (1 hour). For VRE, availability factor equals the hourly capacity factor assessed in sections S2.1 and S2.2.

Table S23: Model investment decision variables.

Index	Description	Unit
$x_{r,v,i}$	Share of potential of the i-th VRE v cell in province r to be developed	[fraction]
$cap^{sub}_{r,v,i}$	Capacity of spur line connecting the i-th VRE v cell in province r to substation sub	[GW]
$cap^{lc}_{r,sub}$	Capacity of trunk line connecting the substation sub in province r to load center lc	[GW]
$cap_{r\widetilde{r}}$	Capacity of transmission line between r and $\widetilde{r}$	[GW]
$cap^{sto}_{r,s}$	Capacity of storage s in province r	[GW]

Table S24: Model operation decision variables.

Index	Description	Unit
$inte_{r,v,t}$	Generation from VRE v integrated to grid in province r and time step t	[GW]
$trans_{r,\lambda,t}$	Power transmitted from generator λ (VRE or generation layer L2–L5) in province r and time step t	[GW]
$F_{r\widetilde{r},t}$	Power flow along the transmission line from province r to $\widetilde{r}$ in time step t	[GW]
$sto^{c}_{r,s,\lambda,t}$	Power charged into storage s in province r and time step t by generator λ	[GW]
$sto^{d}_{r,s,t}$	Power discharged from storage s in province r and time step t	[GW]
$sto^{e}_{r,s,t}$	Total energy stored in storage s in province r and at the end of time step t	[GWh]
$resv_{r,s,t}$	Reserve capacity from storage s in province r and time step t	[GW]
$load_{r,l,t}$	Load of generation layer l in province r and time step t	[GW]
$resv_{r,l,t}$	Operating reserves provided by non-must-run generation layer l in province r and time step t	[GW]
$cap^{ru}_{r,l,t}$	Hourly ramp-up capacity of generation layer l in province r and time step t	[GW]
$cap^{rd}_{r,l,t}$	Hourly ramp-down capacity of generation layer l in province r and time step t	[GW]
$z^{CHP}_{r,t}$	Binary variable used with big-M to determine coal CHP CCS if online in province r and time step t	[01]

4.2 Objective function

The objective function minimizes the total annualized capital cost and operational cost related to deploying new VRE (wind and solar) in China's power system that can support economy-wide carbon neutrality in a target year, i.e., 2060, building on existing infrastructure (e.g., transmission lines and existing VRE

installations) and necessary firm capacity installations (e.g., BECCS, coal CCS, gas CCS, hydro, and nuclear).

The total annualized capital cost and operating cost related to deploying new VRE include generation cost of newly installed VRE, grid connection cost of these new installations, cost of new inter-provincial transmission lines, the fuel cost of firm capacity outside must-run hours, ramping cost of firm capacity, cost of reserve capacity, and cost of energy storage capacity.

$$\min : f = \sum_r \sum_v \sum_i \mathcal{L}_{r,v,i} \times x_{r,v,i} \times \sum_t af_{r,v,i,t} \times CAP_{r,v,i} \quad \text{(S4-1a)}$$

$$+ \sum_{r\tilde{r}} \xi_{r\tilde{r}} \times (C^{Var}_{r\tilde{r}} \times D_{r\tilde{r}} + C^{Fixed}_{r\tilde{r}}) \times cap_{r\tilde{r}} \quad \text{(S4-1b)}$$

$$+ \sum_r \sum_v \sum_i \xi_{spur} \times (C^{Fixed}_{spur} + C^{Var}_{spur} \times D^{sub}_{r,i}) \times cap^{sub}_{r,v,i} \quad \text{(S4-1c)}$$

$$+ \sum_r \sum_{sub} \xi_{trunk} \times (C^{Fixed}_{trunk} + C^{Var}_{trunk} \times D^{lc}_{r,sub}) \times cap^{lc}_{r,sub} \quad \text{(S4-1d)}$$

$$+ \sum_r \sum_s [(\xi_s \times C_s + C^{FOM}_s) \times cap^{sto}_{r,s} + \sum_t C^{VOM}_s \times sto^d_{r,s,t}] \quad \text{(S4-1e)}$$

$$+ \sum_r \sum_{l \neq L1} \sum_t (C^{fuel}_l + C^{VOM}_l) \times load_{r,l,t} \quad \text{(S4-1f)}$$

$$+ \sum_r \sum_{l \neq L1} \sum_t (C^{ru}_l \times cap^{ru}_{r,l,t} + C^{rd}_l \times cap^{rd}_{r,l,t}) \quad \text{(S4-1g)}$$

$$+ \sum_r \sum_{l \neq L1} \sum_t C^{resv}_l \times resv_{r,l,t} \quad \text{(S4-1h)}$$

$$+ \mathcal{C}, \quad \text{(S4-1i)}$$

where,

S4-1a is annualized cost of VRE installations;

S4-1b is annualized cost of inter-provincial transmission;

S4-1c is annualized cost of spur line transmission;

S4-1d is annualized cost of trunk line transmission;

S4-1e is annualized cost of storage capacity;

S4-1f is total variable cost of firm generators in a year (outside must-run hours);

S4-1g is total ramping cost of firm generators in a year;

S4-1h is total cost of reserve capacity in a year;

S4-1i is a constant term that represents firm generators' total annualized capital cost and variable costs in must-run hours.

4.3 Constraints

4.3.1 Demand

The total supply of electricity in each province in each time step should be equal to the total demand for electricity. Since a fraction of demand is met by pre-determined must-run capacity, we only need to balance the residual demand, $DEM^{nmr}_{r,t}$, by other layers of firm generators (L2–L5), VRE generation, energy storage system discharging, and power transmitted from other provinces. The demand balance constraints are expressed as follows:

$$\sum_{l=L2}^{L5} load_{r,l,t} + \sum_{\widetilde{r}} F_{\widetilde{r}r,t} + \sum_{v} inte_{r,v,t} + \sum_{s} sto^{d}_{r,s,t} = DEM^{nmr}_{r,t}, \forall r, t. \quad \text{(S4-2)}$$

4.3.2 VRE output

Generation from VRE can be integrated into the local grid, transmitted to other provinces, or charged into energy storage systems. In each province, power output from each VRE type should be less than or equal to the maximum power capacity supplied by the VRE plant in each time step t:

$$inte_{r,v,t} + trans_{r,v,t} + \sum_{s} sto^{c}_{r,s,v,t} \leq \sum_{i} x_{r,v,i} \times af_{r,v,i,t} \times CAP_{r,v,i}, \forall r, v, t, \quad \text{(S4-3)}$$

where $x_{r,v,i} \in [0, 1]$. For each cell, the total installed capacity chosen by the model should be greater than or equal to the existing capacity, meaning that the decision variable $x_{r,v,i}$ should be greater than or equal to the parameter $X_{r,v,i}$:

$$x_{r,v,i} \geq X_{r,v,i}, \forall r, v, i. \quad \text{(S4-4)}$$

4.3.3 Dispatchable supply

For each generation layer l, supply load to meet the demand and for reserve capacity, for transmission to other provinces, and for charging storage systems should be less than or equal to the maximum capacity of the corresponding generation layer in each time step t:

$$load_{r,l,t}/(1 - \varphi_l) + resv_{r,l,t} + trans_{r,l,t} + \sum_{s} sto^{c}_{r,s,l,t} \leq af_{r,l,t} \times CAP_{r,l,t}, \forall r, l \in \{L2, \ldots, L5\}, t, \quad \text{(S4-5)}$$

where φ_l is the energy penalty for CCS-equipped layers.

The minimum output of coal CHP CCS should be constrained if online (supply electricity, transmitted to other provinces or served as reserve capacity) during the non-must run period:

$$load_{r,L4,t} + trans_{r,L4,t} + resv_{r,L4,t} \leq z^{CHP}_{r,t} \times M^{CHP}_{r}, \forall r, t, \quad \text{(S4-6)}$$

$$z^{CHP}_{r,t} \times \theta \times CAP_{r,L4,t} \leq load_{r,L4,t} + trans_{r,L4,t}, \forall r, t. \quad \text{(S4-7)}$$

In equation S4-6 and S4-7, $z_{r,t}^{CHP} = 1$ means the coal CHP is online and $z_{r,t}^{CHP} = 0$ means it is offline in province r and time step t.

4.3.4 Transmission operation

Intra-provincial transmission. The capacity of each spur line should be larger than or equal to the maximum output of VRE installations in its corresponding cell in the model period,

$$cap_{r,v,i}^{sub} \geq x_{r,v,i} \times \max_{t \in T}[af_{r,v,i,t}] \times CAP_{r,v,i}, \forall r, v, i. \tag{S4-8}$$

The capacity of each trunk line should be larger than or equal to the maximum output of all the VRE installations that connect to its corresponding substation in the model period,

$$cap_{r,sub}^{lc} \geq \sum_{v} \sum_{i}^{I_{r,v,sub}} x_{r,v,i} \times CAP_{r,v,i} \times \max_{t \in T}[\frac{\sum\limits_{v} \sum\limits_{i}^{I_{r,v,sub}} af_{r,v,i,t} \times CAP_{r,v,i}}{\sum\limits_{v} \sum\limits_{i}^{I_{r,v,sub}} CAP_{r,v,i}}], \forall r, sub, \tag{S4-9}$$

where the term

$$\sum_{v} \sum_{i}^{I_{r,v,sub}} x_{r,v,i} \times CAP_{r,v,i} = \frac{\sum\limits_{v} \sum\limits_{i}^{I_{r,v,sub}} x_{r,v,i} \times CAP_{r,v,i}}{\sum\limits_{v} \sum\limits_{i}^{I_{r,v,sub}} CAP_{r,v,i}} \times \sum_{v} \sum_{i}^{I_{r,v,sub}} CAP_{r,v,i}$$

determines the total VRE capacity installed in grid cells that connect to the substation sub.

Inter-provincial transmission. We apply the pipeline model (also called transportation model) to simulate the inter-provincial transmission flow to avoid introducing binary variables, which has been validated in [86] and used in many mid-long term power planning models [39, 42, 87],

$$\sum_{r\widetilde{r}} F_{r\widetilde{r},t}/(1 - \varphi_{r\widetilde{r}})^{D_{r\widetilde{r}}} = \sum_{\lambda} trans_{r,\lambda,t}, \forall r, t. \tag{S4-10}$$

New transmission capacity from province r to $\widetilde{r}$ is allowed if there is an existing line (i.e. $CAP_{r\widetilde{r}}^{0} > 0$, see Fig. S15). Power transmission from province r to $\widetilde{r}$ and the reverse direction in each time step t combined should be less than or equal to the total transmission capacity:

$$0 \leq F_{r\widetilde{r},t}/(1 - \varphi_{r\widetilde{r}})^{D_{r\widetilde{r}}} + F_{\widetilde{r}r,t}/(1 - \varphi_{\widetilde{r}r})^{D_{\widetilde{r}r}} \leq cap_{r\widetilde{r}} + cap_{\widetilde{r}r}, \forall r\widetilde{r}, \widetilde{r}r, t, \tag{S4-11}$$

$$cap_{r\widetilde{r}} \geq CAP_{r\widetilde{r}}^{0}, \forall r\widetilde{r}. \tag{S4-12}$$

4.3.5 Reserve capacity

Power systems require spare capacity to meet unexpected changes to supply and demand, a requirement that increases with penetration of intermittent VRE. These are typically divided into at least two

types of reserve requirements: capacity planning reserve margins and operational reserves (e.g., Chen 2021, Zhuo 2022). The reserve margin ensures there is sufficient resource adequacy to meet peak load (typically, on an annual basis) plus an additional margin to account for unplanned outages and mis-estimated demand, among other uncertainties. Operational reserve requirements ensure that there is spare capacity of online generators to meet forecast errors in VRE and load. In the RESPO model, owing to its generation layer approach, these reserve constraints must be modified to consider aspects of spare capacity.

First, a load reserve constraint ensures that in each time step of the year, available resources including firm generators, renewable energy up to its hourly availability factor, storage, and transmission can meet demand plus an additional margin (denoted as ρ_0). The load reserve margin ρ_0 is set to be 5% following [88]. Equation S4-13 shows the reserve margin constraint:

$$\begin{aligned}(1+\rho_0)\times\sum_{r\in R_{gr}} DEM_{r,t} \leq \sum_{r\in R_{gr}} [\sum_{l=L_1}^{L_5} af_{r,l,t}\times CAP_{r,l,t} \\ +\sum_v\sum_i x_{r,v,i}\times af_{r,v,i,t}\times CAP_{r,v,i} \\ +\sum_{\widetilde{r}} F_{\widetilde{r}r,t} \\ +\sum_s sto^d_{r,s,t}+sto^{resv}_{r,s,t} \\ -\sum_\lambda trans_{r,\lambda,t}], \forall gr\in GR, t.\end{aligned} \tag{S4-13}$$

Second, a VRE reserve constraint ensures that in each time step of the year, there is sufficient spare capacity of firm generators and storage to meet a shortfall of VRE capacity, equal to total VRE capacity integrated in that hour times a reserve rate (ρ_1). Reserve requirement rates for VRE integration are also set to 5% ($\rho_1 = 0.05$) following [87] and China grid codes. We run a scenario with higher reserve rates ($\rho_0 = \rho_1 = 0.1$) to test the sensitivity of these assumptions. Equation S4-14 shows the VRE integration reserve constraint:

$$\sum_{r\in R_{gr}}(\sum_{l=L_2}^{L_5} resv_{r,l,t}+\sum_s resv_{r,s,t})\geq \rho_1\times\sum_{r\in R_{gr}}\sum_v(inte_{r,v,t}+trans_{r,v,t}), \forall gr\in GR, t. \tag{S4-14}$$

4.3.6 Ramp capacity

We introduce a intermediate variable $p^{out}_{r,l,t}$ to present the total power output of generation layer l in province r and time step t, where $p^{out}_{r,l,t} = load_{r,l,t} + trans_{r,l,t} + \sum_s sto^c_{r,s,l,t}$, for simplicity. And the ramp (up or down) capacity of firm generators in the model is defined by equation S4-15 and S4-16:

$$cap^{ru}_{r,l,t} = \begin{cases} p^{out}_{r,l,t} - p^{out}_{r,l,t-1}, & p^{out}_{r,l,t} > p^{out}_{r,l,t-1} \\ 0, & p^{out}_{r,l,t} \leq p_{r,l,t-1} \end{cases}, \forall t > 1, \tag{S4-15}$$

$$cap^{rd}_{r,l,t} = \begin{cases} p^{out}_{r,l,t-1} - p^{out}_{r,l,t}, & p^{out}_{r,l,t} < p^{out}_{r,l,t-1} \\ 0, & p^{out}_{r,l,t} \geq p^{out}_{r,l,t-1} \end{cases}, \forall t > 1. \tag{S4-16}$$

Changes in output of firm generators incur additional costs due to reduced efficiency and increased wear and tear. Thereby, the ramp capacity per hour needs to be explicitly expressed in the model for all firm generator types in all provinces. The equations above can be reformulated as equations S4-17–S4-20:

$$cap^{ru}_{r,l,t} \geq 0, \forall r, l, t > 1, \tag{S4-17}$$

$$cap^{rd}_{r,l,t} \geq 0, \forall r, l, t > 1, \tag{S4-18}$$

and

$$cap^{ru}_{r,l,t} \geq p^{out}_{r,l,t} - p^{out}_{r,l,t-1}, \forall r, l, t > 1, \tag{S4-19}$$

$$cap^{rd}_{r,l,t} \geq p^{out}_{r,l,t-1} - p^{out}_{r,l,t}, \forall r, l, t > 1. \tag{S4-20}$$

The change in generation between two consecutive time steps is constrained by the hourly ramp rate μ:

$$cap^{ru}_{r,l,t} \leq \mu^{\text{up}}_{l} \times CAP_{r,l,t}, \forall r, l \in \{L2, L5\}, t > 1, \tag{S4-21}$$

$$cap^{rd}_{r,l,t} \leq \mu^{\text{dn}}_{l} \times CAP_{r,l,t}, \forall r, l \in \{L2, L5\}, t > 1. \tag{S4-22}$$

For generation layers L3 and L4, the ramp up/down capacity is constrained by the ramp up/down rate multiplied by $CAP_{r,l,t}$ plus must-run capacity, respectively.

4.3.7 Storage operation

Energy in storage. The energy stored in storage systems at the first time step of the model is set to be equal to the energy in storage at the last time step of the model. We use $sto^{e}_{r,s,0}$ to denote the energy in storage at the first time step:

$$sto^{e}_{r,s,0} = sto^{e}_{r,s,T}, \forall r, s. \tag{S4-23}$$

The energy in storage $sto^{e}_{r,s,t}$ in time step t is a function of the energy in storage in time step $t-1$ and the charging/discharging schedules in time step t. The energy in storage in province r cannot exceed its capacity multiplied by the duration of storage technology ($cap^{sto}_{r,s} \times \Delta_s$). This constraint is represented

by equations S4-24 and S4-25:

$$0 \leq sto^{e}_{r,s,t} \leq cap^{sto}_{r,s} \times \Delta_s, \qquad \forall r, s, t, \qquad \text{(S4-24)}$$

$$sto^{e}_{r,s,t} = (1 - \zeta_s) \times sto^{e}_{r,s,t-1} + \eta^{c}_{s} \times \sum_{\lambda} sto^{c}_{r,s,\lambda,t} \times \Delta_t - \frac{sto^{d}_{r,s,t}}{\eta^{d}_{s}} \times \Delta_t, \qquad \forall r, s, t \geq 1. \qquad \text{(S4-25)}$$

Storage capacity. In 2060, a significant share of reserve capacity needs to be provided by the energy storage system. Therefore, the sum of discharging capacity and reserve capacity (if any) minus charging capacity [2] in each hour should be less than or equal to the capacity of energy storage for all storage types for all provinces:

$$sto^{c}_{r,s,t} \leq cap^{sto}_{r,s}, \qquad \text{(S4-26)}$$

$$sto^{d}_{r,s,t} \leq cap^{sto}_{r,s}, \qquad \text{(S4-27)}$$

$$sto^{d}_{r,s,t} + resv_{r,s,t} - sto^{c}_{r,s,t} \leq cap^{sto}_{r,s}, \forall r, s, t. \qquad \text{(S4-28)}$$

Also, the sum of energy for discharging and reserve (if any) in time step t should be less than or equal to the total energy in storage in the previous time step $t - 1$. Equation S4-29 represents this constraint:

$$sto^{d}_{r,s,t} \times \Delta_t + resv_{r,s,t} \times \Delta_t \leq sto^{e}_{r,s,t-1} \forall r, s, t \geq 1. \qquad \text{(S4-29)}$$

In addition, We set upper and lower bounds for PHS installation in each province because the maximum capacity of PHS is limited by the resource (see Table S16) and the existing PHS capacity sets the lower bound. Equation S4-30 represents this constraint:

$$CAP^{0}_{r,PHS} \leq cap^{sto}_{r,PHS} \leq CAP^{max}_{r,PHS}, \forall r. \qquad \text{(S4-30)}$$

5 Scenario design

In this section, we provide detailed information for scenarios that we use as robustness tests (results shown in Fig. 3).

5.1 Scenario definitions

- VRE
  - "Wind CapEx 0.5X","Wind CapEx 2X", "Solar CapEx 0.5X", "Solar CapEx 2X": The CapEx (including equipment costs and other initial investment costs) and O&M cost of each scenario are multiplied by the stated factor.
  - "VRE Cell Clustered": Each type of VRE technology is clustered as described in section S2.7 and then enters the model optimization.

[2]A specific type of energy storage systems in a province in a time step should either charge or discharge. We allow the storage system to supply reserve capacity when it is charging or discharging not in its full capacity.

- Different land use scenarios are shown in Table S25.

Table S25: VRE land use scenarios.

Wind\Solar	Conservative	Base	Open
Conservative	Wind conservative + solar conservative	Wind conservative + solar base	Wind conservative + solar open
Base case	Wind base + solar conservative	Base case	Wind base + solar open
Open	Wind open + solar conservative	Wind open + solar base	Wind open + solar open

- Storage
  - "Battery CapEx 0.5X", "Battery CapEx 2X": The CapEx of Li-ion battery storage is multiplied by the stated factor.
  - "With CAES", "With VRB", "With CAES and VRB": Only PHS and BAT are included as the storage technologies adopted in the base case. These scenarios include long-duration storage technologies (CAES, VRB, and both) in addition to PHS and BAT.
- Firm generator
  - "Coal CCS 350 GW": The non-CHP coal CCS capacity of each province in 2060 is 10% ($\sim$100 GW in total) of that in 2020, thus we have 350 GW coal CCS in total under this scenario. In our base case, the capacity of non-CHP coal in 2060 is zero, and CHP coal + CCS is about 250 GW. We assume this additional 100 GW coal + CCS has the same cost as the coal CHP + CCS.
  - "Nuclear 150 GW": The nuclear installation capacity of each province is scaled by the factor 150/218 (the base case has a total nuclear installation of 218 GW).
  - "Nuclear Flex 0.5": The must-run ratio of nuclear is 50% of its capacity (85% in the base case).
  - "Gas capacity 0.5X": The natural gas installation capacity of each province is scaled by the stated factor.
  - "Gas ramp 0.5X": The ramp rate of natural gas ccs is 25%/h, compared to 50%/h in the base case.
  - "Neg Emis 400 Mt", "Neg Emis 200 Mt", "Net Zero Emis": The capacity of BECCS are assumed at 91 GW, 50 GW, 23 GW to scale the total negative emissions of the system to 400 Mt, 200 Mt, and 0, respectively.
- Transmission
  - "UHV CapEx 2X", "UHV CapEx 0.5X": Costs shown in Table S14 for inter-provincial transmission lines and intra-provincial transmission lines (spur lines and trunk lines) are multiplied by the stated factor.

- "Unconstrained UHV Expansion": Interprovincial transmission lines can be constructed or strengthed between any two provinces, with the exception of Tibet and Hainan which are allowed to connect to neighboring provinces only owing to the challenges of estimating costs and building lines over heavily mountainous terrain and over the sea, respectively. In the base case, we only consider strengthening the capacity between provinces already connected by interprovincial lines. A new pre-optimization algorithm is applied to preliminarily determine the voltage and AC/DC selection for the lines, with specific details provided in Section S3.4.2.

- Demand
  - "Demand 1.2X": The hourly demand of each province is multiplied by 1.2 so that the national total demand reaches 18.5 PWh.
  - "Load reduction": Introduce the load reduction technology to improve the demand flexibility of the system, see section 5.2 for details.
- Reliability
  - "Resv ratio 2X": The reserve rates for demand reserve and VRE integration reserve are set to 0.1 (0.05 in the base case).
- Temporal coverage
  - "Two-year period": We use hourly capacity factors of onshore-wind, offshore-wind, utility-scale solar, and distributed solar in 2015 and 2016 and extend the modeling period to two years.

5.2 Load reduction

In the load reduction scenario, the load reduction option is modeled as a virtual generator with zero capital cost and fixed operation and maintenance costs, and a variable cost is set to be equal to the value of lost load. There is no public information available for the compensation of load reduction in China, and therefore, we use compensation information for demand responses as a reference.

At the time of writing, eleven provinces in China have initiated demand response pilots, though payments for demand reduction differ among provinces [89]. Some provinces create a demand response market to determine the price for load reduction (e.g., Jiangsu [90], Shandong [91], Zhejiang [92]). Anhui province set a pre-fixed unit compensation for load reduction (4–6 yuan/kWh) [93], and this range in Henan province is 3–4.5 yuan/kWh according to the response time length [94]. Tianjin had a relatively lower pre-fixed compensation level (2 yuan/kWh) [95]. Given the demand response is still in pilot in China and its cost could be further reduced, we adopt the compensation level in Tianjin pilot as the value of curtailed load (2.0 yuan/kWh). Similar to [39], we set a constraint that the total amount of curtailed load must be less than or equal to 2.4 hours of average hourly demand (about 10% of average daily demand) in a year.

6 Results and sensitivity analysis

6.1 Annual installation capacity and supply chain capabilities

In this study, we find that about 2,000 GW wind (onshore and offshore), 3,900 GW solar (utility-scale and DPV), and 740 GW battery storage are built to achieve a negative carbon emission power system in China by 2060. We translate this capacity installation in 2060 into the annual installation capacity of wind, solar, and battery storage based on two assumptions: 1) the increase in capacity of each technology from 2021 to 2060 is the same; and 2) the installation of wind, solar, and battery storage reaches the targeted capacity until 2060. For wind and solar, we further assume the existing capacity would fully retire in 2030, and the annual retirement rate of installed capacity before 2030 is constant.

By the end of 2020, China has installed 282 GW wind (onshore and offshore), thus the net annual increment of wind from 2021 to 2060 is about 43 GW. By the end of 2020, China has installed 253 GW solar (utility-scale and DPV), thus the net annual increment of solar from 2021 to 2060 is about 91 GW. We assume that the life span of newly build wind and solar is 25 years. Therefore, we can calculate the annual installation of wind and solar based on these assumptions, and the results are shown in Table S26.

Table S26: Annual installation capacity (GW) of wind and solar from 2021 to 2060.

Technology	2021–2030	2031–2045	2046–2055	2056–2060
Wind	71	43	115	86
Solar	116	91	197	192

The installed capacity of battery storage in China before 2021 is negligible, thus the net annual increment of battery storage from 2021 to 2060 is 74 GWh. We assume the life span of newly built battery storage is 15 years (see Table S12). Therefore, we need an annual installation of 74 GWh between 2021 and 2035, 148 GWh between 2036 and 2050, and 222 GWh between 2051 and 2060.

For wind turbines, the total production capacity of China's wind turbine manufacturers in 2021 was estimated to be higher than 200 GW, with the capacity of the top 20 manufacturers exceeding 118 GW [96], surpassing the maximum annual capacity addition estimated by our model ($\sim$120 GW). For solar panels, [97] reports that the total production capacity of China in 2021 was around 359 GW and hired about 100 thousand production staff. The per capita output rate of solar panels in 2021 is about 3.8 MW/yr [98], and this value is predicted to be 5.8 MW/yr in 2030 [98]. Assuming the same employment level and increased per capita output, the production capacity in 2030 will exceed 500 GW, significantly higher than the maximum annual addition ($\sim$200 GW). Considering the critical mineral demand for developing large-scale renewable energy, [99] finds that the minerals of Te, Se, and Dy may face physical shortage based on the present mineral reserve. However, other studies are more optimistic. [100] believes that the supply of critical minerals can support a 100% renewable global energy system. [101] and [102] argue that the effective recycling of critical minerals and technology improvement can relieve the potential material shortage. Some studies also point to unexplored reserves that can be utilized for increasing

demand in the future. For example, [101] suggests that the total resource reserve on the earth should be sufficient for demand, and even the tightest demand for Te could be met via future exploration and exploitation; [103] also points out that the wealth of minerals in the seabed may be an important source of supply. For energy storage batteries, China's production capacity is expected to surpass 750 GWh with a throughput higher than 300 GWh in 2022 [104], which can fully cover the maximum annual addition (222 GWh, or 55.5 GW with maximum discharging hours of four). Note that we do not forecast China's future exports of these products, which would reduce the amount of capacity dedicated to meeting domestic demand.

6.2 VRE land use sensitivity

We conduct a suite of 3×3 scenarios to analyse the potential impact of land use for building wind and utility-scale solar. In this section, we show the installation factor (the wind or solar deployment capacity divided by the maximum installation capacity potential) by province of the base case scenario and different land use scenarios from Fig. S17 to S25.

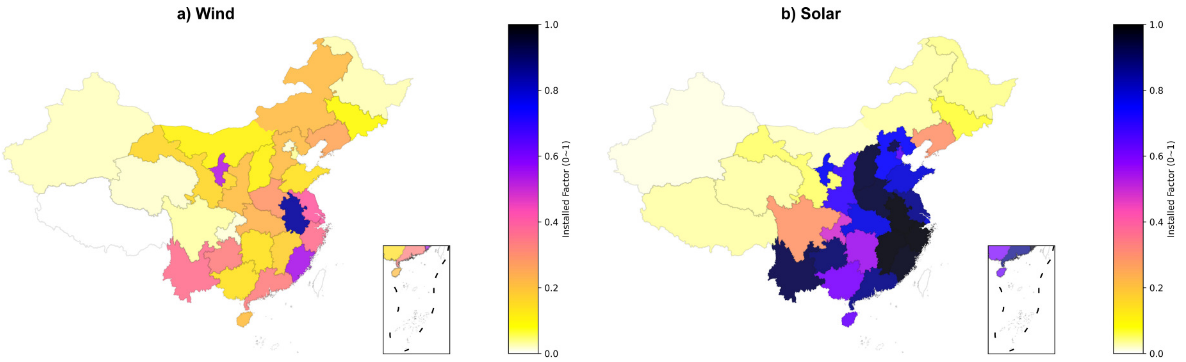

Fig. S17: Provincial installation factor: Base case.

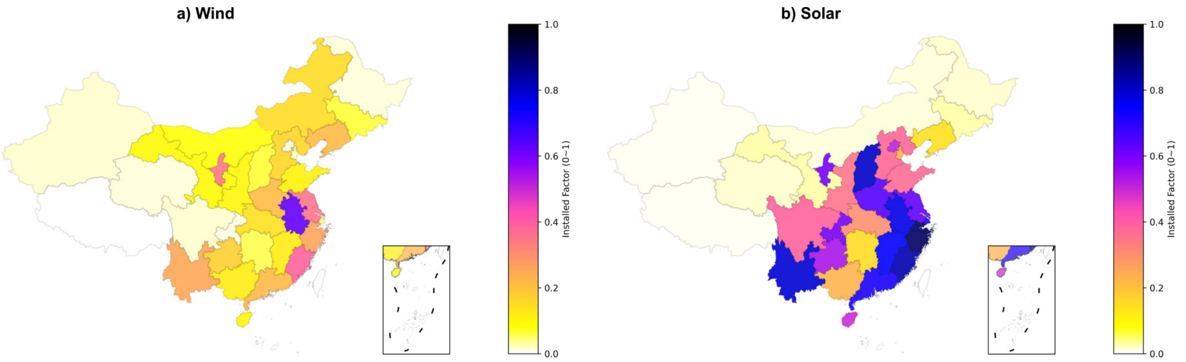

Fig. S18: Provincial installation factor: Wind open + solar open.

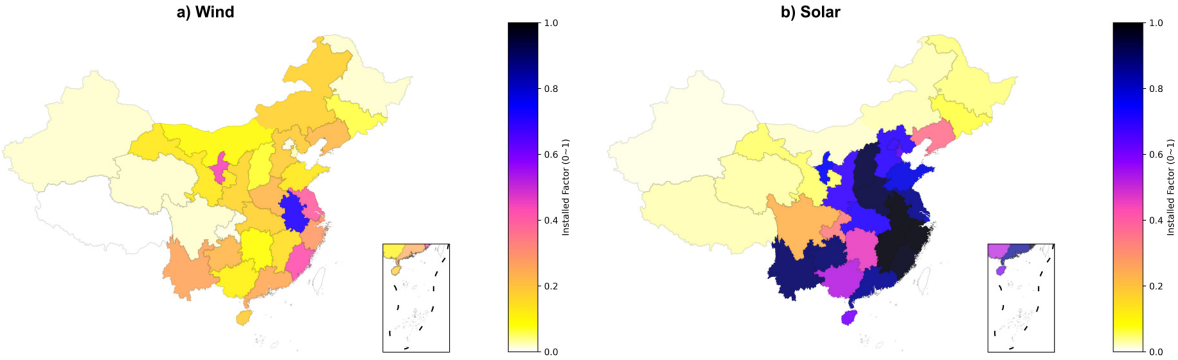

Fig. S19: Provincial installation factor: Wind open + solar base.

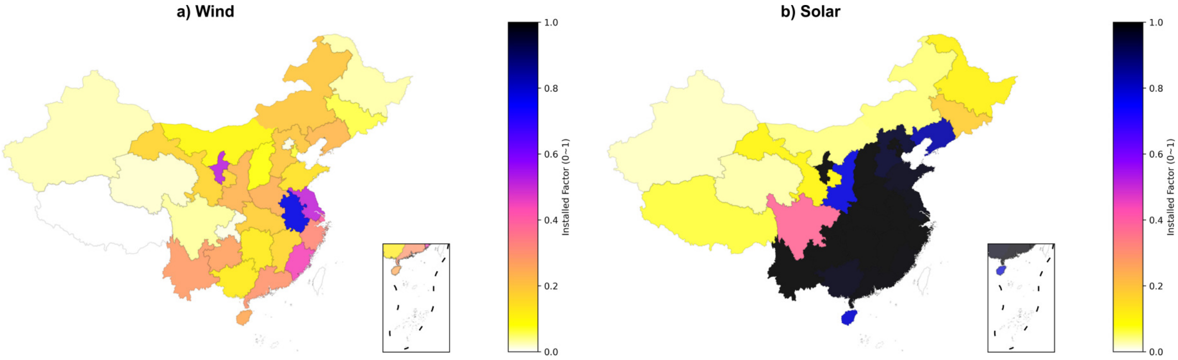

Fig. S20: Provincial installation factor: Wind open + solar conservative.

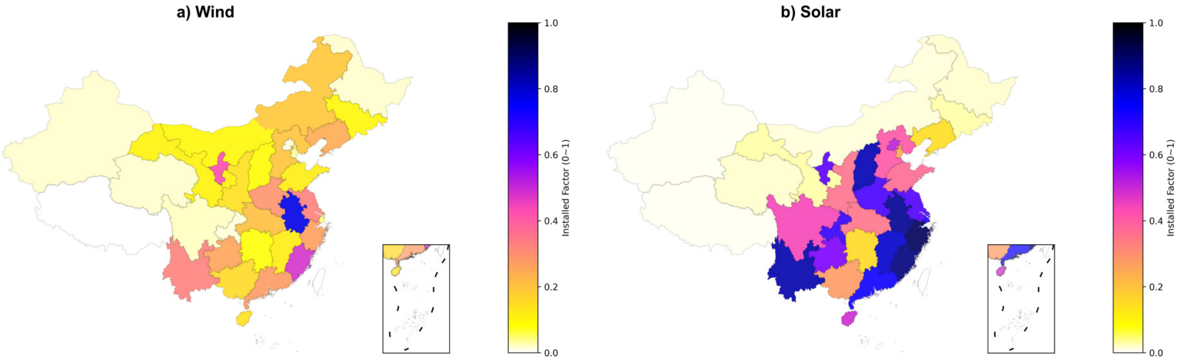

Fig. S21: Provincial installation factor: Wind base + solar open.

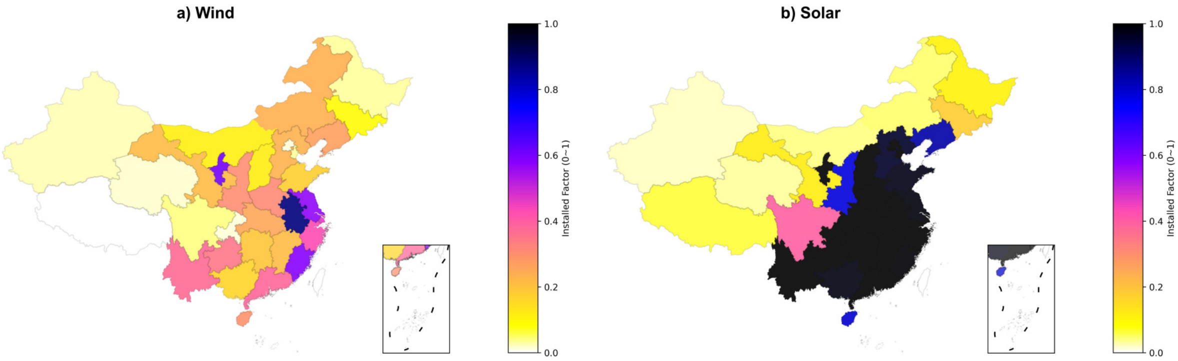

Fig. S22: Provincial installation factor: Wind base + solar conservative.

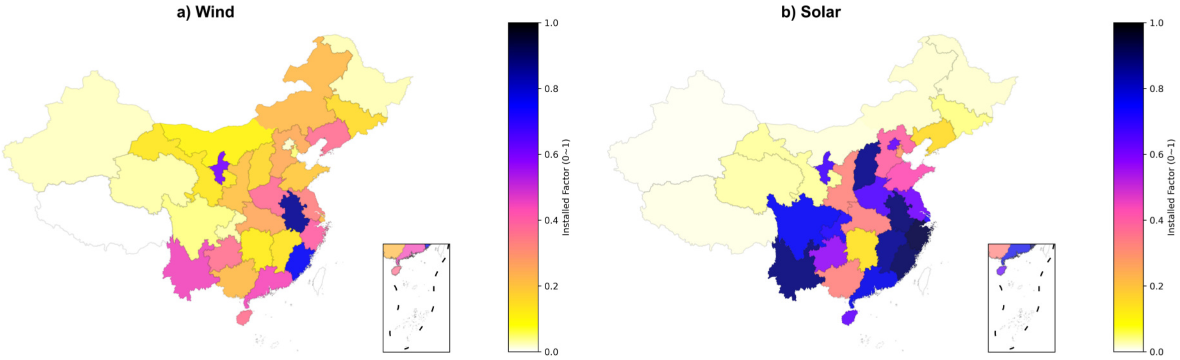

Fig. S23: Provincial installation factor: Wind conservative + solar open.

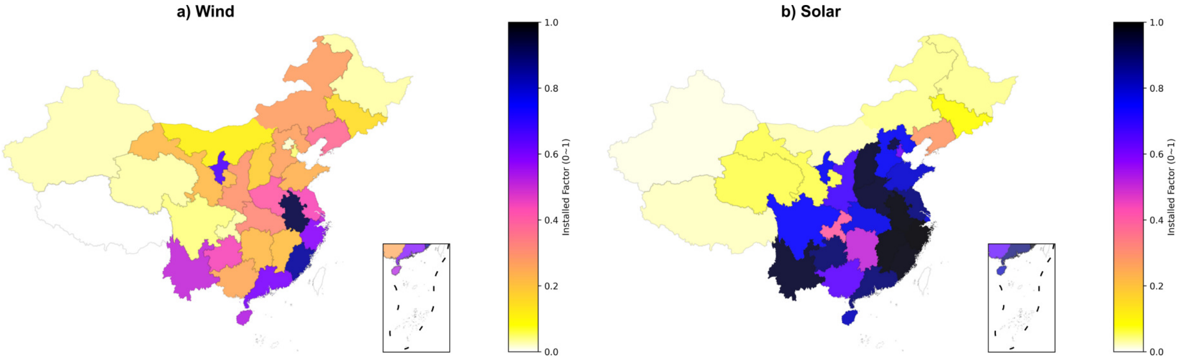

Fig. S24: Provincial installation factor: Wind conservative + solar base.

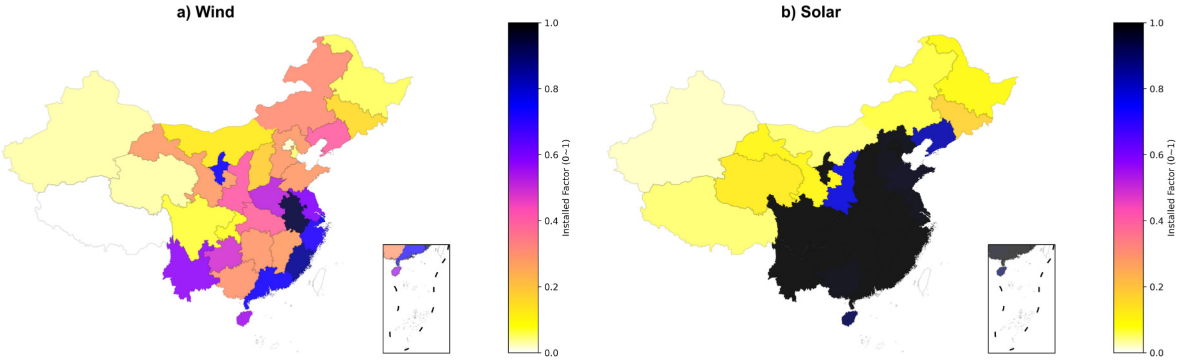

Fig. S25: Provincial installation factor: Wind conservative + solar conservative.

6.3 VRE distribution

Wind and solar deployment maps of the base case and all sensitivities are shown from Fig. S26 to Fig. S59.

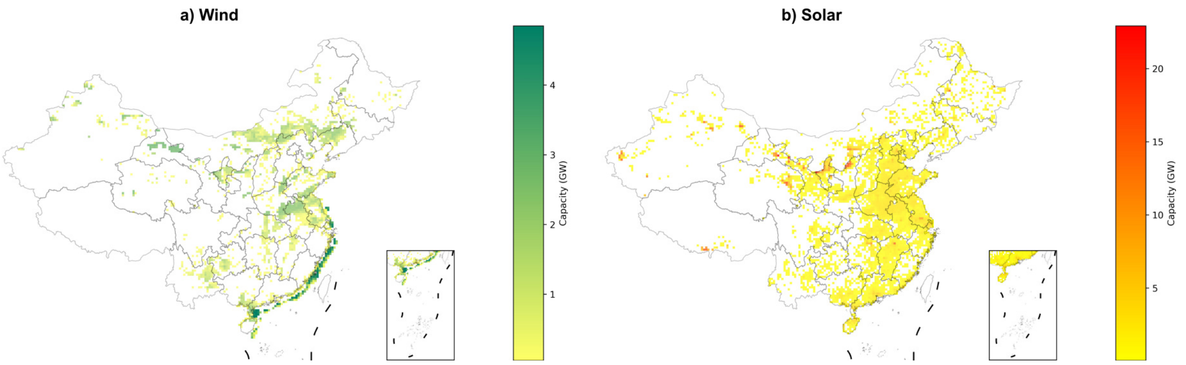

Fig. S26: VRE distribution: Base.

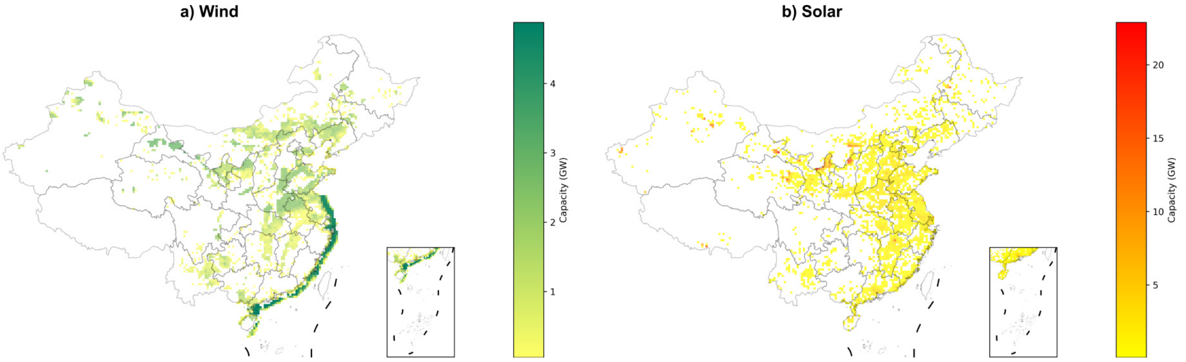

Fig. S27: VRE distribution: Wind CapEx 0.5X.

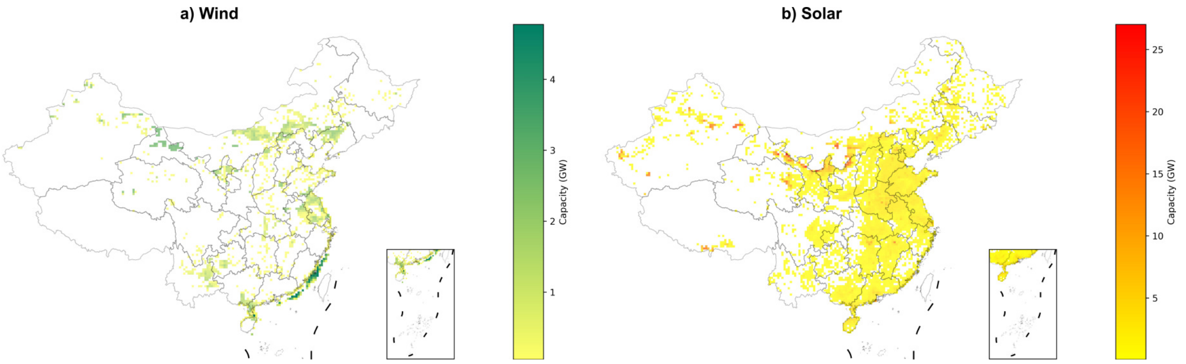

Fig. S28: VRE distribution: Wind CapEx 2X.

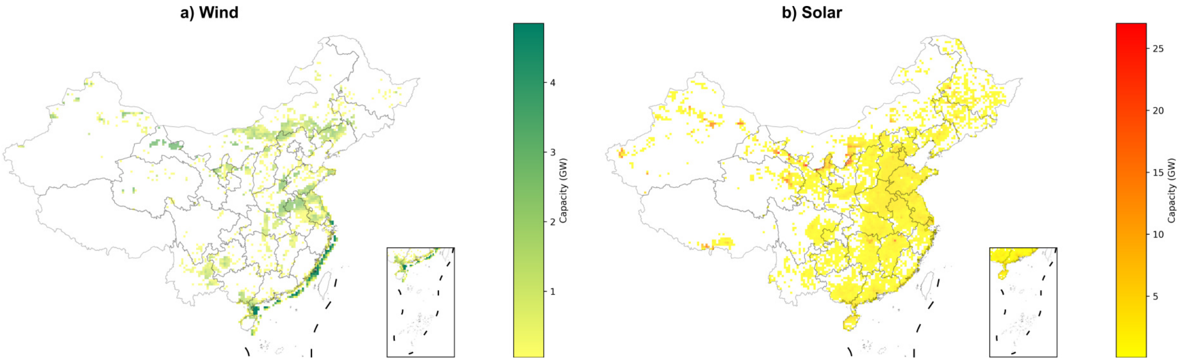

Fig. S29: VRE distribution: Solar CapEx 0.5X.

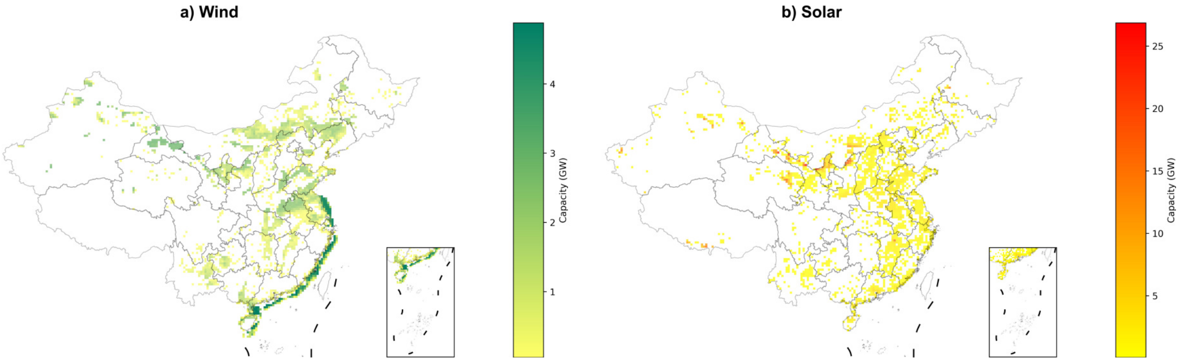

Fig. S30: VRE distribution: Solar CapEx 2X.

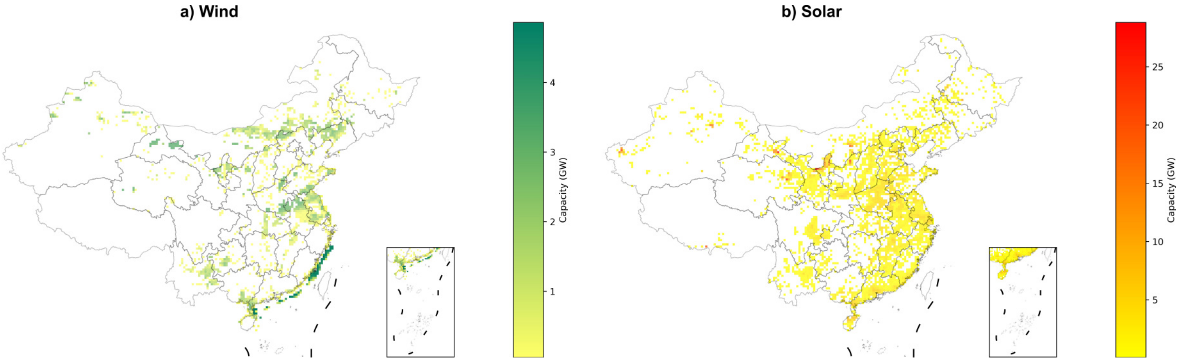

Fig. S31: VRE distribution: Wind open + solar open.

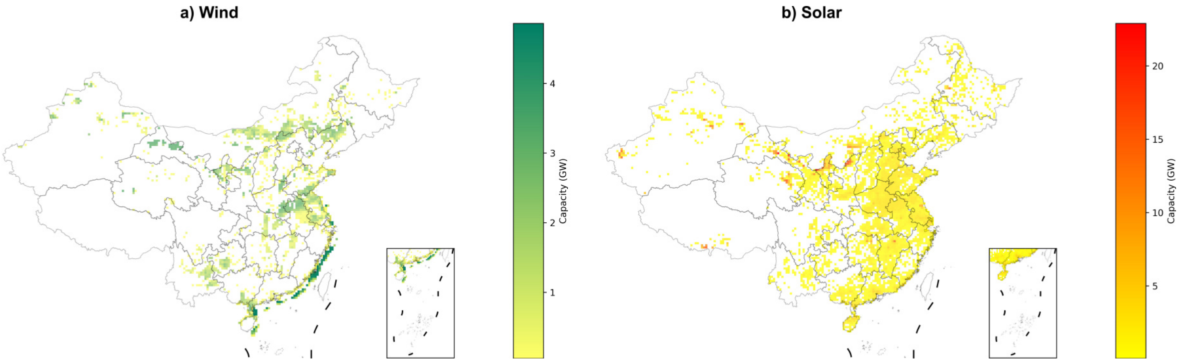

Fig. S32: VRE distribution: Wind open + solar base.

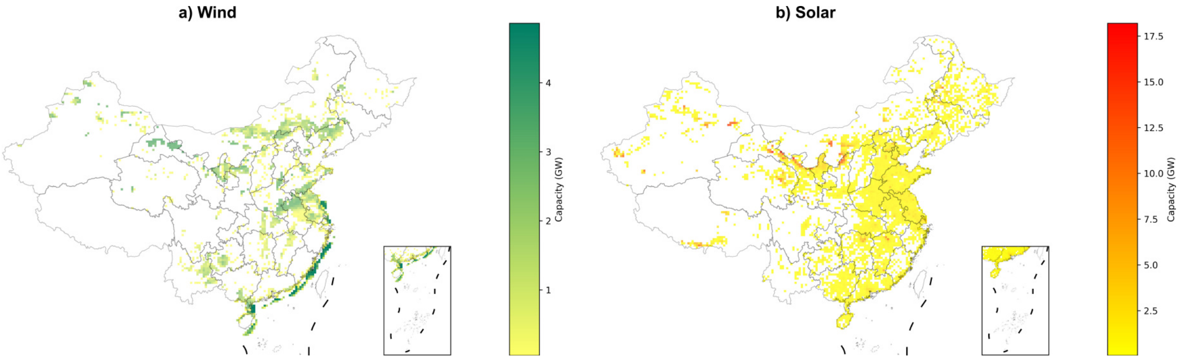

Fig. S33: VRE distribution: Wind open + solar conservative.

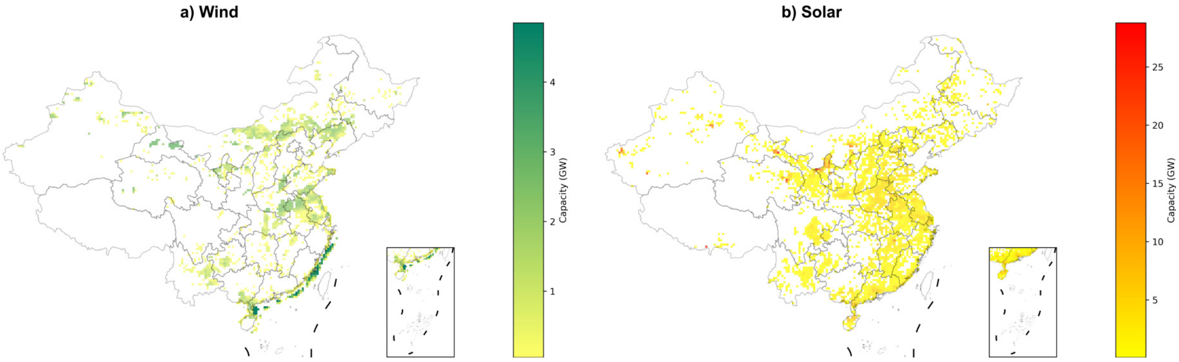

Fig. S34: VRE distribution: Wind base + solar open.

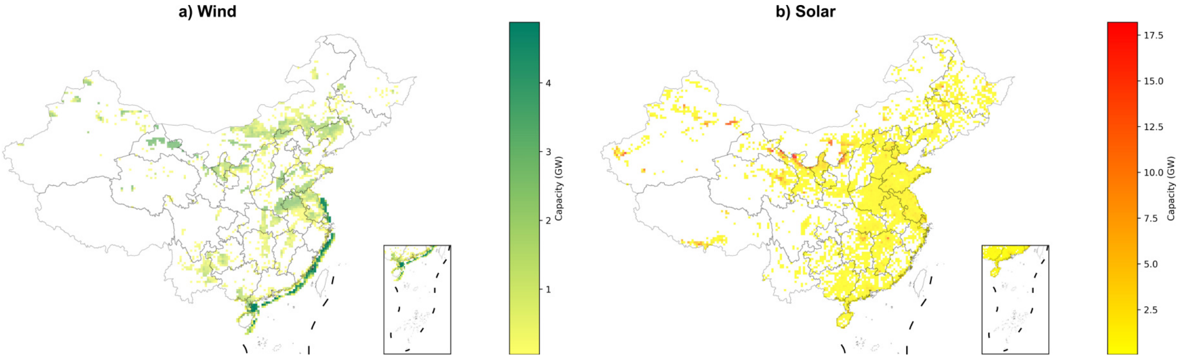

Fig. S35: VRE distribution: Wind base + solar conservative.

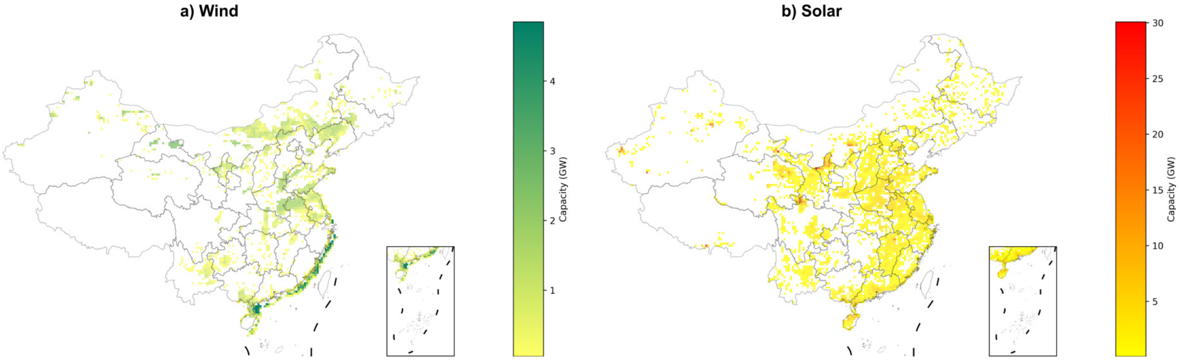

Fig. S36: VRE distribution: Wind conservative + solar open.

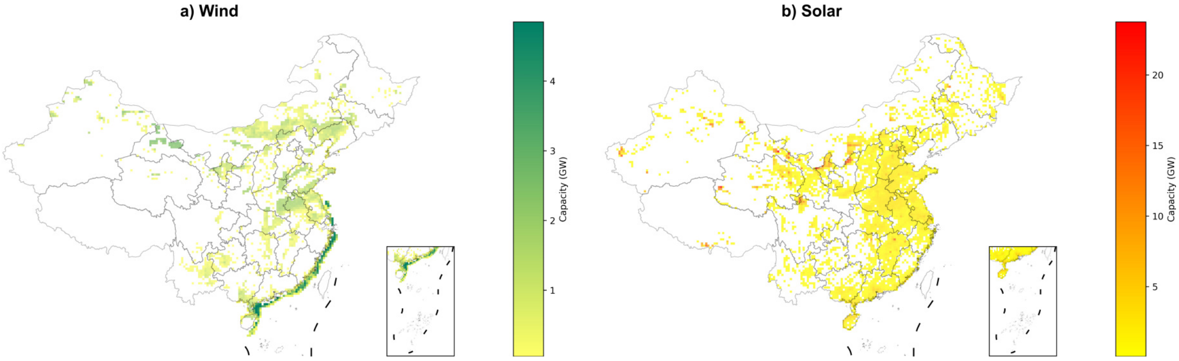

Fig. S37: VRE distribution: Wind conservative + solar base.

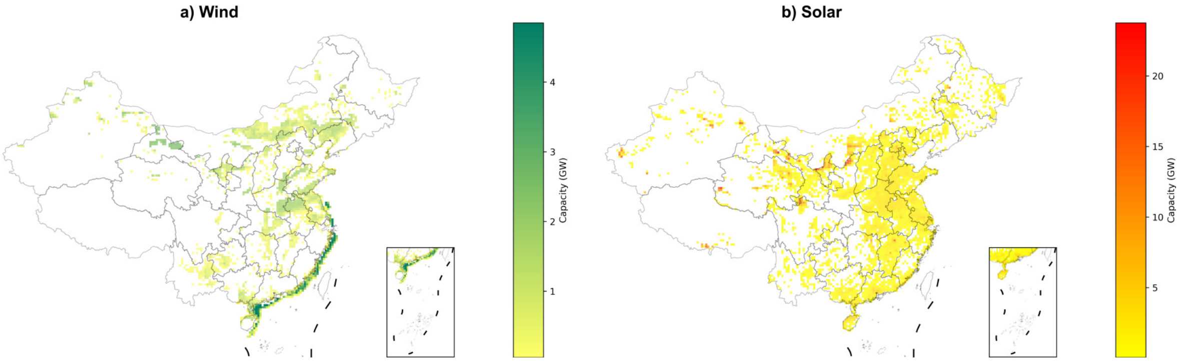

Fig. S38: VRE distribution: Wind conservative + solar conservative.

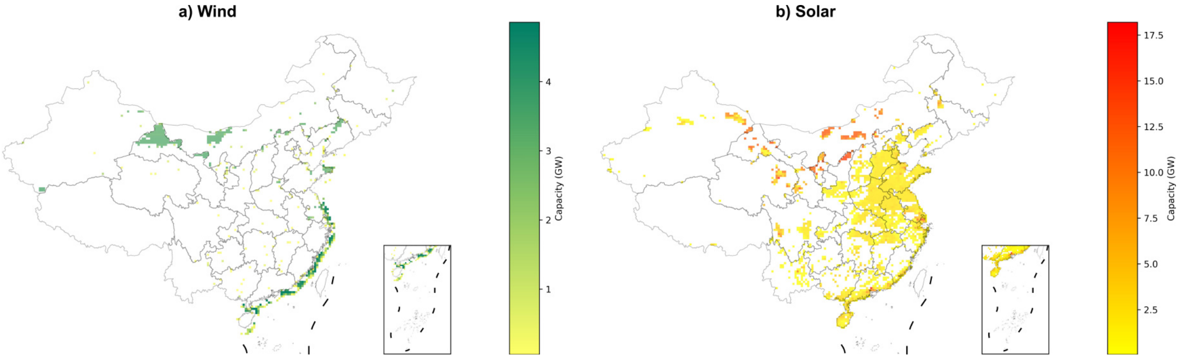

Fig. S39: VRE distribution: VRE Cell Clustered.

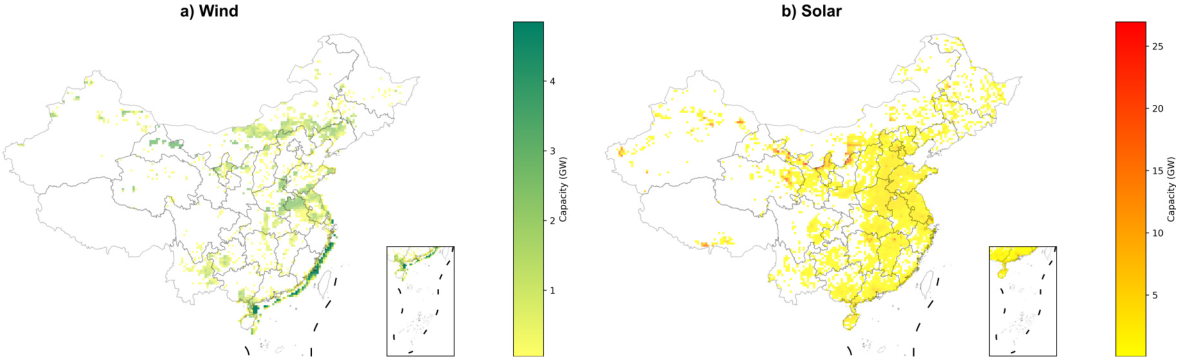

Fig. S40: VRE distribution: Battery CapEx 0.5X.

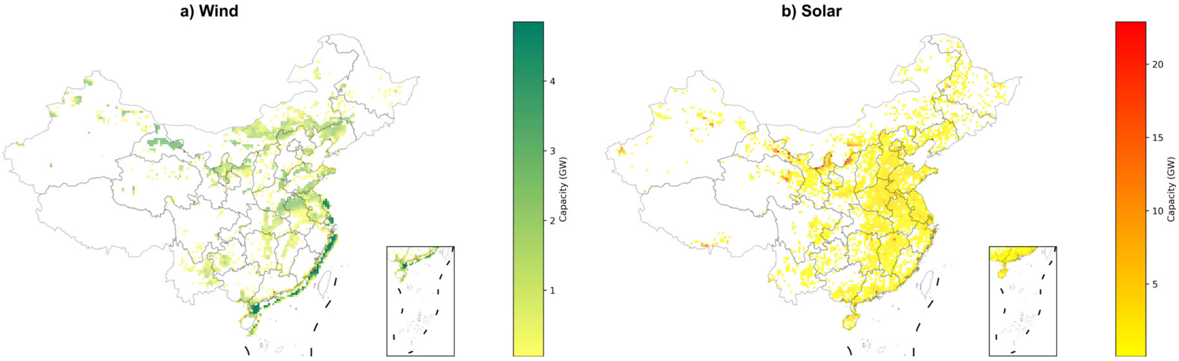

Fig. S41: VRE distribution: Battery CapEx 2X.

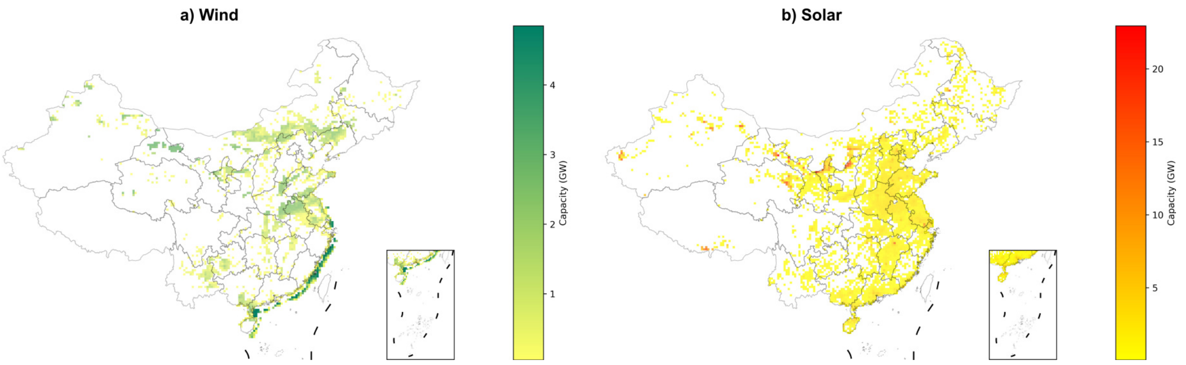

Fig. S42: VRE distribution: With CAES.

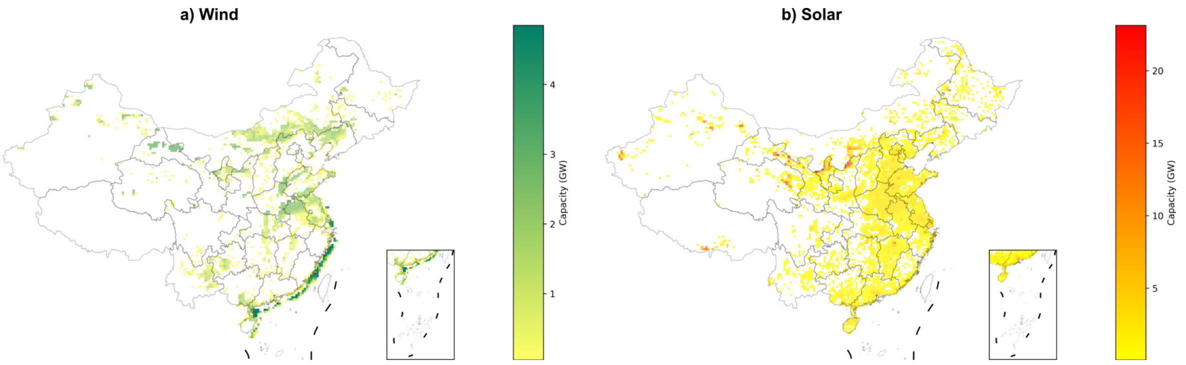

Fig. S43: VRE distribution: With VRB.

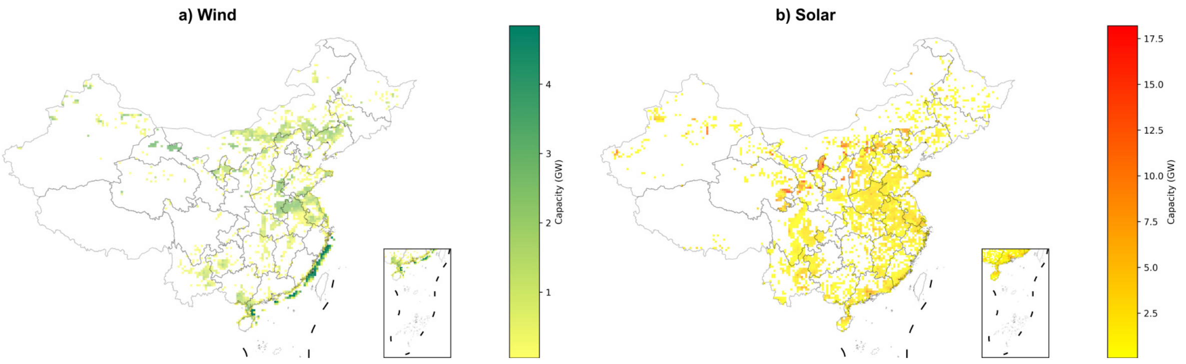

Fig. S44: VRE distribution: With CAES and VRB.

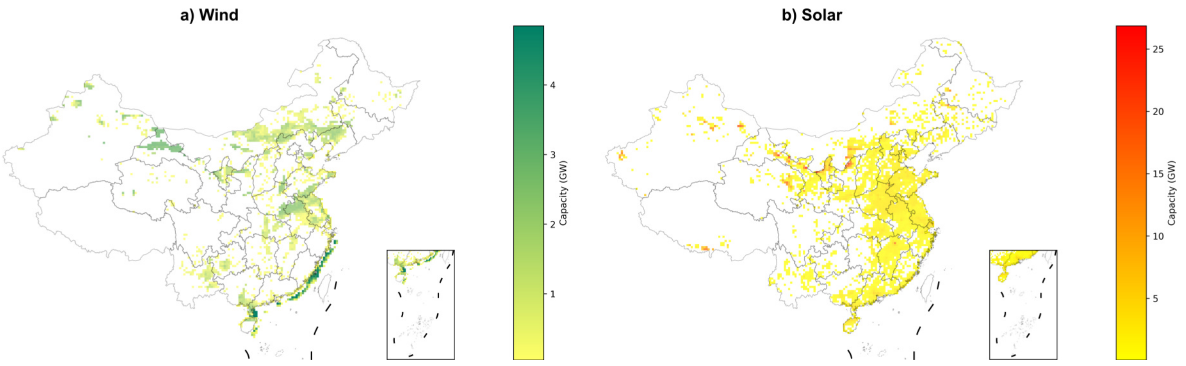

Fig. S45: VRE distribution: UHV CapEx 0.5X.

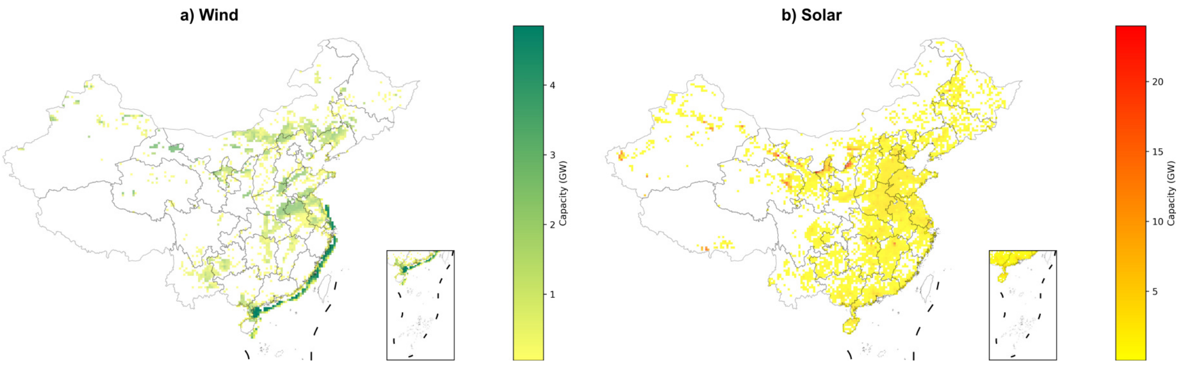

Fig. S46: VRE distribution: UHV CapEx 2X.

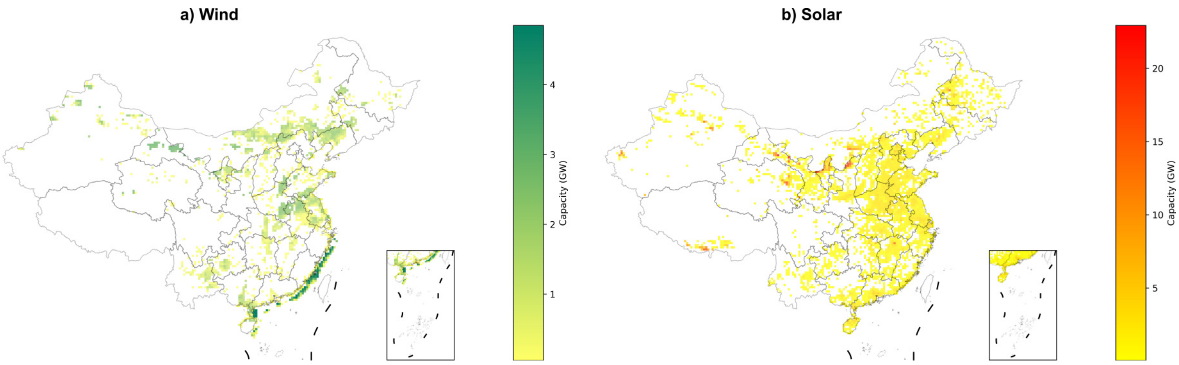

Fig. S47: VRE distribution: Unconstrained UHV Expansion.

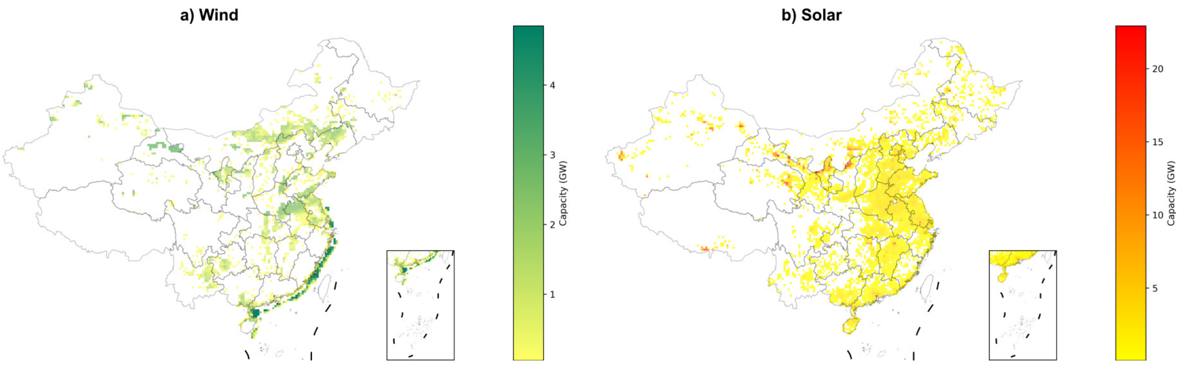

Fig. S48: VRE distribution: Resv Ratio 2X.

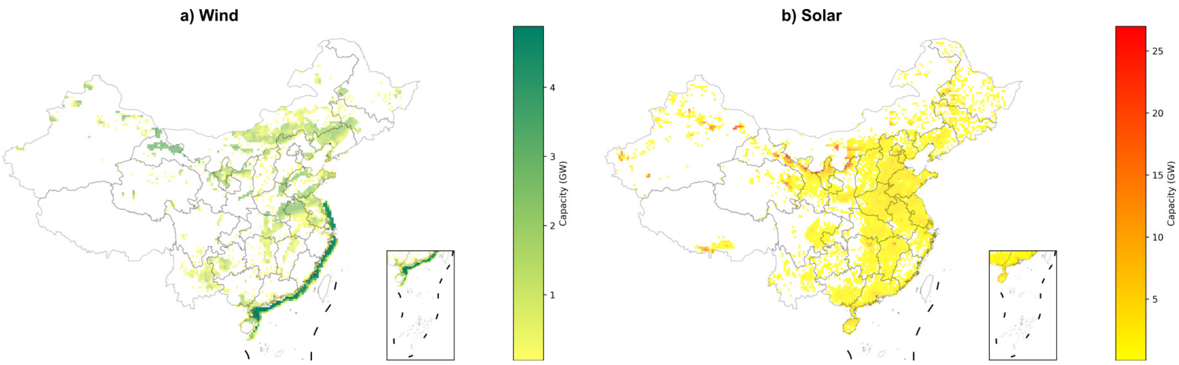

Fig. S49: VRE distribution: Demand 1.2X.

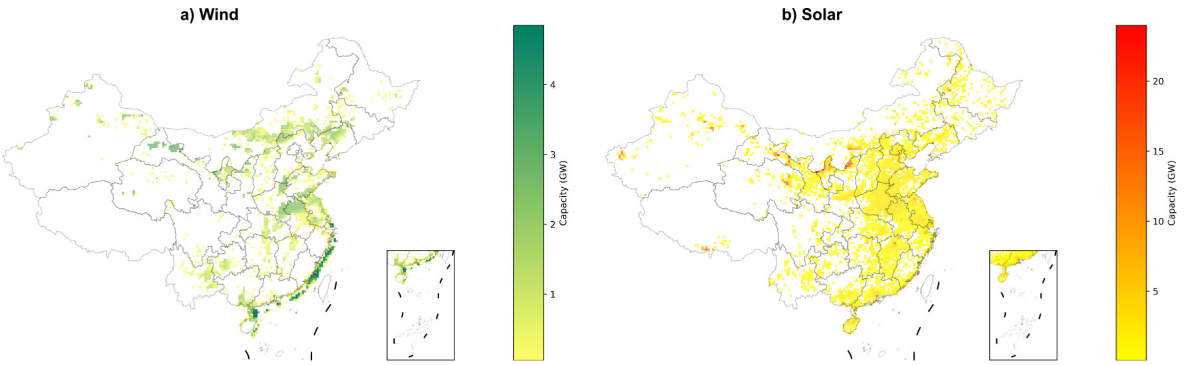

Fig. S50: VRE distribution: Coal CCS 350 GW.

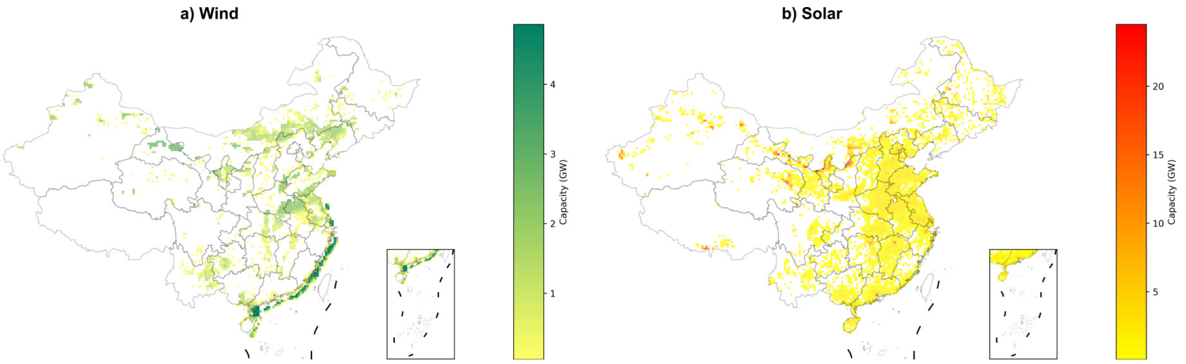

Fig. S51: VRE distribution: Nuclear 150 GW.

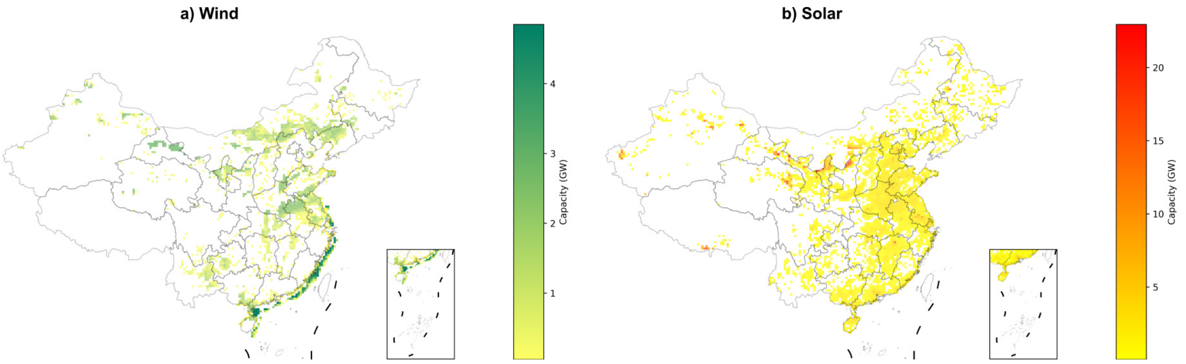

Fig. S52: VRE distribution: Nuclear Flex 0.5.

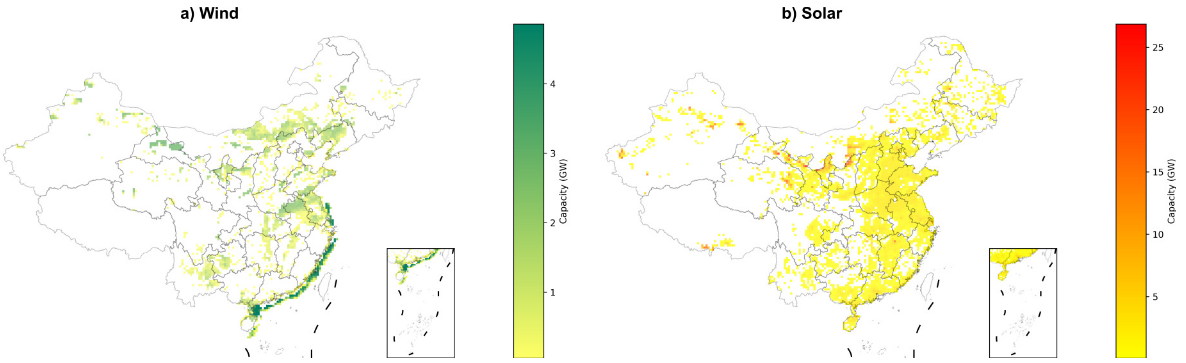

Fig. S53: VRE distribution: Gas Cap 0.5X.

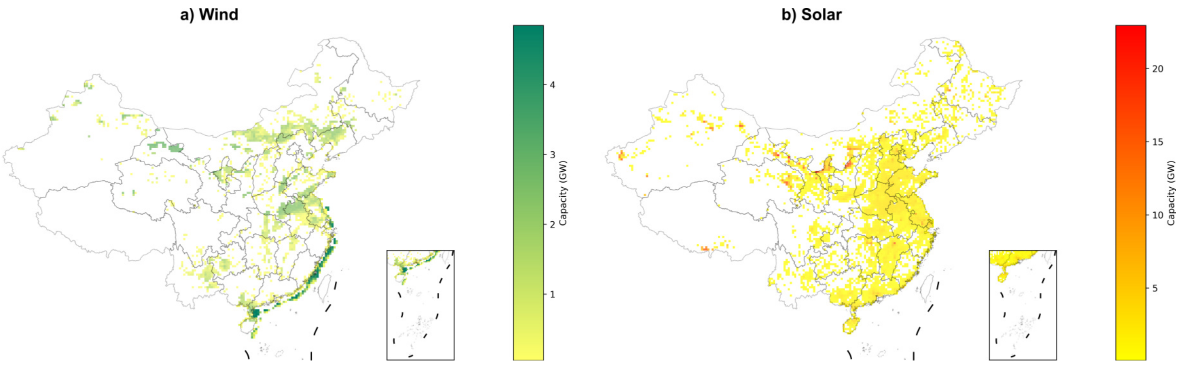

Fig. S54: VRE distribution: Gas ramp 0.25.

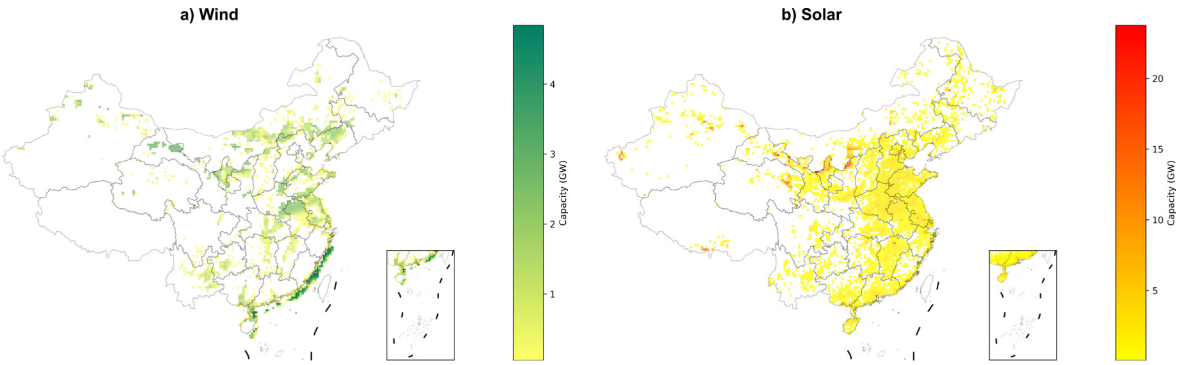

Fig. S55: VRE distribution: load reduction.

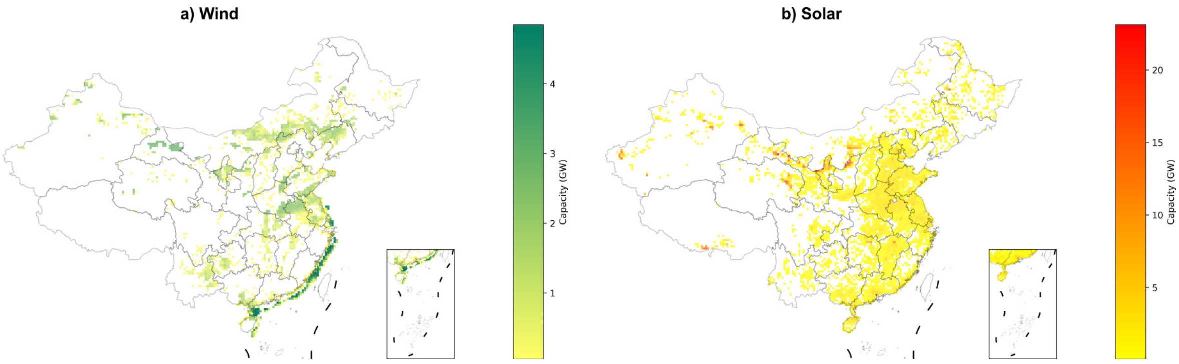

Fig. S56: VRE distribution: Neg Emis 400 Mt.

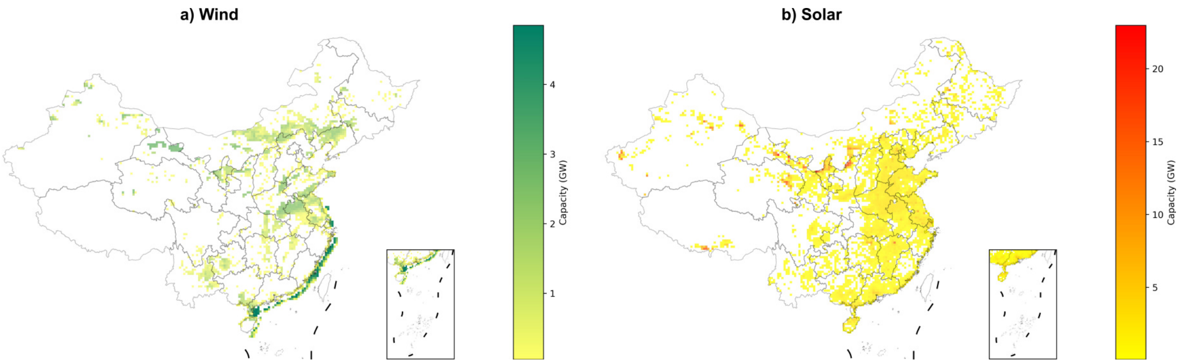

Fig. S57: VRE distribution: Neg Emis 200 Mt.

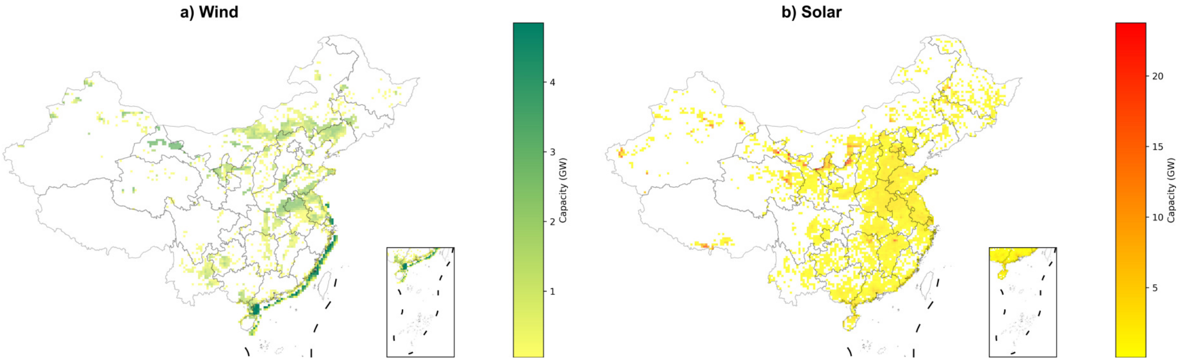

Fig. S58: VRE distribution: Net Zero Emis.

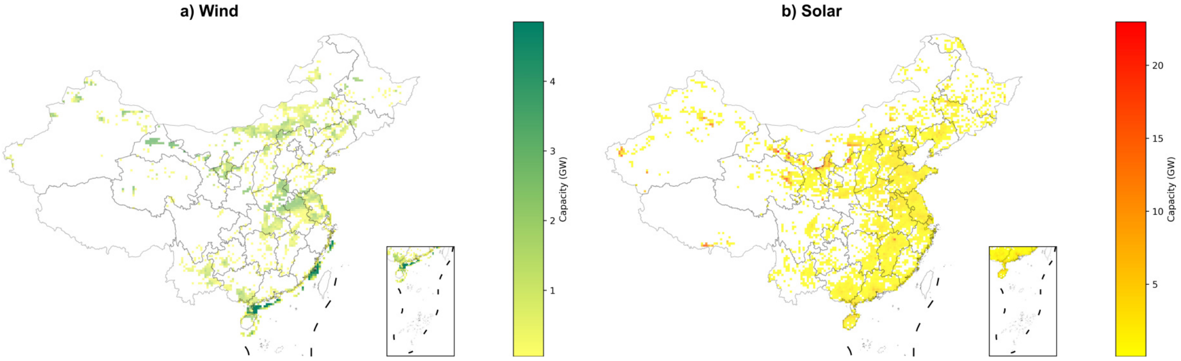

Fig. S59: VRE distribution: Two-year Period.

6.4 Load profile

We show the national load profile of one week in each season[3] from Fig. S60 to S93. As solar can only supply power during the daytime, wind plays an important role in supplying power throughout the whole day. VRE curtailment mainly appears during the daytime with high solar (and coincident wind) power output and a lack of capacity of storage or inter-provincial transmission capacity.

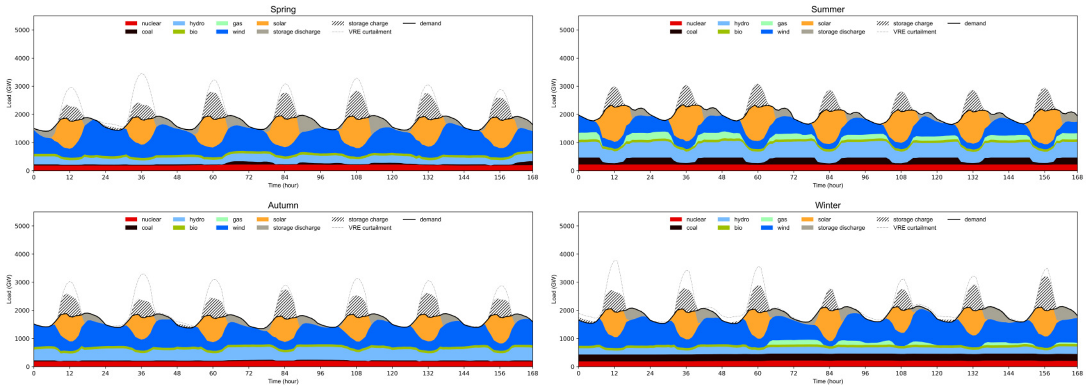

Fig. S60: Load profile: Base.

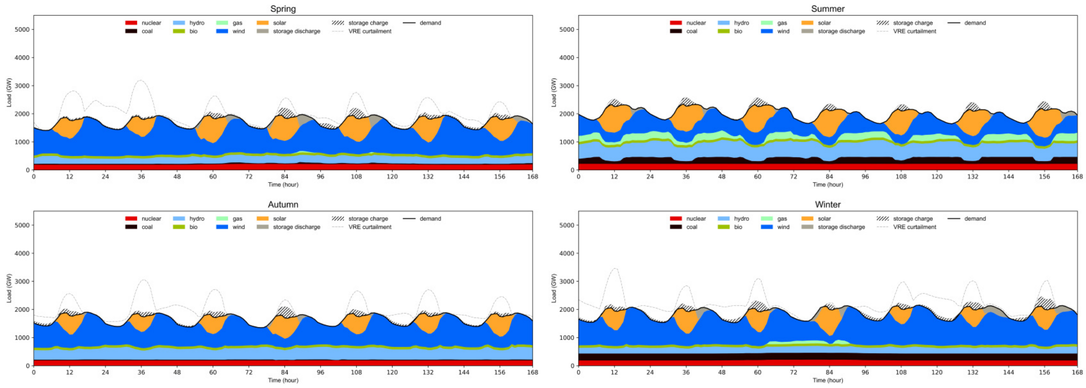

Fig. S61: Load profile: Wind CapEx 0.5X.

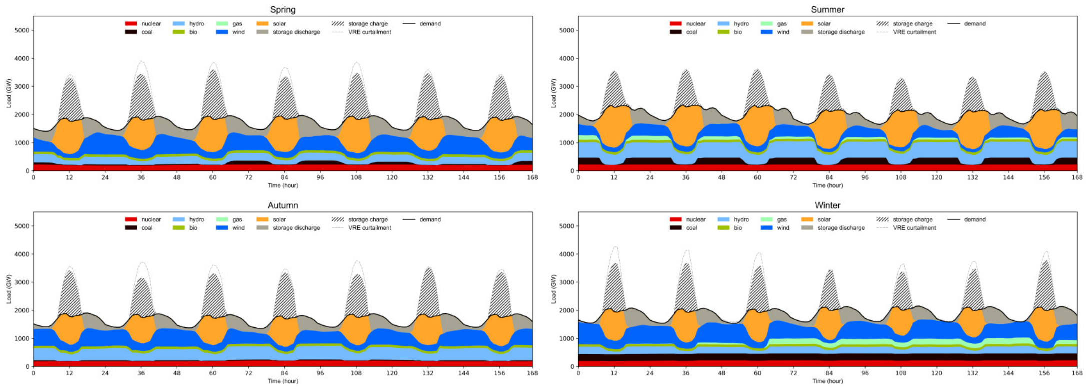

Fig. S62: Load profile: Wind CapEx 2X.

[3] The first week of April for spring, the first week of August for summer, the last week of October for autumn, and the first week of January for winter.

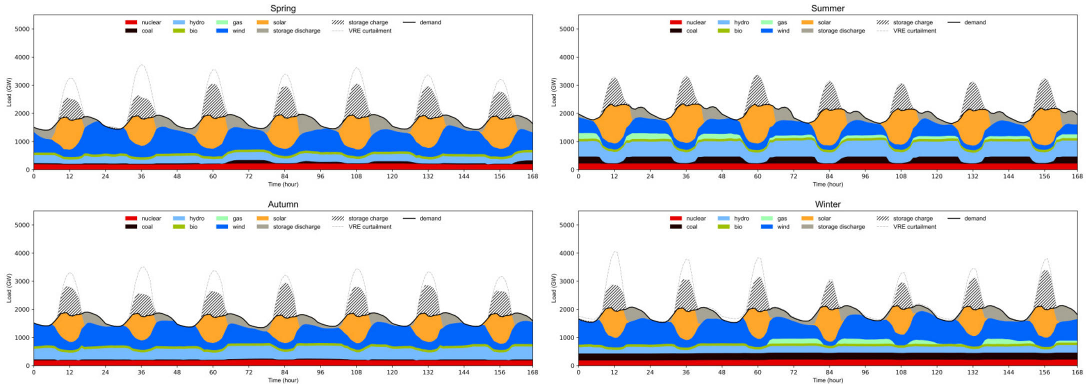

Fig. S63: Load profile: Solar CapEx 0.5X.

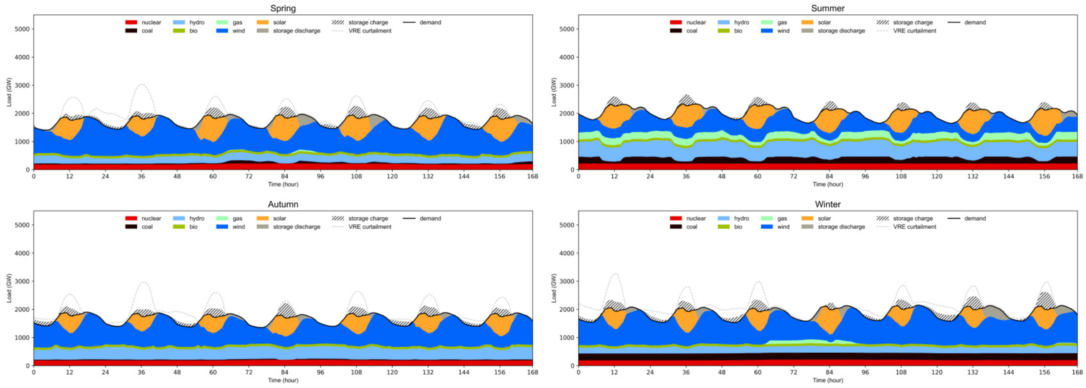

Fig. S64: Load profile: Solar CapEx 2X.

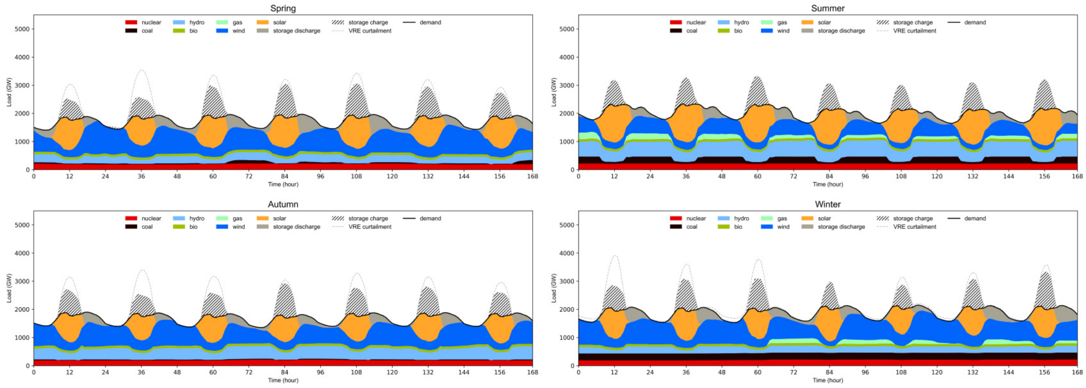

Fig. S65: Load profile: Wind open + solar open.

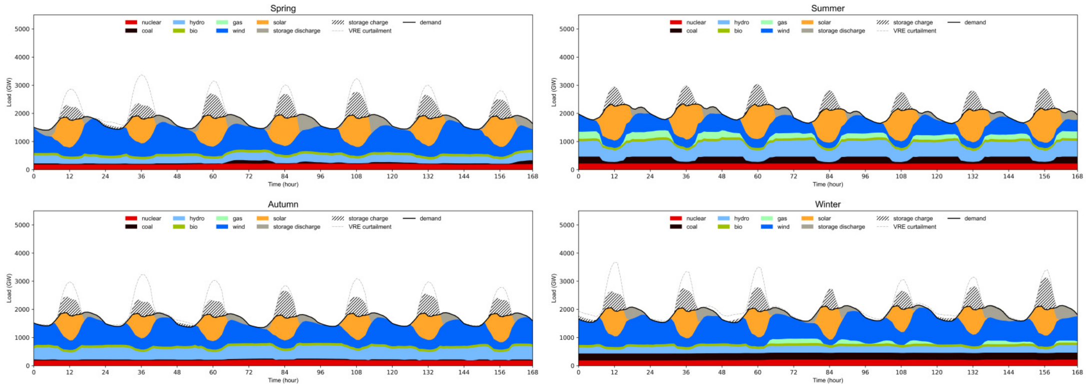

Fig. S66: Load profile: Wind open + solar base.

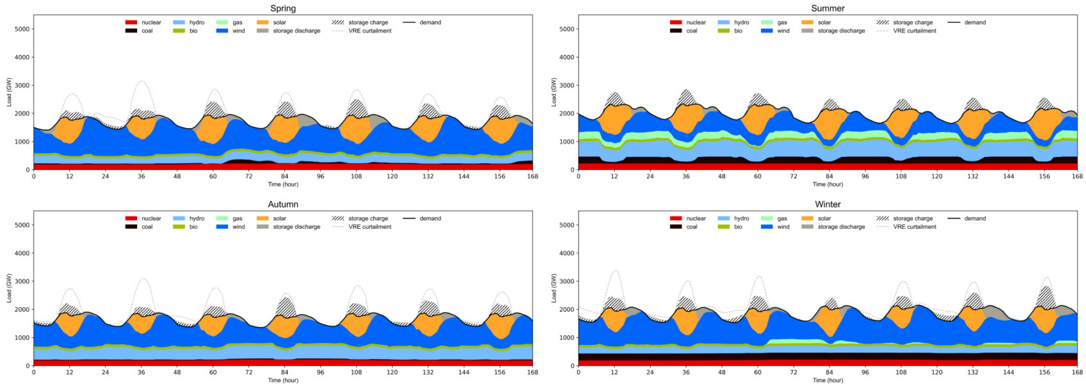

Fig. S67: Load profile: Wind open + solar conservative.

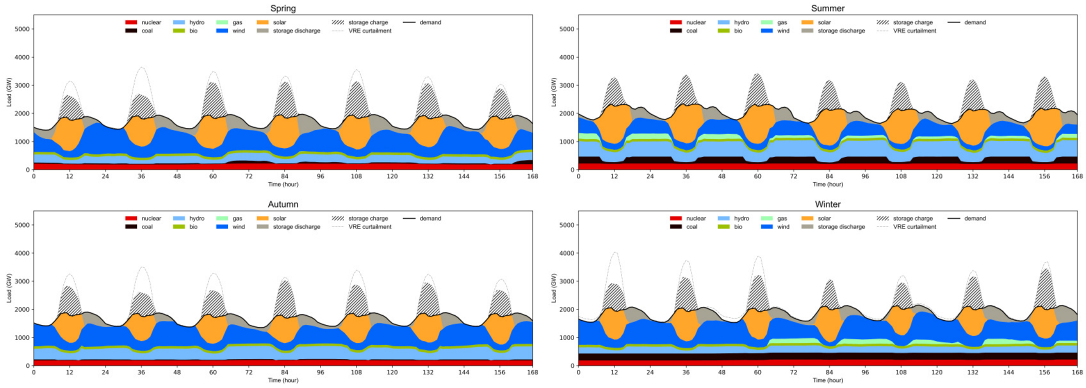

Fig. S68: Load profile: Wind base + solar open.

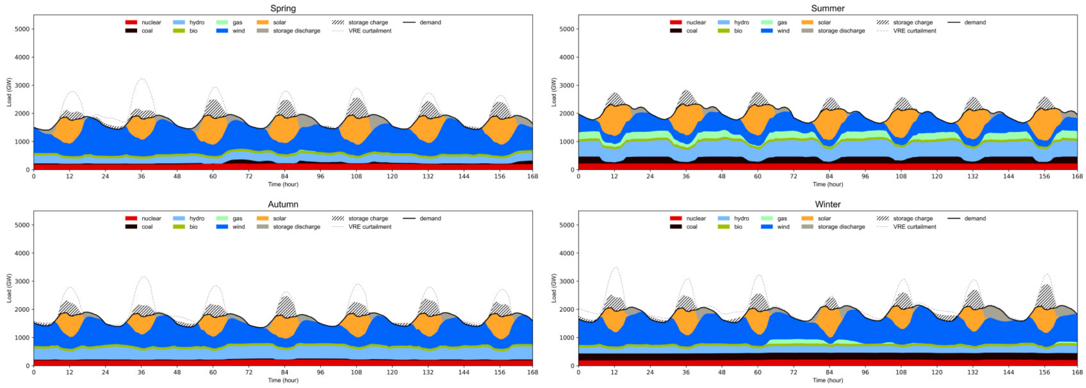

Fig. S69: Load profile: Wind base + solar conservative.

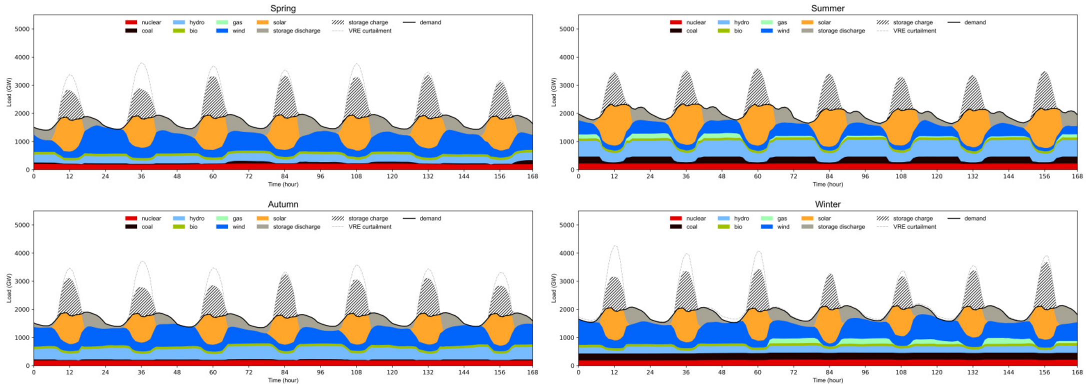

Fig. S70: Load profile: Wind conservative + solar open.

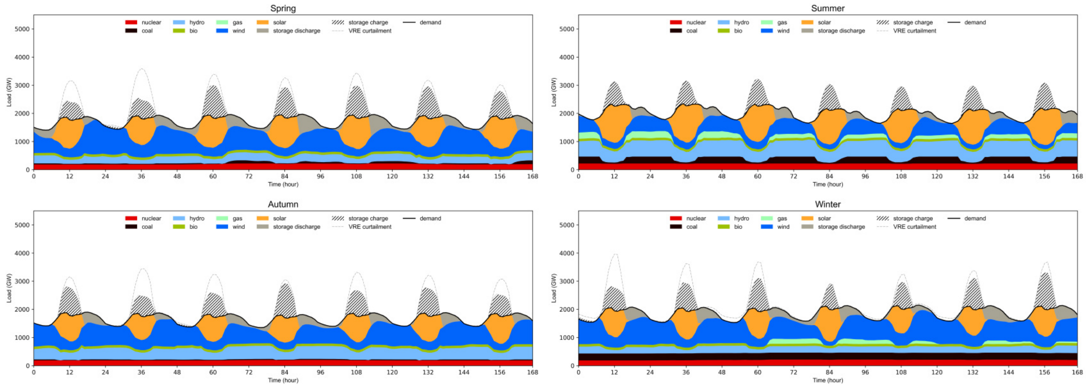

Fig. S71: Load profile: Wind conservative + solar base.

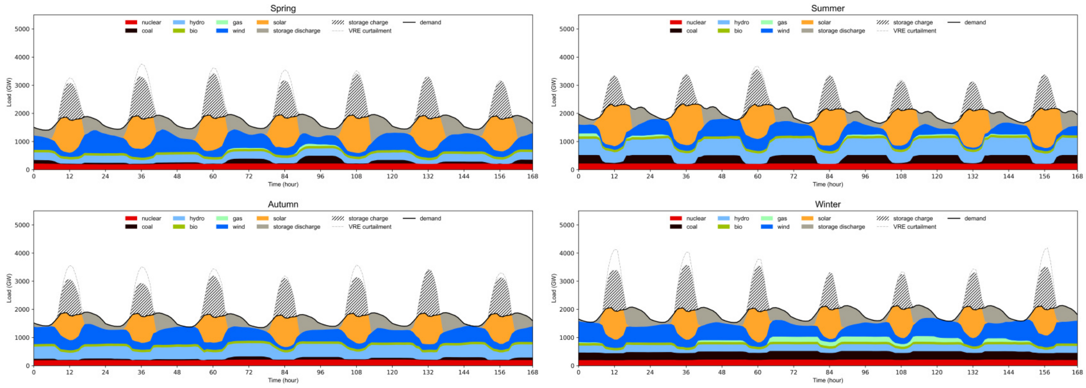

Fig. S72: Load profile: Wind conservative + solar base.

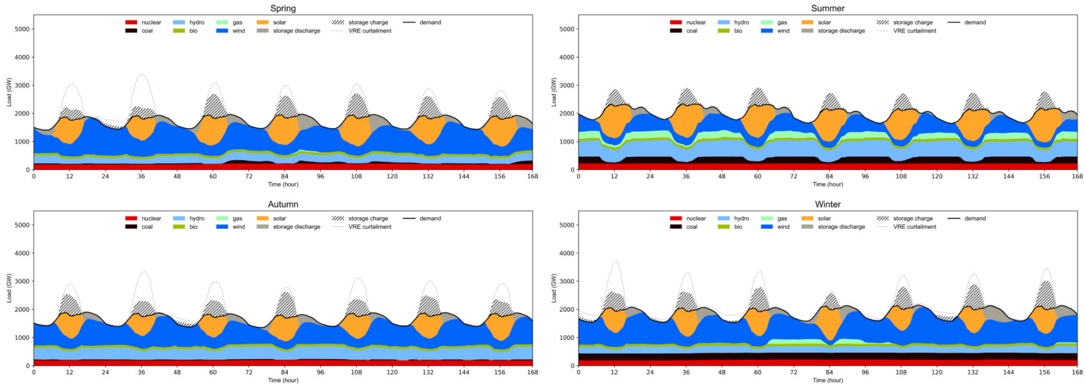

Fig. S73: Load profile: Wind conservative + solar conservative.

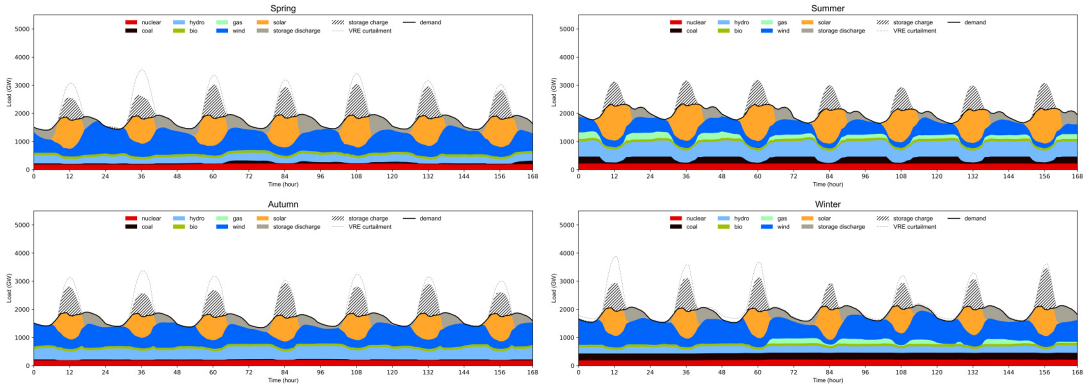

Fig. S74: Load profile: Battery CapEx 0.5X.

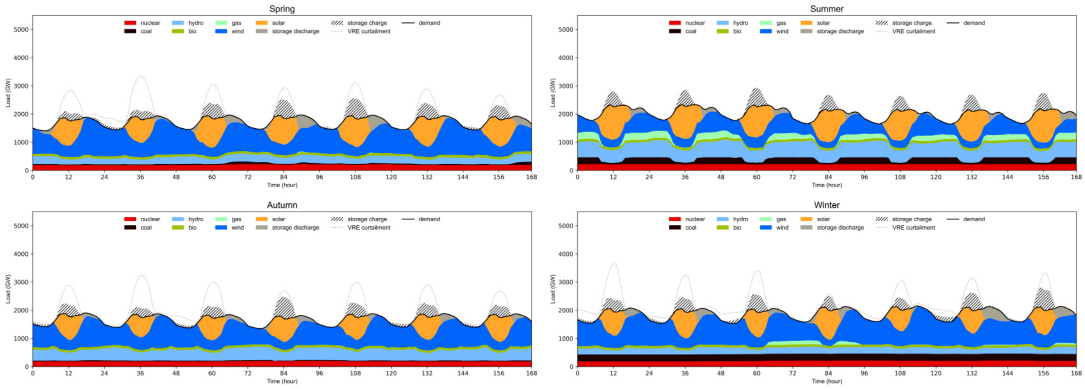

Fig. S75: Load profile: Battery CapEx 2X.

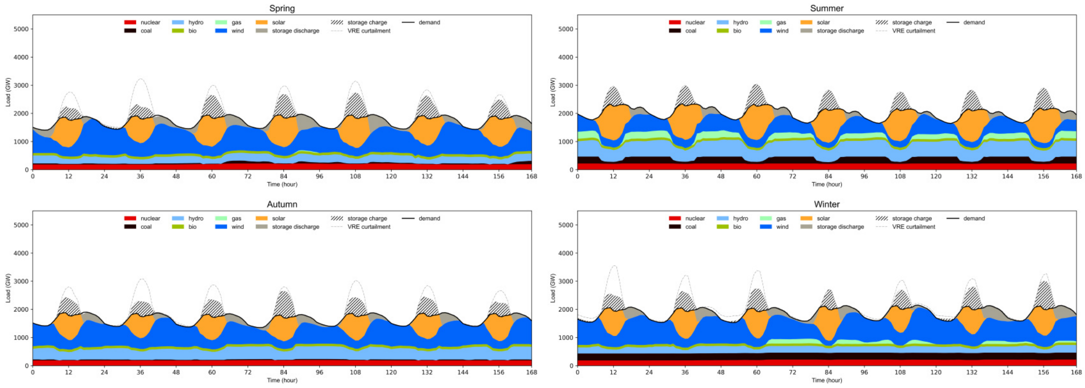

Fig. S76: Load profile: With CAES.

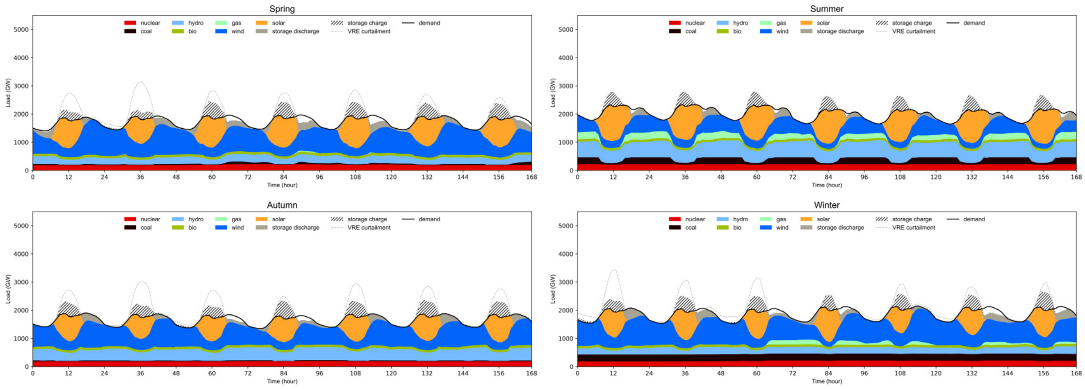

Fig. S77: Load profile: With VRB.

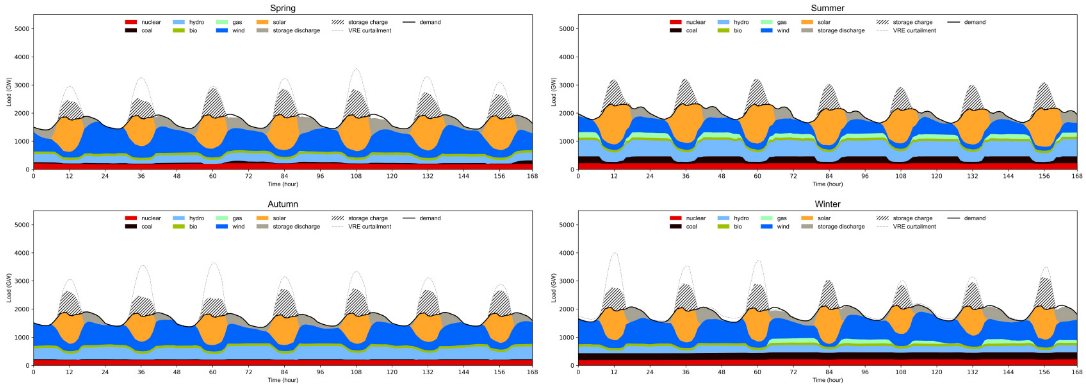

Fig. S78: Load profile: With CAES and VRB.

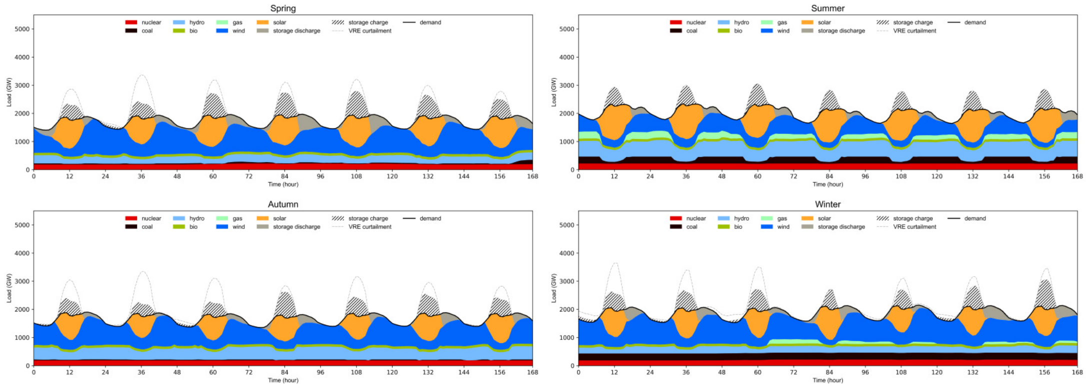

Fig. S79: Load profile: UHV CapEx 0.5X.

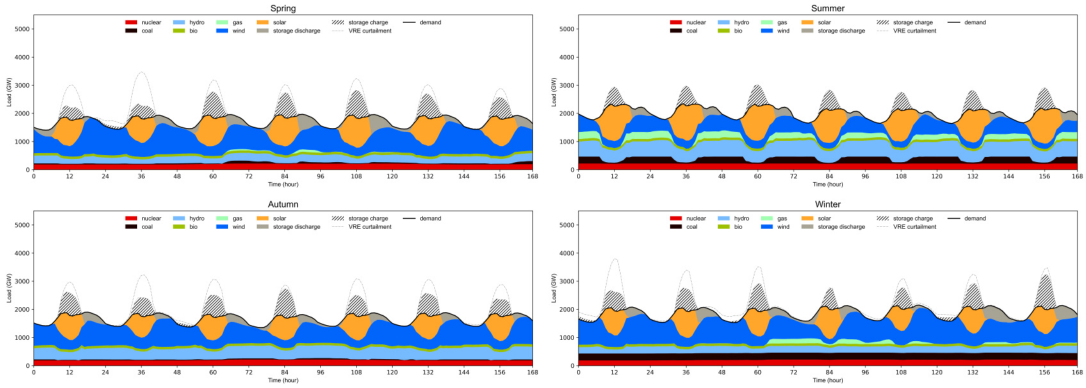

Fig. S80: Load profile: UHV CapEx 2X.

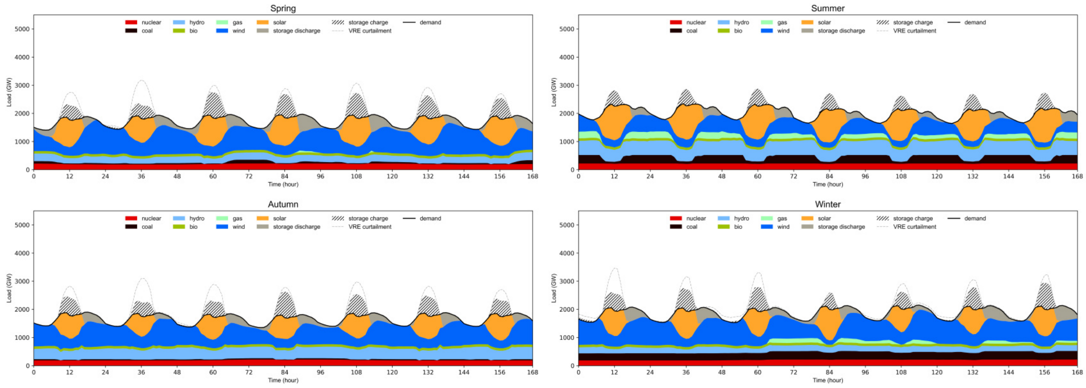

Fig. S81: Load profile: Unconstrained UHV Expansion.

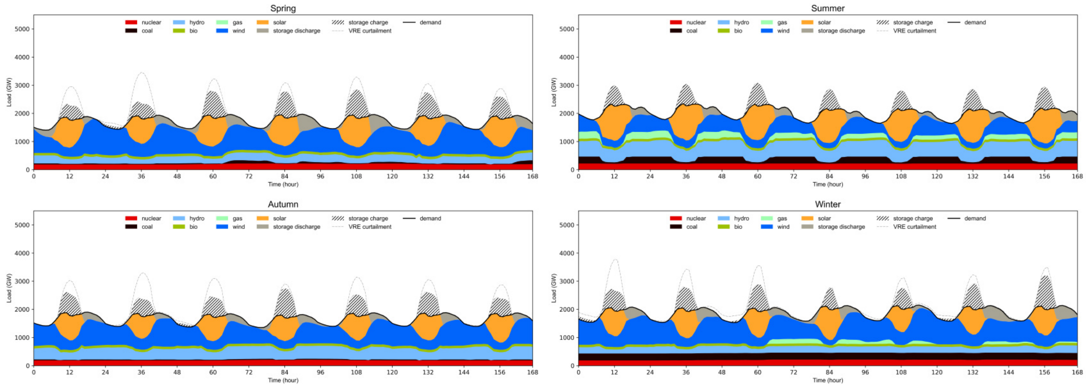

Fig. S82: Load profile: Resv ratio 2X.

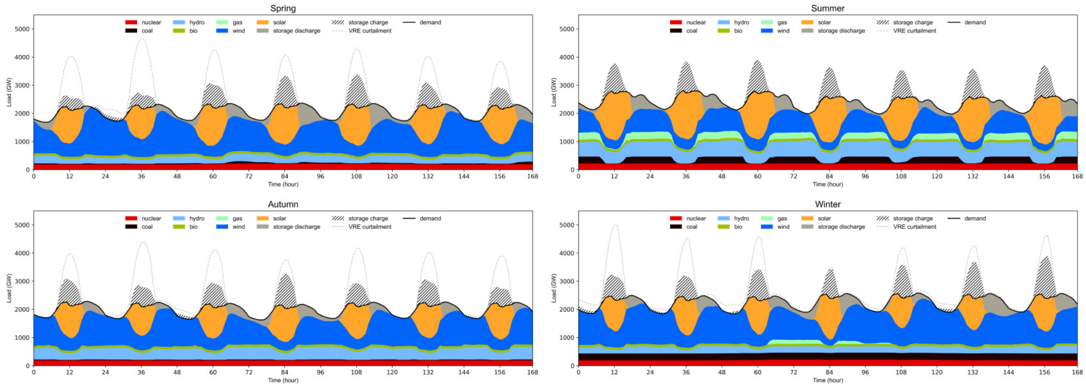

Fig. S83: Load profile: Demand 1.2x.

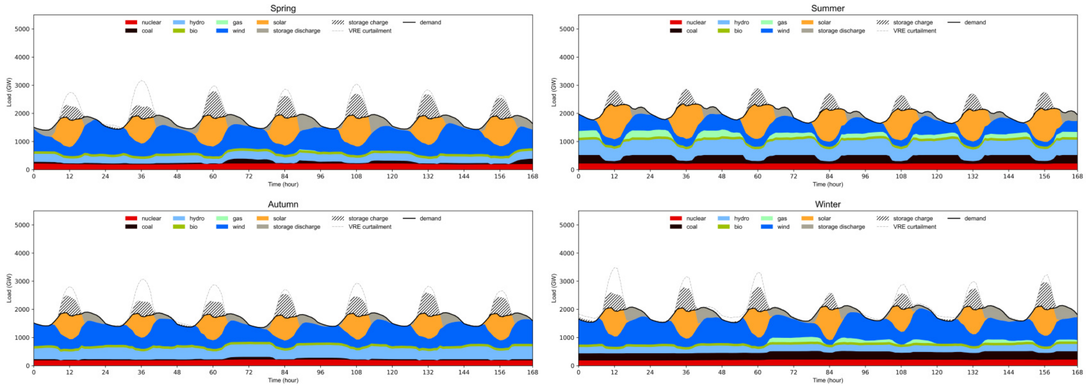

Fig. S84: Load profile: Coal CCS 350 GW.

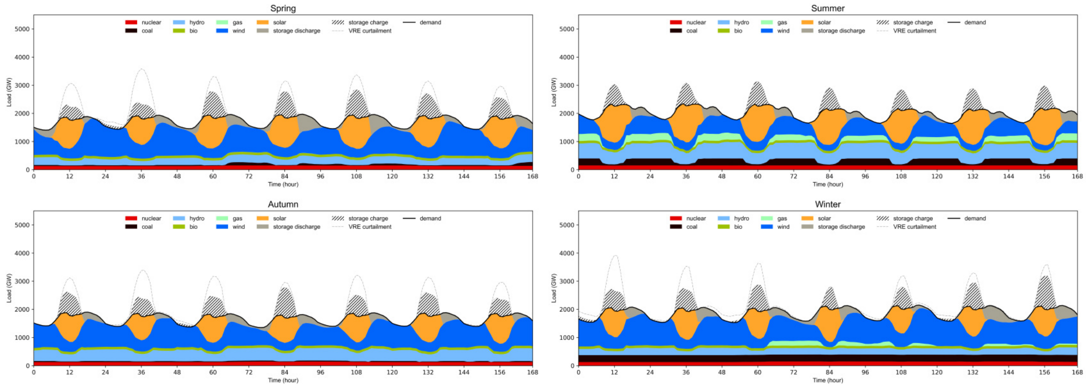

Fig. S85: Load profile: Nuclear 150GW.

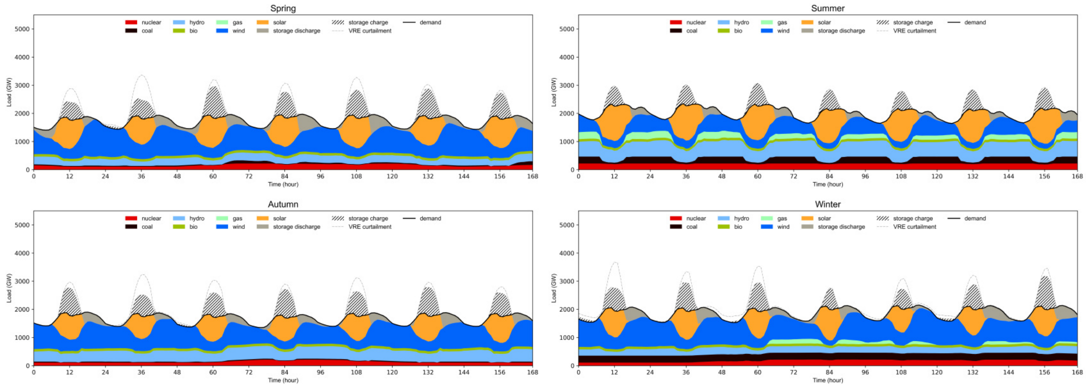

Fig. S86: Load profile: Nuclear Flex 0.5.

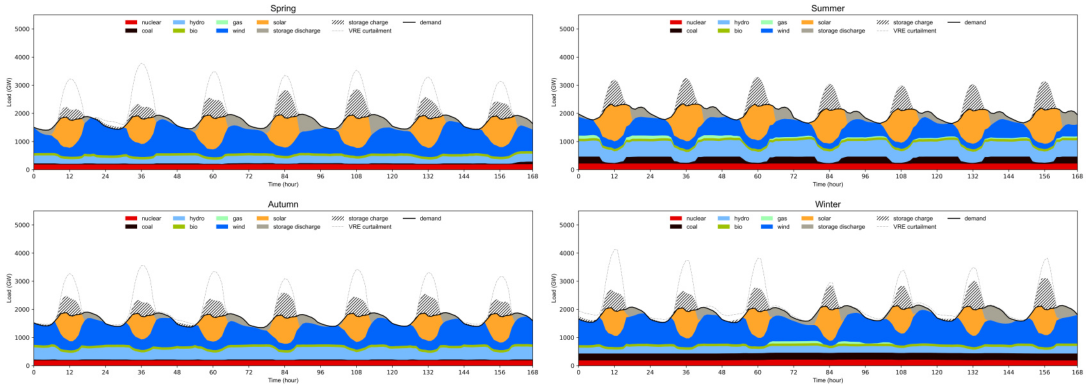

Fig. S87: Load profile: Gas Capacity 0.5X.

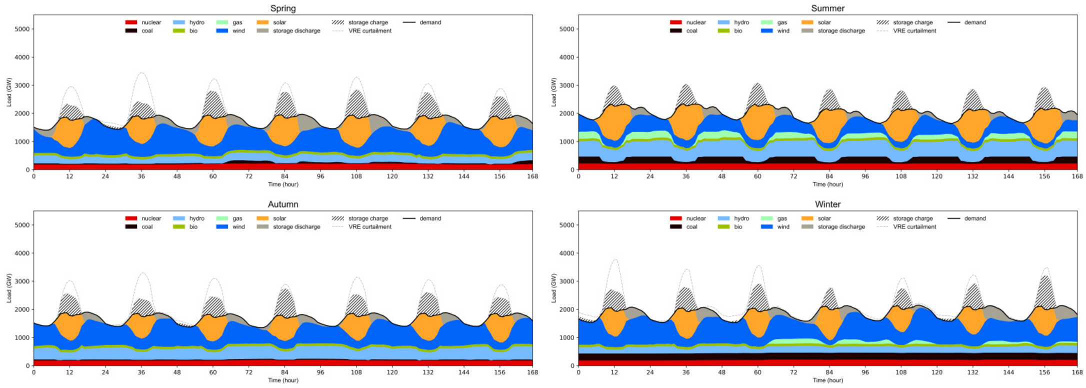

Fig. S88: Load profile: Gas ramp 0.25.

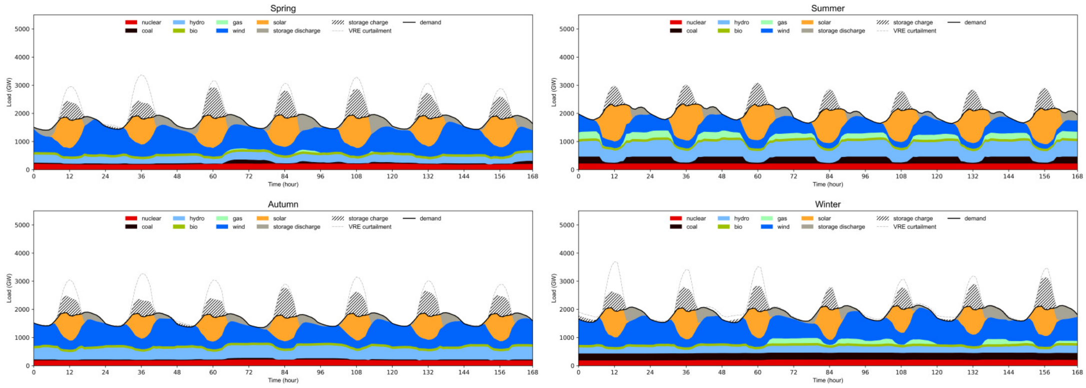

Fig. S89: Load profile: load reduction.

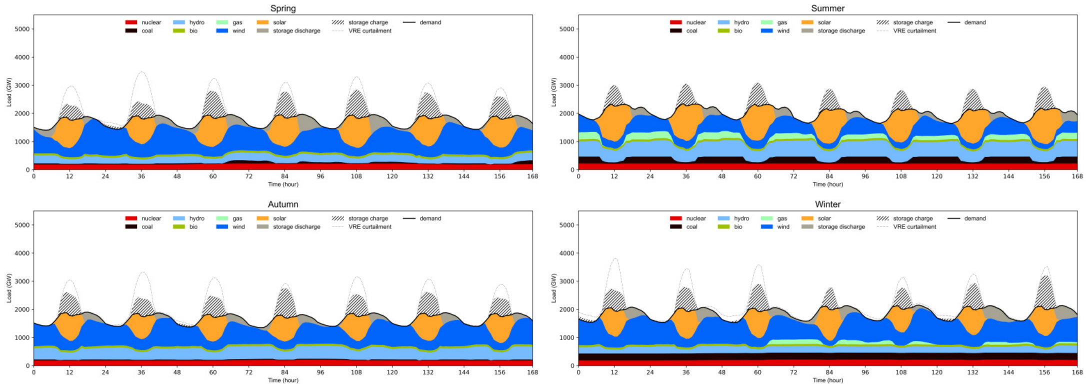

Fig. S90: Load profile: Neg Emis 400Mt.

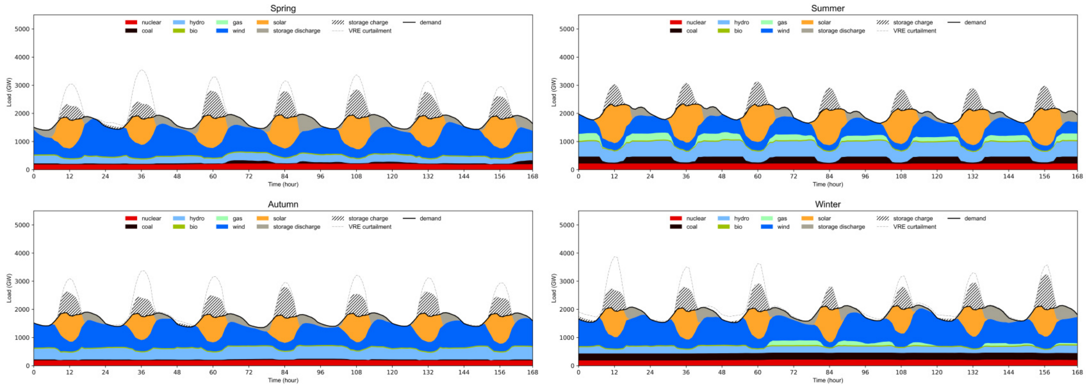

Fig. S91: Load profile: Neg Emis 200Mt.

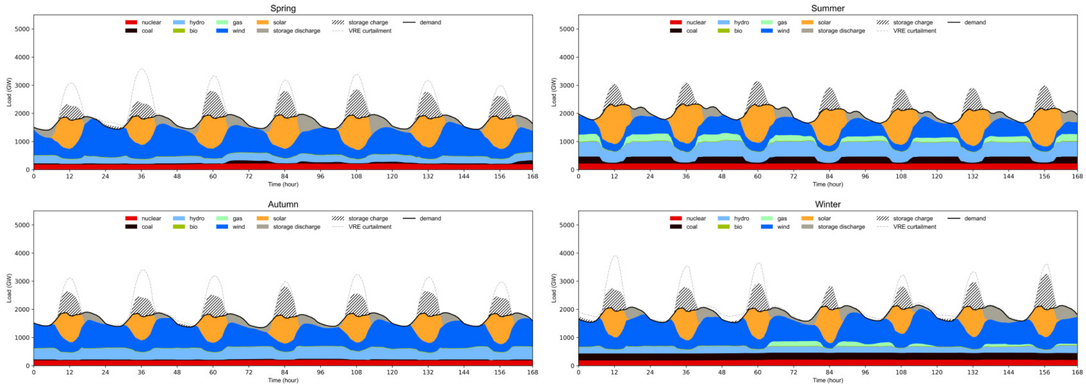

Fig. S92: Load profile: Net Zero Emis.

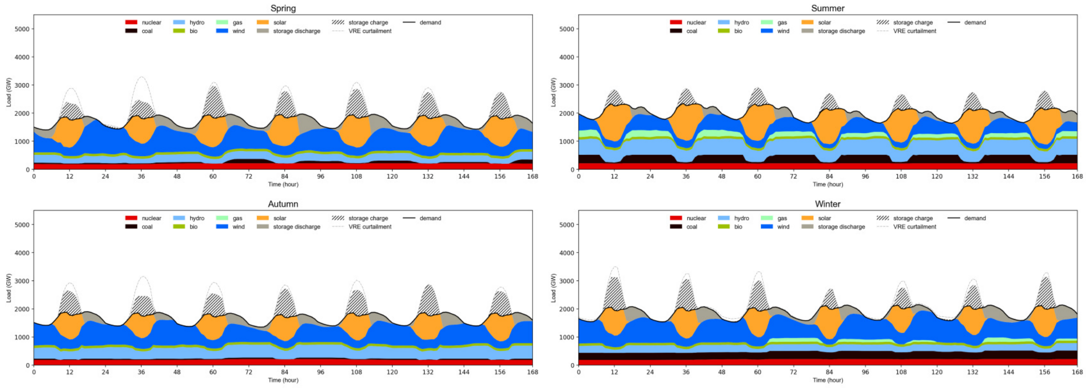

Fig. S93: Load profile: Two-year Period.

6.5 Storage operation

The charging and discharging patterns of PHS and battery storage aggregated at the national level are shown from Fig. S94 to Fig. S126. At each time step, a given energy storage may only charge or discharge, though different provinces may experience different charging or discharging states. We calculate the net charging or discharging capacity at the national level for each time step and generate these storage operation plots.

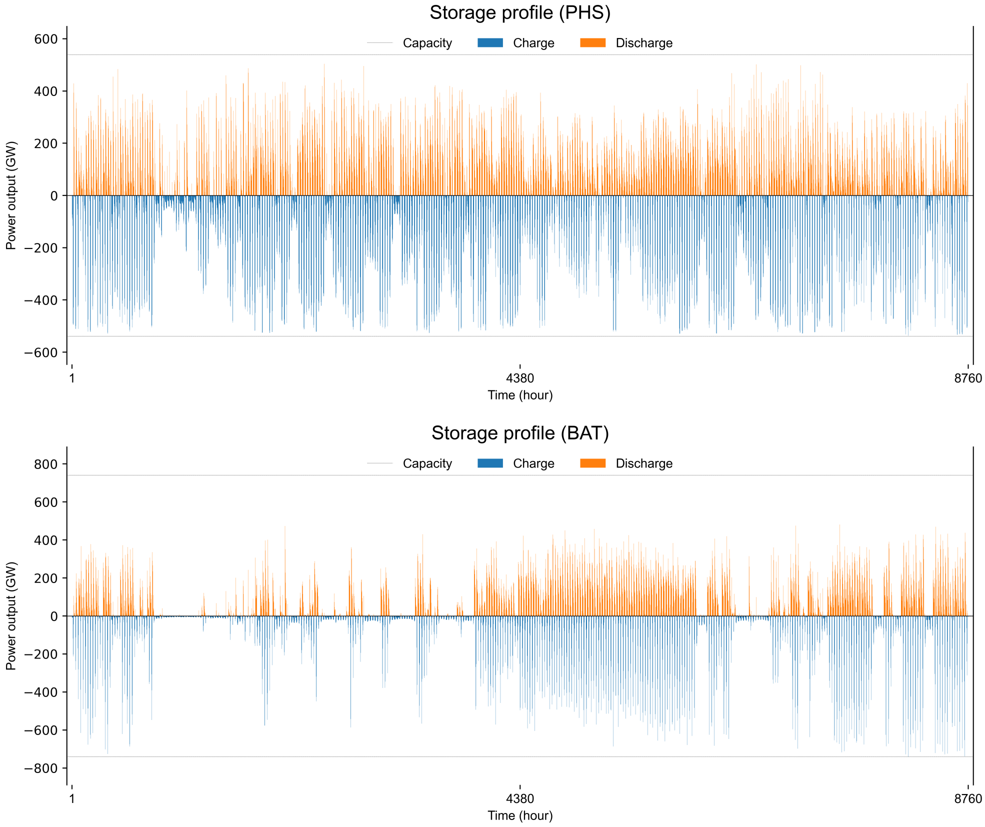

Fig. S94: Storage operation: Base case.

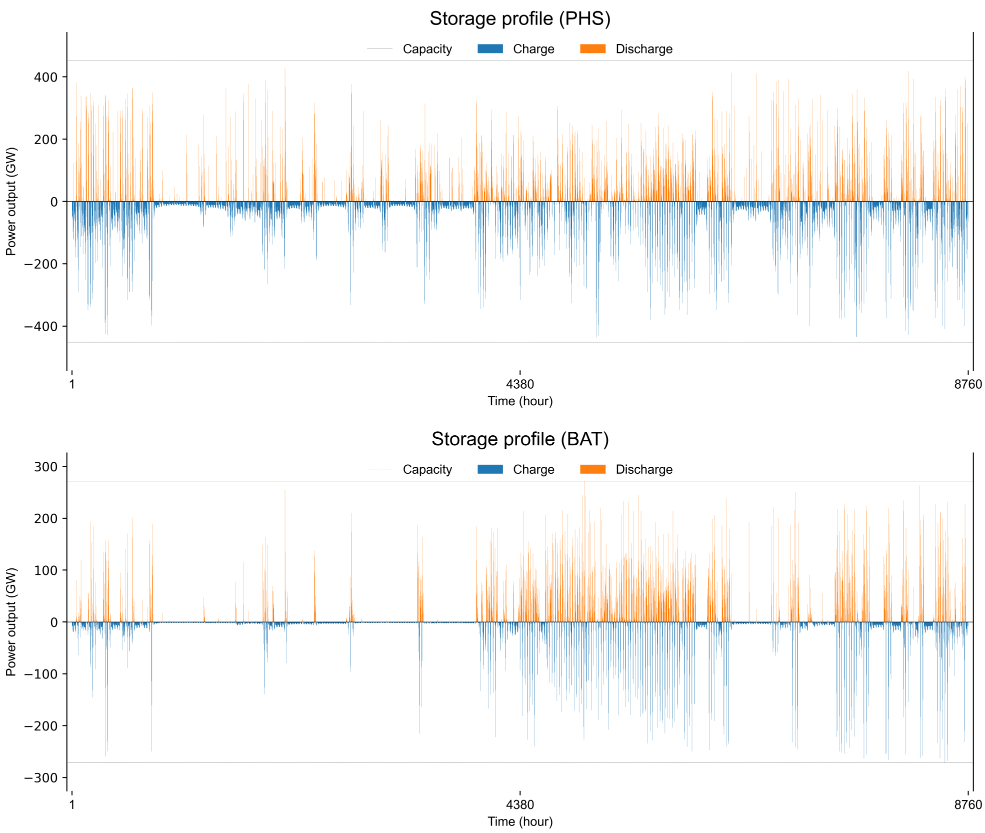

Fig. S95: Storage operation: Wind CapEx 0.5X.

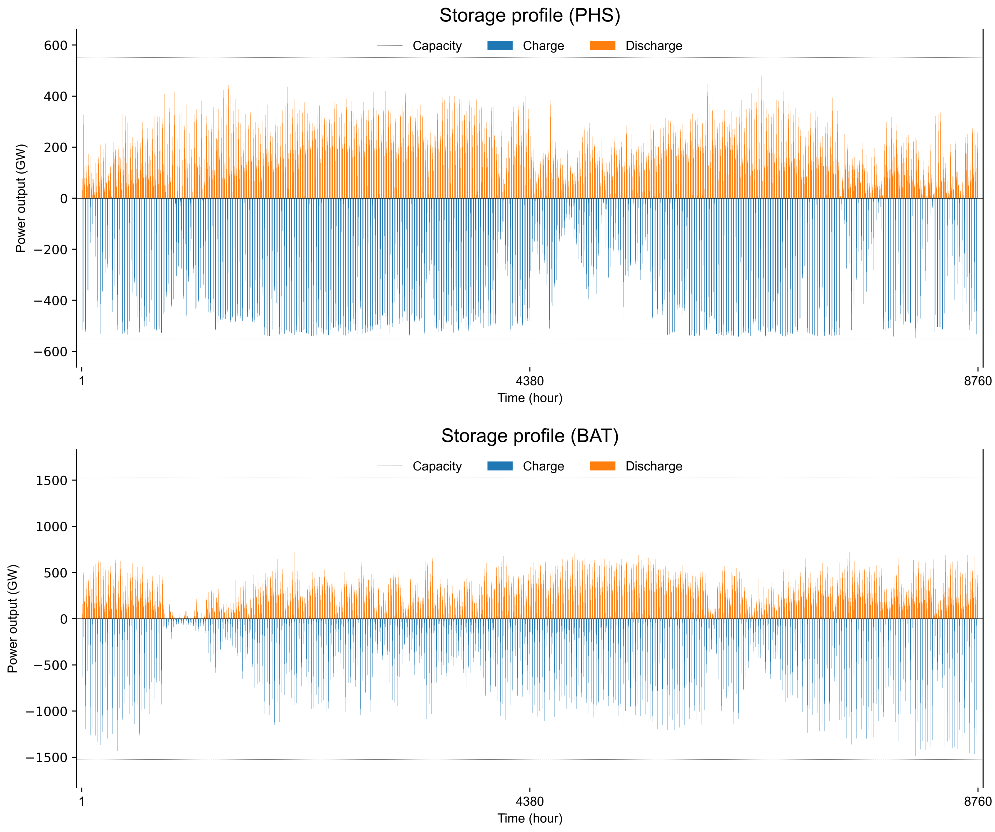

Fig. S96: Storage operation: Wind CapEx 2X.

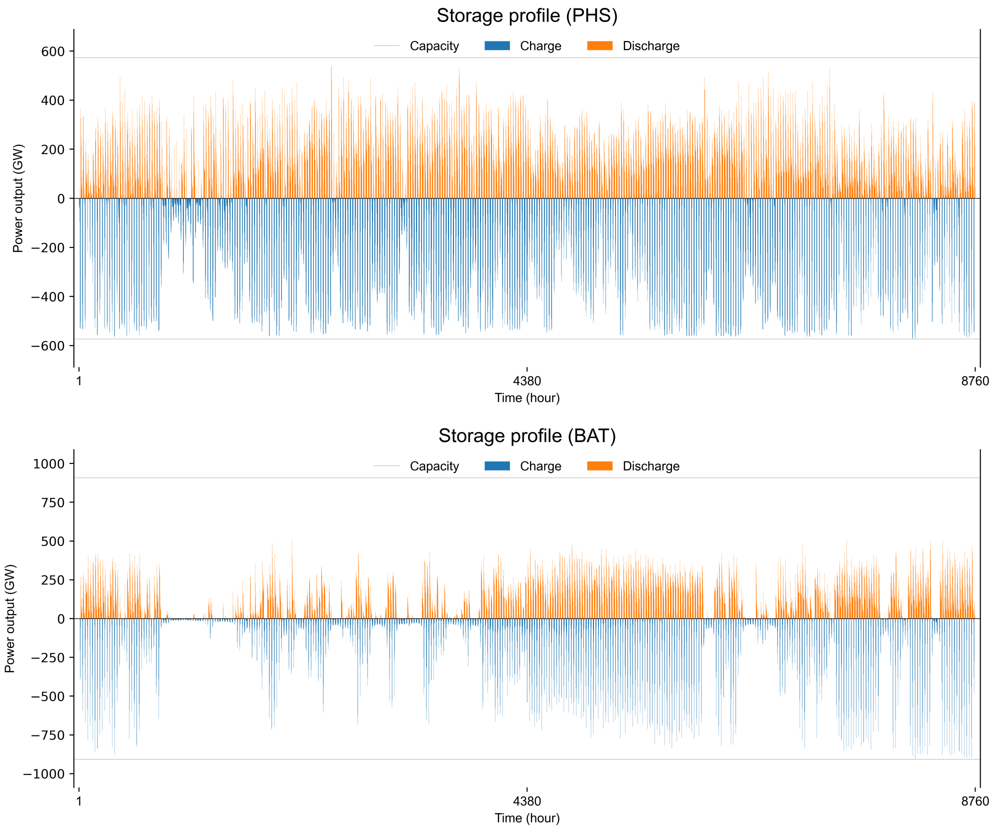

Fig. S97: Storage operation: Solar CapEx 0.5X.

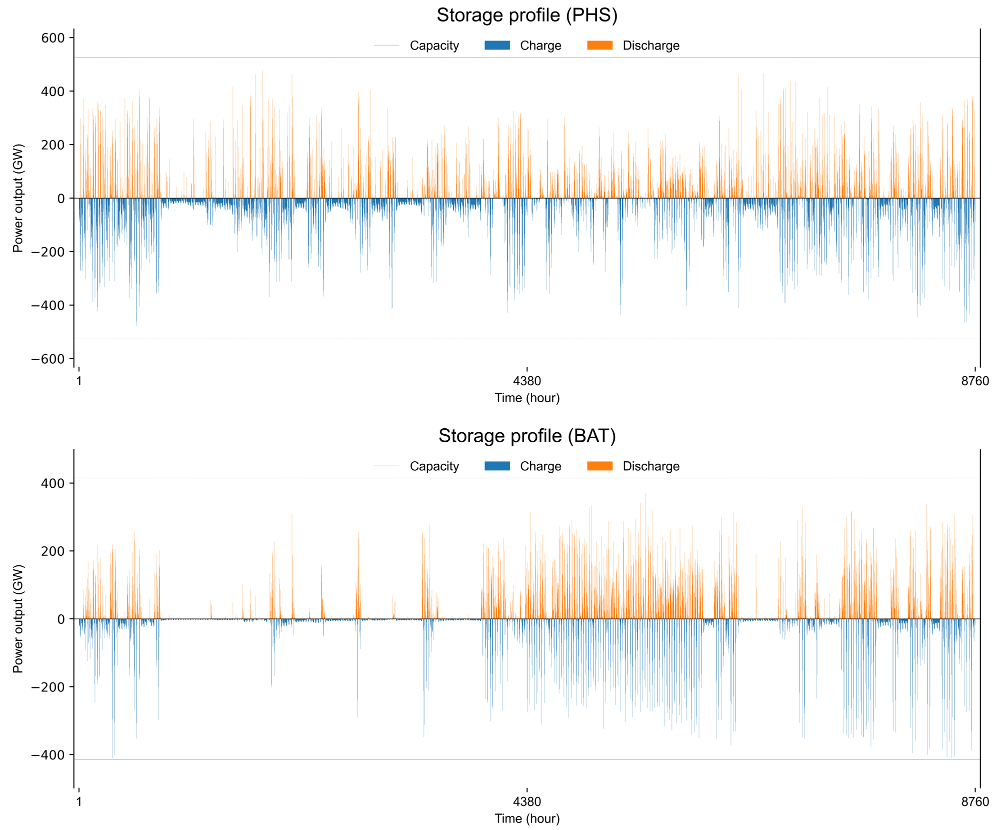

Fig. S98: Storage operation: Solar CapEx 2X.

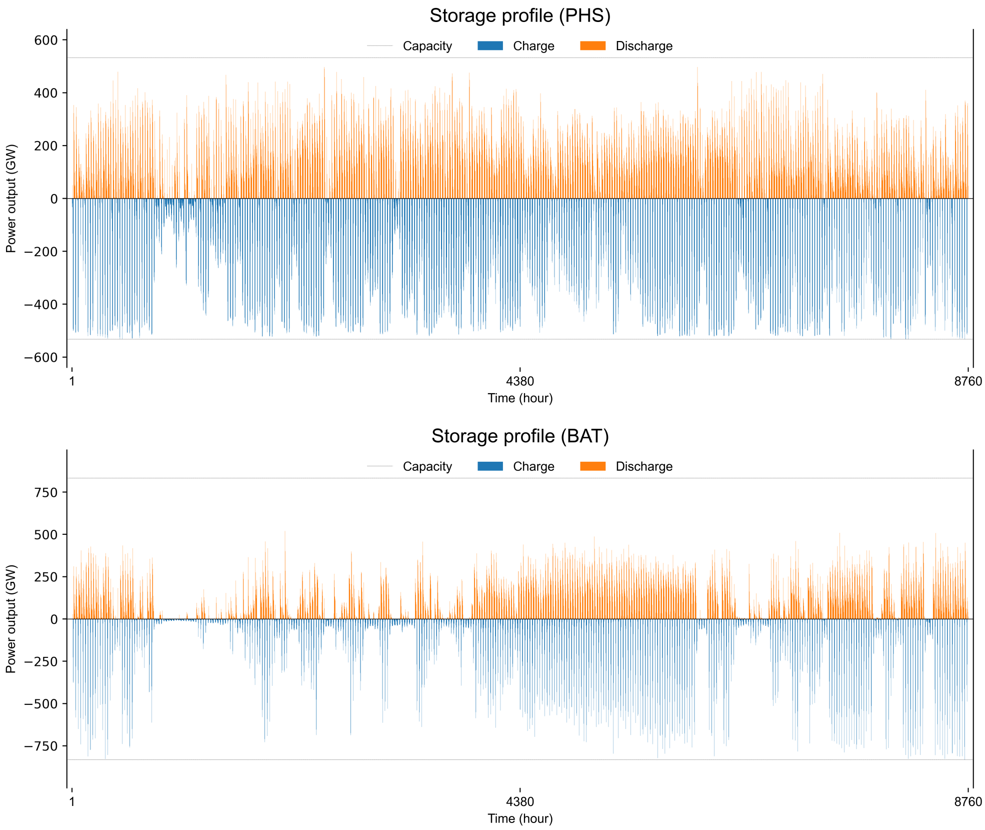

Fig. S99: Storage operation: Wind open + solar open.

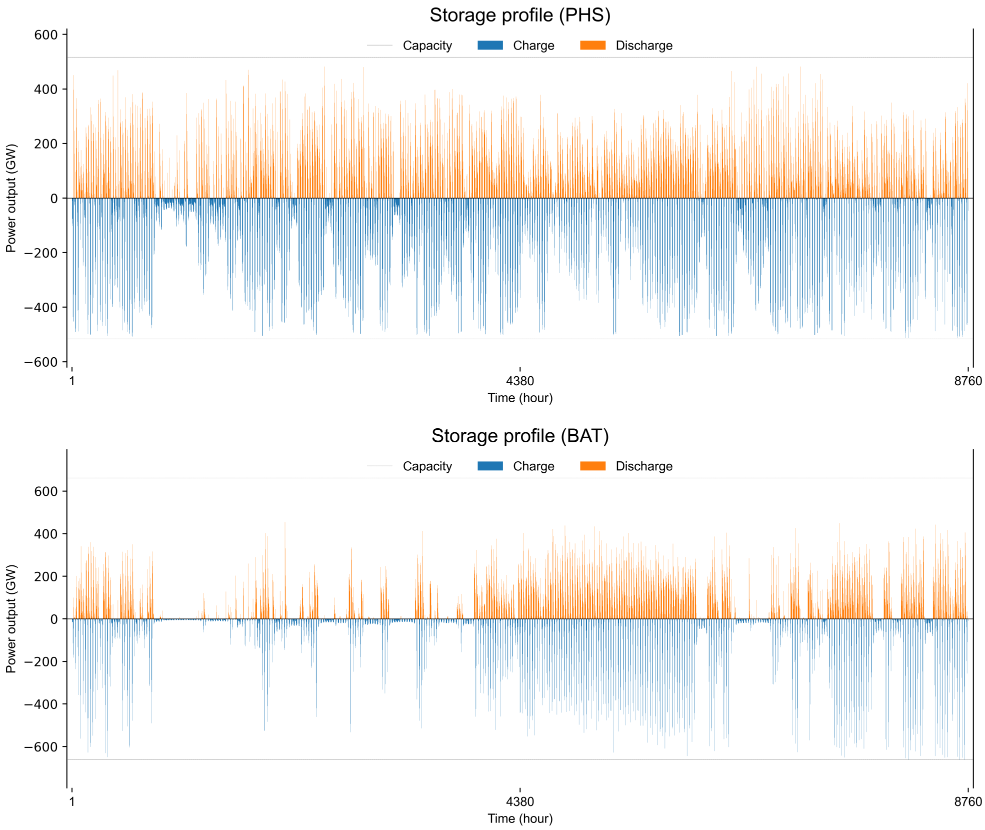

Fig. S100: Storage operation: Wind open + solar base.

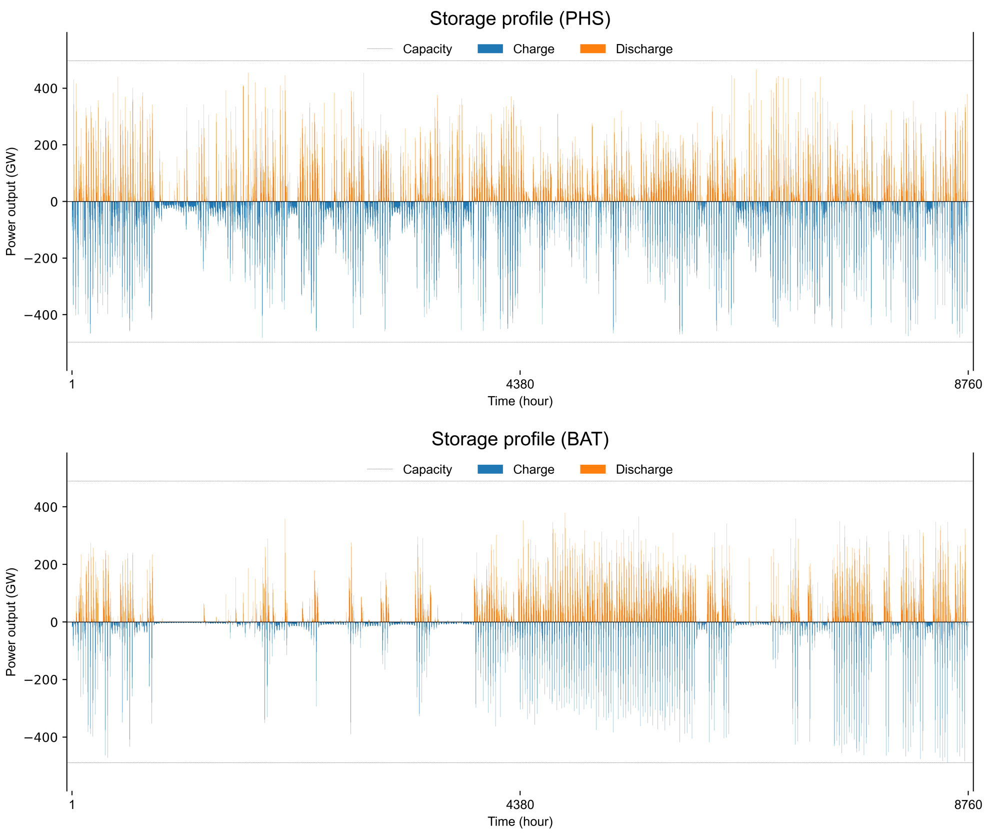

Fig. S101: Storage operation: Wind open + solar conservative.

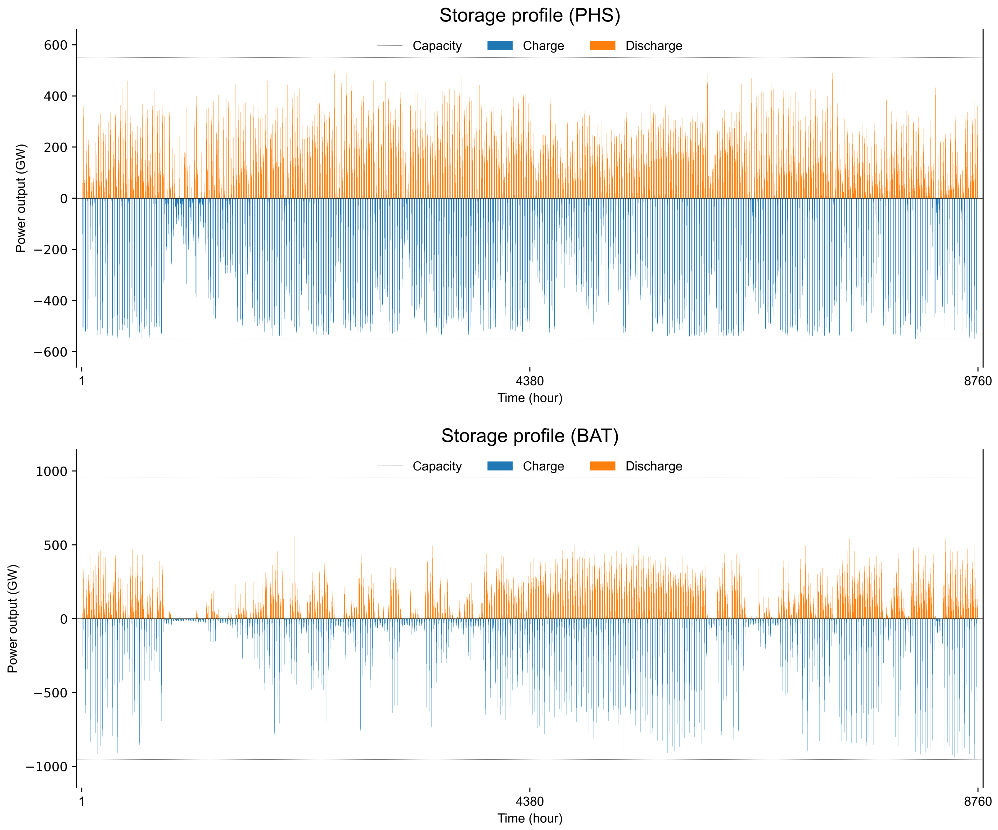

Fig. S102: Storage operation: Wind base + solar open.

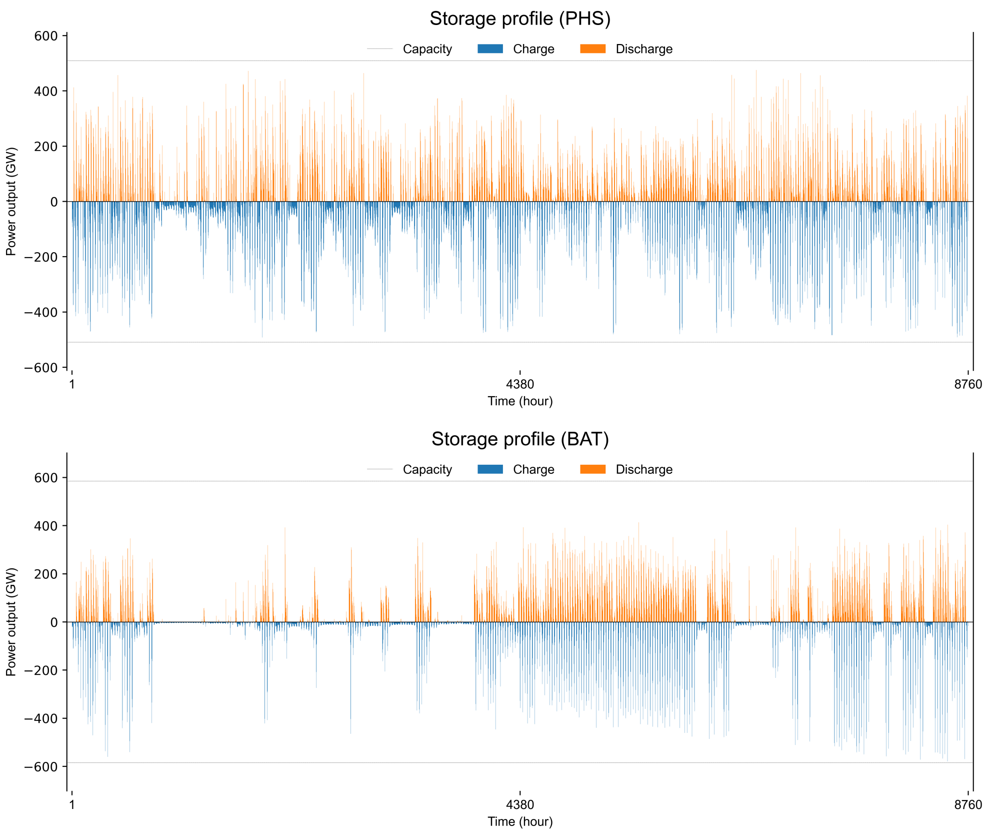

Fig. S103: Storage operation: Wind base + solar conservative.

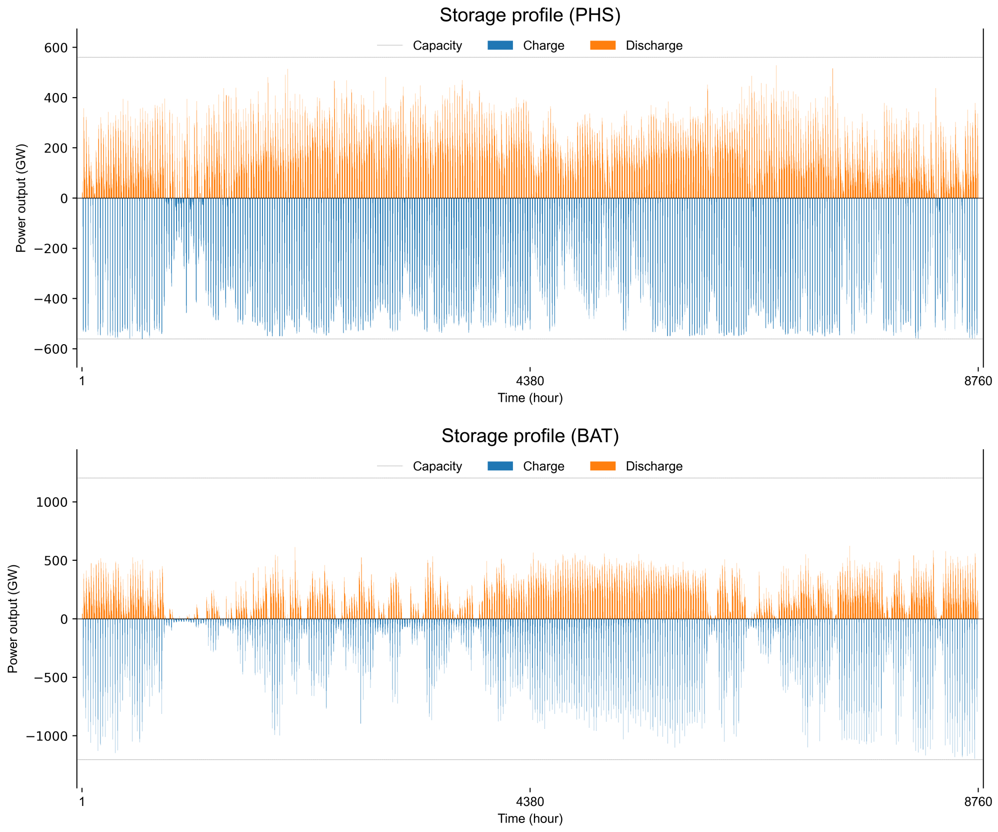

Fig. S104: Storage operation: Wind conservative + solar open.

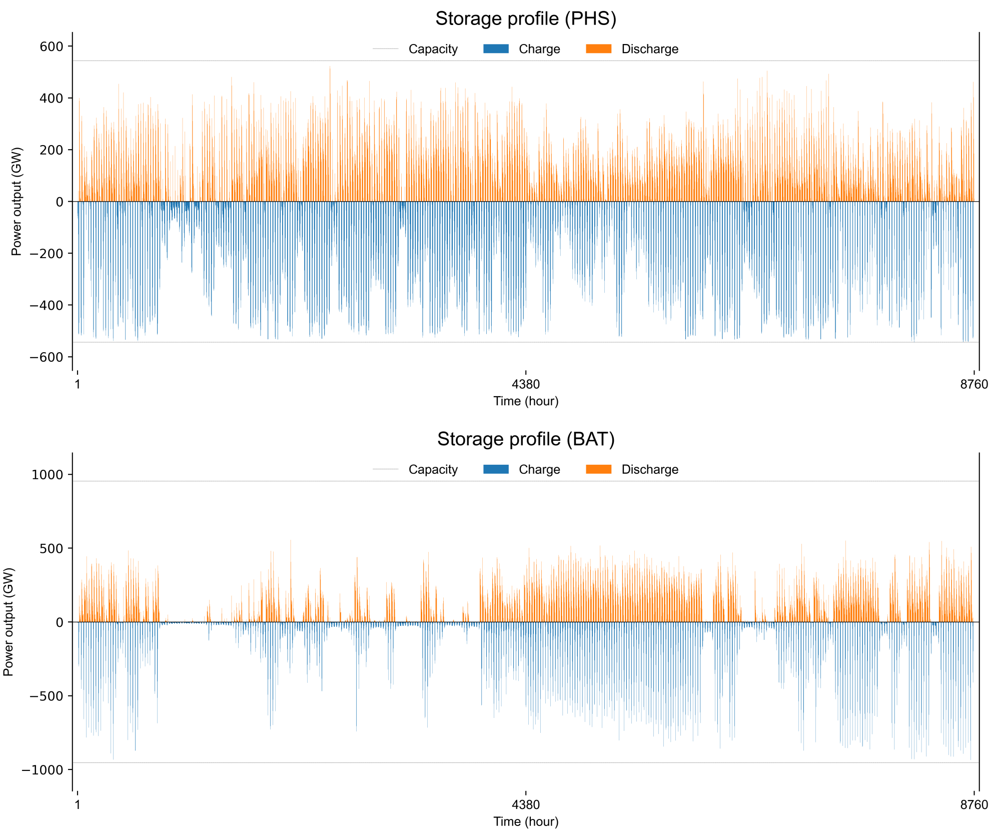

Fig. S105: Storage operation: Wind conservative + solar base.

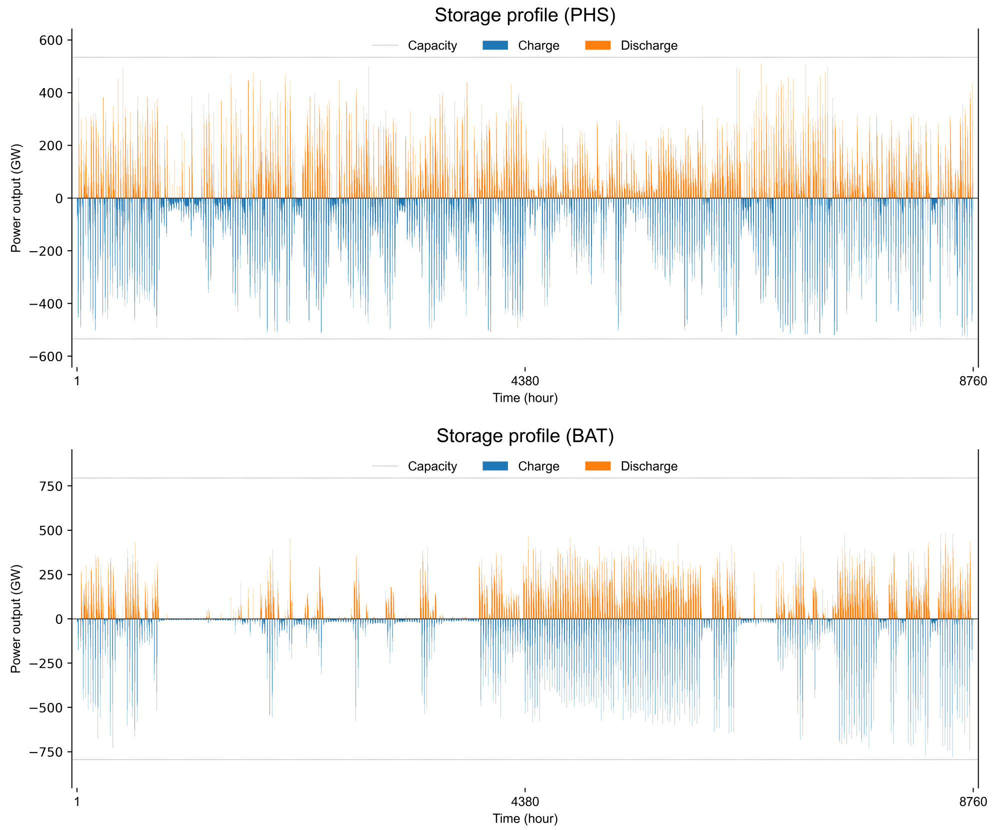

Fig. S106: Storage operation: Wind conservative + solar conservative.

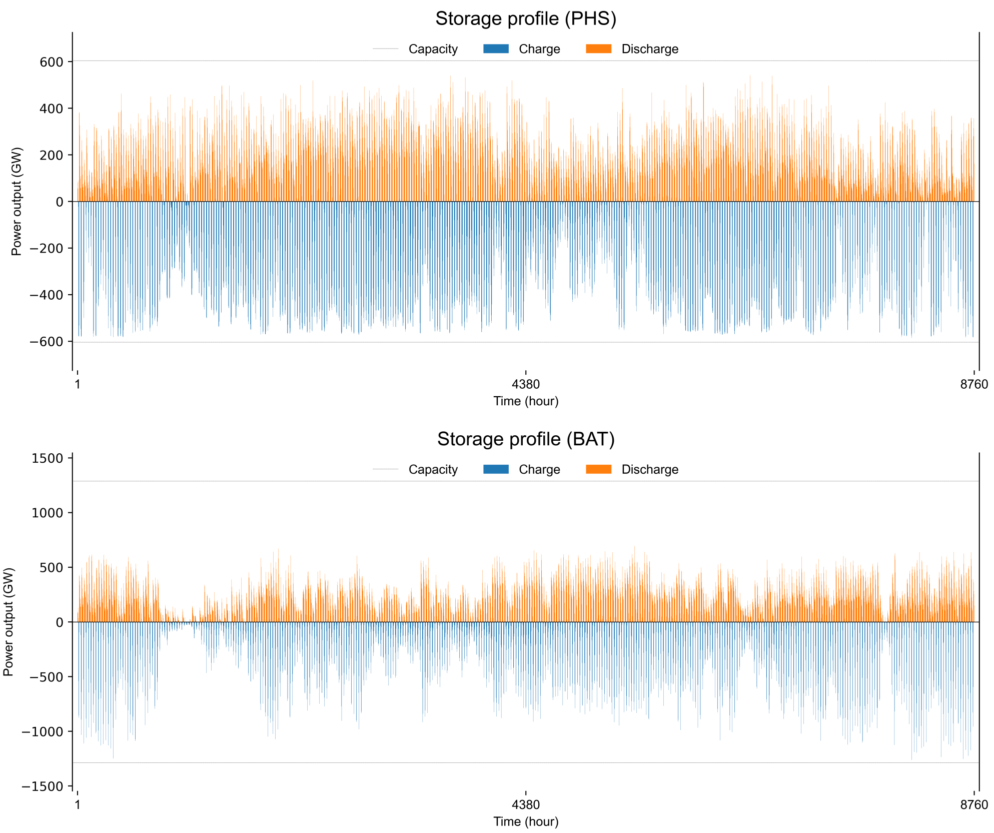

Fig. S107: Storage operation: VRE Cell Clustered.

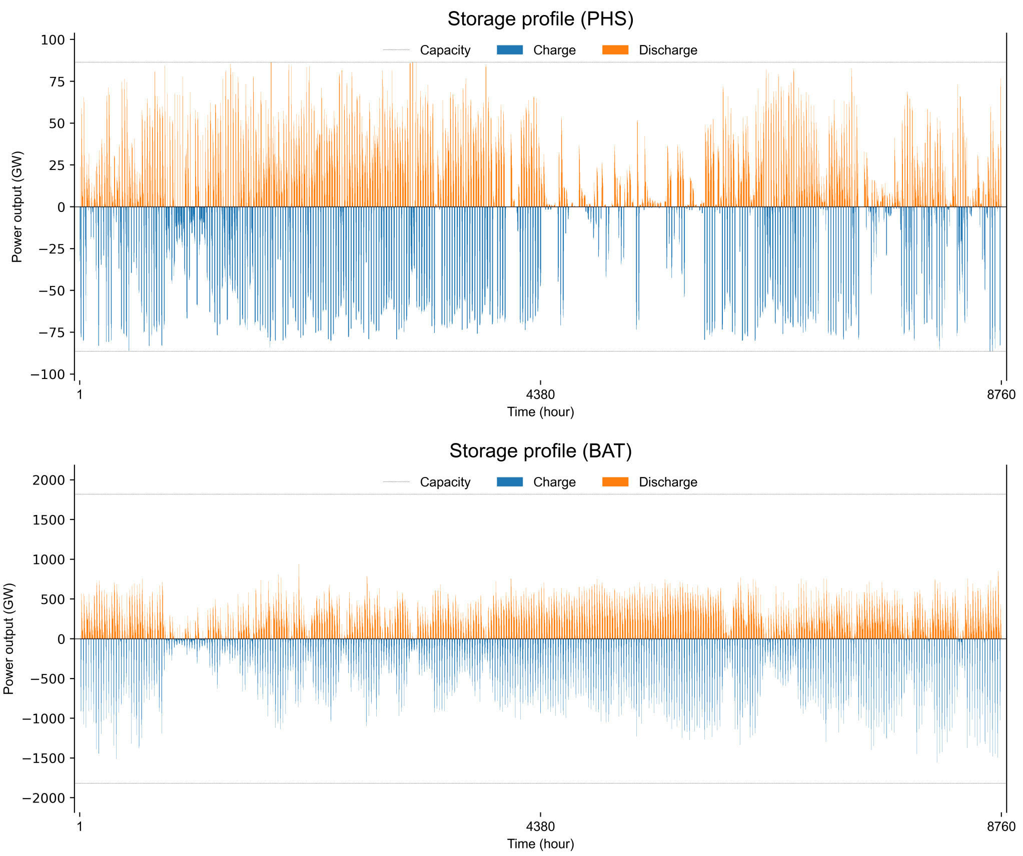

(a) Battery CAPEX 0.5X

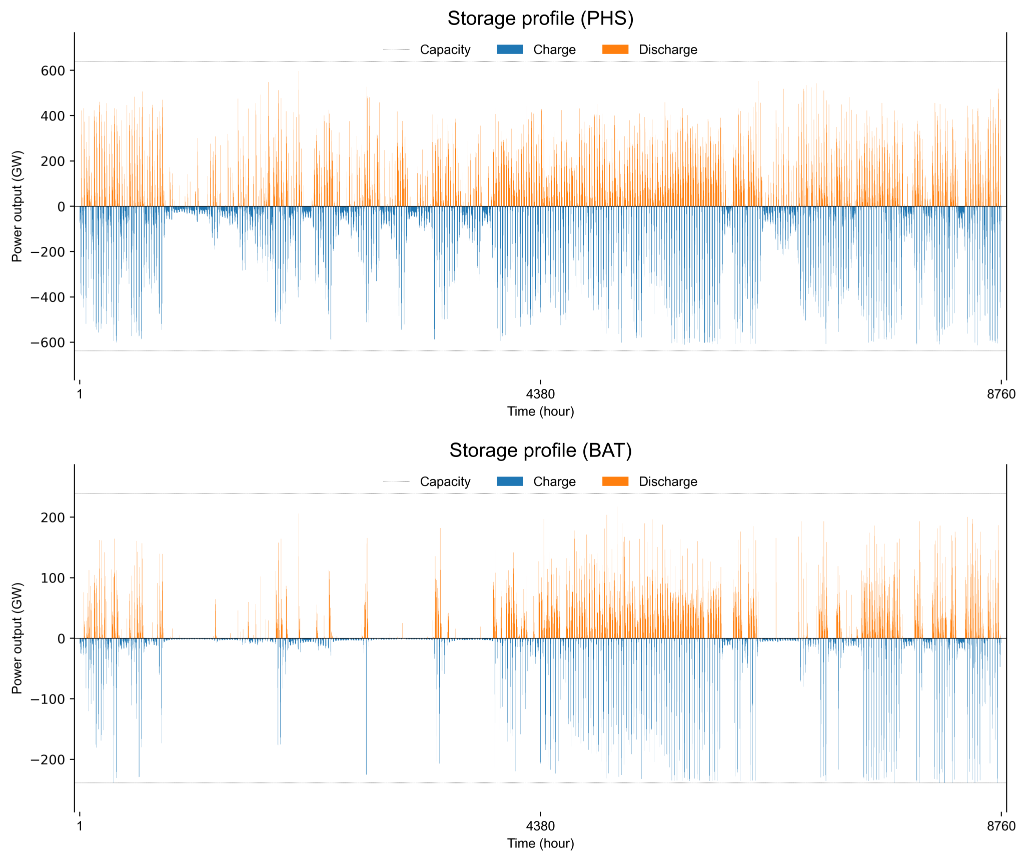

(b) Battery CAPEX 2X

Fig. S108: Storage operation: Battery CapEx 0.5X and 2X.

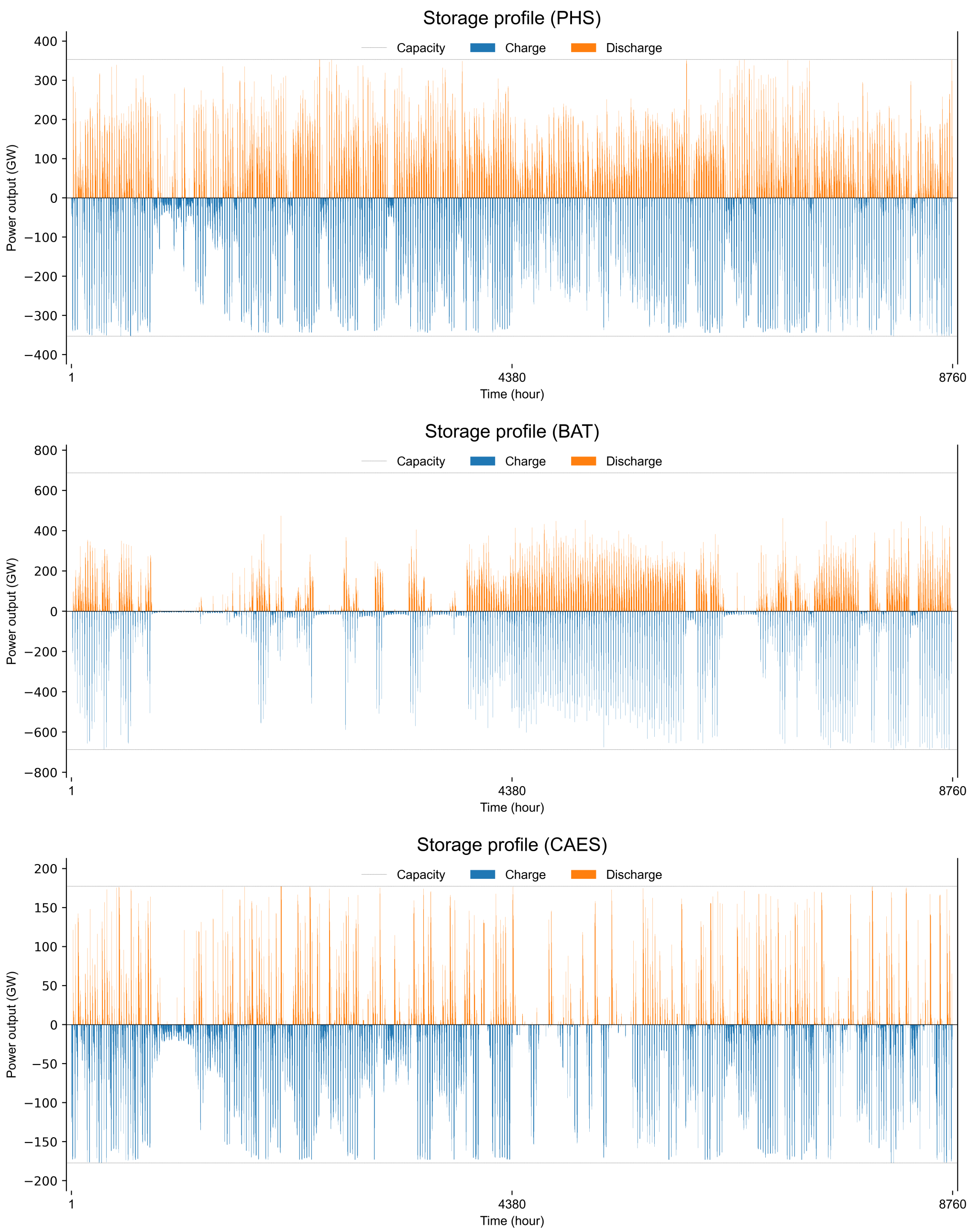

Fig. S109: Storage operation: With Caes.

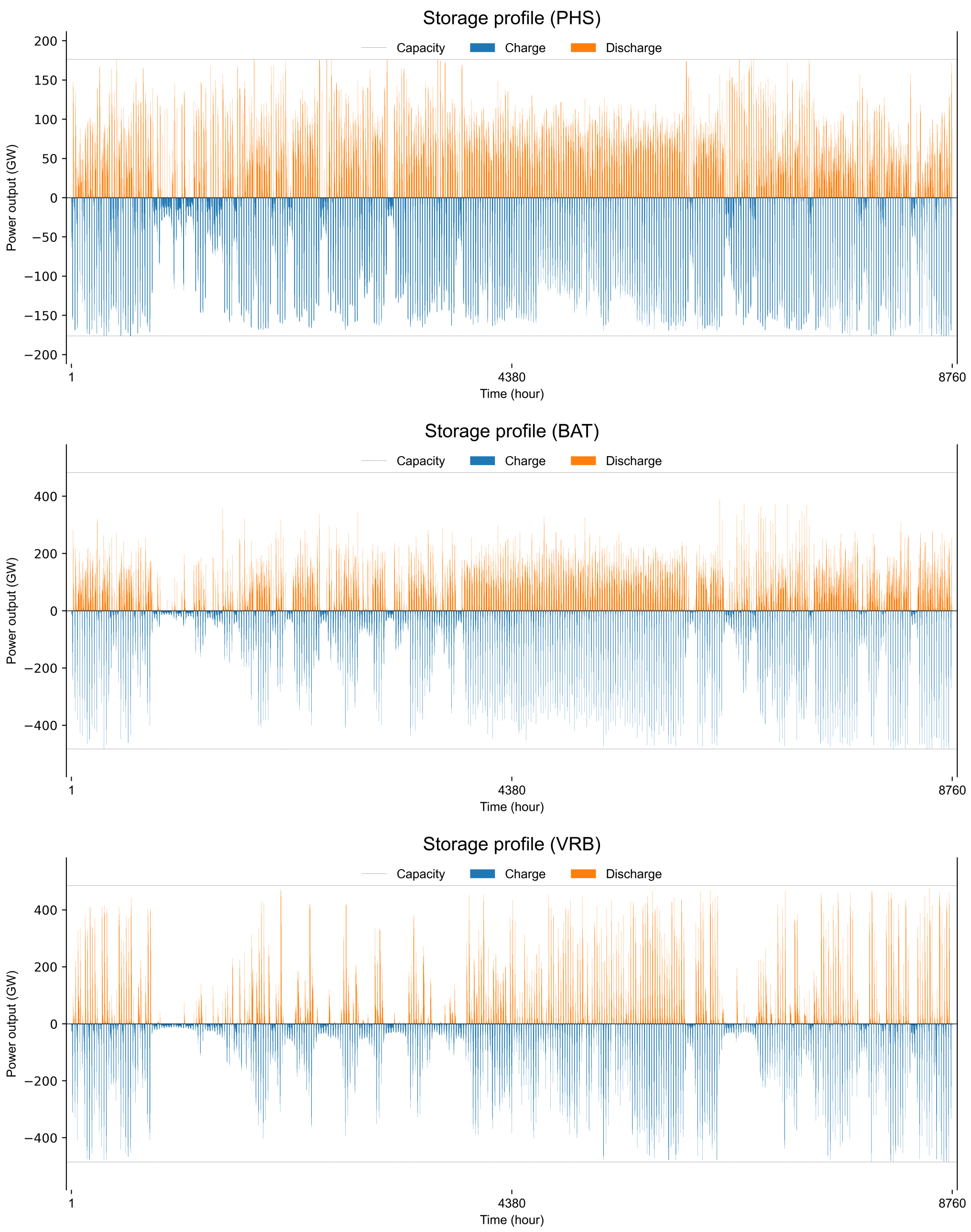

Fig. S110: Storage operation: With VRB.

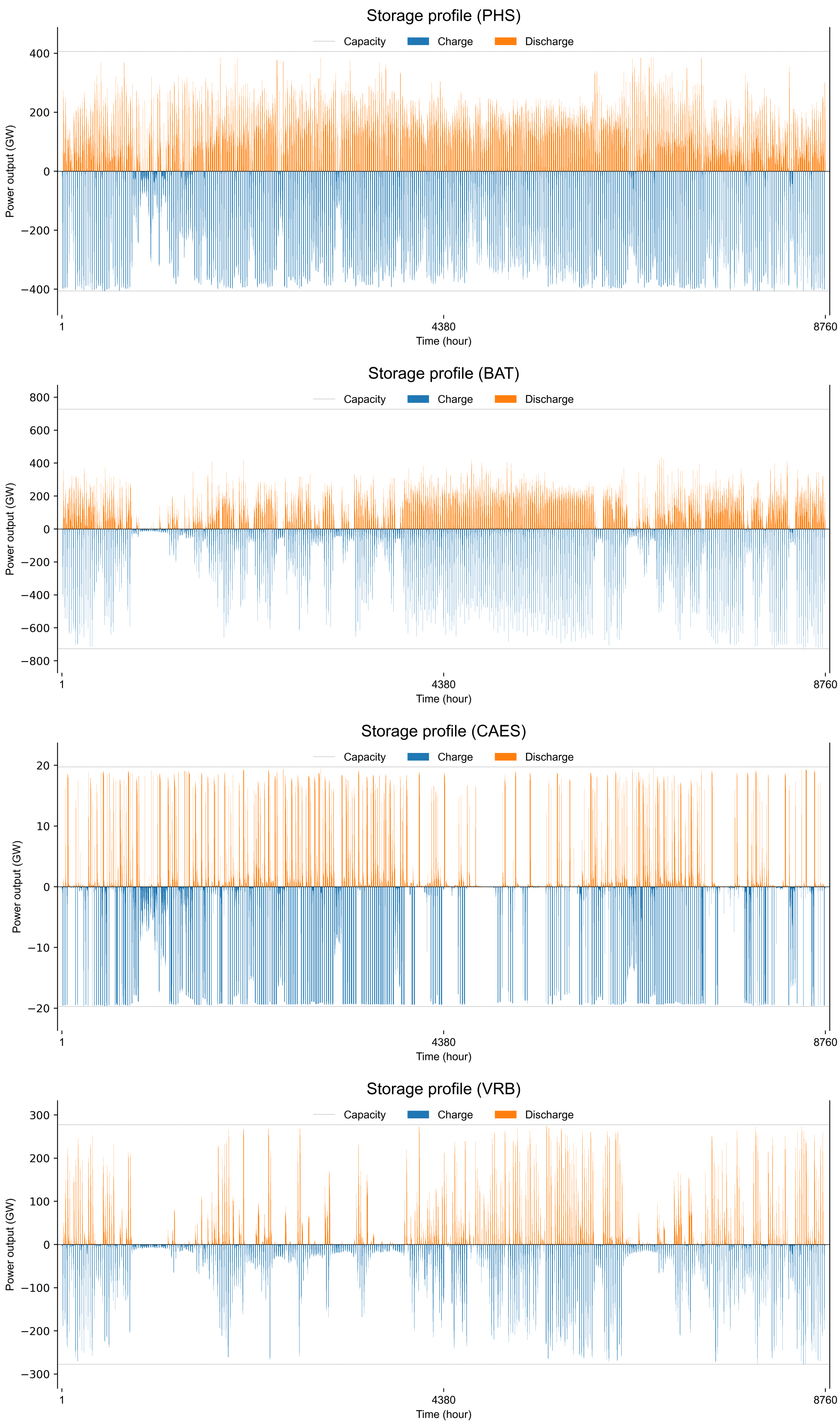

Fig. S111: Storage operation: With CAES and VRB.

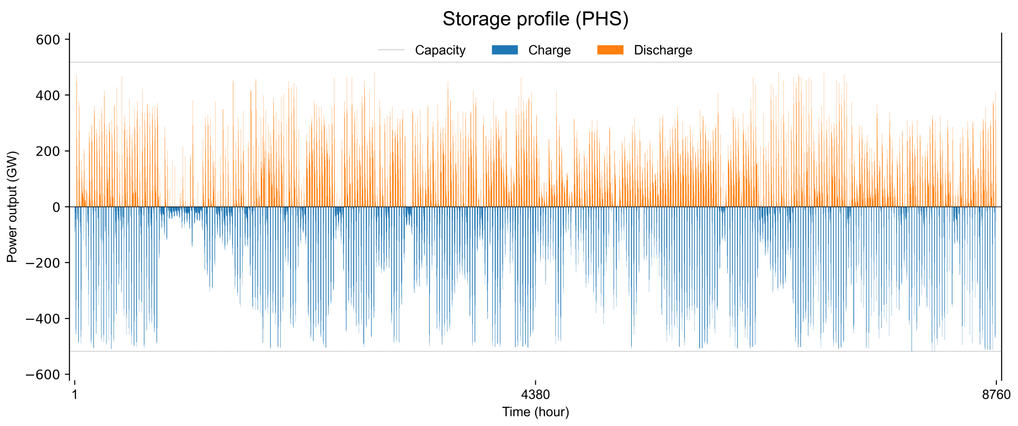

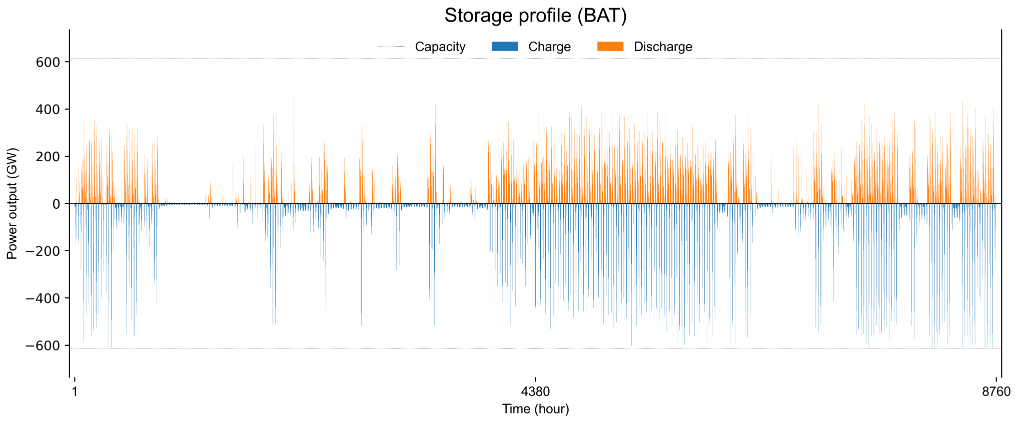

Fig. S112: Storage operation: UHV CapEx 0.5X.

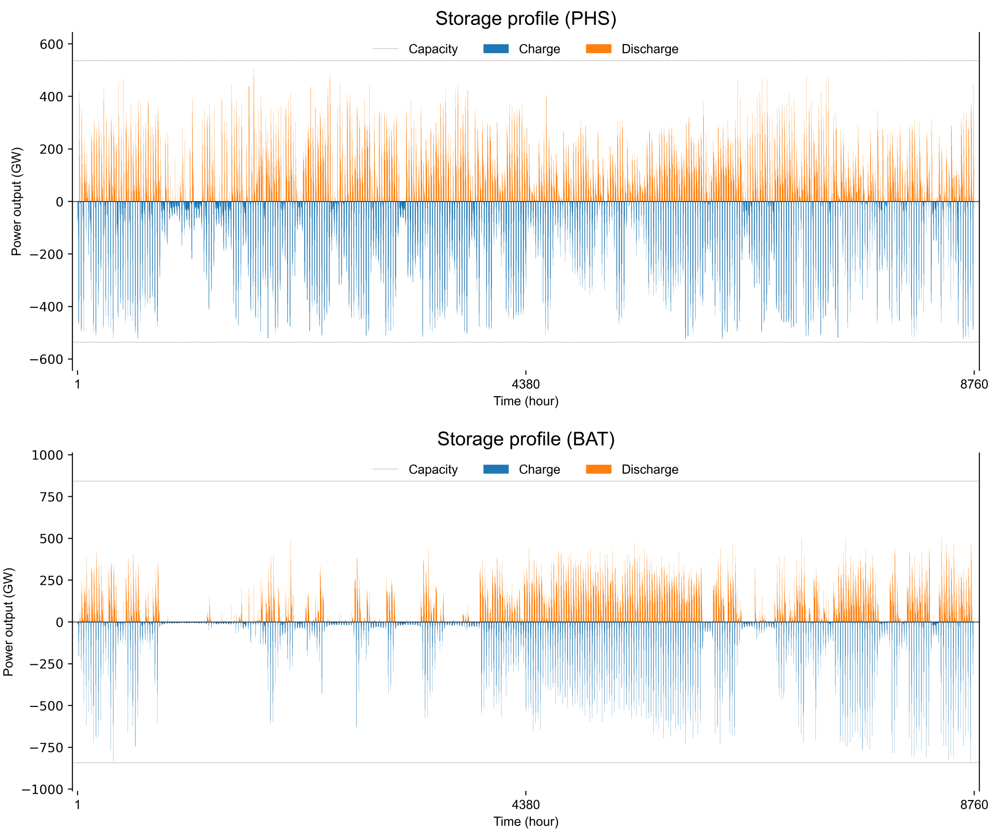

Fig. S113: Storage operation: UHV CapEx 2X.

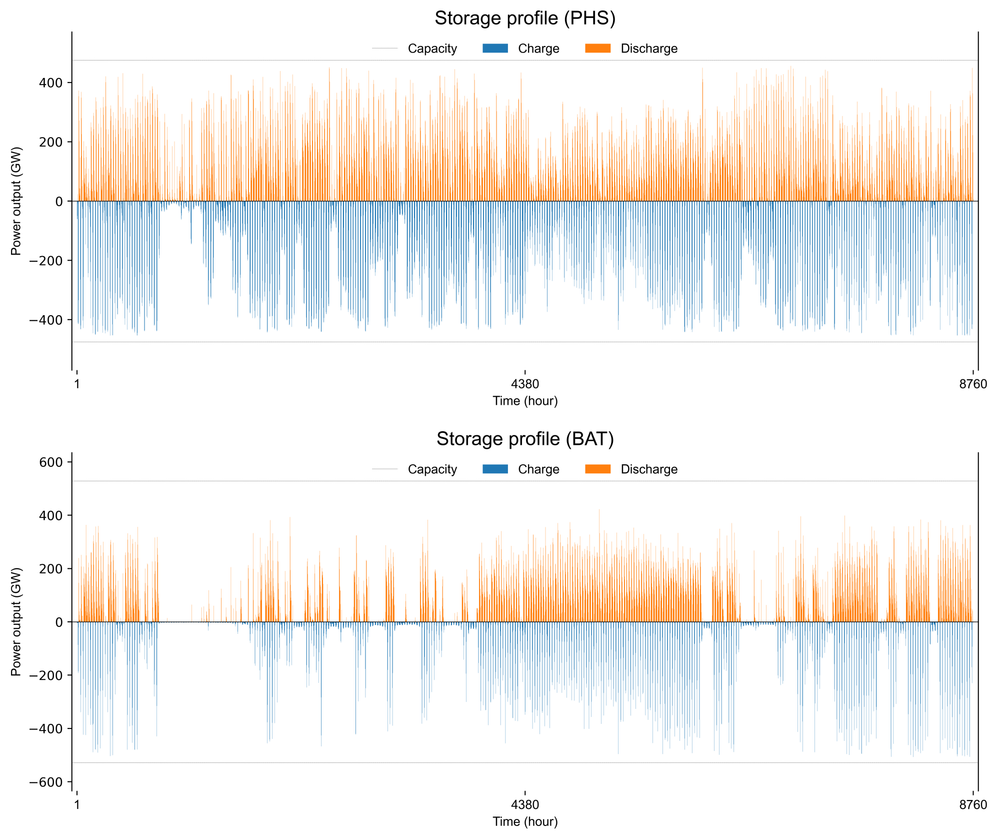

Fig. S114: Storage operation: Unconstrained UHV Expansion.

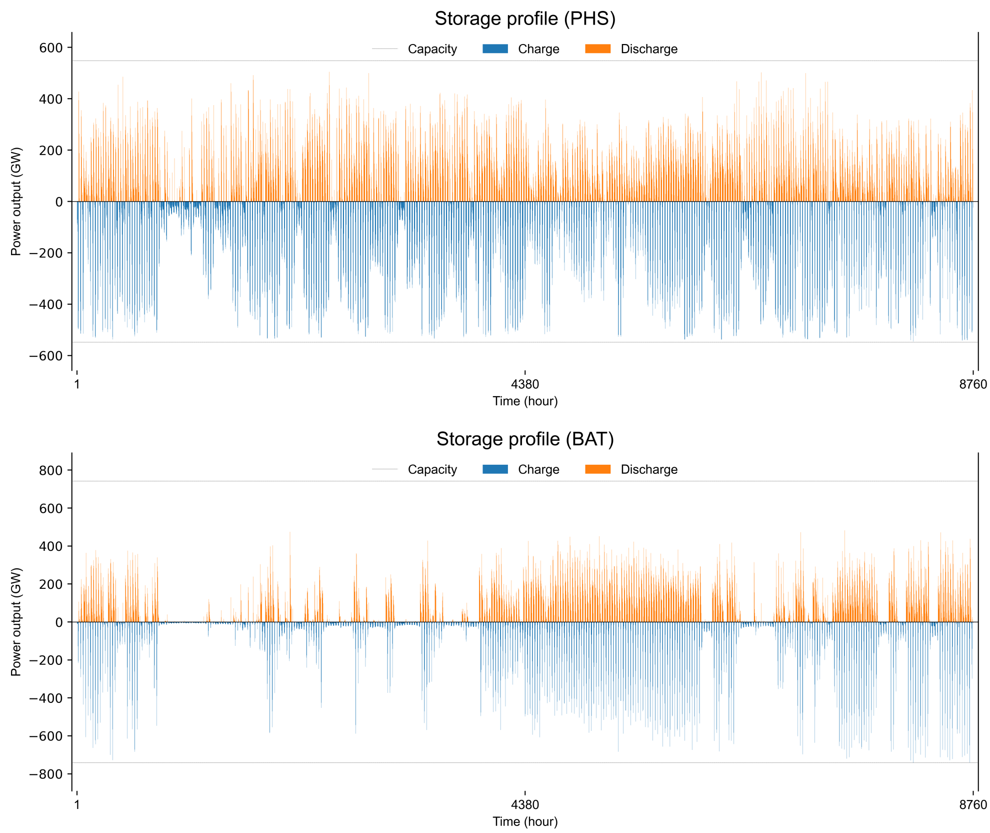

Fig. S115: Storage operation: Resv Ratio 2X.

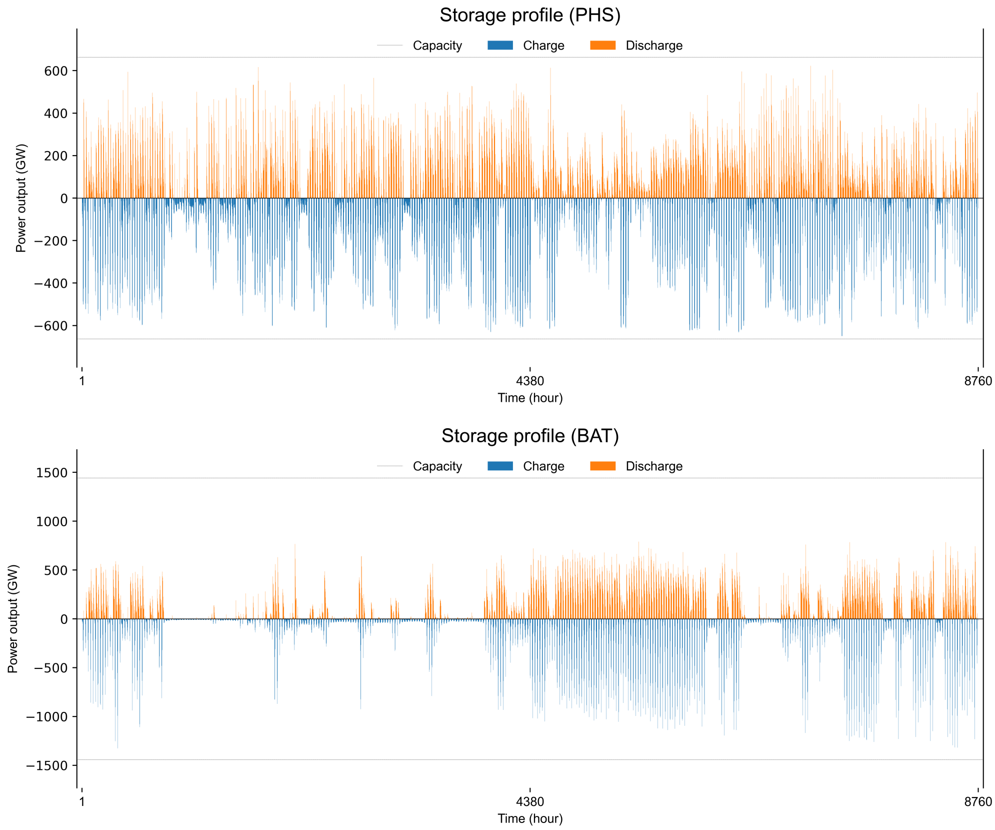

Fig. S116: Storage operation: Demand 1.2X.

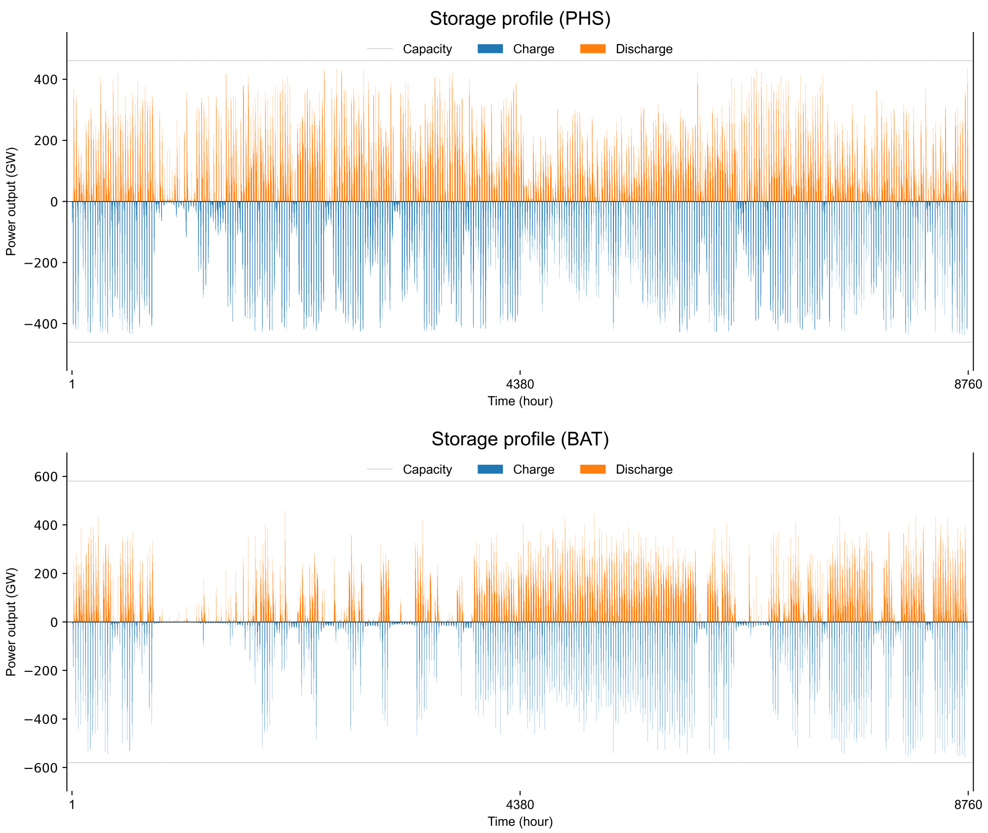

Fig. S117: Storage operation: Coal CCS 350 GW.

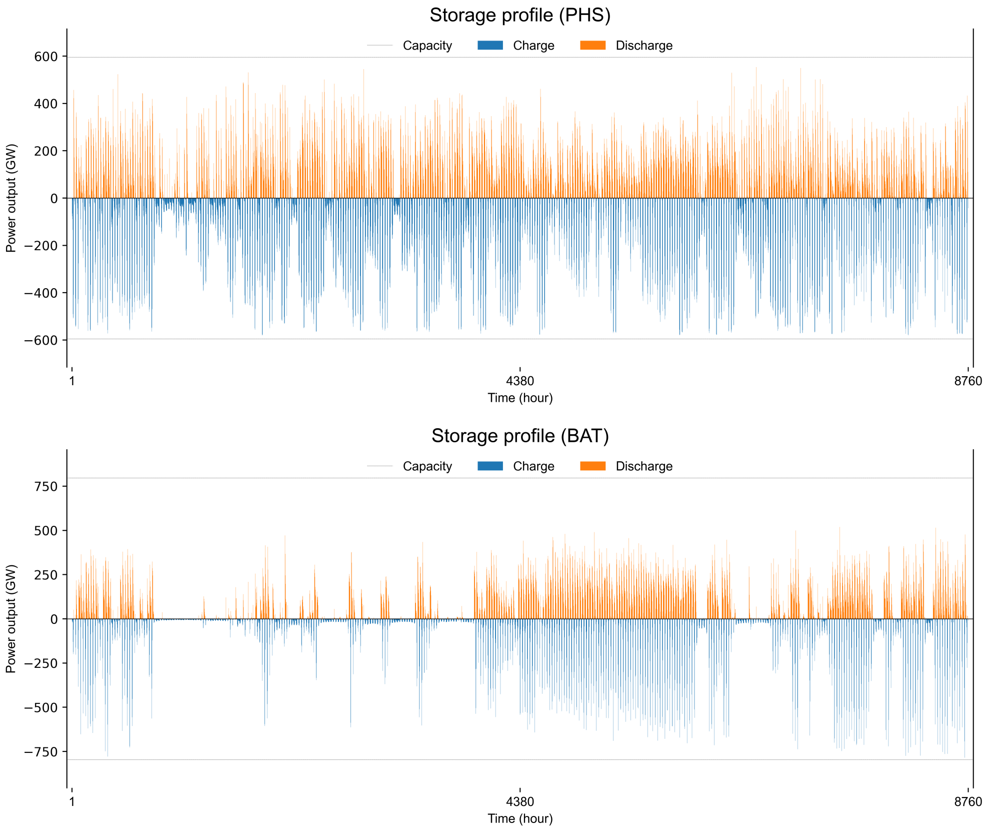

Fig. S118: Storage operation: Nuclear 150GW.

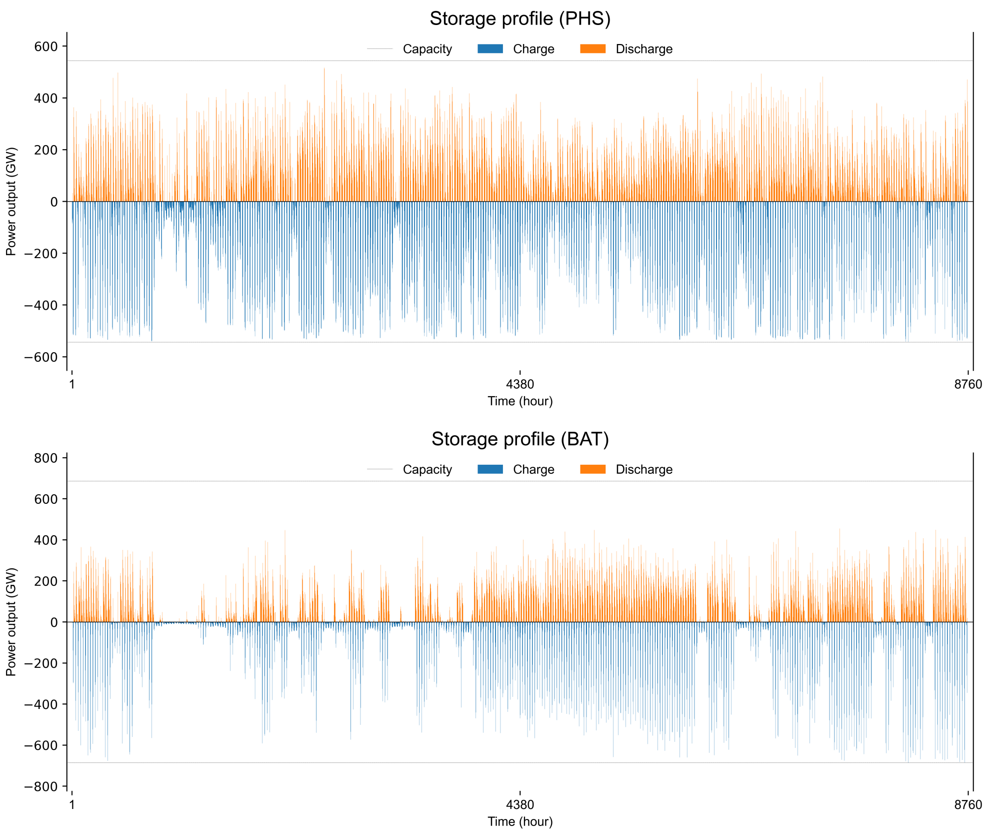

Fig. S119: Storage operation: Nuclear Flex 0.5.

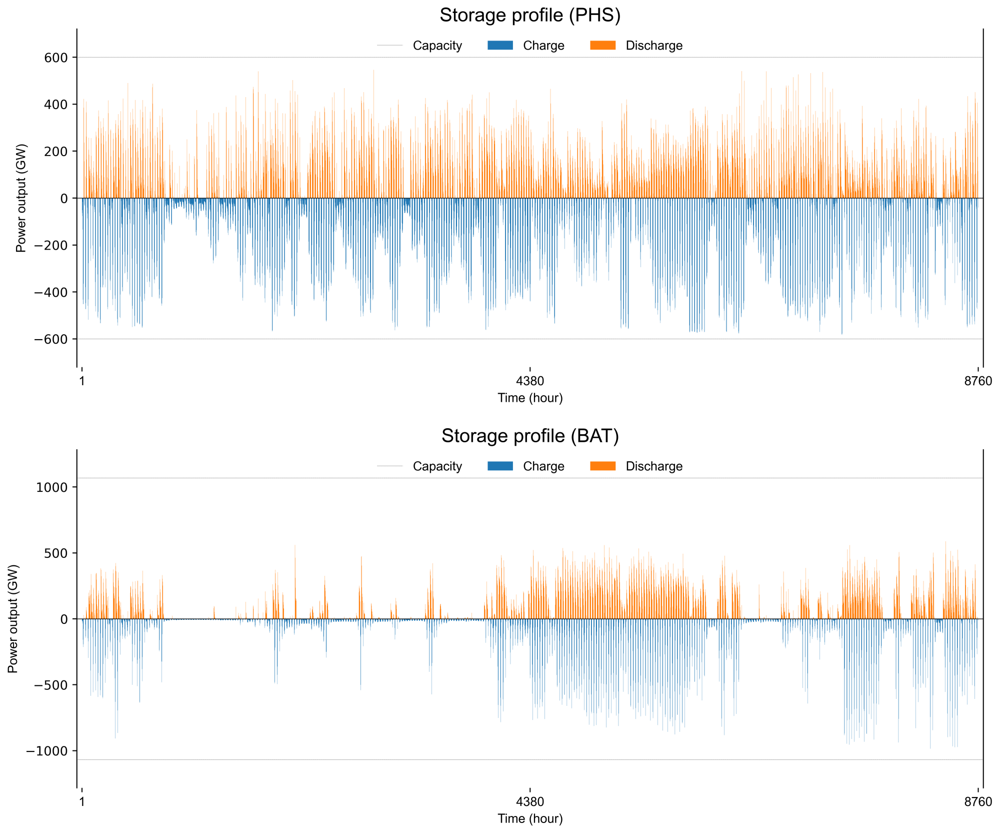

Fig. S120: Storage operation: Gas Capacity 0.5X.

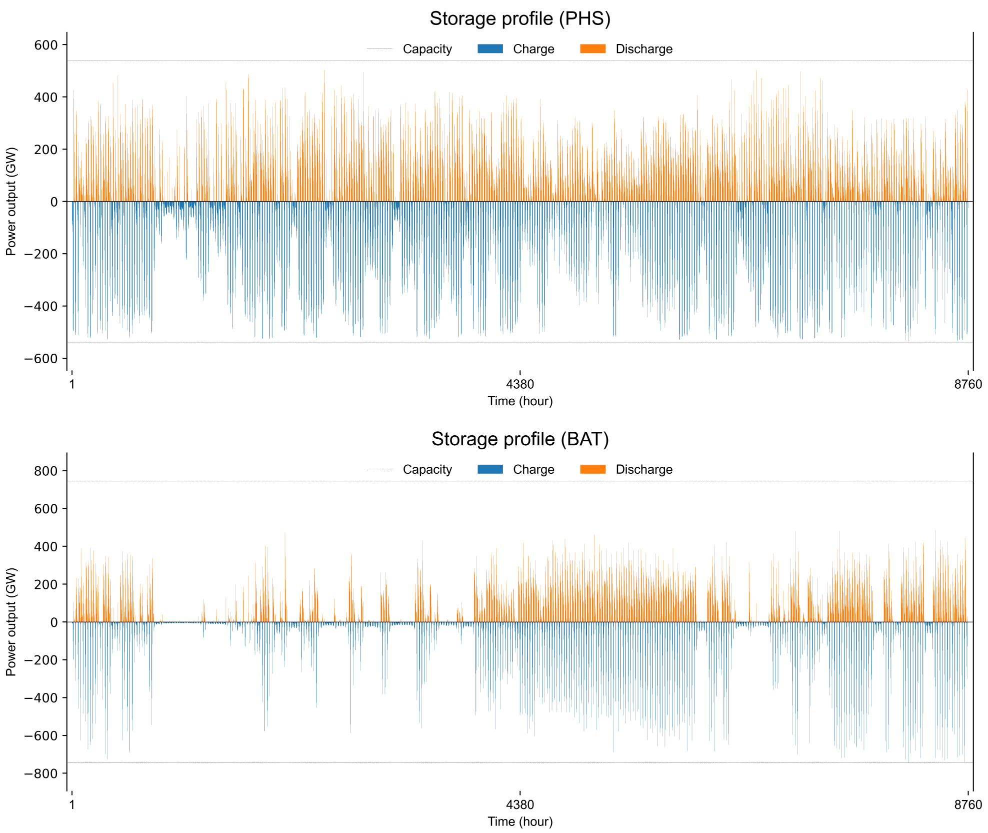

Fig. S121: Storage operation: Gas ramp 0.25.

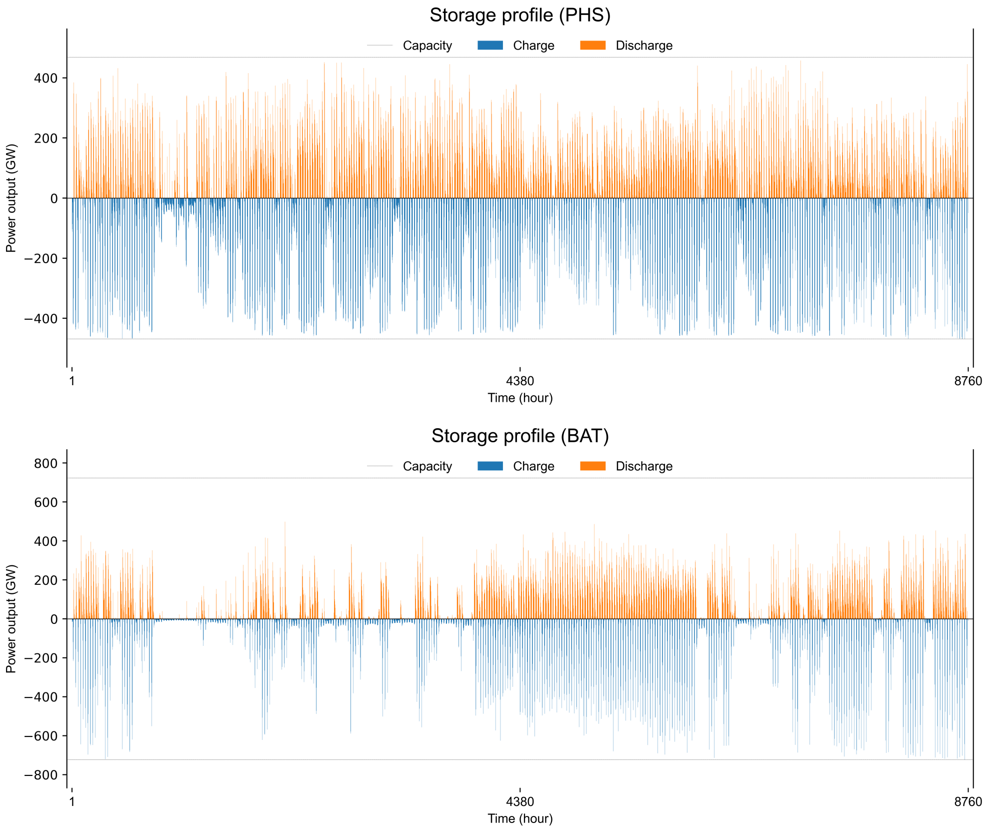

Fig. S122: Storage operation: load reduction.

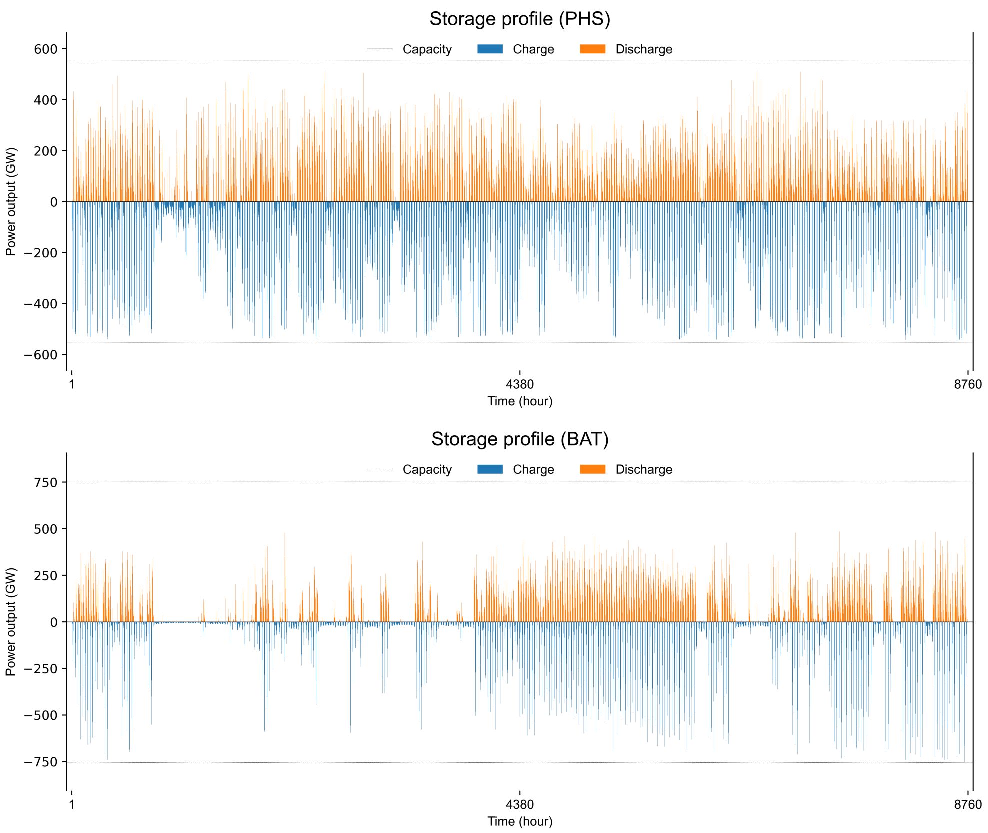

Fig. S123: Storage operation: Neg Emis 400Mt.

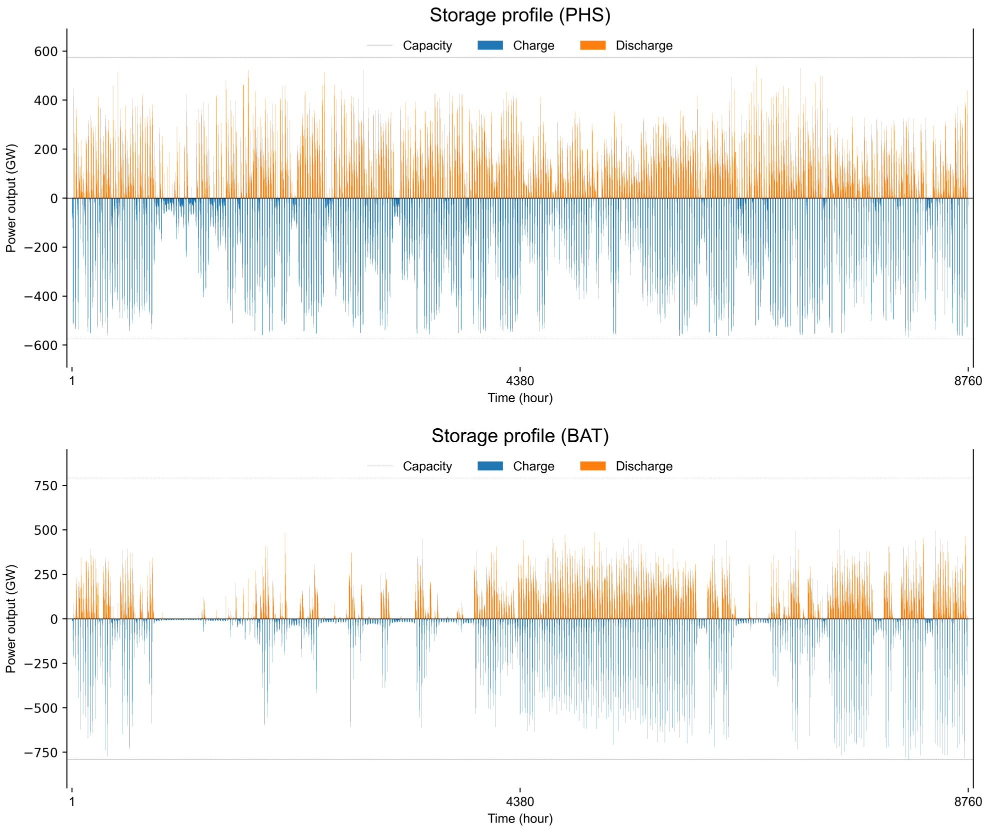

Fig. S124: Storage operation: Neg Emis 200Mt.

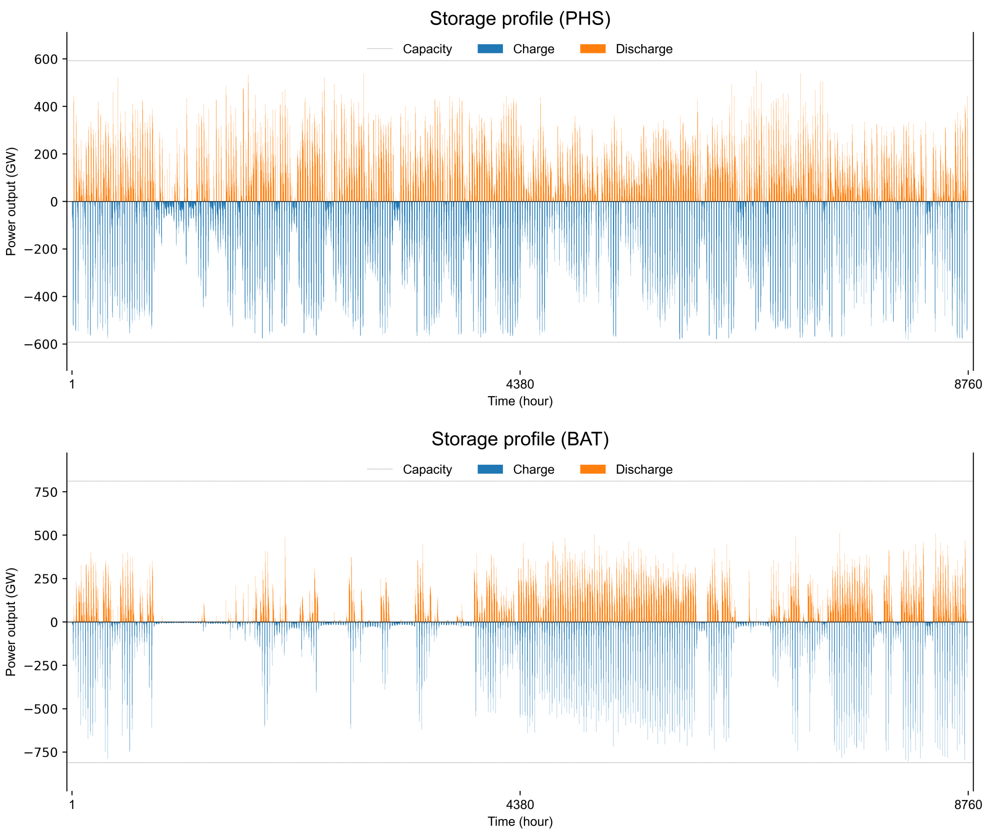

Fig. S125: Storage operation: Net Zero Emis.

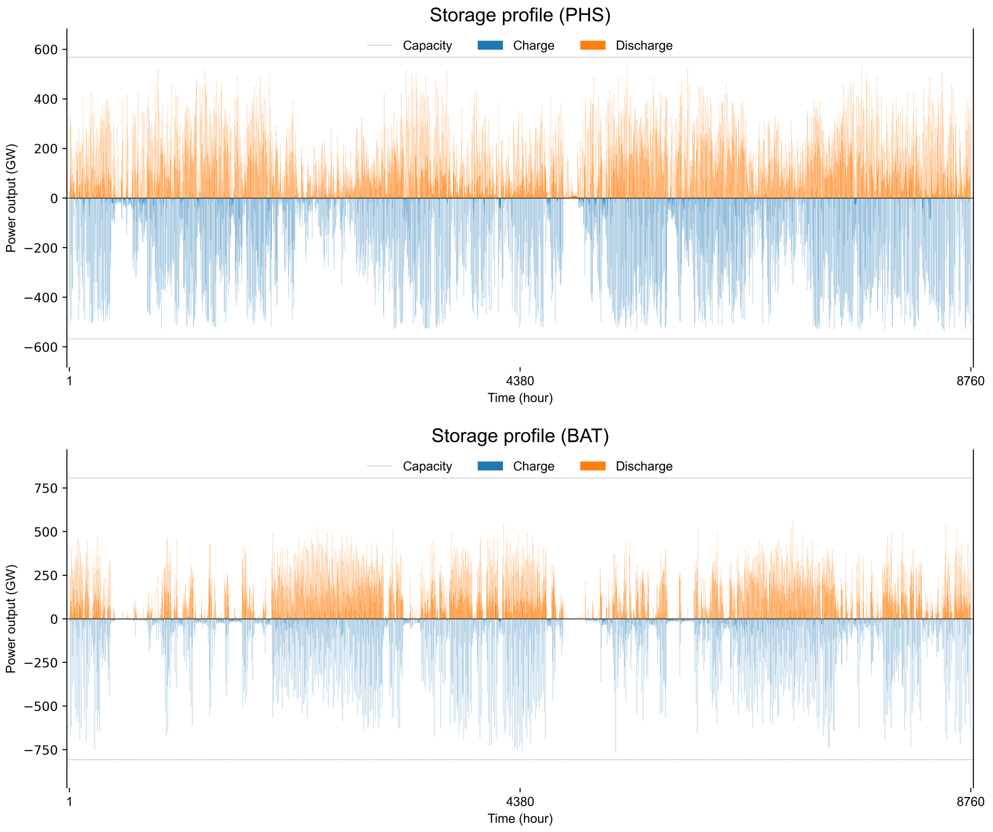

Fig. S126: Storage operation: Two-year period.

6.6 Transmission

6.6.1 UHV utilization and congestion

The annual average transmission line utilization rate is the hourly load (in either direction) divided by the maximum capacity. The congestion rate is defined as the share of congestion hours (i.e., operating at full capacity) over a year. We show these for each inter provincial transmission line in the base case in Fig. S127.

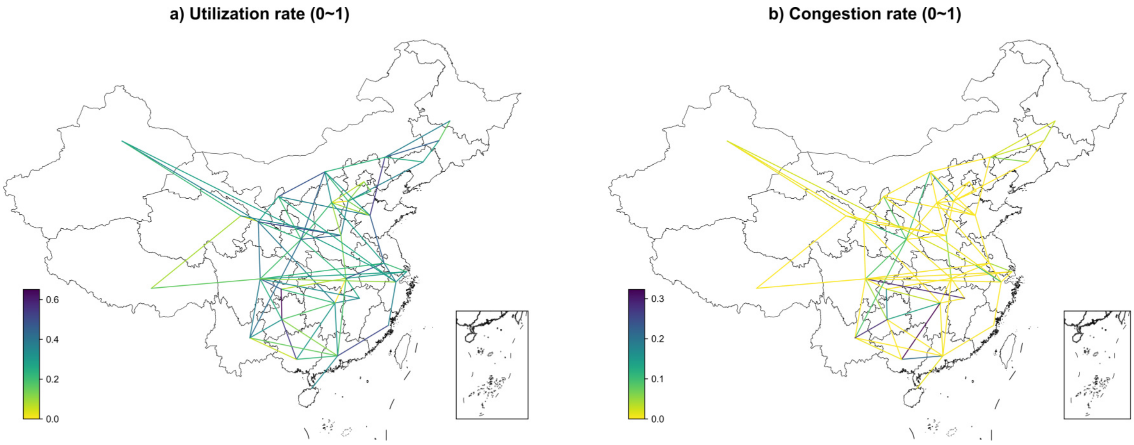

Fig. S127: Utilization and congestion rate of UHV transmission line

6.6.2 Sensitivity results

Newly-built capacity and annual net transmission flow of each interprovincial transmission line are presented from Fig. S128 to S161, respectively.

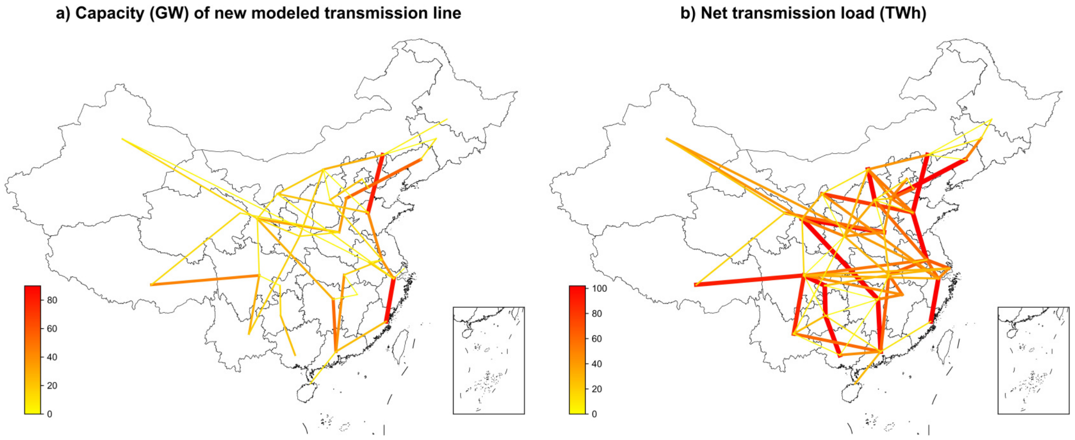

Fig. S128: Newly-built capacity and annual net transmission flow: Base.

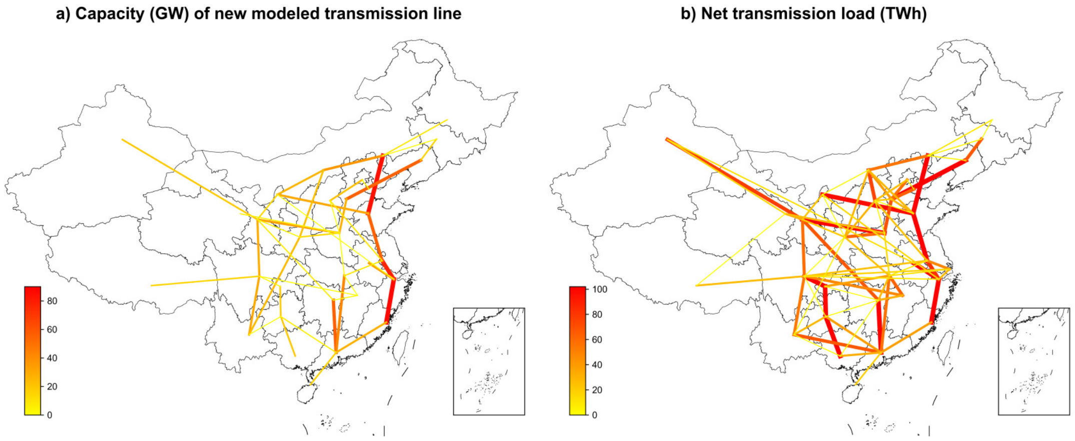

Fig. S129: Newly-built capacity and annual net transmission flow: Wind CapEx 0.5X.

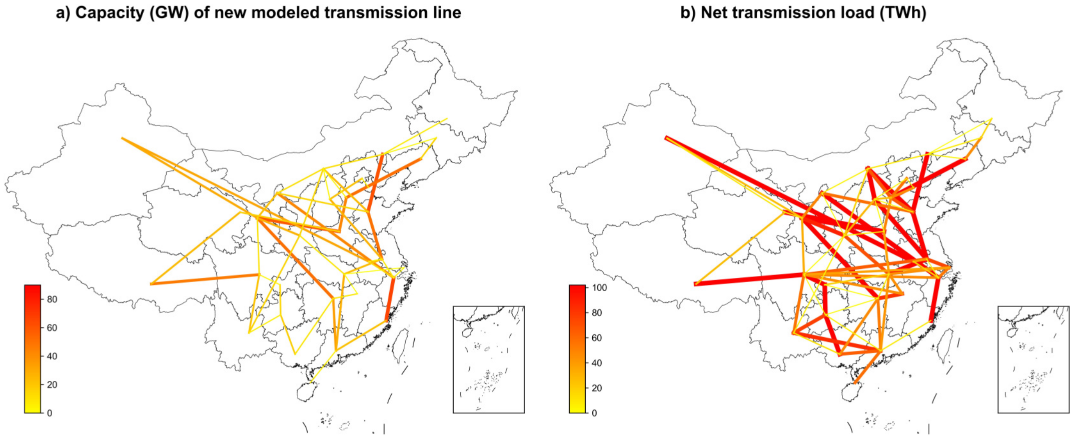

Fig. S130: Newly-built capacity and annual net transmission flow: Wind CapEx 2X.

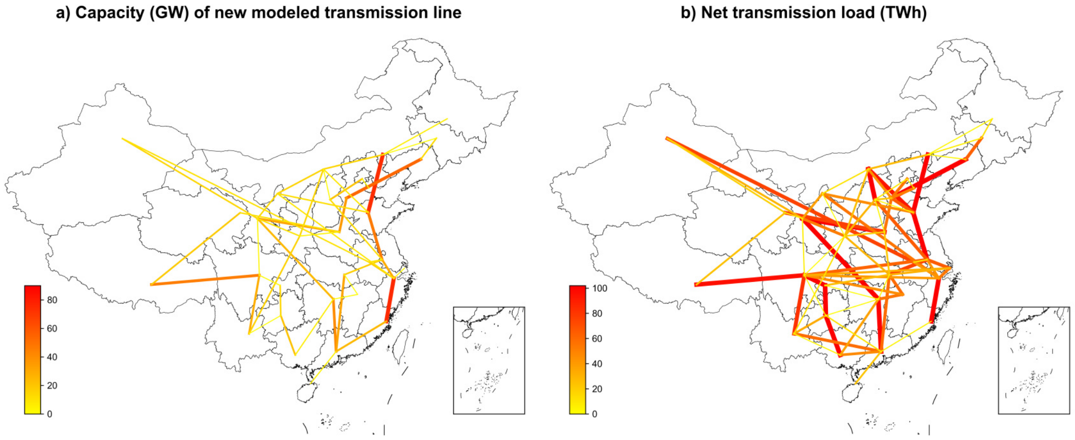

Fig. S131: Newly-built capacity and annual net transmission flow: Solar CapEx 0.5X.

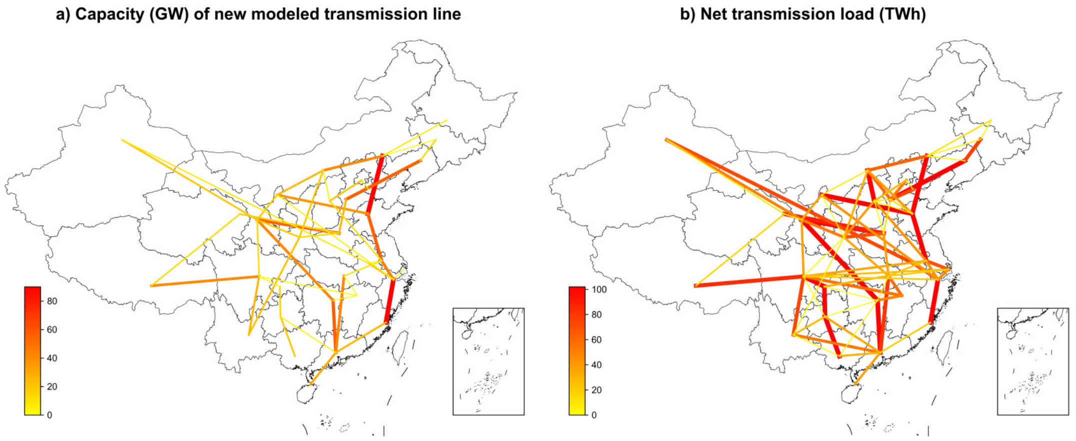

Fig. S132: Newly-built capacity and annual net transmission flow: Solar CapEx 2X.

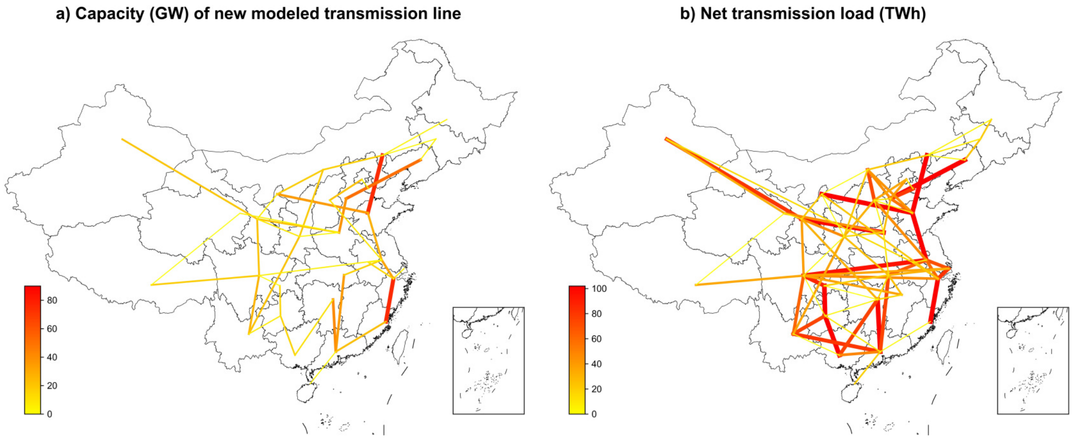

Fig. S133: Newly-built capacity and annual net transmission flow: Wind open + solar open.

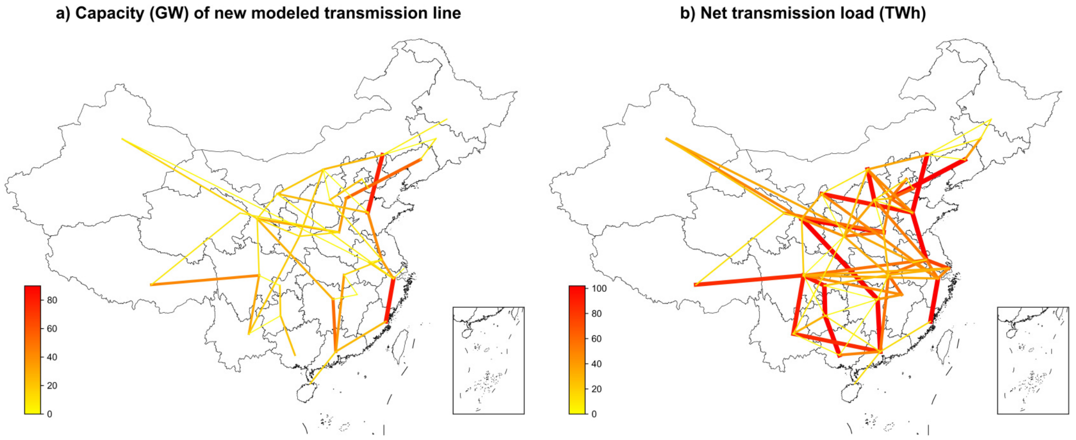

Fig. S134: Newly-built capacity and annual net transmission flow: Wind open + solar base.

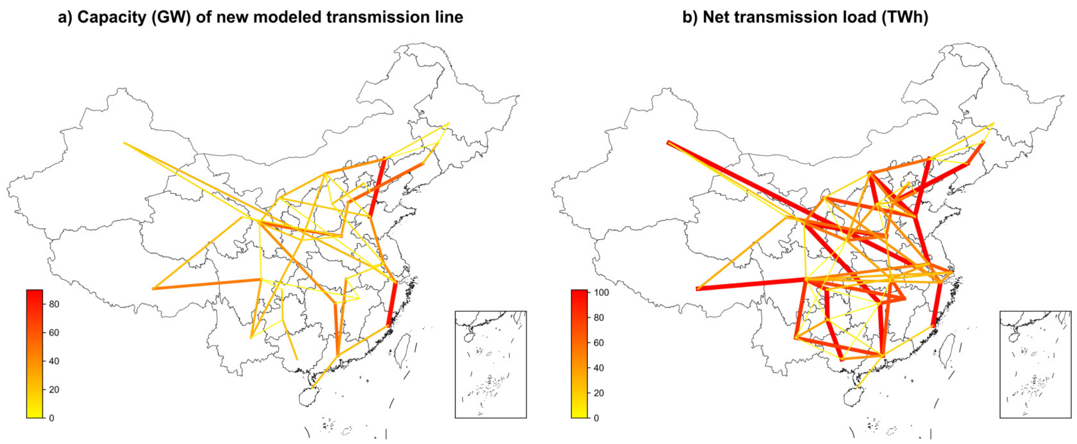

Fig. S135: Newly-built capacity and annual net transmission flow: Wind open + solar conservative.

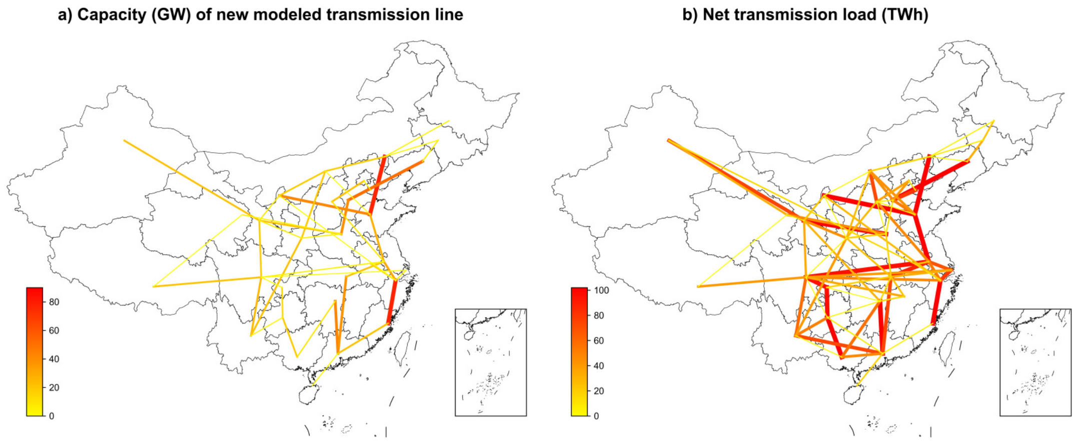

Fig. S136: Newly-built capacity and annual net transmission flow: Wind base + solar open.

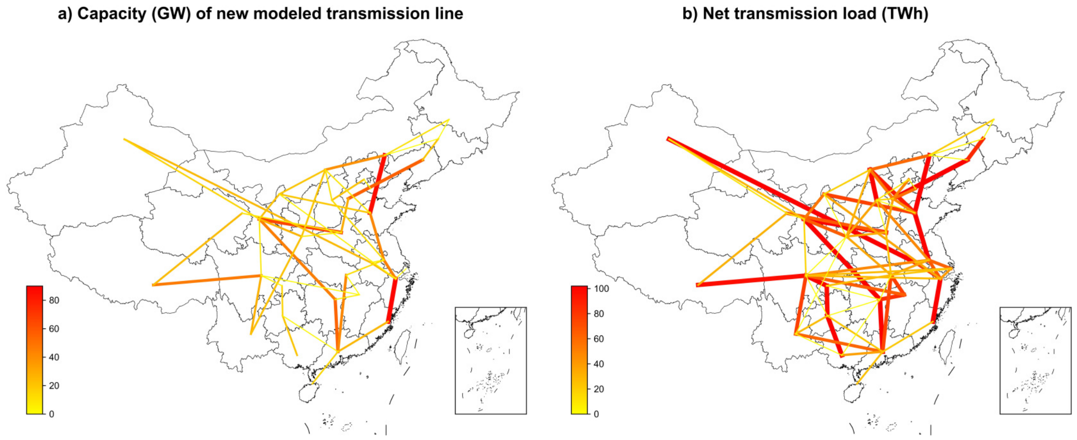

Fig. S137: Newly-built capacity and annual net transmission flow: Wind base + solar conservative.

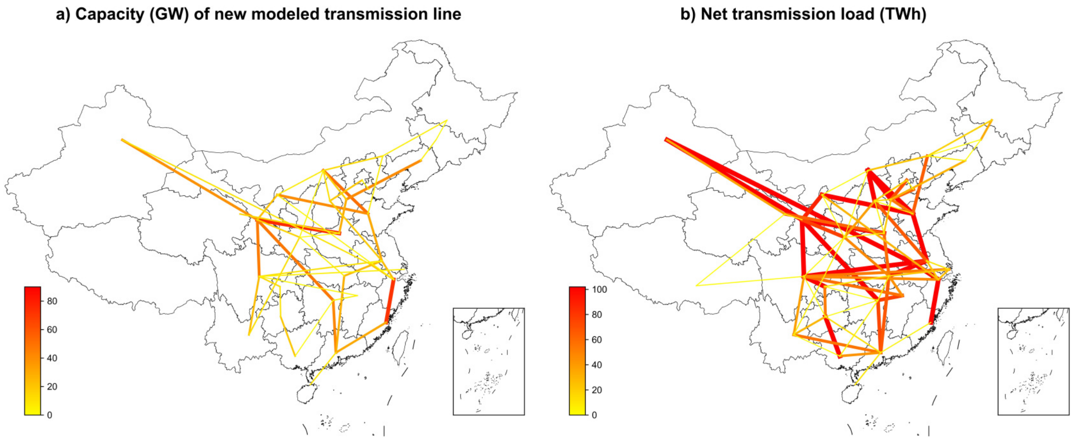

Fig. S138: Newly-built capacity and annual net transmission flow: VRE Cell Clustered.

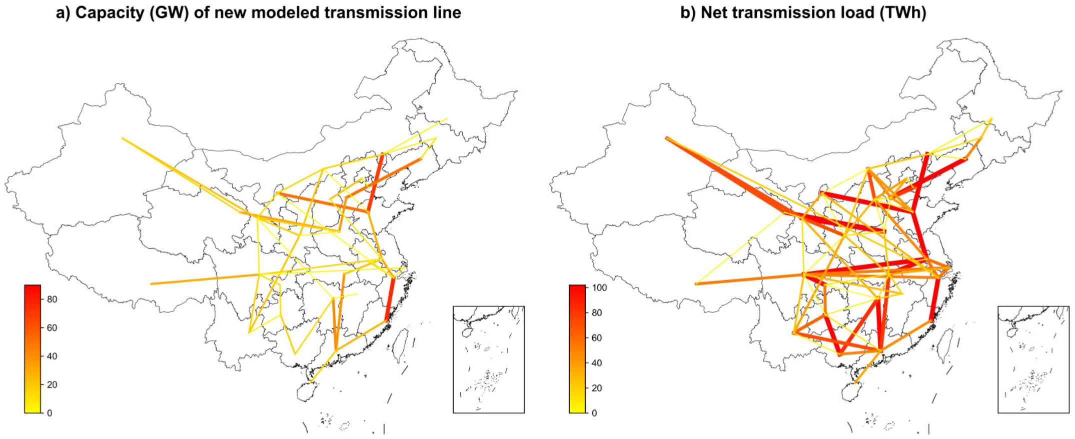

Fig. S139: Newly-built capacity and annual net transmission flow: Wind conservative + solar open.

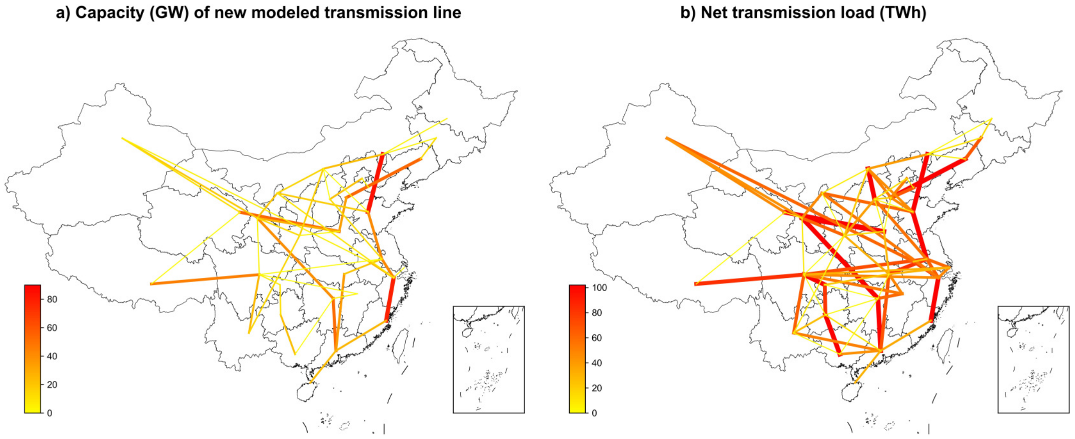

Fig. S140: Newly-built capacity and annual net transmission flow: Wind conservative + solar base.

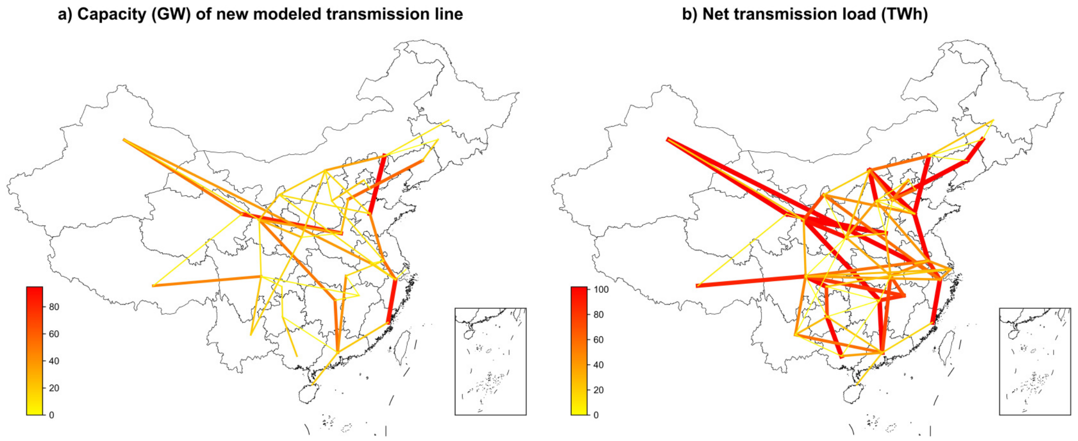

Fig. S141: Newly-built capacity and annual net transmission flow: Wind conservative + solar conservative.

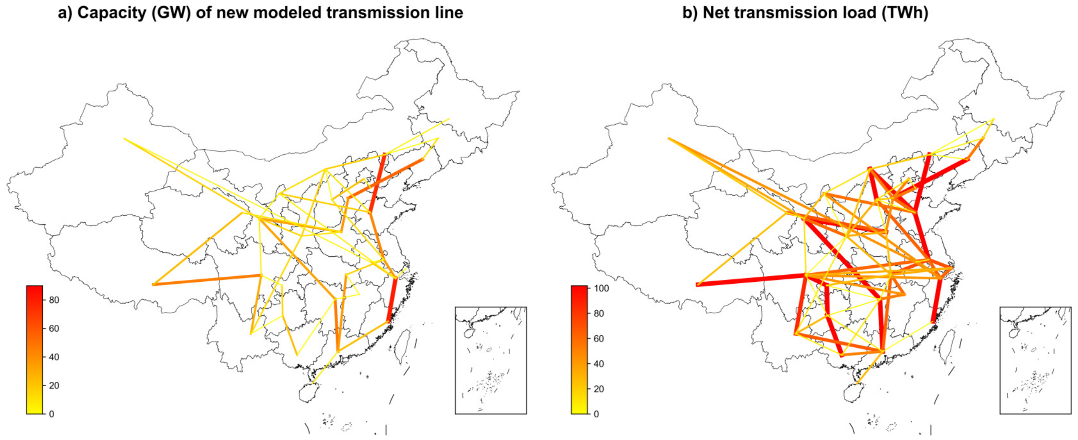

Fig. S142: Newly-built capacity and annual net transmission flow: Battery CapEx 0.5X.

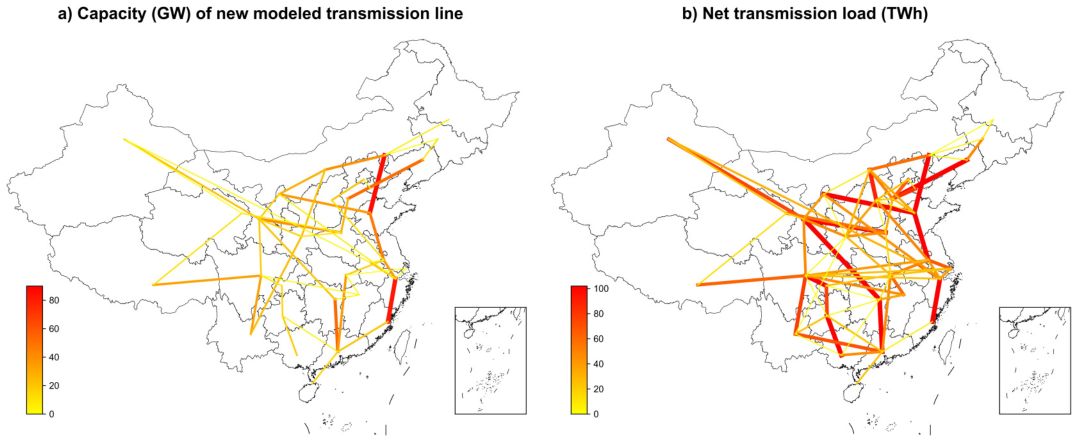

Fig. S143: Newly-built capacity and annual net transmission flow: Battery CAPEX 2X.

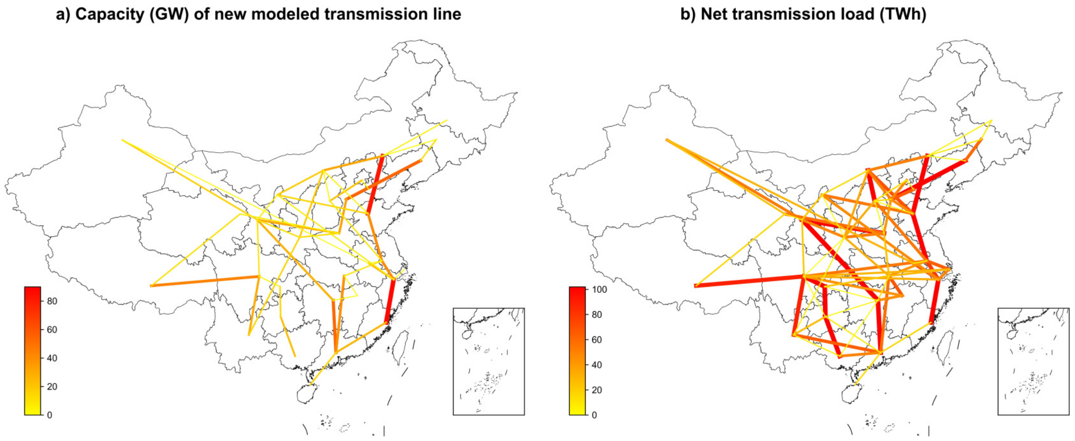

Fig. S144: Newly-built capacity and annual net transmission flow: With CAES.

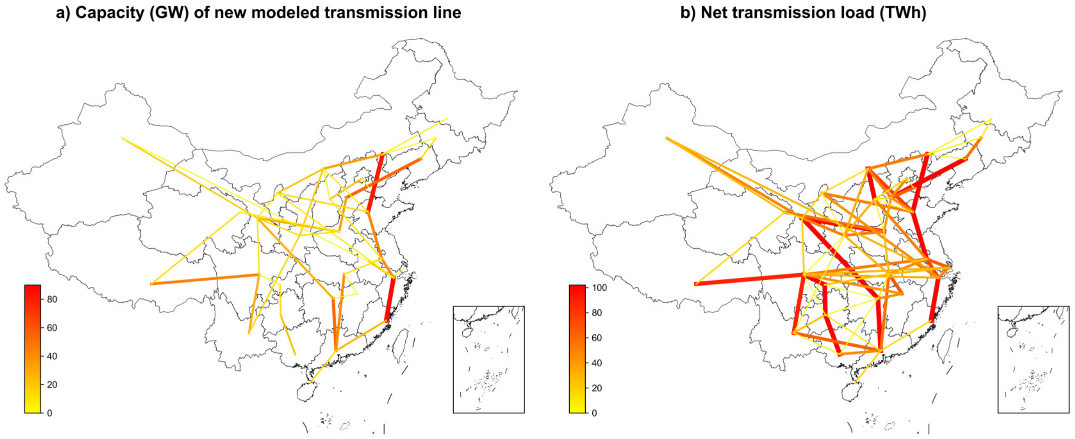

Fig. S145: Newly-built capacity and annual net transmission flow: With VRB.

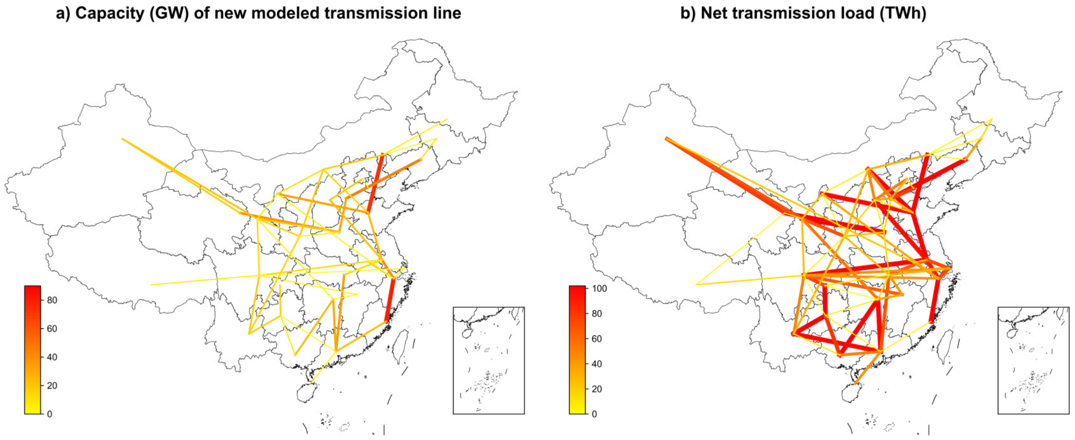

Fig. S146: Newly-built capacity and annual net transmission flow: With CAES and VRB.

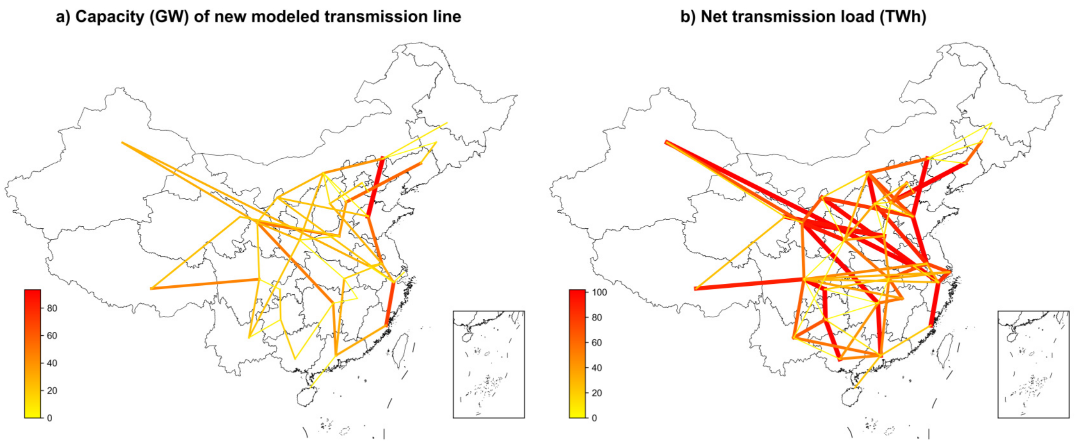

Fig. S147: Newly-built capacity and annual net transmission flow: UHV CapEx 0.5X.

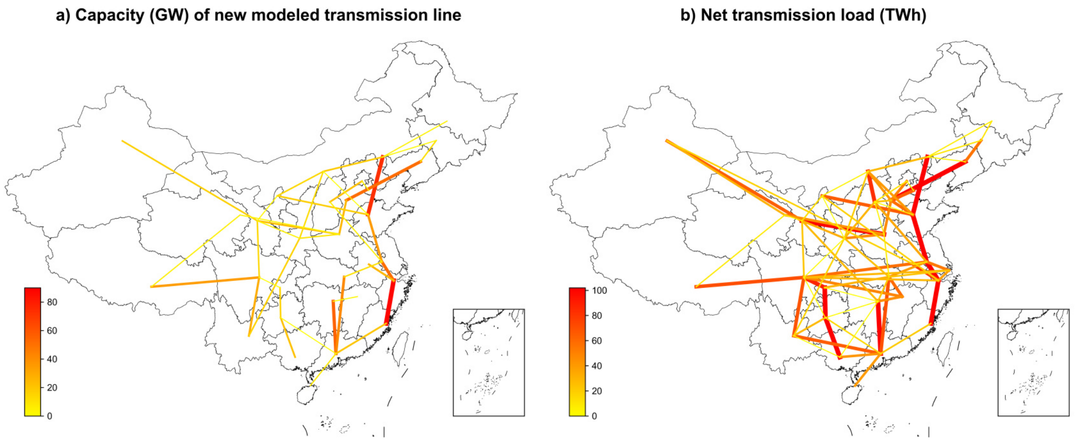

Fig. S148: Newly-built capacity and annual net transmission flow: UHV CapEx 2X.

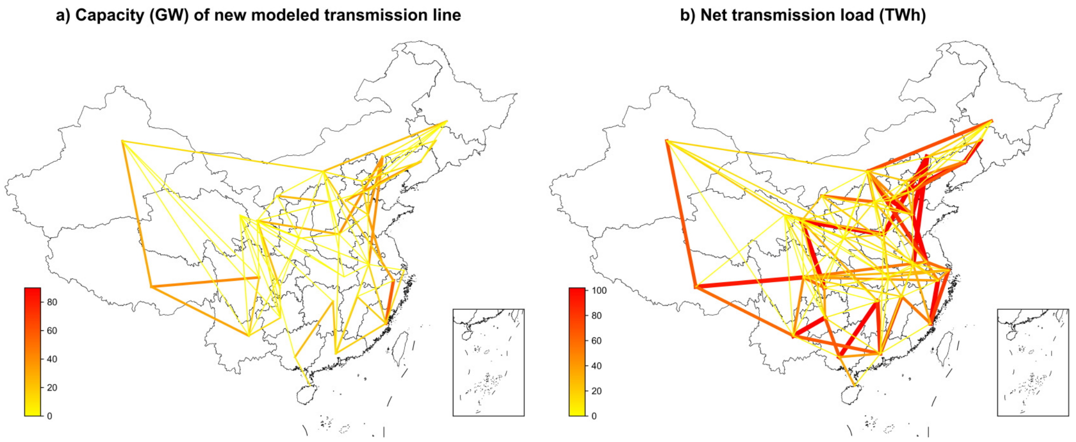

Fig. S149: Newly-built capacity and annual net transmission flow: Unconstrained UHV Expansion.

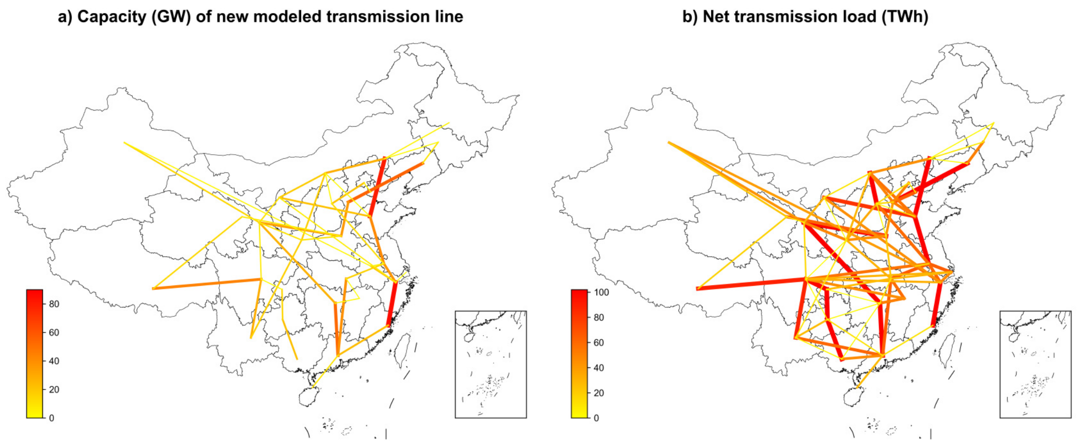

Fig. S150: Newly-built capacity and annual net transmission flow: Resv ratio 2X.

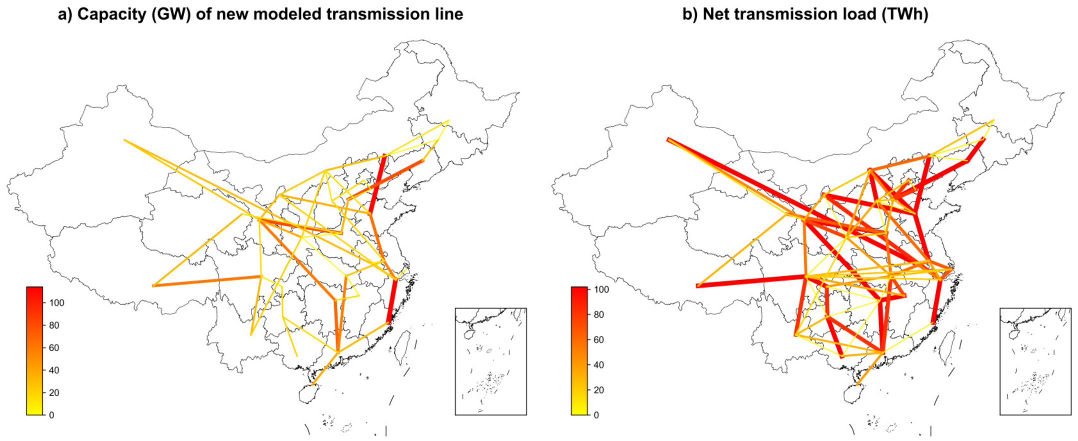

Fig. S151: Newly-built capacity and annual net transmission flow: Demand 1.2X.

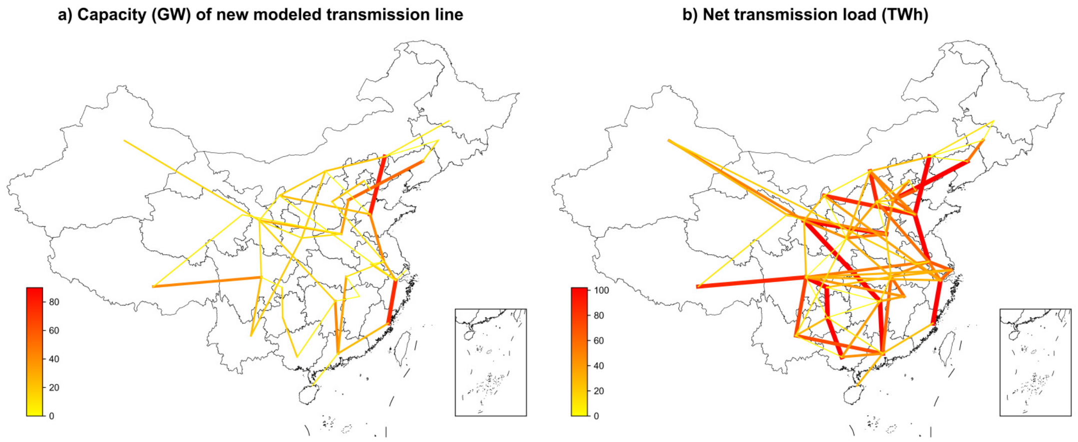

Fig. S152: Newly-built capacity and annual net transmission flow: Coal CCS 350 GW.

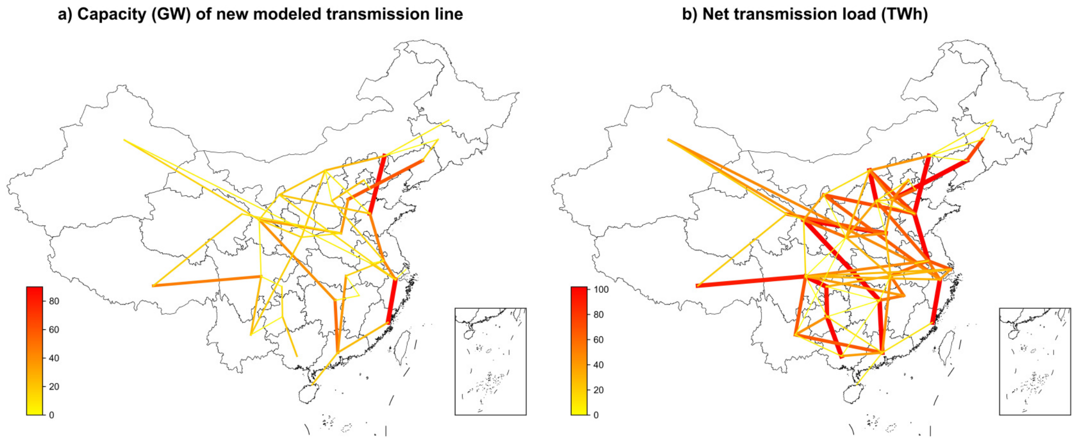

Fig. S153: Newly-built capacity and annual net transmission flow: Nuclear 150GW.

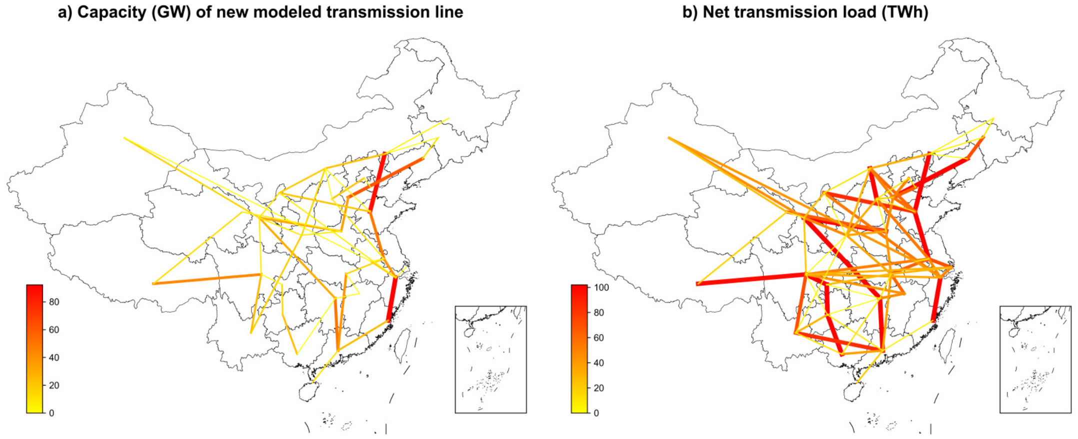

Fig. S154: Newly-built capacity and annual net transmission flow: Nuclear Flex 0.5.

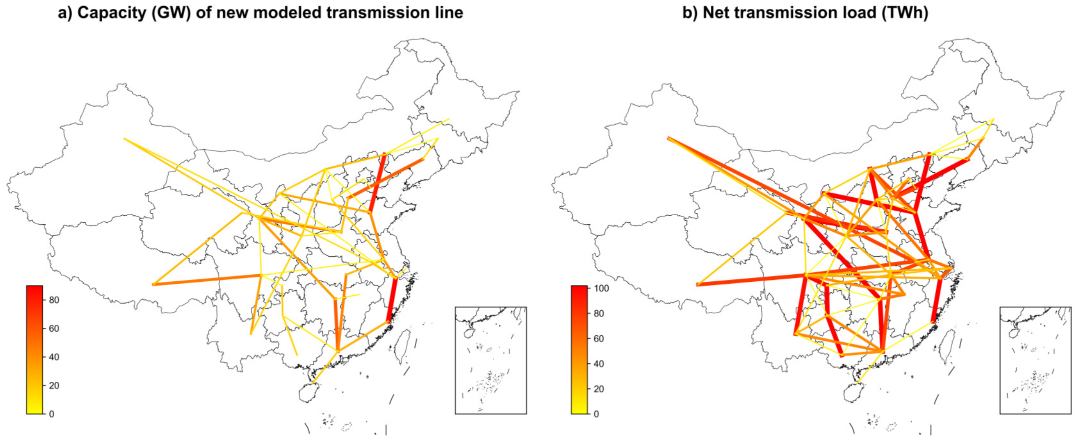

Fig. S155: Newly-built capacity and annual net transmission flow: Gas Capacity 0.5X.

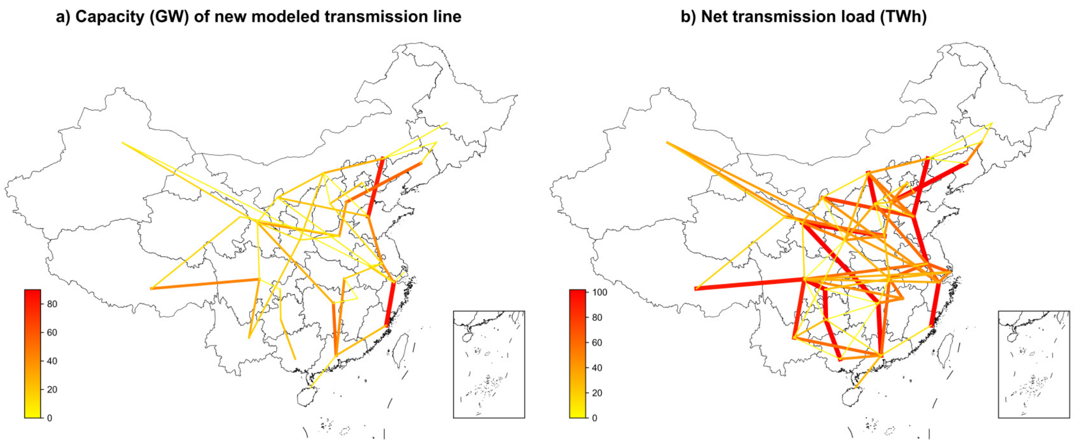

Fig. S156: Newly-built capacity and annual net transmission flow: Gas ramp 0.25.

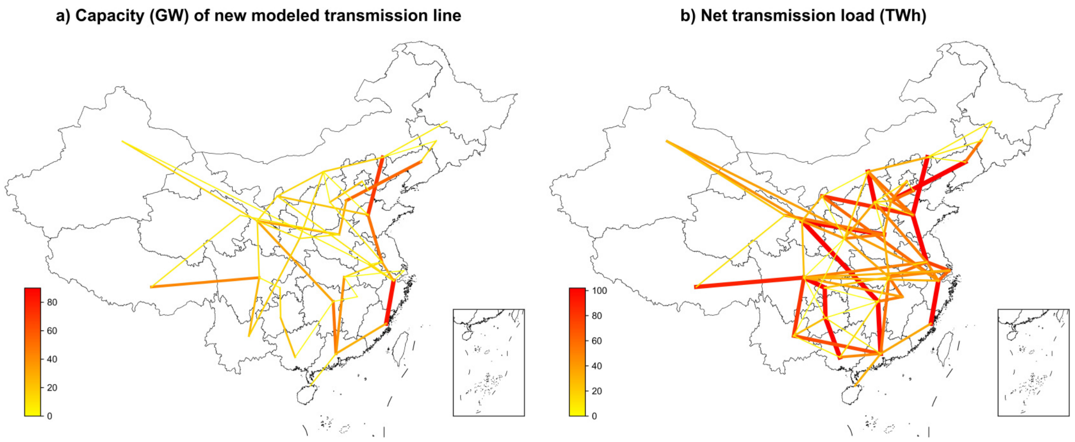

Fig. S157: Newly-built capacity and annual net transmission flow: Load reduction.

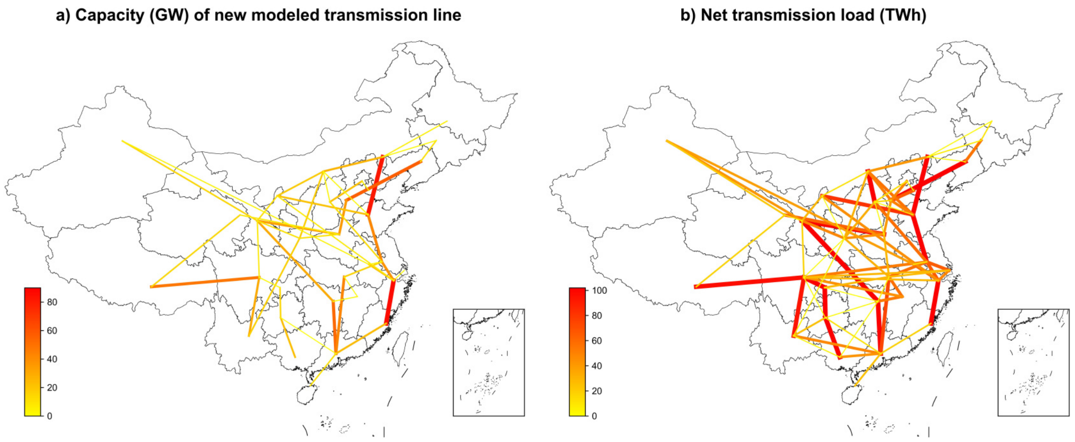

Fig. S158: Newly-built capacity and annual net transmission flow: Neg Emis 400Mt.

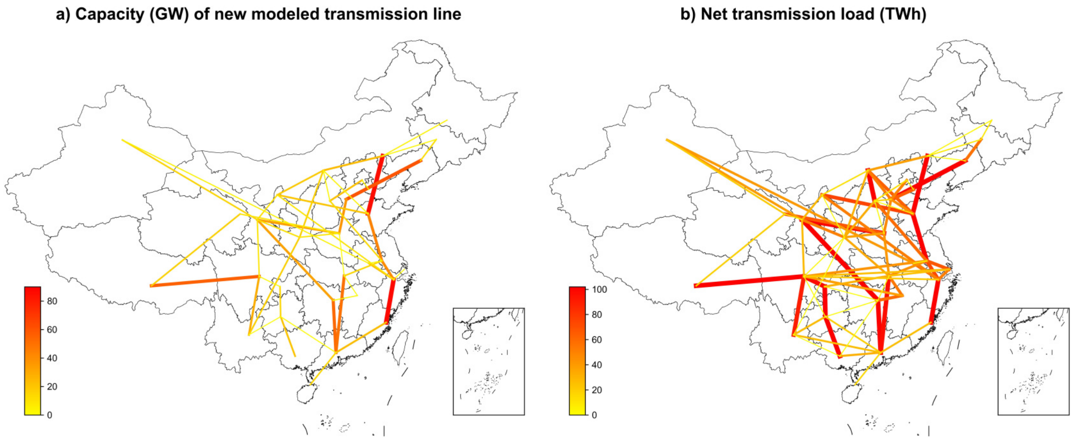

Fig. S159: Newly-built capacity and annual net transmission flow: Neg Emis 200Mt.

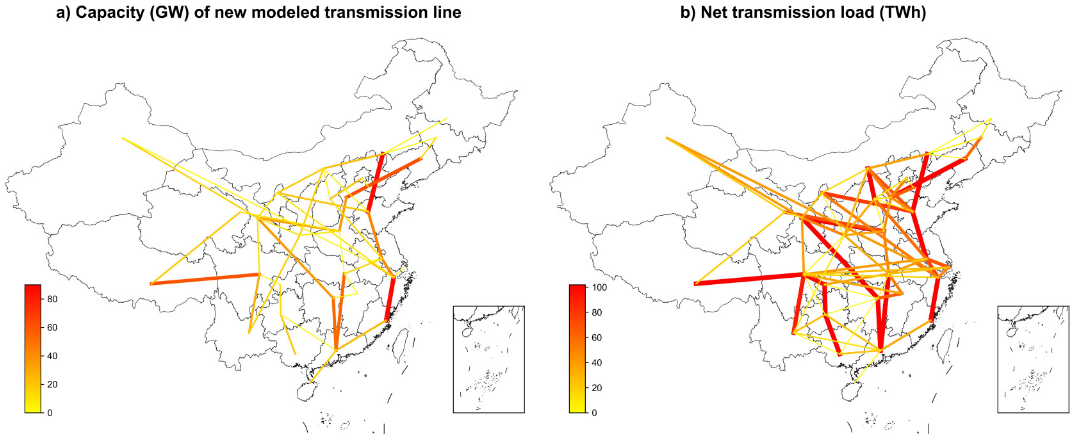

Fig. S160: Newly-built capacity and annual net transmission flow: Net Zero Emis.

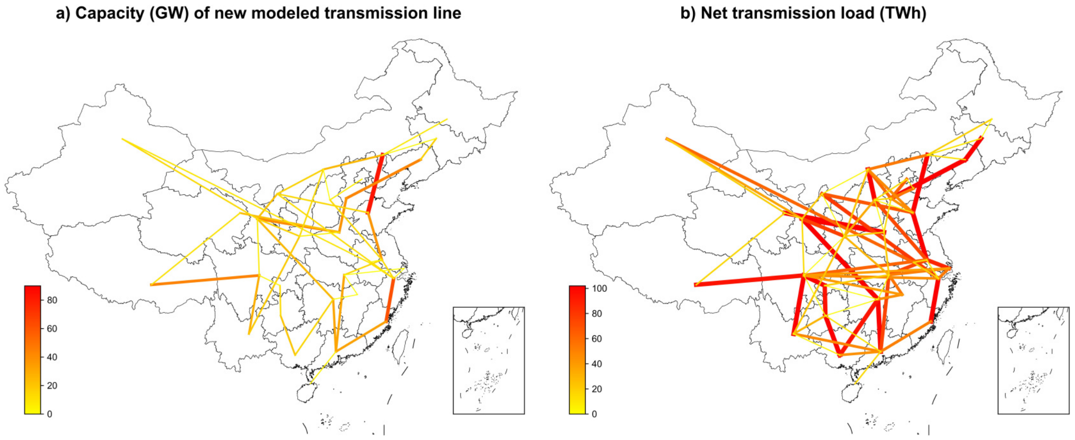

Fig. S161: Newly-built capacity and annual net transmission flow: Two-year period.

References

[1] M.M. Rienecker, M.J. Suarez, R. Todling, J. Bacmeister, L. Takacs, H.-C. Liu, W. Gu, M. Sienkiewicz, R.D. Koster, R. Gelaro, I. Stajner, and J.E. Nielsen. The GEOS-5 Data Assimilation System— Documentation of Versions 5.0.1, 5.1.0, and 5.2.0, 12 2008.

[2] Rob Lucchesi. File Specification for GEOS-5 FP (Forward Processing), 2013.

[3] Cristina L Archer and Mark Z Jacobson. Evaluation of global wind power. *Journal of Geophysical Research: Atmospheres*, 110(D12), 2005.

[4] Xi Lu, Michael B McElroy, Chris P Nielsen, Xinyu Chen, and Junling Huang. Optimal integration of offshore wind power for a steadier, environmentally friendlier, supply of electricity in china. *Energy Policy*, 62:131–138, 2013.

[5] Tianguang Lu, Peter Sherman, Xinyu Chen, Shi Chen, Xi Lu, and Michael McElroy. India's potential for integrating solar and on- and offshore wind power into its energy system. *Nature Communications*, 11, 12 2020.

[6] Shi Chen, Xi Lu, Yufei Miao, Yu Deng, Chris P. Nielsen, Noah Elbot, Yuanchen Wang, Kathryn G. Logan, Michael B. McElroy, and Jiming Hao. The potential of photovoltaics to power the belt and road initiative. *Joule*, 3:1895–1912, 8 2019.

[7] Xi Lu, Shi Chen, Chris P Nielsen, Chongyu Zhang, Jiacong Li, He Xu, Ye Wu, Shuxiao Wang, Feng Song, Chu Wei, Kebin He, Michael B Mcelroy, and Jiming Hao. Combined solar power and storage as cost-competitive and grid-compatible supply for China's future carbon-neutral electricity system. *Proceedings of the National Academy of Sciences of the United States of America*, 2021.

[8] Fei Feng and Kaicun Wang. Does the modern-era retrospective analysis for research and applications-2 aerosol reanalysis introduce an improvement in the simulation of surface solar radiation over China? *International Journal of Climatology*, 39(3):1305–1318, 2019.

[9] World Bank Group. Global Solar Atlas version 2.0. (Solargis, Slovakia), 2021.

[10] Xinliang Xu, Jiyuan Liu, Shuwen Zhang, Rendong Li, Changzhen Yan, and Shixin Wu. China Multi-Temporal Land Use/Cover Dataset (CNLUCC) (in Chinese). Resource and Environmental Science Data Center, 2018. https://www.resdc.cn/DOI/DOI.aspx?DOIID=54.

[11] MLR, NDRC, MST, MIIT, MHURD, and MC. Opinions on supporting the development of new industries and new business forms to promote mass entrepreneurship and innovation land use. [EB/OL], 09 2015. http://www.gov.cn/zhengce/2015-09/18/content_5023796.htm.

[12] Ministry of Land and Resources of the People's Republic of China, Poverty Alleviation Office of the State Council of the People's Republic of China, and National Energy Administration of the People's Republic of China. Opinions on supporting photovoltaic poverty alleviation and regulating

the land use of photovoltaic power generation industry (in Chinese). [EB/OL], 10 2017. http://www.nea.gov.cn/2017-10/10/c_136669687.htm.

[13] Ministry of Land and Resources (MLR) of the People's Republic of China. *Letter from the General Office of the Ministry of Land and Resources on land use for photovoltaic power generation*, 10 2016.

[14] Ministry of Natural Resources of the People's Republic of China. Guidelines for industrial land use policy (2019 version) (in Chinese). [EB/OL], 04 2019. http://www.gov.cn/zhengce/zhengceku/2019-10/14/content_5439551.htm.

[15] State Forestry Administration (SFA) of the People's Republic of China. Notice on issues related to the use of forest land in the construction of photovoltaic power stations (in Chinese), 12 2015. https://www.forestry.gov.cn/main/5925/20200414/090421953922884.html.

[16] Ministry of Water Resources (MWR) of the People's Republic of China. The guiding opinions of the ministry of water resources on strengthening the spatial control of the shoreline of river and lake waters. [EB/OL], 05 2022. http://www.gov.cn/gongbao/content/2022/content_5701574.htm.

[17] The Standing Committee of the National People's Congress of the People's Republic of China. Grassland law of the people's republic of china. [EB/OL], 06 2013. www.mee.gov.cn/ywgz/fgbz/fl/200212/t20021228_81958.shtml.

[18] National Development, Ministry of Land Reform Commission, and State Environmental Protection Administration Resources. Interim measures for the administration of construction land and environmental protection of wind farm projects. [EB/OL], 08 2005. https://zfxxgk.ndrc.gov.cn/upload/images/202210/20221071549665.pdf.

[19] State Forestry and Grassland Administration (SFGA) of the People's Republic of China. Notice on regulating the use of forest land in the construction of wind farm projects. [EB/OL], 02 2019. http://www.gov.cn/zhengce/zhengceku/2019-09/30/content_5435366.htm.

[20] The State Council of the People's Republic of China. Basic farmland protection regulations. [EB/OL], 01 2011. https://www.gov.cn/gongbao/content/2011/content_1860862.htm.

[21] Jing Hu, Robert Harmsen, Wina Crijns-Graus, and Ernst Worrell. Geographical optimization of variable renewable energy capacity in china using modern portfolio theory. *Applied Energy*, 253:113614, 2019.

[22] Diego Silva Herran, Hancheng Dai, Shinichiro Fujimori, and Toshihiko Masui. Global assessment of onshore wind power resources considering the distance to urban areas. *Energy Policy*, 91:75–86, 2016.

[23] Xi Lu and Michael B McElroy. Global potential for wind-generated electricity. In *Wind Energy Engineering*, pages 51–73. Elsevier, 2017.

[24] David EHJ Gernaat, Harmen Sytze de Boer, Vassilis Daioglou, Seleshi G Yalew, Christoph Müller, and Detlef P van Vuuren. Climate change impacts on renewable energy supply. *Nature Climate Change*, 11(2):119–125, 2021.

[25] The National People's Congress of the People's Republic of China. Land Administration Law of the People's Republic of China. [EB/OL], 9 2019. `http://www.npc.gov.cn/npc/c30834/201909/d1e6c1a1eec345eba23796c6e8473347.shtml`.

[26] Ministry of Land and Resources (MLR) of the People's Republic of China. Notice on supporting the development of photovoltaic power generation industry to regulate land management related work (in Chinese). [EB/OL], 2023. `https://www.gov.cn/zhengce/zhengceku/2023-04/03/content_5749824.htm`.

[27] Grassland Law of the People's Republic of China (in Chinese). [EB/OL], 4 2021. `https://flk.npc.gov.cn/detail2.html?ZmY4MDgxODE3YWIyMmI4YTAxN2FiZDVhZDI4NjA1N2E`.

[28] Guiding Opinions of the Ministry of Water Resources on Strengthening the control of shoreline space of river and lake waters (in Chinese). [EB/OL], 5 2022. `https://www.gov.cn/gongbao/content/2022/content_5701574.htm`.

[29] NASA LP DAAC. Modis land cover type yearly global 500 m, 2021.

[30] Hua Zhang. *Research on PV Energy Potential of Rooftop in Urban Area*. PhD thesis, Tianjin University, 5 2016.

[31] Hongbin Gu. China Renewable Energy Development Report 2020 (in Chinese). Technical report, China Renewable Energy Engineering Institution, 6 2021.

[32] Michael R. Davidson, Da Zhang, Weiming Xiong, Xiliang Zhang, and Valerie J. Karplus. Modelling the potential for wind energy integration on China's coal-heavy electricity grid. *Nature Energy*, 1, 7 2016.

[33] Ryan Wiser, Joseph Rand, Joachim Seel, Philipp Beiter, Erin Baker, Eric Lantz, and Patrick Gilman. Expert elicitation survey predicts 37% to 49% declines in wind energy costs by 2050. *Nature Energy*, 6:555–565, 5 2021.

[34] Global Energy Interconnection Development and Cooperation Organization. Research on China's energy and electric power development plan in 2030 and prospect in 2060 (in Chinese), 3 2021.

[35] National Renewable Energy Laboratory. Annual Technology Baseline. [EB/OL]. `https://atb.nrel.gov/electricity/2021/technologies`.

[36] Huaibin Wang, Fang Hu, and Yiwen Liu. Analysis of parity-grid of PV power generation in China. *Solar Energy*, 8:13–18, 8 2021.

[37] Qingyou Yan, Qifeng Wei, and Guangyu Qin. Empirical research on economy of roof-mounted pv power grid connected based on lcow model. *Science Technology and Industry*, 21:73–79, 10 2021.

[38] International Renewable Energy Agency. Renewable power generation costs in 2019, 2020.

[39] Patrick R. Brown and Audun Botterud. The value of inter-regional coordination and transmission in decarbonizing the us electricity system. *Joule*, 5:115–134, 1 2021.

[40] NDRC. Outline of the 14th Five-Year Plan (2021-2025) for National Economic and Social Development and Vision 2035 of the People's Republic of China, 3 2021.

[41] Xiaonan Han, Qiushi Li, and Ke Sun. Comparison Study on Enegy Saving and Consumption Reduction of UHV AC and EHV AC Transmission Technology. *Electric Technology*, 47:145–149, 2 2021.

[42] Xinyu Chen, Yaxing Liu, Qin Wang, Jiajun Lv, Jinyu Wen, Xia Chen, Chongqing Kang, Shijie Cheng, and Michael B. McElroy. Pathway toward carbon-neutral electrical systems in china by mid-century with negative co2 abatement costs informed by high-resolution modeling. *Joule*, 5:2715–2741, 10 2021.

[43] Xiliang Zhang, Xiaodan Huang, Da Zhang, Yong Geng, Lixin Tian, Ying Fan, and Wenyin Chen. Research on the Pathway and Policies for China's Energy and Economy Transformation toward Carbon Neutrality (in Chinese). *Journal of Management World*, 38 (01)(1):35–66, 2022. `http://www.mwm.net.cn/web/xq?leafid=956&docid=4373`.

[44] Building Energy Research Center of Tsinghua University. *2019 annual report on China building energy efficiency (in Chinese)*. China Building Industry Press, 2019.

[45] Institution of Climate Change ans Sustainable Development of Tsinghua University. *China's long-term low-carbon development strategies and pathways comprehensive report*. China Environment Publishing, 2021.

[46] Ministry of Housing and Urban-Rural Development of the People's Republic of China. 2019 statistical yearbook of urban and rural development (in Chinese), 12 2020.

[47] International Energy Agency. The Role of China's ETS in Power Sector Decarbonisation, 2021.

[48] China Electricity Council. *National Power Industry Statistical Data Collection 2019*. China Electricity Council, 1 2020.

[49] Tongwen Shan. Positioning and development path suggestions of natural gas power generation in China's energy transition period. China Offshore Oil And Gas. *POWER DSM*, 02:205–214, 4 2021.

[50] IIASA. ADVANCE Synthesis Scenario Database. `https://www.iiasa.ac.at/web/home/research/researchPrograms/Energy/ADVANCE-Synthesis-Scenario-Database.html`.

[51] Xi Lu, Liang Cao, Haikun Wang, Wei Peng, Jia Xing, Shuxiao Wang, Siyi Cai, Bo Shen, Qing Yang, Chris P. Nielsen, and Michael B. McElroy. Gasification of coal and biomass as a net carbon-negative power source for environment-friendly electricity generation in China. *Proceedings of the National Academy of Sciences of the United States of America*, 116:8206–8213, 2019.

[52] Jingli Fan, Jia Li, Shuiping Yan, Chunjiang Yu, Xian Zhang, Ping Xiao, Tao Wang, Xiaolong Wang, Zihui Zeng, Shuo Sheng, Xianshan Ma, and Mengxiang Fang. Application potential analysis for bioenergy carbon capture and storage technology in China. *Thermal Power Generation*, 50:73–79, 1 2021.

[53] Shiyan Chang, Dingpeng Zheng, and Fu Meng. Bioenergy with carbon capture and storage (BECCS) in the pursuit of the 2°C /1.5°C target. *Journal of Global Energy Interconnection (in Chinese)*, 2:277–287, 2019.

[54] Xiaodan Huang, Shiyan Chang, Dingqian Zheng, and Xiliang Zhang. The role of BECCS in deep decarbonization of China's economy: A computable general equilibrium analysis. *Energy Economics*, 92:104968, 2020.

[55] Rui Wang, Haoran Li, Wenjia Cai, Xueqin Cui, Shihui Zhang, Jin Li, Yuwei Weng, Xinke Song, Bowen Cao, Lei Zhu, et al. Alternative pathway to phase down coal power and achieve negative emission in china. *Environmental Science & Technology*, 56(22):16082–16093, 2022.

[56] Rui Wang, Wenjia Cai, Le Yu, Wei Li, Lei Zhu, Bowen Cao, Jin Li, Jianxiang Shen, Shihui Zhang, Yaoyu Nie, et al. A high spatial resolution dataset of china's biomass resource potential. *Scientific Data*, 10(1):1–15, 2023.

[57] Ming Ren, Chen Huang, Yazhen Wu, Andre Deppermann, Stefan Frank, Petr Havlík, Yuyao Zhu, Chen Fang, Xiaotian Ma, Yong Liu, et al. Enhanced food system efficiency is the key to china's 2060 carbon neutrality target. *Nature food*, pages 1–13, 2023.

[58] Yiming Wei, Jianing Kang, Lancui Liu, Qi Li, Pengtao Wang, Juanjuan Hou, Qiaomei Liang, Hualiao, Shifeng Huang, and Biying Yu. A proposed global layout of carbon capture and storage in line with a 2 °c climate target. *Nature Climate Change*, 11:112–118, 2 2021.

[59] Lee M. Osmonson, Feliks M. Persits, Douglas W. Steinshouer, and Timothy R. Klett. Geologic Provinces of the World (US Geological Survey), 2000. `https://certmapper.cr.usgs.gov/data/wep/dds60/wep_prv.htm`.

[60] Yuanbin Wu and Jing Huang. *Roadmap for carbon capture, utilization and storage technology development in China (2019)*. Science Press, 2019.

[61] Jingli Fan, Jingying Fu, Xian Zhang, Kai Li, Wenlong Zhou, Klaus Hubacek, Johannes Urpelainen, Shuo Shen, Shiyan Chang, Siyue Guo, et al. Co-firing plants with retrofitted carbon capture and storage for power-sector emissions mitigation. *Nature Climate Change*, pages 1–9, 2023.

[62] Johan Carlsson, Roberto Lacal Arantegui, Arnulf Jäger-Waldau, Marika Vellei Bergur Sigfusson, Davide Magagna, Mindaugas Jakubcionis, Maria del Mar Perez Fortes, Stavros Lazarou, Jacopo Giuntoli, Eveline Weidner, Giancarlo de Marco, Amanda Spisto, and Carmen Moles. ETRI 2014 Energy Technology Reference Indicator projections for 2010-2050. Technical report, Electronics and Telecommunications Research Institution, 2014.

[63] China Electricity council. Power industry statistics collection for 2020, 10 2020.

[64] National Energy Administration. Medium and long term development plan of pumped storage (2021-2035). [EB/OL], 8 2021. `http://zfxxgk.nea.gov.cn/1310193456_16318589869941n.pdf`.

[65] Xinjian Xiao and Kejun Jiang. China's nuclear power under the global 1.5 °c target: Preliminary feasibility study and prospects. *Advances in Climate Change Research*, 9:138–143, 6 2018.

[66] Energy Technologies institude. The ETI Nuclear Cost Drivers Project: Summary Report, 2018.

[67] Oliver Schmidt, Sylvain Melchior, Adam Hawkes, and Iain Staffell. Projecting the future levelized cost of electricity storage technologies. *Joule*, 3:81–100, 1 2019.

[68] Haisheng Chen, Thang Ngoc Cong, Wei Yang, Chunqing Tan, Yongliang Li, and Yulong Ding. Progress in electrical energy storage system: A critical review. *Progress in Natural Science*, 19:291–312, 2009.

[69] Fei Cao, Xiangyu Niu, Huijun Li, and Jiejun Zhao. Analysis on operation and maintenance cost of variable speed pumped-storage unit. *Water Power*, 44:96–99, 6 2018.

[70] Behnam Zakeri and Sanna Syri. Electrical energy storage systems: A comparative life cycle cost analysis. *Renewable and sustainable energy reviews*, 42:569–596, 2015.

[71] Wesley Cole, A Will Frazier, and Chad Augustine. Cost Projections for Utility-Scale Battery Storage: 2021 Update. Technical report, 2021.

[72] Todd Aquino, Mathew Roling, Chris Baker, and Lukas Rowland. Battery Energy Storage Technology Assessment. Technical report, Platte River Power Authority, 2017.

[73] Hong Li and Yingchun Lyu. A review on electrochemical energy storage. *Journal of Elctrochem*, 21:412–424, 10 2015.

[74] Guannan He, Jeremy Michalek, Soummya Kar, Qixin Chen, Da Zhang, and Jay F. Whitacre. Utility-scale portable energy storage systems. *Joule*, 5:379–392, 2 2021.

[75] Jacqueline A. Dowling, Katherine Z. Rinaldi, Tyler H. Ruggles, Steven J. Davis, Mengyao Yuan, Fan Tong, Nathan S. Lewis, and Ken Caldeira. Role of long-duration energy storage in variable renewable electricity systems. *Joule*, 4:1907–1928, 9 2020.

[76] Hossein Safaei and David W. Keith. How much bulk energy storage is needed to decarbonize electricity? *Energy and Environmental Science*, 8:3409–3417, 12 2015.

[77] Paul Albertus, Joseph S Manser, and Scott Litzelman. Long-duration electricity storage applications, economics, and technologies. *Joule*, 4(1):21–32, 2020.

[78] Omar J. Guerra, Jiazi Zhang, Joshua Eichman, Paul Denholm, Jennifer Kurtz, and Bri Mathias Hodge. The value of seasonal energy storage technologies for the integration of wind and solar power. *Energy and Environmental Science*, 13:1909–1922, 7 2020.

[79] Nestor A Sepulveda, Jesse D Jenkins, Aurora Edington, Dharik S Mallapragada, and Richard K Lester. The design space for long-duration energy storage in decarbonized power systems. *Nature Energy*, 6(5):506–516, 2021.

[80] Kendall, Vilayanur Viswanathan, Jan Alam, Charlie Vartanian, Vincent Sprenkle, and Richard Baxter Mongird. 2020 Grid Energy Storage Technology Cost and Performance Assessment. Technical report, 12 2020.

[81] International Renewable Energy Agency. Electricity storage: technology brief, 2012.

[82] Rui Shan, Jeremiah Reagan, Sergio Castellanos, Sarah Kurtz, and Noah Kittner. Evaluating emerging long-duration energy storage technologies. *Renewable and Sustainable Energy Reviews*, 159:112240, 2022.

[83] Hongjie He, Ning Zhang, Ershun Du, Yi Ge, and Chongqing Kang. Review on Modeling Method for Operation Efficiency and Lifespan Decay of Large-scale Electrochemical Energy Storage on Power Grid Side. *Automation of Electric Power System*, 44:73–79, 1 2020.

[84] Dong Han, Zenghai Zhao, Bingzhong Yan, Zhenghui Cui, and Yan Ren. Status and prospect of china's pumped storage development in 2021 (in Chinese). *Water power*, pages 1–4, 2022.

[85] Yue Zhang and Zijian Cao. *Analysis report on domestic and foreign power grid development in 2020 (in Chinese)*. China Electric Power Press, 11 2020.

[86] Rubén Romero, A Monticelli, Ae Garcia, and Sérgio Haffner. Test systems and mathematical models for transmission network expansion planning. *IEE Proceedings-Generation, Transmission and Distribution*, 149(1):27–36, 2002.

[87] Zhenyu Zhuo, Ershun Du, Ning Zhang, Chris P Nielsen, Xi Lu, Jinyu Xiao, Jiawei Wu, and Chongqing Kang. Cost increase in the electricity supply to achieve carbon neutrality in china. *Nature Communications*, 13(1):1–13, 2022.

[88] Renshun Wang, Yu Zhao, Fuyuan Ma, Yuzhong Gong, Guangchao Geng, and Quanyuan Jiang. Operational bottleneck analysis and energy storage demand evaluation for high proportion of renewable energy consumption in receiving-end grid. *Power System Technology*, 2021.

[89] Sheng He, Yuting Xu, Songsong Chen, Jindou Yuan, and Feixiang Gong. Prospect and the 14th Five-Year Plan of power demand response development effect in China (in Chinese). *POWER DSM*, 23:1–6, 11 2021.

[90] Notice on Printing and Distributing Implementation Rules of Jiangsu Province Electric Power Demand Response (revised version) (in Chinese). [EB/OL]. http://gxj.yangzhou.gov.cn/yzjingxw/tzgg/201807/ed8d318588104c33b9ba45aef6537238.shtml.

[91] Shandong Energy Bureau. Notification on 2020 Power Demand Response (in Chinese). [EB/OL]. http://fgw.yantai.gov.cn/art/2020/7/8/art_3027_2774771.html.

[92] Zhejiang Development and Reform Commission. Notification on the Implementation of Electricity Demand Response for 2020 (in Chinese). [EB/OL]. http://fzggw.zj.gov.cn/art/2020/7/8/art_1599544_50128806.html.

[93] Anhui Energy Bureau. Notice of Energy Bureau of Anhui Province on Printing and Distributing Anhui Province Electric Power Demand Response Implementation Plan (trial) (in Chinese). [EB/OL]. http://fzggw.ah.gov.cn/public/22554241/146418981.html.

[94] Henan Development and Reform Commission. Notice on the Implementation of Power Demand Response in 2019 (in Chinese). [EB/OL]. http://fgw.henan.gov.cn/2019/04-09/742118.html.

[95] Tianjin Industrial and Information Technology Bureau. Notice of Tianjin Bureau of Industrial and Information Technology on Printing Implementation Rules of Electric Power Demand Response in Summer 2021 (in Chinese). [EB/OL]. http://gyxxh.tj.gov.cn/ZWGK4147/ZCWJ6355/wjwj/202107/t20210705_5495772.html.

[96] Chinese Wind Energy Association. Development of China's wind power industry chain and suggestions (in Chinese), 2022.

[97] China Photovoltaic Industry Association. Annual report on China's PV industry 2020-2021 (in Chinese), 2021.

[98] China Photovoltaic Industry Association. Roadmap for the Development of China's PV Industry, 2021 edition (in Chinese), 2022.

[99] Peng Wang, Wei-Qiang Chen, Xueqin Cui, Jiashuo Li, Wen Li, Chenyang Wang, Wenjia Cai, and Xinyi Geng. Critical mineral constraints in global renewable scenarios under 1.5° c target. *Environmental Research Letters*, 17(12):125004, 2022.

[100] Manish Ram, Dmitrii Bogdanov, Arman Aghahosseini, A Gulagi, AS Oyewo, M Child, U Caldera, K Sadovskaia, J Farfan, LSNS Barbosa, et al. Global energy system based on 100% renewable energy–power, heat, transport and desalination sectors. *Study by Lappeenranta University of Technology and Energy Watch Group, Lappeenranta, Berlin*, 2019. https://ccsi.columbia.edu/sites/default/files/content/docs/EWG_LUT_100RE_All_Sectors_Global_Report_2019.pdf.

[101] Alicia Valero, Antonio Valero, Guiomar Calvo, and Abel Ortego. Material bottlenecks in the future development of green technologies. *Renewable and Sustainable Energy Reviews*, 93:178–200, 2018.

[102] Adrien Fabre, Mouez Fodha, and Francesco Ricci. Mineral resources for renewable energy: Optimal timing of energy production. *Resource and Energy Economics*, 59:101131, 2020.

[103] Norman Toro, Pedro Robles, and Ricardo I Jeldres. Seabed mineral resources, an alternative for the future of renewable energy: A critical review. *Ore Geology Reviews*, 126:103699, 2020.

[104] Ministry of Industry and Information Technology of the People's Republic of China. The development of lithium-ion battery industry in china in 2022, 3 2023. https://wap.miit.gov.cn/gxsj/tjfx/dzxx/art/2023/art_87a66c4fedd047e2a3f4fead23e99718.html.
